# Uncovering the mechanism of Ge-Gen-Qin-Lian decoction for treating ulcerative colitis based on network pharmacology and molecular docking verification

**DOI:** 10.1042/BSR20203565

**Published:** 2021-02-10

**Authors:** Lin Xu, Jiaqi Zhang, Yifan Wang, Zedan Zhang, Fengyun Wang, Xudong Tang

**Affiliations:** 1 China Academy of Chinese Medical Sciences, Beijing, China; 2 Department of Gastroenterology, Xiyuan Hospital of China Academy of Chinese Medical Sciences, Beijing, China; 3 Department of Gastroenterology, Peking University Traditional Chinese Medicine Clinical Medical School (Xiyuan), Beijing, China

**Keywords:** Ge-Gen-Qin-Lian decoction, Molecular docking, Network pharmacology, Traditional Chinese Medicine, Ulcerative colitis

## Abstract

Background: Ge-Gen-Qin-Lian Decoction (GGQLD), a traditional Chinese medicine (TCM) formula, has been widely used for ulcerative colitis (UC) in China, but the pharmacological mechanisms remain unclear. This research was designed to clarify the underlying pharmacological mechanism of GGQLD against UC.

Method: In this research, a GGQLD-compound-target-UC network was constructed based on public databases to clarify the relationship between active compounds in GGQLD and potential targets. Gene ontology (GO) and Kyoto encyclopedia of genes and genomes (KEGG) pathway enrichment analyses were performed to investigate biological functions associated with potential targets. A protein–protein interaction network was constructed to screen and evaluate hub genes and key active ingredients. Molecular docking was used to verify the activities of binding between hub targets and ingredients.

Results: Finally, 83 potential therapeutic targets and 118 corresponding active ingredients were obtained by network pharmacology. Quercetin, kaempferol, wogonin, baicalein, and naringenin were identified as potential candidate ingredients. GO and KEGG enrichment analyses revealed that GGQLD had anti-inflammatory, antioxidative, and immunomodulatory effects. The effect of GGQLD on UC might be achieved by regulating the balance of cytokines (e.g., IL-6, TNF, IL-1β, CXCL8, CCL2) in the immune system and inflammation-related pathways, such as the IL-17 pathway and the Th17 cell differentiation pathway. In addition, molecular docking results demonstrated that the main active ingredient, quercetin, exhibited good affinity to hub targets.

Conclusion: This research fully reflects the multicomponent and multitarget characteristics of GGQLD in the treatment of UC. Furthermore, the present study provided new insight into the mechanisms of GGQLD against UC.

## Introduction

Ulcerative colitis (UC) is an idiopathic chronic inflammatory bowel disease (IBD) characterized by persistent inflammation of the entire large intestine that causes abdominal pain, bloody diarrhea, and fecal urgency [1]. The incidence of UC, especially in Asia, is increasing in the 21st century [2]. While the etiology of UC is still incompletely elucidated, it is thought to be associated with innate and adaptive immunity, gut barrier function, and pathogen sensing and response [3,4]. The treatment of UC mainly includes medical management and colectomy, but current therapies are limited because a large proportion of patients with UC are resistant or intolerant to standard drug treatments [5,6]. Since dissatisfaction with conventional medication, complementary and alternative therapies, including traditional Chinese medicine (TCM), dietary supplements, probiotics, and mind/body interventions [7,8], are commonly used in patients with UC [9,10]. Among them, the therapeutic efficacy and safety of several TCM formulas in IBD have been shown in some double-blind randomized controlled trials [11,12].

Ge-Gen-Qin-Lian decoction (GGQLD), a well-known TCM formula, was first described in Shang Han Lun written by Zhongjing Zhang [13] and has more than 2000 years of clinical application history in China. GGQLD is composed of four herbs: Gegen (Puerariae lobatae Radix), Huangqin (Scutellariae Radix), Huanglian (Coptidis Rhizoma), and Gancao (Glycyrrhizae Radix), at a ratio of 5:3:3:2 [14]. In the clinic, GGQLD has been commonly used for the treatment of diarrhea [15]. Recently, a meta-analysis including 22 trials involving 2028 patients with UC showed that using GGQLD significantly improved the clinical effectiveness and recurrence rate compared with using Western medicine [16]. However, despite the known therapeutic effects of GGQLD, its pharmacological and molecular mechanisms of action have not been fully elucidated.

**Figure 1:**
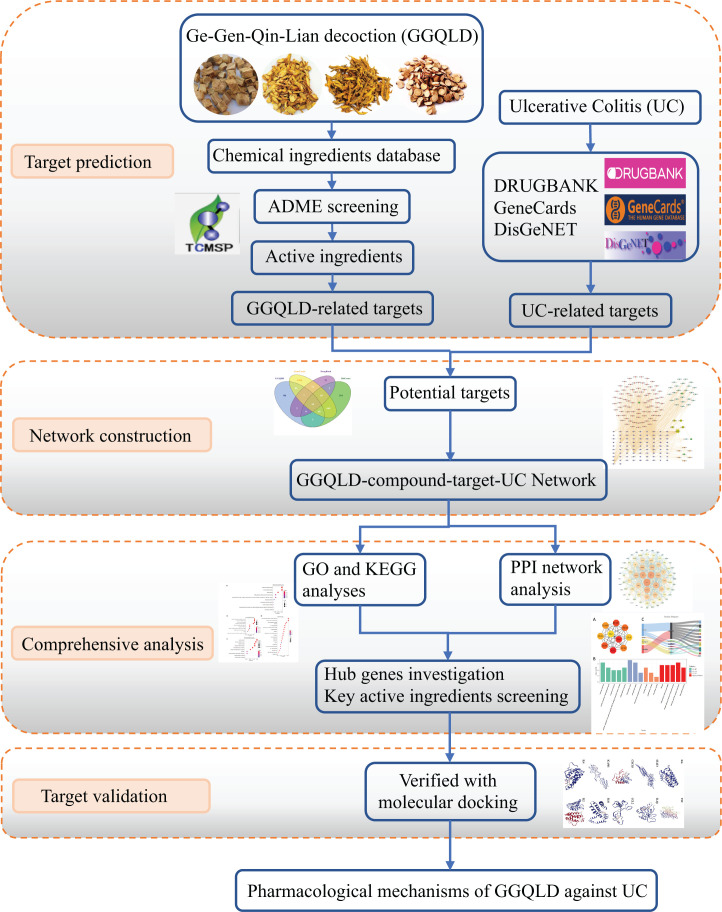
The graphical abstract for underlining the pharmacological mechanisms of GGQLD against UC

Network pharmacology is a part of bioinformatics, integrating bioinformatics, and system medicine. Since network pharmacology can reflect the characteristics of multiple components and targets of TCM, it has been widely used in TCM research and advanced drug discovery in recent years [17–20]. This provides a novel way to clarify the mechanisms of GGQLD that exert therapeutic effects in UC. In the present study, we attempted to undertake a network pharmacology study to unveil the crucial genes and pathways that are involved in the pathogenesis of GGQLD against UC (see Figure 1). These results may provide a new target for the treatment of GGQLD against UC.

## Methods

### Construction of the GGQLD chemical ingredient database

All chemical ingredients of each herb in GGQLD were obtained from the Traditional Chinese Medicine Systems Pharmacology Database and Analysis Platform (TCMSP) (https://tcmspw.com/tcmsp.php) [21]. TCMSP is a unique systems pharmacology platform that integrates large-scale structural data (29,384 chemicals in total with 13,144 unique molecules) with manually curated information for all registered herbs in Chinese pharmacopeia [22]. It can also provide ADME (absorption, distribution, metabolism, and excretion)-related property data, such as human oral bioavailability (OB), half-life (HL), drug-likeness (DL), Caco-2 permeability (Caco-2), blood–brain barrier (BBB), and Lipinski’s rule of five (MW, AlogP, TPSA, Hdon, and Hacc) [23].

### Drug screening and evaluation

Drug screening and evaluation in GGQLD were mainly identified by OB and DL administered by TCMSP. OB and DL are two important indicators in ADME. DL is a qualitative concept used in drug design for how druglikeness a substance is concerning factors such as bioavailability and helps optimize pharmacokinetic and pharmaceutical properties, such as solubility and chemical stability [24]. OB is the fraction of an orally administered drug that reaches systemic circulation, which reveals the convergence of the ADME process. High oral bioavailability is often a key indicator to determine the druglikeness properties of bioactive molecules as therapeutic agents [25]. Then, according to recommendations from the TCMSP, we established preset criteria of OB ≥ 30% and DL ≥ 0.18 to screen for the possible bioactive ingredients in GGQLD.

### GGQLD-related target prediction

The corresponding protein targets of each active ingredient in GGQLD were predicted using TCMSP. Predicted targets from TCMSP were extracted and converted to gene names using UniProtKB (http://www.uniprot.org). Target information was set to *Homo sapiens* and aggregated for further analysis.

### UC-related gene database construction

UC-related genes were retrieved from the DisGeNET database (https://www.disgenet.org/), GeneCards database (https://www.genecards.org/), and DrugBank database (https://www.drugbank.ca/). DisGenNET is a knowledge management platform integrating and standardizing data about disease-associated genes and variants from multiple sources. It also covers the full spectrum of human diseases as well as normal and abnormal traits [26,27]. The GeneCards database is a one-stop shop for searchable human gene annotations [28,29]. DrugBank is a web-enabled database containing comprehensive molecular information about drugs, their mechanisms, their interactions, and their targets [30]. DrugBank was searched for known UC drugs/drug-like compounds that were approved by the United States Food and Drug Administration (FDA) for clinical trials. Finally, the common targets shared by at least two databases were retained as UC-related targets, while the other targets were removed.

### Construction of a GGQLD-compound-target-UC network for GGQLD

Intersections of GGQLD targets and UC-related targets were regarded as potential targets of GGQLD for the treatment of UC. The corresponding chemical compounds of the intersecting targets were thought to be possible therapeutic components that affect UC. The potential targets were obtained by a Venn diagram. A GGQLD-compound-target-UC (G-U) network was constructed to clarify the relationship between active compounds in GGQLD and potential targets. This network was constructed and visualized using Cytoscape 3.7.2 software.

### Functional enrichment analysis of GGQLD-related targets

To investigate biological functions associated with potential targets, we performed Gene Ontology (GO) and Kyoto Encyclopedia of Genes and Genomes (KEGG) pathway enrichment analyses via the ‘clusterProfiler’ R package. The ‘clusterProfiler’ R package serves as a user-friendly enrichment tool with integrated gene cluster analysis based on multiple resources [31]. GO enrichment analysis was used to explain and annotate genes by three dimensions, including cellular component (CC), molecular function (MF), and biological process (BP) analyses. The KEGG database was mainly used for pathway analysis. In addition, at least three genes were contained, and *P*-values below 0.05 were considered statistically significant.

### Protein–protein interaction network between targets in the G-U network

Protein–protein interaction (PPI) is of pivotal importance in the regulation of biological systems and is consequently implicated in the development of disease states [32]. The potential targets were introduced into the STRING database (https://string-db.org/) to perform PPI analysis. STRING, an online PPI analysis database, currently features the largest number of organisms (5090) and proteins (24.6 million) and has very broad and diverse, benchmarked data sources [33]. The species was limited to ‘*Homo sapiens*’, and the minimum required interaction score was set to be high confidence (0.700). The PPI network was visualized with Cytoscape 3.7.2 software.

### Hub gene and key active compound screening and evaluation

To further identify the hub genes in the potential targets, the top 10 hub genes were identified using MCC methods in ‘cytoHubba’ of Cytoscape 3.7.2 software [34]. Furthermore, GO and KEGG pathway analyses of hub genes were performed via the Database for Annotation, Visualization and Integrated Discovery (DAVID) (https://david.ncifcrf.gov/), a web-based analysis tool. Gene counts >3 and a *P*-value below 0.05 were considered as the cutoff criteria. To reveal the relationship between hub genes and possible therapeutic components, the corresponding chemical compounds of hub genes were incorporated into a Sankey diagram.

### Verification through molecular docking

Molecular docking was used to predict the activities of binding of proteins to compounds via CB-Dock (http://cao.labshare.cn/cb-dock/). CB-Dock is a protein-ligand docking method that automatically identifies the binding sites, calculates the center and size, customizes the docking box size according to the query ligands, and then performs molecular docking with AutoDock Vina [35]. The PDB formats of proteins were derived from the RCSB PDB database (https://www.rcsb.org/), and SDF formats of ligands were obtained from the PubChem database (https://pubchem.ncbi.nlm.nih.gov/), respectively. The styles of the ligand and receptor were set as ‘ball and stick’ and ‘cartoon’, respectively. The colors of the ligand and receptor were set as ‘By Element’ and ‘By Chain’, respectively. The vina sore represents a certain binding activity between a ligand and a protein. The lower the vina score is, the more stable the ligand binds to the protein.

## Results

### Bioactive compounds in GGQLD

Bioactive ingredients in GGQLD and corresponding ADME information were extracted from the TCMSP data server. In total, 154 bioactive compounds were identified in GGQLD. After filtering by druglikeness threshold (OB ≥ 30% and DL ≥ 0.18), a total of 139 ingredients were identified, including 14 components of Huanglian, 35 components of Huang Qin, 4 components of Gegen, and 92 components of Gancao. Some compounds are shared by two or more herbs, such as quercetin (MOL000098), which is shared by Huanglian and Gancao; beta-sitosterol (MOL000358), which is shared by Gegen and Huang Qin; coptisine (MOL001458) and epiberberine (MOL002897), which are shared by Huanglian and Huang Qin; and formononetin (MOL000392), which is shared by Gegen and Gancao. Detailed information regarding these ingredients is shown in Supplementary Table S1.

### Identification of targets in GGQLD

Potential targets in GGQLD were predicted in TCMSP. In total, 259 targets were identified for 139 bioactive compounds (4 compounds did not receive predictions). Afterward, predicted targets from the TCMSP were extracted and converted to gene names using UniProtKB. From the results, we can see that many compounds were predicted to target the same proteins, such as 62 ingredients targeted to PPARG.

### Identification of UC-related targets

The identification of UC-related targets was searched using the keyword ‘ulcerative colitis’ from the following resources: DisGeNET database, GeneCards database, and DrugBank database. A total of 5273 genes were searched, including 914 genes from the DisGeNET database, 4356 genes from the GeneCards database, and 64 genes from the DrugBank database, and 17 FDA-approved compounds were identified for the treatment of UC. As a result, there are 776 common targets shared by at least two databases, which were retained as UC-related targets.

### Construction of a GGQLD-compound-target-UC network

**Figure 2:**
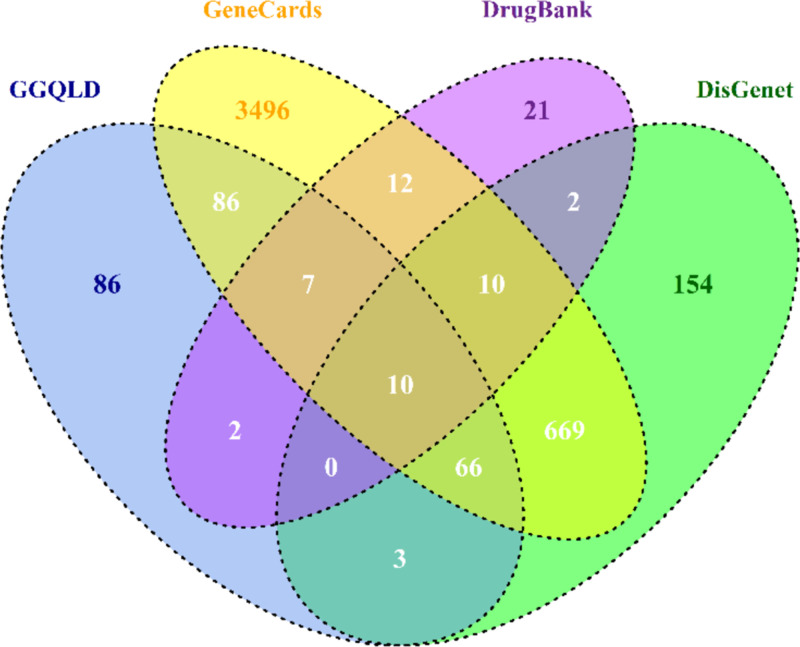
Venn diagram Venn diagram showing the numbers of overlapping and specific genes among the four databases (GGQLD, DisGenet, GeneCards, and DrugBank). The overlapping genes were selected for further analysis.

Based on the GGQLD target database and UC-related target database, 83 overlapping genes were identified as potential targets for GGQLD against UC, as shown in the Venn diagram (Figure 2). The results showed that 10 genes, namely, ABCG2, CD40LG, CYP3A4, IFNG, MPO, NOS2, NR1I2, PPARG, PTGS1, and PTGS2, overlapped from the four databases (GGQLD, DisGenet, GeneCards, DrugBank). Detailed information regarding these targets is shown in Supplementary Table S2.

**Figure 3:**
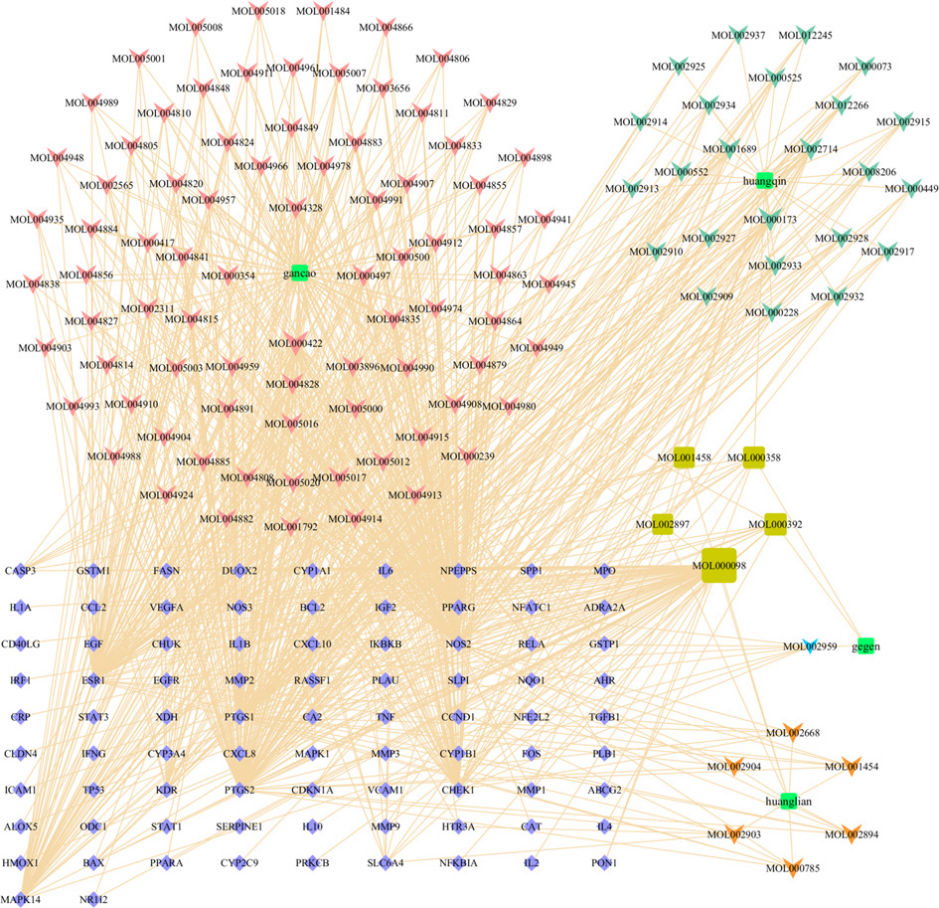
The G-U network The green squares represent the drug; the purple diamonds represent the potential targets; the arrows represent the drug ingredients; and the olive square indicates shared ingredients. The colors indicate different herbs. The line between two nodes represents the interaction, and the size of each node indicates the number of connections.

There were 118 active ingredients corresponding to the 83 potential targets. To better understand the complex relationships between all bioactive compounds in GGQLD and their UC-related targets, a G-U network was constructed (Figure 3). There were 205 nodes in the G-U network composed of 118 active ingredients and 83 UC targets connected by 866 interactions. In addition, as shown in Figure 3, node size indicated the number of connections calculated using ‘NetworkAnalyzer’, an analytical tool in Cytoscape 3.7.2. The top five ingredients were quercetin, kaempferol, wogonin, baicalein, and naringenin. The details of the five ingredients are described in Table 1. The details of the G-U network are listed in Supplementary Table S3.

**Table 1:** The top five ingredients in the G-U network

Mol ID	Molecule name	MW	AlogP	Hdon	Hacc	OB (%)	Caco-2	BBB	DL	FASA-	HL	Chemical structure
MOL000098	Quercetin	302.25	1.5	5	7	46.43	0.05	-0.77	0.28	0.38	14.4	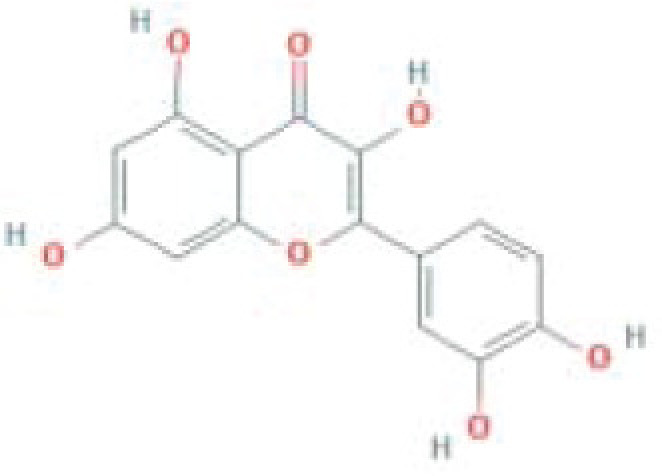
MOL000422	Kaempferol	286.25	1.77	4	6	41.88	0.26	-0.55	0.24	0	14.74	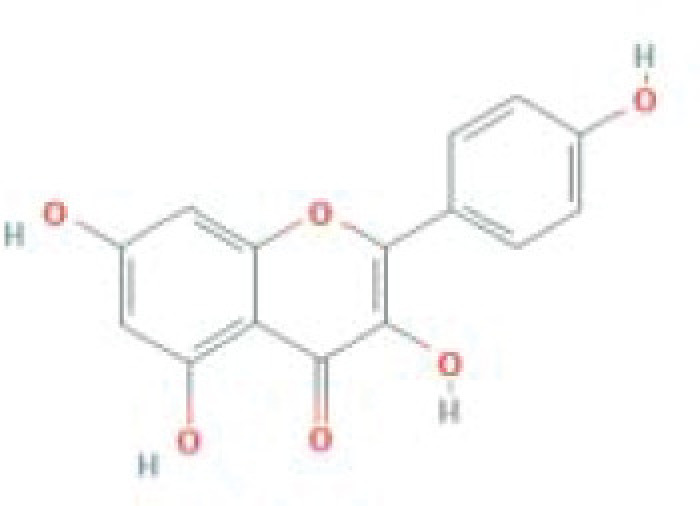
MOL000173	Wogonin	284.28	2.59	2	5	30.68	0.79	0.04	0.23	0.32	17.75	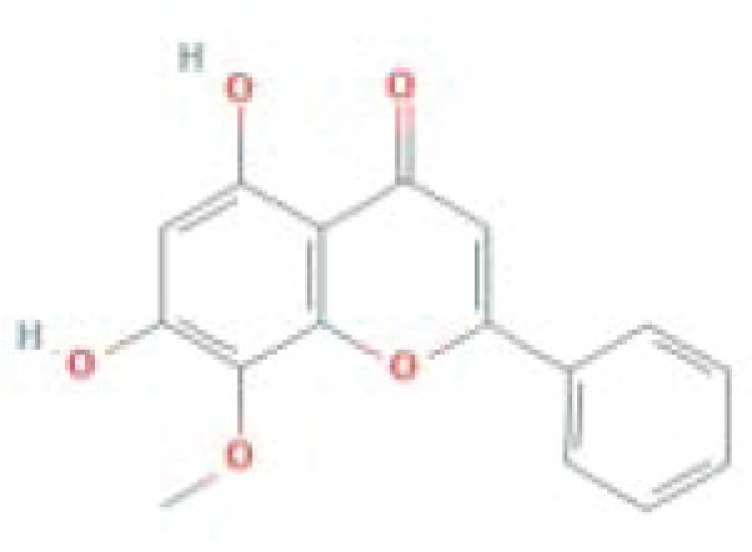
MOL002714	Baicalein	270.25	2.33	3	5	33.52	0.63	-0.05	0.21	0.36	16.25	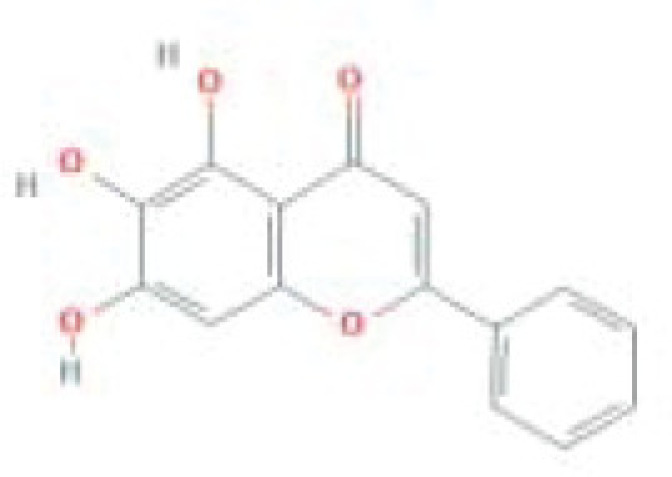
MOL004328	Naringenin	272.27	2.3	3	5	59.29	0.28	-0.37	0.21	0.4	16.98	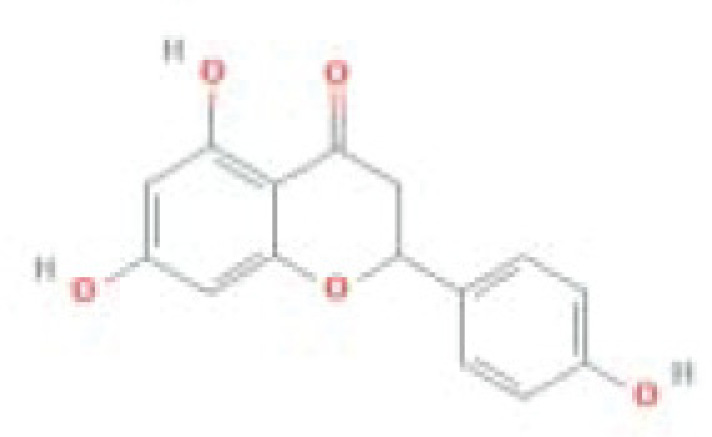

### GO and KEGG pathway enrichment analyses of the G-U network

**Figure 4:**
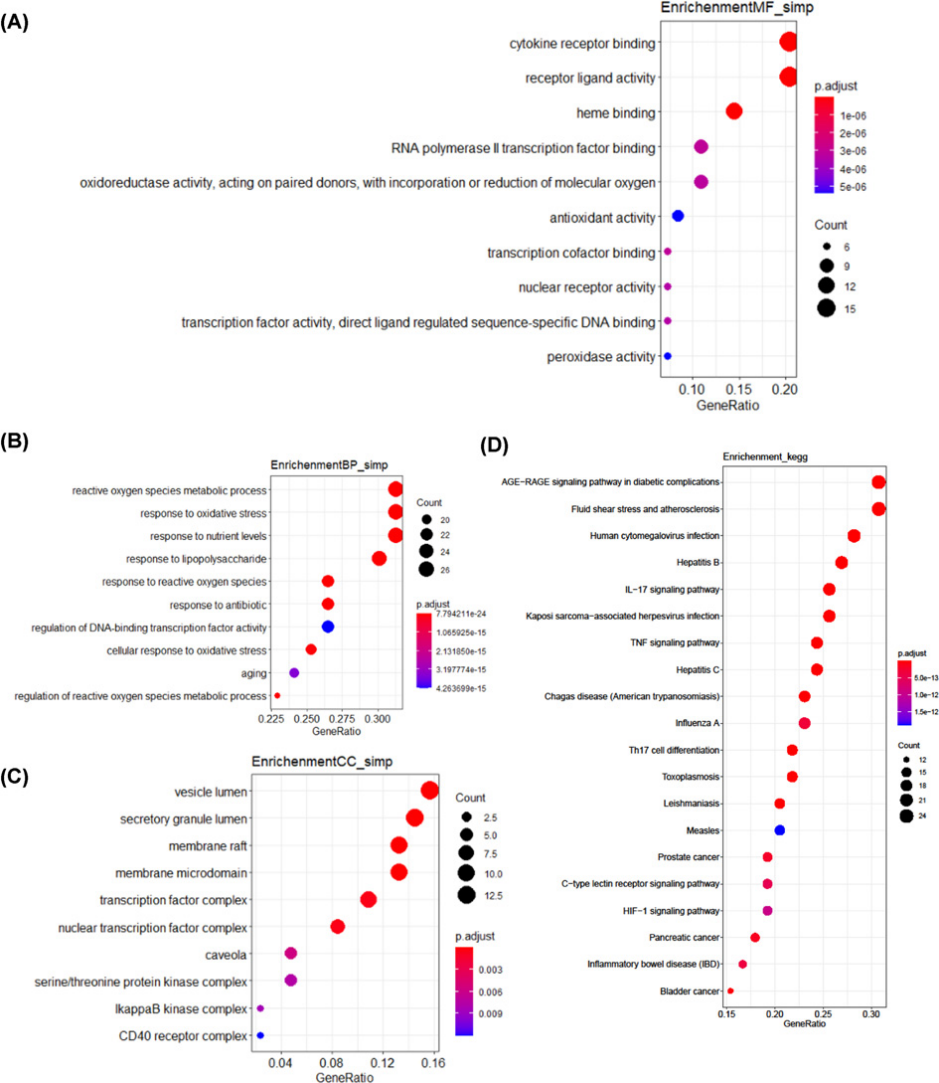
GO and KEGG pathway enrichment analysis results (**A**) MF enrichment analysis; (**B**) BP enrichment analysis; (**C**) CC enrichment analysis; (**D**) KEGG enrichment analysis. The size of each node indicates enriched counts, the abscissa represents the enriched gene ratio, and color means enriched adjusted *P*-value.

To further validate whether biological functions enriched by candidate targets as mentioned above were correlated with UC, GO and KEGG enrichment analyses were performed via R. Three GO terms were analyzed, which included CC, MF, and BP. The top 10 GO enrichment results for each term are shown in Figure 4. In the CC group, the GO terms mainly included the ‘vesicle lumen’, ‘secretory granule lumen’, and ‘membrane raft’. In the MF group, the GO terms were mainly involved ‘cytokine receptor binding’, ‘heme binding’, and ‘receptor ligand activity’. Moreover, BP results indicated that GGQLD may regulate UC-related biological processes, such as ‘reactive oxygen species metabolic process’, ‘response to lipopolysaccharide’, ‘response to reactive oxygen species’, and ‘response to oxidative stress’.

Additionally, 123 KEGG pathways were found. KEGG enrichment analysis showed that the 83 potential targets were closely related to several critical pathways associated with UC, such as ‘IL-17 signaling pathway’, ‘tumor necrosis factor (TNF) signaling pathway’, ‘Th17 cell differentiation’, ‘inflammatory bowel disease’, ‘Hypoxia-inducible factor (HIF)-1 signaling pathway’, and ‘inflammatory bowel disease’. The top 20 enriched KEGG signaling pathways are shown in Figure 4. These findings demonstrated that the regulation of inflammation and immunity may be the main mechanism of GGQLD in the treatment of UC. The detailed GO and KEGG pathway enrichment analyses results are listed in Supplementary Table S4.

### PPI network visualization

**Figure 5:**
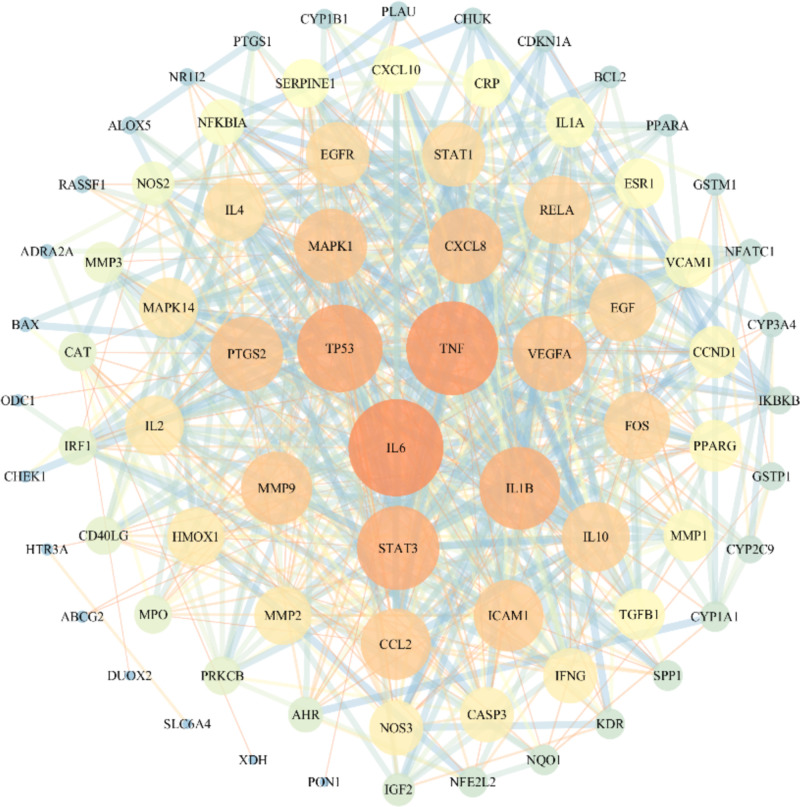
The PPI network of the potential targets Node size from large to small represents the descending order of the degree value. The line width between two nodes represents the interaction; the darker the color is, the more significant is the target.

To further investigate the relationship between the 83 overlapping genes, the PPIs were analyzed using the STRING online database, and high confidence target protein interaction data with a score >0.7 were selected. The obtained PPI network file was imported into Cytoscape 3.7.2 software for visualization. We found 77 nodes and 609 interactions in the PPI network, and the results are shown in Figure 5. ‘NetworkAnalyzer’ was used to show the importance of nodes.

### Hub gene investigation and screening of key active ingredients

**Figure 6:**
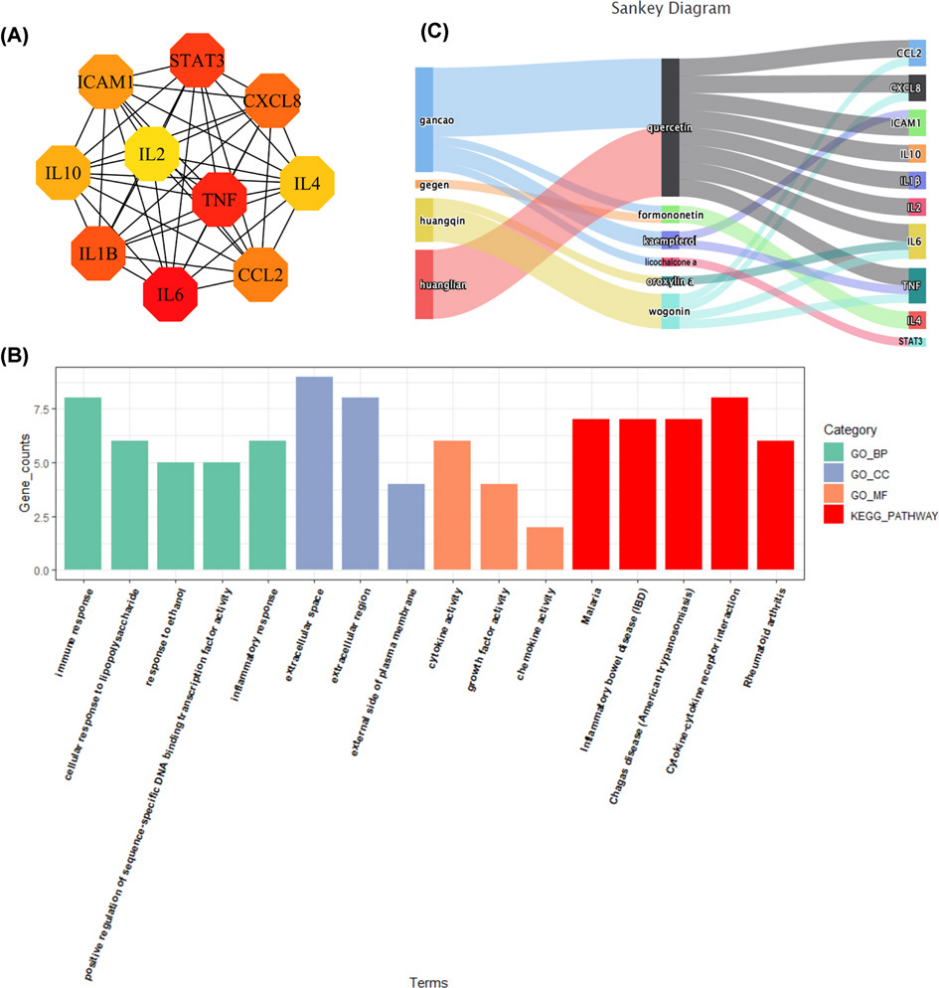
The results of hub gene investigation and screening of key active ingredients (**A**) The 10 hub genes identified from the PPI network. The darker the color is, the more significant the gene. (**B**) The top 5 GO terms and KEGG pathways of the 10 hub genes. Each bar indicates a BP/CC/MF/KEGG term, color represents an enriched type, abscissa means the number of enriched genes. (**C**) The Sankey diagram reveals the relationship between herbs, ingredients, and targets. The left blocks represent the herb, the middle blocks represent the ingredients, and the right blocks represent the hub targets.

According to the PPI network, the top 10 core nodes were identified, ranked by MCC in the ‘cytoHubba’ plugin. Thus, 10 genes were identified as hub genes: IL-6, TNF, STAT3, IL-1β, CXCL8, CCL2, intracellular adhesion molecule-1 (ICAM1), IL-10, IL-4, and IL-2, as shown in Figure 6A. Additionally, GO and KEGG analyses were conducted again on the 10 hub genes to validate their biological functions. The enrichment analysis results validated that the 10 hub genes were strongly related to UC. For instance, IL-6 is involved in ‘immune response’, ‘cellular response to lipopolysaccharide’, and ‘inflammatory bowel disease’, all of which are closely related to UC. The top 5 GO terms and KEGG pathways of the 10 hub genes are shown in Figure 6B. The detailed GO and KEGG enrichment of the 10 hub genes are listed in Supplementary Table S5. Moreover, a Sankey diagram was used to show the corresponding chemical compound that acts on hub genes, as shown in Figure 6C. Six ingredients in GGQLD, namely, quercetin, formononetin, kaempferol, licochalcone A, oroxylin A, and wogonin, which corresponded to the hub genes were identified. From the Sankey diagram, we can see that quercetin, shared by Huanglian and Gancao, targets most of the hub genes.

### Verification with molecular docking

**Figure 7:**
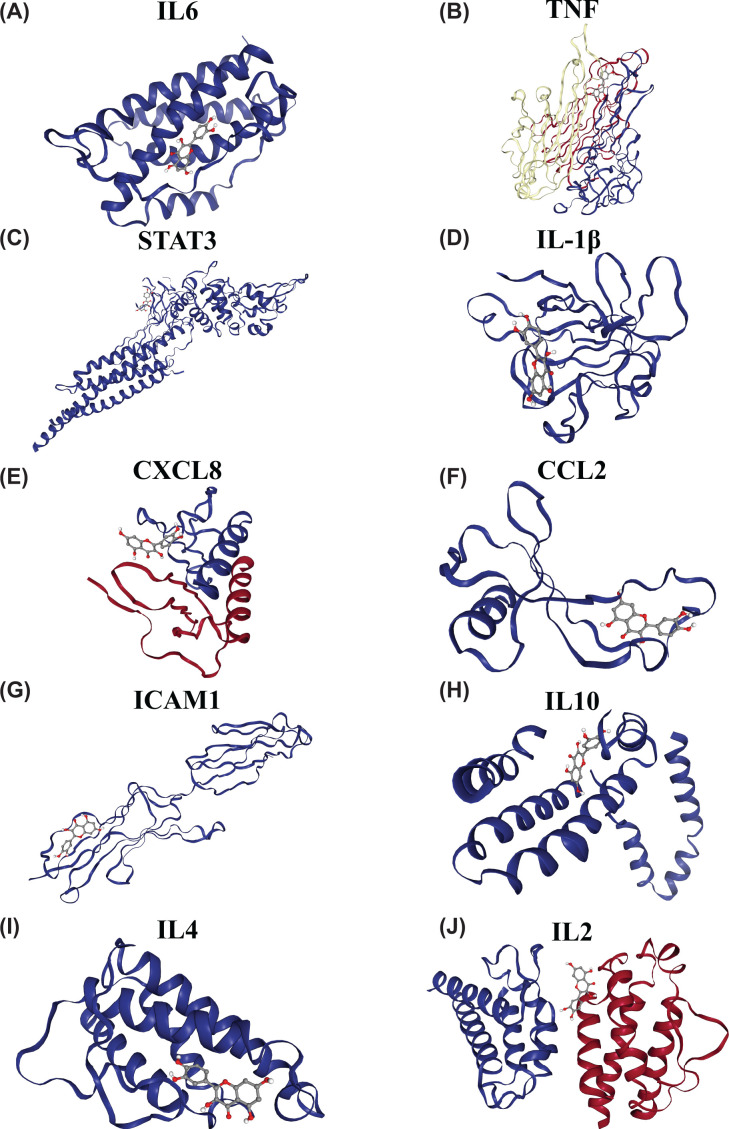
Molecular docking models of quercetin binding to the 10 hub targets Quercetin binds to IL-6 (**A**), TNF (**B**), STAT3 (**C**), IL-1β (**D**), CXCL8 (**E**), CCL2 (**F**), ICAM1 (**G**), IL-10 (**H**), IL-4 (**I**), and IL-2 (**J**). Ball and stick represents quercetin; cartoon represents a hub target.

To further validate potential targets in UC, we performed molecular docking on quercetin with the 10 hub genes (IL-6, TNF, STAT3, IL-1β, CXCL8, CCL2, ICAM1, IL-10, IL-4, and IL-2). Docking analysis successfully predicted Vina scores, which were all negative and less than −6, between quercetin and the 10 hub genes; the results are listed in Table 2. In particular, molecular docking between quercetin and TNF has the highest cavity size and the lowest Vina score. Overall, molecular docking results indicated that quercetin had good binding activities to the 10 hub genes, as shown in Figure 7.

**Table 2:** The results of cavity-detection guided blind docking

Ligand	Protein	Vina score	Cavity size	Center			Size		
				*x*	*y*	*z*	*x*	*y*	*z*
Quercetin	IL-6	−7.2	551	4	−2	10	27	21	21
	TNF	−9.1	863	8	63	31	21	21	21
	STAT3	−8	527	16	12	18	21	21	21
	IL-1	−6.7	197	40	9	56	21	21	21
	CXCL8	−6.3	185	−15	22	24	21	21	21
	CCL2	−6.3	48	31	30	16	21	21	21
	ICAM1	−6.6	100	50	92	−12	21	21	21
	IL10	−6.6	485	11	26	17	21	21	21
	IL4	−7.3	105	82	35	11	21	21	21
	IL2	−7.7	615	32	49	35	21	21	21

## Discussion

Currently, TCM is being more frequently chosen by patients to treat UC, and many TCM formulas have been successfully utilized for the treatment of UC [36,37], including GGQLD. *In vivo* and *in vitro* studies have provided multiple lines of evidence indicating that some ingredients of GGQLD, such as berberine and glabridin, have definite beneficial effects against UC [16]. Given the particularity of multicomponent, multitarget, and multichannel TCM formulas, the underlying molecular mechanisms triggered by GGQLD in the treatment of UC are far from being clarified. New approaches are now being considered for drug–target exploration and identification of potential active ingredients in TCM research, including network pharmacology [38]. Here, we used network pharmacology technology to predict the putative mechanism involved within the therapeutic effect of GGQLD in UC. In the present study, we identified 139 bioactive compounds and 259 targets from the 4 herbs in GGQLD. Furthermore, 83 potential therapeutic targets and 118 corresponding active ingredients were identified, and a G-U network was established with 205 nodes and 866 interactions. Additionally, GO and KEGG enrichment was further enriched based on the 83 potential target genes, and a PPI network was constructed. Finally, 5 potential ingredients and 10 hub genes were identified.

Consistent with previous studies, BP enrichment results indicated that a large number of genes were associated with oxidative stress, a key etiological factor of UC, including ‘reactive oxygen species metabolic process’, ‘response to reactive oxygen species’, ‘response to oxidative stress’, ‘regulation of reactive oxygen species metabolic process’, and ‘cellular response to oxidative stress’. The gastrointestinal tract is prone to reactive oxygen species (ROS) attack. Alterations in the balance between ROS production and the capacity to rapidly detoxify reactive intermediates lead to oxidative stress, which is an essential factor in the pathogenesis of gastrointestinal mucosal disease [39]. During inflammatory episodes, neutrophils and macrophages infiltrate the intestinal mucosa at the sites of IBD and release large amounts of ROS and cytokines, including interleukin (IL)-1β, IL-6, and TNF-α. Excessive levels of ROS released by the inflamed stroma elicit oxidative damage to DNA [40], proteins [41], and lipids, ultimately promoting the initiation and progression of UC. The response to lipopolysaccharide (LPS) was also enriched in BP, which could also play a pathogenic role in UC [42]. LPS is a major component of the outer membrane of gram-negative bacteria [43], and plays a key role in host–pathogen interactions with the innate immune system [44] and the development of inflammatory diseases [45]. LPS impairs intestinal barrier function by inhibiting intestinal restitution and stimulating the release of proinflammatory cytokines. Therefore, our results indicate that GGQLD may also have a role in regulating the intestinal microbiota.

Additionally, the KEGG results indicate that most of the disease-related pathways are immune and inflammatory, tumor-related signaling pathways, and virus infection-related signaling pathways, most of which have been reported to be closely related to UC. For example, IL-17, a key mediator in the pathogenesis of intestinal inflammation, is upregulated in inflamed mucosa from UC patients. The disease severity in UC patients is also correlated with the IL-17 level in peripheral blood mononuclear cells [46]. In addition, IL-17 is produced mainly by T helper 17 (Th17) cells and other sources, including natural killer cells, mast cells, and neutrophils. Th17 cells are considered to be key effector T cells of UC pathophysiology. New data from mouse models of IBD suggest that T cell plasticity, particularly along the Th1-Th17 and Th17-Treg axes, play an important role in the regulation of intestinal immune responses [47]. Our functional enrichment analysis results indicated that anti-inflammation, antioxidation, and immunomodulatory effects may be the mechanism of GGQLD against UC.

Therefore, we further constructed a PPI network to unravel the complex molecular relationships underlying the potential targets, and 10 hub genes, namely, IL-6, TNF, STAT3, IL-1β, CXCL8, CCL2, ICAM1, IL-10, IL-4, and IL-2, were identified by ‘cytoHubba’. Most of them are cytokines, such as proinflammatory cytokines (IL-6, TNF IL-1β, CXCL8, and CCL2), and anti-inflammatory cytokines (IL-10, IL-4, and IL-2). Cytokines are structurally diverse proteins with profound functional relevance to the maintenance of physiological homeostasis, including chemokines, interferons, interleukins, lymphokines, and tumor necrosis factors [48]. Cytokines are involved in intestinal homeostasis and pathological processes associated with IBD [49]. For example, M1 macrophages promote colonic inflammation via the production of the pro-inflammatory cytokines IL-6, CCL2, and TNF-α [50]. IL-6, a key proinflammatory cytokine in the pathogenesis of multiple inflammatory diseases, acts on mesenchymal and epithelial cells to induce the recruitment of polymorphonuclear leukocytes (PMNs) and macrophages essential for wound healing. Recently, a genome-wide meta-analysis of 20,550 patients with CD, 17,647 patients with UC, and more than 40,000 individuals without IBD (controls) indicated that therapeutics designed to block IL-6R signaling might be effective in the treatment of IBD [51]. Chemokines are small secreted proteins that orchestrate the migration and positioning of immune cells within tissues [52]. During innate and adaptive responses, chemokines are essential for the function of the immune system. CXCL8 is one of the first and most intensively studied chemokines acting as a proinflammatory chemokine [53]. Patients with UC have elevated levels of CXCL8 and CCL2 in the colonic mucosa compared with healthy volunteers [54]. TNF is another key cytokine in IBD pathology and induces intestinal epithelial cell apoptosis in the context of IBD and murine disease models [55]. Furthermore, treatment strategies targeting TNF signaling, such as infliximab, adalimumab, and golimumab, are administered systemically and efficaciously in UC.

Cytokines are crucial for the maintenance of the immune system. For example, IL-10 is a key immunosuppressive cytokine expressed by many cell types and is particularly important in maintaining intestinal microbe-immune homeostasis. Polymorphisms in the IL-10 locus confer risk for UC, deficient in either IL-10 or IL-10 receptor exhibit severe intestinal inflammation and marked pro-inflammatory cytokine secretion in mice and humans [56]. IL-10 production by Th17 cells has been strongly related to the acquisition of regulatory properties by Th17 cells and the resolution of intestinal inflammation [57]. IL-4, the core signature of Th2 responses, induces the differentiation of naive helper T cells to Th2 cells [58]. Enormous reports have also provided evidence that IL-4 participates in the pathogenesis of IBD [59,60]. Human IL-4-treated regulatory macrophages promote epithelial wound repair, reduce cytokine-induced epithelial barrier defects and are beneficial in a murine model of acute colitis [61]. IL-2 is known as a T cell growth factor [62], negatively regulates immune-mediated inflammation and stimulates tissue repair processes [63]. Low-dose IL-2 has therapeutic effects on DSS-induced colitis and potential clinical value in treating UC [64].

Cytokine signaling pathways involving transcription factors of the signal transducers and activators of transcription (STAT) family play a key role in the pathogenesis of IBD [48]. STAT3 activation occurs as a result of cytokine binding (e.g., IL-­6), and several studies have reported an increased expression of STAT3 or STAT3 phosphorylation in human IBD [65,66]. STAT3 has also been shown to be critical in modulating the balance of Th17 and regulatory T (Treg) cells as well as in promoting CD4(+) T cell proliferation [67]. ICAM-1 is a transmembrane glycoprotein of the immunoglobulin family that is constitutively expressed on vascular endothelial cells and upregulated in inflamed colonic tissue [68]. It has been demonstrated that ICAM-1 expression is increased in colonic lysates from UC patients. ICAM-1 can also be up-regulated in response to proinflammatory stimuli, such as TNF-α, IL-1β, and IFN-γ. In particular, ICAM-1 signaling seems to produce the recruitment of inflammatory immune cells [69] such as macrophages [70] and granulocytes [71]. Meanwhile, the GO and KEGG analyses of the 10 hub nodes showed that a considerable number of genes were involved in the ‘inflammatory bowel disease’ and ‘cytokine–cytokine receptor interaction’ pathways.

The Sankey diagram indicated that quercetin corresponds to most of the hub targets. Quercetin is a plant-derived polyphenolic compound belonging to the flavonols (a subclass of flavonoids) and has shown beneficial effects in the prevention and/or treatment of several pathological conditions due to its antioxidant, anti-inflammatory, antifibrotic, antimicrobial, and antitumoral activities [72–74]. In the gastrointestinal tract, quercitrin can reduce early-stage inflammation by reducing IL-1β, IL-6, and TNF-α levels and NF-κB expression [74]. Various studies have shown the anti-inflammatory activity of quercetin in experimental colitis [75,76]. Moreover, modulation of the intestinal microbiota is another way for quercetin to exert a therapeutic effect. Recent studies have shown that quercetin reshapes intestinal microbiota in several diseases, such as obesity, nonalcoholic fatty liver disease, and atherosclerosis, suggesting a prebiotic effect of flavonol [77,78]. Furthermore, molecular docking was conducted between quercetin and the 10 hub genes to verify the binding activities between active ingredients and potential targets. The results showed that quercetin had good binding activities to IL-6, TNF, STAT3, IL-1β, CXCL8, CCL2, ICAM1, IL-10, IL-4, and IL-2.

Cytokines that either promote or suppress intestinal inflammation have led to some efficacious therapeutics for IBD [79]. In general, these therapies can be grouped into two categories: blockade of pro-inflammatory cytokine pathways or enhancement of anti-inflammatory cytokine pathways [80]. The discovery and characterization of these therapies indicate that simultaneous regulation of anti-inflammatory factors and pro-inflammatory factors may become a more potential treatment strategy for IBD. Our research suggests that GGQLD may have such a therapeutic effect.

## Conclusions

Current research based on network pharmacology could provide a novel and systematic analysis method for the research of Chinese herbal formulas. However, it should be noted that there were some limitations in our article. On the one hand, focusing on validated target genes may exclude potential targets that have not been experimentally validated. On the other hand, there is a lack of verification analyses in our research. Thus, further clinical and basic research is needed to validate our results and to elucidate the molecular mechanism in GGQLD.

Overall, the present study suggests that quercetin, kaempferol, wogonin, baicalein, and naringenin may be the main active ingredients in GGQLD. Cytokines (e.g., IL-6, TNF, IL-1β, CXCL8, CCL2, IL-10, IL-4, and IL-2) might be potential therapeutic targets of GGQLD in UC. Furthermore, the effect of GGQLD on UC might be achieved by regulating the balance of cytokines in the immune system and inflammation-related pathways, such as the IL-17 pathway and Th17 cell differentiation pathway. Last but not least, this research showed that network pharmacology is a powerful tool for identifying active compounds and potential targets derived from TCM.

## Supplementary Material

Supplementary Tables S1-S5

## Data Availability

The data used to support the result of this study can be obtained from the corresponding author.

## Abbreviations

ADME: absorption, distribution, metabolism, and excretion
BP: biological process
CC: cellular component
DL: drug-likeness
FDA: Food and Drug Administration
G-U: GGQLD-compound-target-UC
GGQLD: Ge-Gen-Qin-Lian Decoction
GO: Gene Ontology
ICAM-1: intracellular adhesion molecule-1
IL: interleukin
KEGG: Kyoto Encyclopedia of Genes and Genomes
LPS: lipopolysaccharide
MF: molecular function
OB: oral bioavailability
PMN: polymorphonuclear leukocyte
PPI: protein–protein interaction
ROS: reactive oxygen species
STAT: signal transducers and activators of transcription
TCMSP: Traditional Chinese Medicine Systems Pharmacology Database and Analysis Platform
Th17: T helper 17
TNF: tumor necrosis factor
Treg: regulatory T
UC: ulcerative colitis
