# Supplementary material for: Uncovering the mechanism of Ge-Gen-Qin-Lian decoction for treating ulcerative colitis based on network pharmacology and molecular docking verification
Source: Biosci Rep. 2021 Feb 10;41(2):BSR20203565. doi: 10.1042/BSR20203565 (PMC7876598; doi:10.1042/BSR20203565)
Supplement: Supplementary Tables S1-S5 [file BSR-2020-3565_supp.pdf]

Table S1: Bioactive Compounds in GGQLD.

zhongyao	Mol ID	Molecule Name	MW	AlogP	Hdon	Hacc	OB (%)	Caco-2	BBB	DL	FASA-	HL
huanglian	MOL002907	Corchoroside A_qt	404.55	1.34	3	6	104.95	-0.91	-1.31	0.78	0.29	6.68
huanglian	MOL008647	Moupinamide	313.38	2.86	3	5	86.71	0.55	-0.51	0.26	0.33	3.71
huanglian	MOL000785	palmatine	352.44	3.65	0	4	64.6	1.33	0.37	0.65	0.13	2.25
huanglian	MOL000622	Magnograndiolide	266.37	1.18	2	4	63.71	0.02	-0.24	0.19	0.3	3.17
huanglian	MOL002903	(R)-Canadine	339.42	3.4	0	5	55.37	1.04	0.57	0.77	0.2	6.41
huanglian	MOL000098	quercetin	302.25	1.5	5	7	46.43	0.05	-0.77	0.28	0.38	14.4
huanglian	MOL002668	Worenine	334.37	3.73	0	4	45.83	1.22	0.24	0.87	0.27	8.41
huanglian	MOL013352	Obacunone	454.56	2.68	0	7	43.29	0.01	-0.43	0.77	0.31	-13.04
huanglian	MOL002897	epiberberine	336.39	3.45	0	4	43.09	1.17	0.4	0.78	0.19	6.1
huanglian	MOL001454	berberine	336.39	3.45	0	4	36.86	1.24	0.57	0.78	0.19	6.57
huanglian	MOL002904	Berlambine	351.38	2.49	0	6	36.68	0.97	0.17	0.82	0.28	7.33
huanglian	MOL002894	berberrubine	322.36	3.2	1	4	35.74	1.07	0.17	0.73	0.24	6.46
huanglian	MOL000762	Palmidin A	510.52	4.52	6	8	35.36	-0.38	-1.47	0.65	0.39	33.17
huanglian	MOL001458	coptisine	320.34	3.25	0	4	30.67	1.21	0.32	0.86	0.26	9.33
huangqin	MOL001689	acacetin	284.28	2.59	2	5	34.97	0.67	-0.05	0.24	0.35	17.25
huangqin	MOL000173	wogonin	284.28	2.59	2	5	30.68	0.79	0.04	0.23	0.32	17.75
huangqin	MOL000228	(2R)-7-hydroxy-5-methoxy-2-phenylchroman-4-one	270.3	2.82	1	4	55.23	0.87	0.26	0.2	0.34	17.02
huangqin	MOL002714	baicalein	270.25	2.33	3	5	33.52	0.63	-0.05	0.21	0.36	16.25
huangqin	MOL002908	5,8,2'-Trihydroxy-7-methoxyflavone	300.28	2.32	3	6	37.01	0.76	-0.07	0.27	0.28	16.17
huangqin	MOL002909	5,7,2,5-tetrahydroxy-8,6-dimethoxyflavone	376.34	2.02	4	9	33.82	0.35	-0.59	0.45	0.19	15.94
huangqin	MOL002910	Carthamidin	288.27	2.03	4	6	41.15	0.16	-0.42	0.24	0.36	15.81
huangqin	MOL002911	2,6,2',4'-tetrahydroxy-6'-methoxychaleone	302.3	2.62	4	6	69.04	-0.07	-0.32	0.22	0.34	21.89
huangqin	MOL002913	Dihydrobaicalin_qt	272.27	2.3	3	5	40.04	0.56	0.18	0.21	0.36	16.13
huangqin	MOL002914	Eriodyctiol (flavanone)	288.27	2.03	4	6	41.35	0.05	-0.66	0.24	0.39	15.88
huangqin	MOL002915	Salvigenin	328.34	2.82	1	6	49.07	0.86	-0.03	0.33	0.21	15.87

huangqin	MOL002917	5,2',6'-Trihydroxy-7,8-dimethoxyflavone	330.31	2.3	3	7	45.05	0.48	-0.11	0.33	0.25	16.37
huangqin	MOL002925	5,7,2',6'-Tetrahydroxyflavone	286.25	2.07	4	6	37.01	0.18	-0.56	0.24	0.38	18
huangqin	MOL002926	dihydrooroxylin A	286.3	2.55	2	5	38.72	0.71	0.03	0.23	0.29	17.58
huangqin	MOL002927	Skullcapflavone II	374.37	2.54	2	8	69.51	0.68	-0.07	0.44	0.2	16.14
huangqin	MOL002928	oroxylin a	284.28	2.59	2	5	41.37	0.76	0.13	0.23	0.29	17.15
huangqin	MOL002932	Panicolin	314.31	2.57	2	6	76.26	0.84	0.31	0.29	0.24	16.78
huangqin	MOL002933	5,7,4'-Trihydroxy-8-methoxyflavone	300.28	2.32	3	6	36.56	0.46	-0.4	0.27	0.31	16.93
huangqin	MOL002934	NEOBAICALEIN	374.37	2.54	2	8	104.34	0.74	-0.19	0.44	0.18	16.5
huangqin	MOL002937	DIHYDROOROXYLIN	286.3	2.55	2	5	66.06	0.67	0.13	0.23	0.31	17.17
huangqin	MOL000358	beta-sitosterol	414.79	8.08	1	1	36.91	1.32	0.99	0.75	0.23	5.36
huangqin	MOL000359	sitosterol	414.79	8.08	1	1	36.91	1.32	0.87	0.75	0.22	5.37
huangqin	MOL000525	Norwogonin	270.25	2.33	3	5	39.4	0.6	-0.17	0.21	0.39	16.93
huangqin	MOL000552	5,2'-Dihydroxy-6,7,8-trimethoxyflavone	344.34	2.55	2	7	31.71	0.93	0	0.35	0.22	16.47
huangqin	MOL000073	ent-Epicatechin	290.29	1.92	5	6	48.96	0.02	-0.64	0.24	0.34	0.63
huangqin	MOL000449	Stigmasterol	412.77	7.64	1	1	43.83	1.44	1	0.76	0.22	5.57
huangqin	MOL001458	coptisine	320.34	3.25	0	4	30.67	1.21	0.32	0.86	0.26	9.33
huangqin	MOL001490	bis[(2S)-2-ethylhexyl] benzene-1,2-dicarboxylate	390.62	7.57	0	4	43.59	0.98	0.68	0.35	0.28	3.02
huangqin	MOL001506	Supraene	410.8	11.33	0	0	33.55	2.08	1.73	0.42	0.27	2.72
huangqin	MOL002879	Diop	390.62	7.44	0	4	43.59	0.79	0.26	0.39	0.28	3.6
huangqin	MOL002897	epiberberine	336.39	3.45	0	4	43.09	1.17	0.4	0.78	0.19	6.1
huangqin	MOL008206	Moslosooflavone	298.31	2.84	1	5	44.09	1.01	0.54	0.25	0.26	17.02
huangqin	MOL010415	11,13-Eicosadienoic acid, methyl ester	322.59	7.55	0	2	39.28	1.46	1.24	0.23	0.21	5.44
huangqin	MOL012245	5,7,4'-trihydroxy-6-methoxyflavanone	302.3	2.28	3	6	36.63	0.43	-0.32	0.27	0.28	16.12

huangqin	MOL012246	5,7,4'-trihydroxy-8-methoxyflavanone	302.3	2.28	3	6	74.24	0.37	-0.43	0.26	0.31	16.85
huangqin	MOL012266	rivularin	344.34	2.55	2	7	37.94	0.65	-0.13	0.37	0.21	16.25
gegen	MOL000441	LUPENONE	424.78	7.36	0	1	11.66	1.48	1.31	0.78	0	
gegen	MOL000358	beta-sitosterol	414.79	8.08	1	1	36.91	1.32	0.99	0.75	0.23	5.36
gegen	MOL000663	lignoceric acid	368.72	10.02	1	2	14.9	1.24	1.01	0.33	0.17	
gegen	MOL000399	Docosanoate	340.66	9.11	1	2	15.69	1.21	0.91	0.26	0	
gegen	MOL011797	(3S,4aR,6aR,6bS,8aR,9R,12aS,14aR,14bR)-4,4,6a,6b,8a,11,11,14b-octamethyl-1,2,3,4a,5,6,7,8,9,10,12,12a,14,14a-tetradecahydropicene-3,9-diol	442.8	6.2	2	2	17.42	0.95	0.38	0.76	0.22	
gegen	MOL001999	scoparone	206.21	1.87	0	4	74.75	0.85	0.46	0.09	0.23	0.73
gegen	MOL000392	formononetin	268.28	2.58	1	4	69.67	0.78	0.02	0.21	0	17.04
gegen	MOL000390	daidzein	254.25	2.33	2	4	19.44	0.59	-0.22	0.19	0	
gegen	MOL002959	3'-Methoxydaidzein	284.28	2.32	2	5	48.57	0.56	-0.32	0.24	0.34	17.04
gegen	MOL004631	7,8,4'-Trihydroxyisoflavone	270.25	2.07	3	5	20.67	0.45	-0.37	0.22	0.41	
gegen	MOL000481	genistein	270.25	2.07	3	5	17.93	0.43	-0.4	0.21	0	
gegen	MOL003641	Soyasapogenol B	458.8	5.11	3	3	16.73	0.43	-0.34	0.75	0.21	
gegen	MOL000357	Sitogluside	576.95	6.34	4	6	20.63	-0.14	-0.93	0.62	0.23	
gegen	MOL000391	Ononin	430.44	0.68	4	9	11.52	-0.74	-1.67	0.78	0	
gegen	MOL002347	(R)-Allantoin	158.14	-1.76	5	7	96.9	-0.99	-1.37	0.03	0.39	11.33
gegen	MOL009720	daidzin	416.41	0.43	5	9	14.32	-1	-1.94	0.73	0.32	
gegen	MOL012297	puerarin	416.41	-0.06	6	9	24.03	-1.15	-2.06	0.69	0.37	
gegen	MOL003629	Daidzein-4,7-diglucoside	578.57	-1.48	8	14	47.27	-2.53	-4.06	0.67	0.31	16.18
gancao	MOL001484	Inermine	284.28	2.44	1	5	75.18	0.89	0.4	0.54	0.3	11.72
gancao	MOL001792	DFV	256.27	2.57	2	4	32.76	0.51	-0.29	0.18	0.42	17.89
gancao	MOL000211	Mairin	456.78	6.52	2	3	55.38	0.73	0.22	0.78	0.26	8.87

gancao	MOL002311	Glycyrol	366.39	4.85	2	6	90.78	0.71	-0.2	0.67	0.28	9.85
gancao	MOL000239	Jaranol	314.31	2.09	2	6	50.83	0.61	-0.22	0.29	0.29	15.5
gancao	MOL002565	Medicarpin	270.3	2.66	1	4	49.22	1	0.53	0.34	0.31	8.46
gancao	MOL000354	isorhamnetin	316.28	1.76	4	7	49.6	0.31	-0.54	0.31	0.32	14.34
gancao	MOL000359	sitosterol	414.79	8.08	1	1	36.91	1.32	0.87	0.75	0.22	5.37
gancao	MOL003656	Lupiwighteone	338.38	3.92	3	5	51.64	0.68	-0.23	0.37	0.36	15.63
gancao	MOL003896	7-Methoxy-2-methyl isoflavone	266.31	3.36	0	3	42.56	1.16	0.56	0.2	0.33	16.89
gancao	MOL000392	formononetin	268.28	2.58	1	4	69.67	0.78	0.02	0.21	0	17.04
gancao	MOL000417	Calycosin	284.28	2.32	2	5	47.75	0.52	-0.43	0.24	0	17.1
gancao	MOL000422	kaempferol	286.25	1.77	4	6	41.88	0.26	-0.55	0.24	0	14.74
gancao	MOL004328	naringenin	272.27	2.3	3	5	59.29	0.28	-0.37	0.21	0.4	16.98
gancao	MOL004805	(2S)-2-[4-hydroxy-3-(3-methylbut-2-enyl)phenyl]-8,8-dimethyl-2,3-dihydropyrano[2,3-f]chromen-4-one	390.51	5.48	1	4	31.79	1	0.25	0.72	0.35	14.82
gancao	MOL004806	euchrenone	406.56	6.35	1	4	30.29	1.09	0.39	0.57	0	15.89
gancao	MOL004808	glyasperin B	370.43	4.02	3	6	65.22	0.47	-0.09	0.44	0	16.1
gancao	MOL004810	glyasperin F	354.38	2.97	3	6	75.84	0.43	-0.15	0.54	0	15.64
gancao	MOL004811	Glyasperin C	356.45	4.73	3	5	45.56	0.71	0.07	0.4	0	3.13
gancao	MOL004814	Isotrifoliol	298.26	2.99	2	6	31.94	0.53	-0.25	0.42	0	7.91
gancao	MOL004815	(E)-1-(2,4-dihydroxyphenyl)-3-(2,2-dimethylchromen-6-yl)prop-2-en-1-one	322.38	3.96	2	4	39.62	0.66	-0.12	0.35	0	16.16
gancao	MOL004820	kanzonols W	336.36	3.63	2	5	50.48	0.63	0.04	0.52	0	0.15

gancao	MOL004824	(2S)-6-(2,4-dihydroxyphenyl)-2-(2-hydroxypropan-2-yl)-4-methoxy-2,3-dihydrofuro[3,2-g]chromen-7-one	384.41	2.96	3	7	60.25	0	-0.76	0.63	0	4.31
gancao	MOL004827	Semilicoisoflavone B	352.36	2.85	3	6	48.78	0.45	-0.33	0.55	0	17.02
gancao	MOL004828	Glepidotin A	338.38	3.9	3	5	44.72	0.79	0.06	0.35	0	16.09
gancao	MOL004829	Glepidotin B	340.4	3.88	3	5	64.46	0.46	-0.09	0.34	0	15.98
gancao	MOL004833	Phaseolinisoflavan	324.4	3.95	2	4	32.01	1.01	0.46	0.45	0	2.66
gancao	MOL004835	Glypallichalcone	284.33	3.4	1	4	61.6	0.76	0.23	0.19	0	17.01
gancao	MOL004838	8-(6-hydroxy-2-benzofuranyl)-2,2-dimethyl-5-chromenol	308.35	4.2	2	4	58.44	1	0.34	0.38	0.34	8.71
gancao	MOL004841	Licochalcone B	286.3	2.88	3	5	76.76	0.47	-0.46	0.19	0	17.02
gancao	MOL004848	licochalcone G	354.43	4.35	3	5	49.25	0.64	-0.04	0.32	0.35	15.75
gancao	MOL004849	3-(2,4-dihydroxyphenyl)-8-(1,1-dimethylprop-2-enyl)-7-hydroxy-5-methoxy-coumarin	368.41	4.03	3	6	59.62	0.4	-0.23	0.43	0	0.69
gancao	MOL004855	Licoricone	382.44	4.16	2	6	63.58	0.53	-0.14	0.47	0	16.37
gancao	MOL004856	Gancaonin A	352.41	4.17	2	5	51.08	0.8	0.13	0.4	0	16.82
gancao	MOL004857	Gancaonin B	368.41	3.91	3	6	48.79	0.58	-0.1	0.45	0	16.49
gancao	MOL004860	licorice glycoside E	693.71	1.59	7	14	32.89	-2.06	-2.8	0.27	0.31	25.39
gancao	MOL004863	3-(3,4-dihydroxyphenyl)-5,7-dihydroxy-8-(3-methylbut-2-enyl)chromone	354.38	3.65	4	6	66.37	0.52	-0.13	0.41	0	15.81
gancao	MOL004864	5,7-dihydroxy-3-(4-methoxyphenyl)-8-(3-methylbut-2-enyl)chromone	352.41	4.17	2	5	30.49	0.9	0.21	0.41	0	14.99

gancao	MOL004866	2-(3,4-dihydroxyphenyl)-5,7-dihydroxy-6-(3-methylbut-2-enyl)chromone	354.38	3.92	4	6	44.15	0.48	-0.28	0.41	0	16.77
gancao	MOL004879	Glycyrin	382.44	4.67	2	6	52.61	0.59	-0.13	0.47	0	1.31
gancao	MOL004882	Licocoumarone	340.4	4.98	3	5	33.21	0.84	0.06	0.36	0	9.66
gancao	MOL004883	Licoisoflavone	354.38	3.65	4	6	41.61	0.37	-0.27	0.42	0	16.09
gancao	MOL004884	Licoisoflavone B	352.36	2.85	3	6	38.93	0.46	-0.18	0.55	0	15.73
gancao	MOL004885	licoisoflavanone	354.38	2.97	3	6	52.47	0.39	-0.22	0.54	0	15.67
gancao	MOL004891	shinpterocarpin	322.38	3.46	1	4	80.3	1.1	0.68	0.73	0.32	6.5
gancao	MOL004898	(E)-3-[3,4-dihydroxy-5-(3-methylbut-2-enyl)phenyl]-1-(2,4-dihydroxyphenyl)prop-2-en-1-one	340.4	4.49	4	5	46.27	0.41	-0.4	0.31	0.43	15.24
gancao	MOL004903	liquiritin	418.43	0.66	5	9	65.69	-1.06	-1.93	0.74	0	17.96
gancao	MOL004904	licopyranocoumarin	384.41	3.04	3	7	80.36	0.13	-0.62	0.65	0	0.08
gancao	MOL004905	3,22-Dihydroxy-11-oxo-delta(12)-oleanene-27-alpha-methoxycarbonyl-29-oic acid	512.75	4.37	1	6	34.32	-0.06	-0.75	0.55	0	3.56
gancao	MOL004907	Glyzaglabrin	298.26	2.1	2	6	61.07	0.34	-0.2	0.35	0	21.2
gancao	MOL004908	Glabridin	324.4	3.95	2	4	53.25	0.97	0.36	0.47	0	0.03
gancao	MOL004910	Glabranin	324.4	4.42	2	4	52.9	0.97	0.31	0.31	0	16.24
gancao	MOL004911	Glabrene	322.38	3.77	2	4	46.27	0.99	0.04	0.44	0	3.63
gancao	MOL004912	Glabrone	336.36	3.12	2	5	52.51	0.59	-0.11	0.5	0	16.09
gancao	MOL004913	1,3-dihydroxy-9-methoxy-6-benzofurano[3,2-c]chromenone	298.26	2.99	2	6	48.14	0.48	-0.19	0.43	0	8.87

gancao	MOL004914	1,3-dihydroxy-8,9-dimethoxy-6-benzofurano[3,2-c]chromenone	328.29	2.98	2	7	62.9	0.4	-0.34	0.53	0	9.32
gancao	MOL004915	Eurycarpin A	338.38	3.92	3	5	43.28	0.43	-0.06	0.37	0	17.1
gancao	MOL004917	glycyroside	562.57	-0.73	6	13	37.25	-1.58	-2.56	0.79	0	14.62
gancao	MOL004924	(-)-Medicocarpin	432.46	0.75	4	9	40.99	-0.6	-1.34	0.95	0	13.2
gancao	MOL004935	Sigmoidin-B	356.4	3.89	4	6	34.88	0.42	-0.41	0.41	0	14.49
gancao	MOL004941	(2R)-7-hydroxy-2-(4-hydroxyphenyl)chroman-4-one	256.27	2.57	2	4	71.12	0.41	-0.25	0.18	0	18.09
gancao	MOL004945	(2S)-7-hydroxy-2-(4-hydroxyphenyl)-8-(3-methylbut-2-enyl)chroman-4-one	324.4	4.42	2	4	36.57	0.72	-0.04	0.32	0	17.95
gancao	MOL004948	Isoglycyrol	366.39	4.36	1	6	44.7	0.91	0.05	0.84	0	6.69
gancao	MOL004949	Isolicoflavonol	354.38	3.63	4	6	45.17	0.54	-0.42	0.42	0	15.55
gancao	MOL004957	HMO	268.28	2.58	1	4	38.37	0.79	0.25	0.21	0	16.56
gancao	MOL004959	1-Methoxyphaseollidin	354.43	4.25	2	5	69.98	1.01	0.48	0.64	0	9.53
gancao	MOL004961	Quercetin der.	330.31	1.82	3	7	46.45	0.39	-0.44	0.33	0	16.61
gancao	MOL004966	3'-Hydroxy-4'-O-Methylglabridin	354.43	3.93	2	5	43.71	1	0.73	0.57	0	-0.61
gancao	MOL000497	licochalcone a	338.43	4.62	2	4	40.79	0.82	-0.21	0.29	0	16.2
gancao	MOL004974	3'-Methoxyglabridin	354.43	3.93	2	5	46.16	0.94	0.47	0.57	0	0.52
gancao	MOL004978	2-[(3R)-8,8-dimethyl-3,4-dihydro-2H-pyrano[6,5-f]chromen-3-yl]-5-methoxyphenol	338.43	4.2	1	4	36.21	1.12	0.61	0.52	0	-0.13
gancao	MOL004980	Inflacoumarin A	322.38	4.7	2	4	39.71	0.73	-0.24	0.33	0	2.31
gancao	MOL004985	icos-5-enoic acid	310.58	7.75	1	2	30.7	1.22	1.09	0.2	0	5.28

gancao	MOL004988	Kanzonol F	420.54	5.3	1	5	32.47	1.18	0.56	0.89	0.28	9.98
gancao	MOL004989	6-prenylated eriodictyol	356.4	3.89	4	6	39.22	0.4	-0.29	0.41	0	16.52
gancao	MOL004990	7,2',4'-trihydroxy − 5-methoxy-3 − arylcoumarin	300.28	2.56	3	6	83.71	0.24	-0.59	0.27	0	0.99
gancao	MOL004991	7-Acetoxy-2-methylisoflavone	294.32	3.15	0	4	38.92	0.74	0.16	0.26	0	17.49
gancao	MOL004993	8-prenylated eriodictyol	356.4	3.89	4	6	53.79	0.43	-0.44	0.4	0	15.7
gancao	MOL004996	gadelaidic acid	310.58	7.75	1	2	30.7	1.2	0.94	0.2	0	5.25
gancao	MOL000500	Vestitol	272.32	3.15	2	4	74.66	0.86	0.3	0.21	0	3
gancao	MOL005000	Gancaonin G	352.41	4.17	2	5	60.44	0.78	0.23	0.39	0	16.13
gancao	MOL005001	Gancaonin H	420.49	4.71	3	6	50.1	0.6	-0.14	0.78	0	16.64
gancao	MOL005003	Licoagrocarpin	338.43	4.51	1	4	58.81	1.23	0.61	0.58	0.27	9.45
gancao	MOL005007	Glyasperins M	368.41	3.22	2	6	72.67	0.49	-0.04	0.59	0	15.57
gancao	MOL005008	Glycyrrhiza flavonol A	370.38	2.17	4	7	41.28	-0.09	-0.81	0.6	0	13.71
gancao	MOL005012	Licoagroisoflavone	336.36	3.48	2	5	57.28	0.71	0.09	0.49	0	19.64
gancao	MOL005013	18α-hydroxyglycyrrhetic acid	486.76	4.55	3	5	41.16	-0.29	-0.78	0.71	0	4.96
gancao	MOL005016	Odoratin	314.31	2.3	2	6	49.95	0.42	-0.24	0.3	0	16.35
gancao	MOL005017	Phaseol	336.36	4.87	2	5	78.77	0.76	-0.06	0.58	0	9.64
gancao	MOL005018	Xambioona	388.49	4.68	0	4	54.85	1.09	0.52	0.87	0	14.5
gancao	MOL005020	dehydroglyasperins C	340.4	4.3	4	5	53.82	0.68	-0.12	0.37	0	2.75
gancao	MOL000098	quercetin	302.25	1.5	5	7	46.43	0.05	-0.77	0.28	0.38	14.4

Table S2: Target information.

GGQLT	DisGenet	GeneCards	DrugBank
HTR2A	AMZ1	NOD2	NR3C1
HTR2C	DUPD1	IL6	CYP3A4
HTR3A	LINC00484	IL10	ABCB1
ADRA1A	RTF1	TLR4	CYP19A1
ADRA1B	MFSD13A	IL23R	CYP3A5
ADRA1D	DPH5	HLA-DRB1	CYP3A7
ADRA2C	KCCAT333	IL1RN	CYP2A6
ADRB2	IBD2	ABCB1	CYP1B1
KCNMA1	IBD3	TNF	CYP2B6
CALM	RNF186	CCR6	CYP2C8
PDE10A	AFTPH	IRF5	CYP2C9
F10	BEST2	ATG16L1	CYP2C19
DRD5	IBD22	IL10RA	ALB
OPRD1	IBD24	IRGM	SERPINA6
DRD1	LINC00994	IL10RB	ABCB11
HSP90AB1	MIR4728	MLH1	ANXA1
PRKACA	CALHM6	INAVA	SLCO1A2
CHRM1	PROSER1	PRTN3	SLC22A8
CHRM2	IGLL2P	CTNNB1	SCN10A
CHRM3	MAST3	CD40LG	PTGER1
CHRM4	NKX2-3	CXCL8	CYP2D6
CHRM5	DEFA6	IL1B	NOS2
OPRM1	GPR18	SMAD7	NR0B1
KCNH2	C5orf66	TLR2	ABCC2
PTGS1	LINC01475	AKT1	ABCG2
PTGS2	TMBIM1	MPO	CYP17A1
RXRA	GALM	TP53	CYP1A1
SCN5A	IBD6	SMAD4	CYP2E1
SLC6A3	MEP1A	MSH2	CYP3A43
SLC6A2	SLC22A23	IL2	CYP4A11
SLC6A4	KCNN1	STAT3	NR1I2
AR	P2RY14	MSH6	CYP11B1
ESR1	BAHD1	HLA-B	HSD11B2
NOS2	NXPE1	IFNG	HSD11B1
NOS3	SPATA48	CAT	SHBG
NCOA2	C17orf67	CRP	CYP11B2
PRSS1	LINC02213	CTLA4	AKR1D1
F7	FLJ31356	NLRP3	SRD5A2
NR3C2	OTUD3	MMP1	HSD3B1
GABRA1	GAL3ST2	FAS	CD40LG
GABRA2	FAM118A	IL4	TPMT
GABRA6	INTS11	ELANE	XDH
GRIA2	RIC8B	FOXP3	HPRT1
CDK2	UQCR10	TNFAIP3	ABCC4
ESR2	PKIG	TGFB1	ABCC5
PIM1	P2RY13	IL17A	SLC28A2
PSMD3	BRINP3	PTPN22	SLC28A3

MMP2	USP12	HLA-DQB1	SLC29A1
HSPA5	LDAH	TNFRSF1B	SLC29A2
ACHE	RPLP2	ICAM1	AOX1
ACACA	PGA5	PTGS2	PPAT
AHSA1	GPR12	GAST	IMPDH
AKR1B1	KIF21B	NGF	PTGS1
MAOB	HIPK1	JAK2	PTGS2
BAX	GBAP1	APC	PPARG
BCL2	LINC00598	MST1	CHUK
ALOX5	TRAF3IP2-AS1	HLA-A	IKBKB
AHR	IGHD3-9	MMP9	ALOX5
ABCG2	IGHD3-10	SLC11A1	MPO
BIRC5	POP5	MUC2	NAT2
BCL2L1	BRWD1	RELA	NAT1
CASP3	YDJC	MEFV	IFNG
CASP8	HSF2	FASLG	MC2R
CASP9	MUC12	PPARG	BCHE
CTSD	POP7	STAT4	
CAV1	TPD52L1	BTNL2	
CCL2	IL17REL	S100A8	
CD40LG	ARPC2	TNFSF15	
CDK1	ZFP90	TNFRSF1A	
TP53	CMC1	IL13	
CLDN4	DENND1B	VEGFA	
COL1A1	ZNF831	ALB	
COL3A1	BSN	IL22	
CRP	LINC01185	TLR5	
CXCL10	TEX41	REG4	
CXCL11	PPP2R3C	CARD9	
CXCL2	PTPRS	STAT1	
CDKN1A	CRNKL1	PSTPIP1	
CYP1A1	EIM	CCND1	
CYP1A2	RNF128	KRAS	
CYP1B1	HNF4G	CTSG	
CYP3A4	IRAK2	NOS2	
DCAF5	NR1D2	IL1R1	
DPP4	UBE4A	ITGB2	
TOP1	CCNY	FGF2	
TOP2A	LURAP1L	REL	
TOP2	ADAD1	IL23A	
DUOX2	ZPBP2	TPMT	
EGFR	GPR65	XDH	
SELE	IL21-AS1	S100A12	
SULT1E1	LINC01250	ERAP1	
ELK1	CELSR3	MYO9B	
EIF6	SFMBT1	EGF	
CCND1	IBD5	LACC1	
CCNB1	TSPAN14	CCR3	

GJA1	RGS14	PTGS1	
GSTM1	REG4	LTF	
GSTM2	CORIN	MIR21	
GSTP1	CMM	IL12B	
HSF1	NCAPD3	SST	
HSPB1	TOLLIP	BPI	
HMOX1	MUC13	HRAS	
HK2	LYRM4	CD4	
NKX3-1	PSD	GPR35	
HAS2	MR1	TCF4	
HIF1A	IL26	SMAD3	
CHUK	SLC9A8	DUOX2	
INSR	ADGRL2	CIITA	
IGF2	FNBP1	IL18	
IGFBP3	NDFIP1	FCGR2A	
ICAM1	SEMA6D	NLRP1	
IFNG	GP2	IL17F	
IRF1	CHST2	PTPRC	
IL1A	MUC3A	CLDN18	
IL1B	PANX1	CCN2	
IL10	APBA1	SPP1	
IL2	HPP1	S100A9	
IL6	PANX2	MUC12	
CXCL8	LACC1	IL12A	
MMP1	ARHGEF6	BRAF	
MGAM	ORMDL3	PIK3CA	
MMP9	ZNF300	C4A	
MAPK1	RTEL1-TNFRSF6B	IL2RA	
MYC	LURAP1L-AS1	CD28	
MPO	APEH	CLDN2	
NQO1	C5orf56	UCN	
POR	TOM1	ICOSLG	
NCF1	DUSP16	MIR145	
NFKBIA	DLG5	CCL2	
NFE2L2	CLDN2	CDH1	
NR1I2	AQP8	NFKB1	
NR1I3	CCNDBP1	TIMP1	
ODC1	DBNL	SLC22A4	
SPP1	CUZD1	ERBB2	
PPARG	CLDN18	CHGA	
PPARA	ATOH1	ATP4A	
PPARD	ASMT	ZFP90	
PTEN	ICAM2	HRH2	
PIK3CG	MGAT5	SLC22A5	
SERPINE1	LPAR6	RET	
PARP1	GAS7	F2	
PCOLCE	MKNK2	F5	
EGF	LSP1	ITGAM	

PTGER3	GPR35	MIR126	
ACPP	UBE2L3	PLA2G2A	
RUNX1T1	PUS10	TLR9	
PRKCA	CAMK2A	NOD1	
PRKCB	MYRF	SELE	
FOS	MSH5-SAPCD1	ODC1	
NPEPPS	ADCY3	HGF	
AKT1	PNKD	PLG	
RAF1	BANK1	MTHFR	
RASSF1	MAML2	HLA-DQA1	
RASA1	ATXN2L	CDKN1A	
ERBB2	NAAA	DEFB4A	
ERBB3	MIR196A2	FCGR3B	
RB1	ADORA3	IRF1	
RUNX2	ACAT2	TFF1	
CHEK2	ARHGEF28	TYK2	
PON1	HDAC11	TERT	
STAT1	LRG1	CD8A	
SLC2A4	HTR3A	MUC5AC	
MMP3	SLC23A1	APOH	
SOD1	STX2	WRN	
SERPIND1	ACSL1	PTEN	
THBD	LILRB4	DEFA5	
F3	VNN1	IL1A	
PLAT	FZD3	MMP2	
JUN	IRGM	ATP12A	
E2F1	ADCY7	MVK	
E2F2	DEFA5	SERPINC1	
RELA	UBAC2	PTPN2	
TGFB1	KSR1	MICA	
TNF	LINC00824	GNAS	
DIO1	PLCL1	VDR	
PLAU	ERAP2	ITGA4	
VCAM1	ANKRD55	PLCG2	
VEGFA	ZGPAT	H19	
XDH	HDAC7	CALCA	
CHEK1	SBNO2	ITGB4	
NCOA1	FGFR1OP	RASGRP1	
PDE3A	SKAP2	MMP3	
FASN	SLC5A8	VCAM1	
FASLG	MUC20	MIR143	
CYP19A1	HHIP	EGFR	
MAPK14	LGALS14	HP	
GSK3B	TIGAR	SERPINE1	
KDR	SLURP1	BAX	
BBC3	COMMD1	HLA-C	
TEP1	NPL	MIR34A	
PRKCD	ST2	CSF2	

MCL1	LYN	LTA	
PKIA	ITPA	CXCR2	
CHRNA7	SLC6A14	PLA2G4A	
FOSL1	TACR2	ACE	
FOSL2	WISP1	CASR	
CYCS	KAT2A	NCF4	
ALOX12	MADCAM1	GHRL	
NFATC1	GSDMB	IL5	
TDRD7	INS-IGF2	INS	
EGLN1	FNDC3A	DNMT1	
NOX5	KIAA1109	GRP	
FABP5	SETD1A	PTGER4	
APOD	SLC9A3	TFF3	
PTPN1	CLCN2	CCR5	
PYGM	MAGEC2	MIF	
CACNA2D1	SMOX	RIPK1	
CDK7	NPSR1	LAMC2	
CYP2C9	MIR629	CDKN2A	
PGR	CYP26B1	MAP3K7	
GABRA5	CBFA2T3	DLG5	
GABRA3	CA1	HLA-DPA1	
CHRNA2	REEP6	CCL5	
MAP2	HTR7	MIR150	
LACTBL1	SLC10A2	CDKN1B	
ADH1C	GPR55	IFNGR1	
ADRA2A	WNT11	HMOX1	
LTA4H	FUT3	MASP2	
MAOA	SLC44A4	PDGFB	
CTRB1	ZMIZ1	CD79A	
ADRB1	GCKR	CCL11	
CA2	THADA	IFNG-AS1	
CCNA2	UBASH3A	CARMIL2	
IL4	SP140	PRKCQ	
SIRT1	FIBP	CD40	
ATP5B	CCL25	MIR141	
MT-ND6	MLN	CXCL1	
HSD3B2	LOC390714	CLEC7A	
HSD3B1	DUOXA2	ZAP70	
MAPK10	ADRA2A	LYST	
OLR1	LOC105379528	IL33	
IKBKB	PARD3	RTEL1	
MAPK8	CAVIN3	FGF7	
PPP3CA	MPIG6B	NAT2	
AKR1C3	TNFRSF17	MMP7	
SLPI	RARRES2	CDKN2B	
MAPK3	PI3	TLR3	
LDLR	SLC15A1	LRBA	
BAD	MAD2L1	NFE2L2	

CAT	NFIL3	IGF1	
MTTP	IL10RB	CYP2C19	
APOB	PDZK1IP1	KRT7	
PLB1	EYA4	AXIN2	
HMGCR	FABP2	TAC1	
UGT1A8	TUSC3	ITGAL	
SREBF1	CARD9	IL15	
GSR	ZNF365	HIF1A	
ABCC1	SUOX	EPX	
ADIPOQ	JAZF1	IL7R	
SOAT2	IFNGR2	MLH3	
AKR1C1	IGF2-AS	CD80	
GOT1	OSMR	IL26	
ABAT	CCL24	MICB	
CES1	CLDN4	TAP2	
SOAT1	MXI1	POLE	
ITM2C	IL17RD	POLD1	
RXRB	DSG2	GALNT12	
STAT3	BW35	MT-CO1	
CDK4	CXCL16	CCL3	
	SLC9A1	SELP	
	SCNN1G	VIP	
	CXCL6	CSF3	
	IL15RA	TFF2	
	CD160	DEFB1	
	FCAR	RNASE3	
	TUFM	MUC6	
	TFF2	FOXD2-AS1	
	EED	TYMS	
	PTPN2	LEP	
	ECM1	CXCL5	
	LAMB1	IBD5	
	PSMG1	TTC7A	
	CDKAL1	HLA-DRA	
	NR5A2	IL6ST	
	MSH5	IL12RB2	
	PRKCQ	MET	
	CIT	IBD3	
	CD226	CASP10	
	SLC22A4	PYY	
	CCL26	IRAK1	
	NFKBIL1	SOCS3	
	TPMT	SOCS1	
	DMBT1	MIR200C	
	MIR193A	FGFR2	
	FERMT1	PDGFRL	
	F11R	IL21	
	NCKIPSD	MIR196B	

	CSF2RA	MADCAM1	
	LOC107984148	MIR221	
	AOC1	PDGFRB	
	DEFB104B	XIAP	
	ALDOB	HSPA1L	
	MEPE	FN1	
	SCYL1	PDCD1	
	DEFB104A	IBD2	
	IL34	CLDN4	
	HNMT	IFNA2	
	SCNN1B	SELL	
	LCT	H2AC18	
	SMAD5	DOCK8	
	MSI1	MIR25	
	MST1R	CD55	
	NELL1	SCT	
	STUB1	MLN	
	H1F0	B2M	
	GZMA	IL1RAPL2	
	VIPR1	HSPD1	
	RIPK2	RIPK2	
	IL18RAP	DPYD	
	CDH3	FCGR3A	
	TNFRSF14	CCK	
	ASAP2	MYC	
	ERAP1	MUTYH	
	NPEPPS	FOS	
	MYO9B	TACR1	
	IRAK3	ETS1	
	CBR1	ADIPOQ	
	SFRP2	MIR155	
	PER3	MIR140	
	MIR196B	TJP1	
	FAAH	CAV1	
	CD209	TGFA	
	APOA4	RFX5	
	MIR26B	COL7A1	
	PTGER4	AURKA	
	RNU1-4	COL17A1	
	SAFB	DCLRE1C	
	ITGAE	NKX2-3	
	LGALS4	CDX2	
	IRF4	FERMT1	
	LAIR1	TREM1	
	SLC11A2	PLAU	
	NFATC2	ALOX5	
	FHL2	HMGB1	
	TRAIP	FUT2	

	HIC1	CXCL10	
	TCN2	MIR222	
	FZD1	MUC1	
	DHX16	MUC3A	
	HERC2	LGALS3	
	ICOSLG	SRC	
	PRRC2A	CD14	
	CRB1	MIRLET7A1	
	SH2B3	MIR192	
	PROCR	MIR20A	
	PARK7	IL18RAP	
	LITAF	SKIV2L	
	SLC22A5	IBD8	
	BPI	JUN	
	ALPI	MAP2K2	
	SF3B6	HPS1	
	IGHV3-69-1	F3	
	IGHV3OR16-7	KLF6	
	MIR195	PECAM1	
	ARHGDIA	NLRP12	
	XRCC6P5	CXCR3	
	IGAN1	MBL2	
	PROK2	ENG	
	EGLN3	FGFR3	
	IGHA1	GZMB	
	S100A12	IBD21	
	LGALS9	ENO1	
	KLK1	C1R	
	DDX39A	IL37	
	CDK2AP2	IL27	
	CARD8	KRT20	
	USP14	IL3	
	NR1H2	IL6R	
	TERF2	MMP13	
	PLA2G7	CCR1	
	FZD4	IBD7	
	ATG16L1	JAK3	
	FUT2	MIR127	
	NFATC1	IL19	
	BACH2	CP	
	HSPA1L	MKI67	
	CD6	MIR27A	
	CLEC16A	SLC6A4	
	TET2	MAPK14	
	CREM	IFNA1	
	MAGI2	CXCL2	
	HSP90B1	NR1I2	
	CDX2	GSTP1	

	CCR9	TLR1	
	TRAF3IP2	BGLAP	
	LINC02210-CRHR1	NFKBIA	
	PADI4	HPGD	
	LAMTOR2	CCL20	
	RNU1-1	LCN2	
	NOX1	TAPBP	
	UGT1A7	IL11	
	MIR141	IBD6	
	MIR192	HNF4A	
	GPBAR1	CXCL12	
	OR10A4	C4B	
	ACTA1	MIR93	
	CDKN2B-AS1	MIR15B	
	CD33	CASP1	
	MAGT1	UBAC2	
	PRTN3	GCG	
	MAP2K2	SLC9A3	
	HPGD	CFB	
	KRT8	GSTM1	
	CEACAM6	TLR6	
	IL1RL1	RFXANK	
	SLC16A1	RFXAP	
	TBPL1	PSC	
	ELF3	F2RL1	
	PLK2	LGALS1	
	RPP14	TMSB4X	
	HLA-DQB2	AREG	
	FZD5	NR3C1	
	GNAO1	CEACAM6	
	TNFSF15	MGMT	
	SLC26A3	EPO	
	SPHK1	MIR483	
	IKZF1	NRAS	
	PRDM1	CXCL9	
	TNFRSF6B	HSPA4	
	TNFAIP3	IDH1	
	RPS6KB1	MIR106B	
	ANTXR2	POMC	
	PLCG2	NTRK1	
	CCHCR1	IL17RA	
	KEAP1	IBD4	
	NR0B1	IBD9	
	FGF7	IL2RB	
	HLA-DRB3	ARID1A	
	TREM1	ADAM17	
	NR4A1	BCL2	
	MYOD1	ORMDL3	

	MIR143	LIFR	
	IL24	ESR1	
	IL10RA	PMS2	
	BTNL2	GPT	
	SLC52A1	CASP3	
	CRYZ	TGM2	
	MIR126	CXCR4	
	ARRB2	IL1RL1	
	MIR200C	RORC	
	BIN1	SH2D1A	
	CHGA	CD44	
	PAX5	GREM1	
	PGD	IBD15	
	CCL8	IBD18	
	MAF	IBD11	
	TNFRSF9	IBD12	
	NTF3	IBD16	
	PAEP	IBD20	
	MUC4	IBD22	
	TLR6	IBD23	
	TXNIP	IBD24	
	E2F4	IBD26	
	TCF7L2	IBD27	
	GHSR	DPP4	
	CCL20	DMBT1	
	GHRL	NOTCH1	
	MASP2	BCL2L1	
	TCF4	STAT6	
	RORC	CYP2C9	
	ACAD8	THBD	
	NOD1	TNFRSF25	
	CCL11	MIR146A	
	CLEC7A	CHUK	
	CXCL5	SMAD2	
	BCL3	CYP3A4	
	REL	NTS	
	SELL	ALOX12	
	IL19	NR1H4	
	CLDN7	PTPN11	
	DEFB1	MIR214	
	AMACR	CYP2D6	
	DNM2	PROS1	
	RRM2B	BRCA2	
	B3GAT1	CFH	
	DCC	CCL7	
	CRHR2	SOD1	
	IL17B	MAPK8	
	CBLL2	MYD88	

	MIR214	HSPA2	
	ACP1	NOX1	
	LOC102723407	CCR9	
	MIR31	KRT19	
	CD74	KIF21B	
	MUL1	KDR	
	AKR1B10	HTR3A	
	CD47	TIMP2	
	BMP7	FGFR1OP	
	HSPA2	SIAE	
	PDGFRB	CCL4	
	IAPP	ITGAE	
	HES1	XRCC1	
	SAA1	EPCAM	
	IRAK1	MUC4	
	ITGB1	HPRT1	
	KRT7	AHR	
	MICB	TLR8	
	PAK1	GUCY2C	
	NR1H4	IL18R1	
	FKBP5	IGF1R	
	ENG	DCC	
	GZMB	GUSB	
	TPM3	ABCG2	
	XRCC3	BCL10	
	TFF1	MIR203A	
	GLI1	BMP6	
	ACKR1	IL9	
	FPR2	EBI3	
	DAP	TNFRSF4	
	GNA12	PLAUR	
	JAK2	LGR5	
	IRF5	PRKCD	
	HNF4A	TLR7	
	CXCR1	MIR193A	
	LTF	RAC1	
	STAT4	VWF	
	ETS1	HDC	
	HLA-DRA	MIR31	
	TYK2	ICAM3	
	LRRK2	NR1H2	
	ACTA2	NDUFA13	
	PRKCB	MAPK3	
	PLAU	PTH	
	DNMT3A	TMEFF2	
	ERN1	MIR200B	
	MSH2	TNFRSF10A	
	TLR5	TNFRSF10B	

	IL37	BRIP1	
	RUNX3	CFHR1	
	XBP1	MIR34C	
	PMP22	MIR215	
	TSLP	MIR451A	
	CRHR1	IBD25	
	DEFB103B	ACKR2	
	SFTPD	NCF1	
	MIR499A	LOC110806262	
	LYZ	ITLN1	
	DEFB103A	PIGR	
	PLA2G2A	CTSD	
	DUOX2	IDO1	
	CTNNBL1	ICAM2	
	CNR1	KLRC4	
	DDX58	BIRC5	
	ISYNA1	OCLN	
	CYP27B1	GATA3	
	IL20	ANXA5	
	TERF2IP	IGSF6	
	BIRC2	TOR1A	
	MIR150	CDH3	
	CD24	CHI3L1	
	MIB1	GAPDH	
	CA2	KRT18	
	HAVCR2	CFTR	
	SERPINH1	CD46	
	DUSP1	F2R	
	PDCD1	SCG5	
	PECAM1	MIR195	
	IDH1	IL18BP	
	SMO	ITGB1	
	CCL21	CYCS	
	ITGAM	HLA-G	
	ITGAX	ENTPD1	
	IGHG3	H2AX	
	NT5E	CYP1A1	
	IL11	CX3CR1	
	IL9	CASP8	
	NEDD9	MMP14	
	NAMPT	IL16	
	ABCC4	AMACR	
	NTN1	SP140	
	F5	TBX21	
	CXCL13	BIRC3	
	H2AFX	RUNX3	
	TGM2	AP3B1	
	CFLAR	ABCC2	

	NR0B2	PARP1	
	CLDN1	AIRE	
	IL23R	PDE4A	
	MST1	GPX2	
	FCGR2A	SRSF6	
	IL7R	EP300	
	CCR6	MIR26A1	
	IL1R2	CYP1A2	
	IL27	SERPINA4	
	CFB	IGF2	
	IL6R	CAMP	
	CHP1	TEK	
	KRAS	BUB1	
	IL23A	BUB1B	
	ARSA	PTPRJ	
	STAT6	DLC1	
	MUC2	PTPN12	
	IL17F	RAD54B	
	CD80	FLCN	
	CDKN1A	MCC	
	CXCL12	MIR34B	
	SOCS3	MIR100	
	TACR1	MIR342	
	MEFV	CRCS11	
	MMP7	CRCS2	
	SLC6A4	CRCS5	
	IL7	CRCS6	
	PTGDS	CRCS9	
	CDR3	CRCS7	
	TRPV1	CRCS8	
	SLC12A9	CCL25	
	CD86	MIR424	
	SLC3A2	MAPK1	
	LPL	TCF7L2	
	MIR145	SLC15A1	
	NFKBIA	PSMB9	
	DEFB4A	CD163	
	CYP3A5	MMP10	
	IL15	HDAC9	
	DEFB4B	NT5E	
	LOC105369230	BDNF	
	BIRC3	FOSL1	
	NQO1	MIR24-1	
	MAP3K8	HSD11B1	
	GDE1	RHOA	
	RABGEF1	RB1	
	UGT1A1	PSMB8	
	MIR122	PCNA	

	APOH	LCK	
	NAT1	MIR29A	
	WG	TNFRSF18	
	CEACAM5	MIR223	
	CDH11	CLDN1	
	CHEK1	PRSS1	
	BUB1B	CARD8	
	CD19	CD24	
	RAD51	TTC37	
	RAP1A	MIR142	
	RARRES3	HSP90AA1	
	SERPINF1	SRP54	
	IDUA	ANXA1	
	SLPI	CEACAM5	
	SST	TNFRSF9	
	SDC1	VIM	
	HSD11B2	CASP9	
	ST14	MUC3B	
	LRP6	ENSG00000278769	
	INSR	MAP2K1	
	KCNA3	MCL1	
	KDR	CCL26	
	IRF1	TNFSF10	
	IL3	RNF114	
	MTR	BBC3	
	BMS1	PDGFRA	
	F2RL1	GPBAR1	
	TNFSF13B	TMEM201	
	SUB1	CFLAR	
	TGFB3	EGR1	
	TG	SLC26A3	
	TGFA	XBP1	
	TNFRSF11A	MIR125A	
	PLA2G6	CTSB	
	CDH1	EPHX1	
	NOTCH1	FHIT	
	PTPRC	CDC42	
	IFIH1	PROCR	
	IL17D	KRT8	
	MLH1	MDM2	
	IL21	MTOR	
	MICA	C3	
	FOXP3	AK2	
	MGMT	EPB42	
	MIR146A	CALR	
	TIMP1	FLT1	
	RAG2	CD274	
	SOCS1	TRAF6	

	MAP2K7	TLR10	
	CTGF	NAT1	
	S100A1	MIR17	
	MYDGF	NFATC1	
	VIP	MIR144	
	CCR3	MIR3936	
	SFRP1	REG3A	
	KRT20	LRP6	
	STAT5A	GGT1	
	CRH	LRRK2	
	MDM2	PRF1	
	MIR21	MIR30A	
	APEX1	LGALS4	
	EPHB2	FUT3	
	B2M	ATM	
	FCGR3B	CRHR2	
	EBI3	DNMT3B	
	LGALS3	MUC16	
	MIP	TGFBR2	
	CCR7	MMP12	
	SEC14L2	MTR	
	DNAH8	GAS5	
	VCAN	DAPK1	
	CTNNB1	ADA	
	TLR7	BLOC1S6	
	IL17C	MIR9-1	
	PLB1	MIR199A1	
	CCL4L1	CRH	
	ACACA	IL25	
	CD55	C1S	
	SOX2	SULT1A1	
	CX3CL1	CDK2	
	SAT1	TYMP	
	NFKB2	CDK4	
	ODC1	FGF10	
	P4HB	ESR2	
	FOXO3	OR5V1	
	CCL4L2	NCAM1	
	NXF1	CFHR3	
	TWIST1	CFHR5	
	HLA-G	PSMD4	
	YWHAZ	IL4R	
	USO1	CCT5	
	IL12B	APEX1	
	SLC11A1	MIR19A	
	VCAM1	IFNGR2	
	PTPN22	SP1	
	ITGAL	IL12RB1	

	IL18R1	TDGF1	
	CD28	CCKBR	
	IL22	ITGA2	
	NR1I2	NQO1	
	S100A9	U2AF1	
	MIR155	NF1	
	TAC1	SLAMF1	
	MMP1	PPM1L	
	MMP3	ADGRE3	
	IL33	RNF186	
	NR3C1	TXN	
	IL16	HLA-DPB1	
	MPZ	BMP4	
	TGFB2	PROM1	
	FCGR3A	SNAI1	
	VDR	HLA-E	
	LCN2	MIR124-1	
	HSPA4	EXO1	
	HLA-A	COX5A	
	TLR1	CFI	
	TGFBR2	KLF4	
	CXCR3	PNLIP	
	HGF	DLAT	
	CD34	CFHR4	
	GORASP1	TGFBR1	
	WNK1	HSPA8	
	LEP	CD59	
	PLA2G1B	CRHR1	
	ISG20	MIR320A	
	SERPINA1	FCGR1A	
	PPARA	PPARA	
	CLU	TIMP3	
	CYP3A4	CBR3-AS1	
	SGSM3	ITPA	
	CFTR	MIR200A	
	CDH13	MIRLET7E	
	CHI3L1	UBE4A	
	CD68	IGFBP3	
	BRAF	CD209	
	HSPA1B	NUP210	
	HSPA1A	CLDN7	
	CCL5	F11R	
	PRKN	CYLD	
	LEPR	RETN	
	LRP5	AGER	
	MET	RPS6KB1	
	SERPINE1	SLC2A1	
	IL4R	MLKL	

	FLT1	FADD	
	CD163	ERCC6	
	ABCG2	MIR23A	
	F2	MIR196A1	
	RASSF1	CNR1	
	EGR1	PIK3R1	
	FASN	JAK1	
	CXCR6	MIR4435-2HG	
	STAT1	MIR4284	
	TFF3	CCNA2	
	PROM1	UGT1A1	
	GH1	FASN	
	CXCL1	TPI1	
	RELA	ALPI	
	CXCR2	MIR642A	
	IL2RA	WAS	
	IL1R1	YAP1	
	IGF2	CHEK2	
	RBM45	KIT	
	ABCB1	CCL24	
	TBC1D9	EDN1	
	CD14	LRP5	
	IL13	MIR15A	
	PPARG	XRCC6	
	CDKN2A	TF	
	HPGDS	NOS3	
	APC	STAT5B	
	MBL2	NUDT15	
	MTHFR	TNFRSF11B	
	TNFRSF1B	TRPV1	
	FHIT	HLA-DRB9	
	GSTM1	IFIH1	
	SLCO6A1	FOXO3	
	CALCA	CD36	
	PTPN11	MIR10B	
	HLA-B	MIR139	
	XRCC1	MIR338	
	GSTP1	MIR18A	
	SYT1	IL21R	
	MYD88	CDX1	
	IL5	XRCC5	
	CXCL10	CX3CL1	
	ANXA1	ARG1	
	NAT2	MRAP	
	GSTK1	ALG9	
	PON1	PLA2G4F	
	ATN1	EPC1	
	CX3CR1	METTL9	

	CYP2B6	IGSF6-DREV1	
	CCND1	KAT5	
	ALB	TSLP	
	CAT	CD69	
	CASP1	BACH2	
	CD40LG	ADORA3	
	PTGS1	AFAP1-AS1	
	HSP90AA1	TCN2	
	REG1A	AMBP	
	IFNA13	SOD2	
	IFNA1	TGFB3	
	S100A8	SFRP2	
	HRAS	GUCA2A	
	CCL3	NLRC4	
	ABCB6	ITK	
	ENO1	TGFB2	
	HMGB1	DNMT3A	
	TERT	ERCC1	
	GLB1	WNT2	
	NOD2	MALAT1	
	STAT3	NEAT1	
	CXCL8	TP73	
	MMP9	GPX1	
	CASP3	MIR149	
	SMAD3	MIRLET7I	
	TNFRSF1A	MIR675	
	IL17A	CYP1B1	
	CTLA4	LOX	
	COX2	MIR29B1	
	SPP1	PON1	
	CRP	ALK	
	MIF	DST	
	IL18	AGT	
	BCL2	MIR146B	
	S100B	CHEK1	
	TLR9	GUCA2B	
	CD40	KDM4C	
	NLRP3	VWA2	
	FAS	SEPSECS	
	MAPK1	FURIN	
	TLR3	TNFRSF6B	
	HLA-DQB1	AGR2	
	AICDA	OTUD3	
	GSTT1	ALOX15	
	HMOX1	DENND1B	
	IDO1	MMP8	
	EGFR	LAMB1	
	POLDIP2	BMP7	

	RNF19A	BECN1	
	MAPK14	CSF1	
	CRK	SNAI2	
	BAX	KRT15	
	AHR	DSG2	
	CD44	TOP1	
	CCL4	CYP2E1	
	LTA	SLC17A5	
	KNG1	ANXA2	
	NGF	TWIST1	
	NOS3	CDKN2B-AS1	
	NFE2L2	NPSR1	
	MMP2	JAG1	
	COX1	MIR181A1	
	FOS	MIR27B	
	GRAP2	EPHA3	
	FLNB	GLI3	
	AHSA1	CD86	
	AIMP2	GRB2	
	HLA-DRB1	HTR4	
	ICAM1	ATG5	
	TP53	NHEJ1	
	PTGS2	FCGR2B	
	IFNG	GSTT1	
	IL1RN	FZD8	
	TLR2	F13A1	
	TRBV20OR9-2	MIR598	
	CCL2	CCN4	
	CCR5	CLDN8	
	ESR1	RASSF1	
	FGF2	BRINP3	
	ACE	BMI1	
	SOD2	THBS1	
	EGF	PRRT2	
	VEGFA	CDK5RAP1	
	CXCR4	ALLC	
	IL10	STX11	
	IL1B	FSCN1	
	MPO	EZH2	
	NFKB1	HSPA5	
	TLR4	MIR92A1	
	NOS2	LGALS3BP	
	IL2	SLIT2	
	IL1A	GSDMB	
	IL4	LINC01475	
	TGFB1	MUC17	
	RAC1	ABCB11	
	APOE	FGF19	

	LAMC2	SP100	
	TNF	BLOC1S1	
	IL6	GGT2	
		GGTLC3	
		MIR182	
		MIR103A1	
		RAD51	
		BRCA1	
		WNT3	
		ANTXR1	
		SLAMF6	
		DACT1	
		MIR130B	
		CTNNA1	
		CTNND1	
		CYP7A1	
		SIRT1	
		PLA2G2E	
		ERGIC1	
		HLA-DRB5	
		SULT2A1	
		SLC10A2	
		KNG1	
		HPS4	
		CXCR1	
		SERPINB5	
		ALPP	
		GLI1	
		LY9	
		PVT1	
		HOTTIP	
		HOXA11-AS	
		PKM	
		RAF1	
		WNT3A	
		STXBP2	
		RAB27A	
		RECK	
		MUC19	
		FLT4	
		MDH2	
		ENPP7	
		UGT1A6	
		ITGAX	
		MAPK9	
		BSN	
		RDX	
		MIR423	

		ABCB4	
		STK11	
		PSMG1	
		SOX2	
		CCNB1	
		ERCC2	
		WNT16	
		HFM1	
		SDSL	
		MIR132	
		SETDB1	
		PCCA	
		SLC10A1	
		DNAL1	
		SCTR	
		CYP8B1	
		ATP8B1	
		LCOR	
		DDX53	
		MT-CYB	
		MIRLET7B	
		MIR542	
		MIR1537	
		G6PC3	
		SYNE1	
		MMP26	
		MSN	
		CDK1	
		SLC44A4	
		LMNA	
		ZNF148	
		PUS10	
		MIR30C1	
		PTGER2	
		EDNRA	
		NRON	
		IRF8	
		MIR185	
		MIR99A	
		MIR486-1	
		GJB6	
		AXIN1	
		MRE11	
		COMT	
		WNT5A	
		MMP11	
		FABP2	
		APOB	

		SOX9	
		CEACAM1	
		WNT4	
		HBEGF	
		DVL1	
		MAP2K5	
		MAPRE1	
		HOTAIRM1	
		SCARNA5	
		LINC00460	
		CCNY	
		MUC20	
		CSK	
		WNT1	
		LEF1	
		MAP2K4	
		WNT2B	
		VEGFD	
		HOTAIR	
		CYP24A1	
		HTR2A	
		ZEB1	
		P2RX7	
		TREX1	
		RARB	
		ABCC1	
		GDF15	
		STAT5A	
		HAVCR2	
		MIR22	
		RAG2	
		CDH2	
		OPRM1	
		SFRP1	
		RSPO1	
		CEACAM7	
		MAF	
		MIR224	
		ATF3	
		NAMPT	
		ZFP36L1	
		SLAMF7	
		ADAM30	
		FIP1L1	
		CYBA	
		ITGA6	
		ANTXR2	
		TYR	

		SERPINH1	
		IGFBP2	
		CASP5	
		PDCD4	
		TRAF3IP2	
		VEGFC	
		CCL21	
		TNFRSF14	
		NME1	
		MSH3	
		ELAVL1	
		ZPBP2	
		IGHE	
		DHFR	
		UNC13D	
		ABCA1	
		TAP1	
		CREB1	
		CDK6	
		PRODH	
		ERBB4	
		YES1	
		UGT1A7	
		MIRLET7F1	
		HTR1A	
		CDKAL1	
		CD27	
		CEACAM3	
		FCAR	
		ICOS	
		NRP1	
		RHOD	
		ERBB3	
		EPHB2	
		PIK3R2	
		CDC25C	
		OGG1	
		RAG1	
		CA9	
		E2F1	
		WNT5B	
		WNT11	
		TBX1	
		CHSY1	
		HAVCR1	
		XRCC3	
		NAT9	
		PRDM10	

		MIR191	
		MIR345	
		MIR454	
		MIR296	
		CYTOR	
		MIR106A	
		SPRY4-IT1	
		DSP	
		IKZF1	
		PTK2	
		SETD2	
		ECM1	
		PRKCA	
		SERPINB2	
		KIAA1109	
		BANK1	
		TPO	
		MUC7	
		MIR30E	
		SI	
		TRPA1	
		GSK3B	
		ANGPT1	
		PRKD1	
		IFI27	
		NOTCH4	
		LAMP1	
		MIR574	
		MIR455	
		MIR135B	
		LITAF	
		TKT	
		PIK3CB	
		TOP2A	
		DLD	
		MST1R	
		TOLLIP	
		FZD7	
		BLK	
		AOC1	
		ILK	
		ADAD1	
		BCL2L11	
		CCND3	
		IL20	
		SMURF1	
		GNA12	
		GATD3A	

		IL2RG	
		BAD	
		UMPS	
		CD82	
		TUG1	
		UCA1	
		PTENP1	
		ATF6	
		EZR	
		TOX	
		CTBP1	
		IL13RA2	
		NTN1	
		IL1RL2	
		AKT2	
		CCND2	
		PLK1	
		ZEB2	
		BAK1	
		PIK3R3	
		KIF1B	
		S100A4	
		PTGES2	
		FERMT3	
		PTPRU	
		TPX2	
		GRPR	
		WNT6	
		RUNX1T1	
		WNT8B	
		PDPN	
		FERMT2	
		PIF1	
		MEG3	
		CASC2	
		KCNQ1OT1	
		SNHG12	
		FER1L4	
		TINCR	
		XIST	
		SOX2-OT	
		DANCR	
		SNHG5	
		SNHG1	
		HULC	
		CRNDE	
		MIR10A	
		MIR375	

		MIRLET7G	
		HNF1A-AS1	
		MIR101-1	
		MIR107	
		LINC00261	
		ZFAS1	
		PCAT1	
		MIR24-2	
		RPL34-AS1	
		SNHG16	
		SNHG20	
		HOXA-AS2	
		BCAR4	
		TUSC7	
		SNHG6	
		ZEB1-AS1	
		LINC-ROR	
		BLACAT1	
		FEZF1-AS1	
		CCAT1	
		TMEM238L	
		PRNCR1	
		HEIH	
		GAPLINC	
		CCAT2	
		NPTN-IT1	
		PANDAR	
		NCRUPAR	
		BANCR	
		GHET1	
		DUXAP9	
		TP53COR1	
		LNCRNA-ATB	
		LOC106029312	
		SERPINF2	
		TET2	
		NRIP1	
		CASP7	
		MIR23B	
		MIR28	
		MIR324	
		MIR29C	
		MIR151A	
		WIF1	
		LACTB	
		TNFSF4	
		CLDN5	
		ADAMTS13	

		HSPA6	
		ACVR2A	
		PTPRT	
		DNTT	
		IL1R2	
		HDAC2	
		SLC29A1	
		IGFBP7	
		STUB1	
		PSMA7	
		ST6GAL1	
		EREG	
		CLCA1	
		CSE1L	
		MT-CO2	
		THY1	
		LEPR	
		TFRC	
		MIR29B2	
		RHO	
		MB	
		MTRR	
		CHAT	
		AQP8	
		INPP5E	
		DDIT3	
		COL14A1	
		DES	
		EPHX2	
		MTHFD1	
		SAG	
		ERAP2	
		HSPA1B	
		EPHA2	
		PRKCZ	
		ARPC2	
		WASHC5	
		ABCB5	
		GSR	
		TNIP1	
		CD63	
		NFKBIL1	
		DDC	
		PPARD	
		GCNT3	
		GADD45A	
		IL17REL	
		WT1	

		CR1	
		C7	
		SIGLEC5	
		MIR615	
		MIR186	
		PRMT1	
		EIF4G1	
		FBXW7	
		MTA1	
		TPBG	
		DKK1	
		FABP4	
		PTPN13	
		FUT4	
		PTPA	
		MIR133B	
		MIR129-2	
		MIR137	
		UBE2L3	
		MIR122	
		EDNRB	
		HOXA13	
		FZD4	
		HDAC1	
		CASP2	
		RRM2	
		IRS1	
		P2RY12	
		PPP2R1B	
		DIABLO	
		CACNA1G	
		PLK4	
		SOX5	
		FOLH1	
		FZD6	
		ADORA1	
		LRP1	
		IGF2R	
		HTR7	
		RALA	
		PXN	
		PSMB7	
		E2F4	
		SAT1	
		MAP2K7	
		TCF3	
		WNT10A	
		TFDP1	

		TIAM1	
		PIK3R5	
		CPE	
		SEMA4A	
		ROS1	
		MELK	
		EPHB6	
		STK24	
		TCF7	
		FZD1	
		FZD3	
		FOXM1	
		TPH1	
		PDGFD	
		SLCO1B3	
		TCOF1	
		QKI	
		KLF5	
		FZD10	
		MBD4	
		ACSL5	
		BNIP3	
		TRIM28	
		LASP1	
		PTTG1	
		SLC16A7	
		RALGDS	
		TCF7L1	
		DCLK1	
		FPGS	
		LGR6	
		XRCC2	
		PLD3	
		PHLPP2	
		PMAIP1	
		PMS1	
		SIL1	
		KMT2C	
		EIF5A2	
		NFYB	
		AGO2	
		AKAP12	
		UHRF1	
		USP28	
		PHLPP1	
		SLC45A2	
		PZP	
		HLTF	

		GPA33	
		CDCP1	
		LLGL1	
		RNF43	
		EVL	
		FAT4	
		TNS4	
		HAPLN3	
		ZNF217	
		HTR3E	
		MACC1	
		PSG2	
		CSMD3	
		SEPTIN9	
		USP40	
		CLDND1	
		KRTDAP	
		P3H3	
		VWA8	
		MRGPRE	
		KRTAP9-2	
		COLCA2	
		COLCA1	
		TP53TG1	
		LINC00472	
		MIR96	
		MIR128-2	
		FBXL19-AS1	
		MIR339	
		MIR181A2	
		MIR32	
		HOXB-AS3	
		MRGPRG-AS1	
		MIR183	
		SNHG3	
		MIR328	
		SCARNA6	
		MIR7-3	
		SNHG17	
		SNORA54	
		MIR497	
		BCYRN1	
		FTX	
		MIR372	
		MIR422A	
		SOX21-AS1	
		MIR95	
		MNX1-AS1	

		DLEU7-AS1	
		CAHM	
		FAM83H-AS1	
		LEF1-AS1	
		LINC-PINT	
		POU5F1P4	
		MIR625	
		HIF1A-AS1	
		SBDSP1	
		CASC11	
		RNY1	
		CASC8	
		MACROD2-AS1	
		VIM-AS1	
		LINC00659	
		LINC01567	
		LINC01133	
		FOXP4-AS1	
		DPP10-AS1	
		RNY3	
		NORAD	
		NNT-AS1	
		SLC25A25-AS1	
		HIPK1-AS1	
		DLGAP4-AS1	
		GABPB1-AS1	
		BACE1-AS	
		C10orf143	
		CASC19	
		LINC00858	
		ZNF582-AS1	
		LINC01507	
		CLMAT3	
		GSEC	
		EHHADH-AS1	
		MAMDC2-AS1	
		DUXAP10	
		RBM5-AS1	
		PURPL	
		MROCKI	
		AOC4P	
		LINC00538	
		WSPAR	
		TUSC8	
		PINCR	
		NCF4-AS1	
		LAMC1-AS1	
		ENSG00000228082	

		BTG3-AS1	
		LINC02223	
		LINC02446	
		LINC02086	
		LINC01617	
		ENSG00000237588	
		ENSG00000260196	
		SEC63P1	
		ENSG00000257241	
		ENSG00000266680	
		DACOR1	
		FGF7P5	
		IBD19	
		TNFSF13B	
		SCG2	
		RPL39P28	
		CYP21A2	
		SCYL1	
		PI3	
		F2RL3	
		CHGB	
		INSL6	
		REN	
		RPS14P1	
		ENO2	
		PTGER3	
		DNAH8	
		DEFB103B	
		TRA	
		GPD2	
		CYP3A5	
		RTEL1-TNFRSF6B	
		CCS	
		ACTA2	
		UCN2	
		KLRK1	
		PRDM1	
		C5AR1	
		TNPO3	
		DPF2	
		TH	
		TYRP1	
		BIRC2	
		PTX3	
		CFP	
		DAXX	
		LGALS9	
		FBLIM1	

		CD19	
		TNFSF11	
		FCN2	
		FCN3	
		CFHR2	
		KRT1	
		GOT1	
		AICDA	
		VDAC1	
		CCHCR1	
		BVES	
		JAZF1	
		SERPINB1	
		FOXP2	
		HLA-F-AS1	
		ZGPAT	
		PARK7	
		HLA-DRB3	
		FAF1	
		MUC13	
		KRT9	
		APOE	
		DEFB103A	
		IKZF3	
		LSP1	
		PLCH2	
		DAP	
		TNFSF8	
		SLC2A4RG	
		MMEL1	
		NXPE1	
		ENTR1	
		C5orf66	
		LINC00598	
		WASL	
		RPN2	
		DCT	
		CSN1S1	
		TNFSF12	
		STK39	
		PLA2G7	
		LIF	
		SEC16A	
		S100B	
		PTGES	
		IL13RA1	
		SATB2	
		TPPP	

		CEP72	
		FCGR2C	
		MROH3P	
		HAMP	
		CD3G	
		CD151	
		GCH1	
		METAP2	
		ALG3	
		SSR2	
		TRDMT1	
		CTDSPL	
		PSMC3IP	
		NEK5	
		IDNK	
		IGLL5	
		MT-RNR2	
		HSD11B2	
		SERPINA1	
		USP4	
		KCP	
		AFP	
		JUP	
		TAGLN	
		MSH5	
		NOS1	
		APEH	
		MDK	
		KIR3DL1	
		C5	
		PFKFB4	
		IP6K2	
		IP6K1	
		SBNO2	
		GZMA	
		COL1A1	
		NTF3	
		LYZ	
		HSPA1A	
		TRAF2	
		GJA1	
		G6PD	
		PRG2	
		RETNLB	
		PSORS1C1	
		ANPEP	
		MIR16-1	
		TARDBP	

		CTSC	
		TCN1	
		ARMH3	
		MIR135A1	
		MIR378A	
		MIR103A2	
		MIR582	
		MIR490	
		RCL1	
		HERC2	
		FLOT1	
		TRIM39	
		MYB	
		HDGF	
		NLRP7	
		TXK	
		KIR2DL1	
		GAL	
		USP12	
		INSL4	
		TBXT	
		MIR196A2	
		DUOXA2	
		GPR65	
		C1orf141	
		IL7	
		PRDX1	
		P2RX3	
		PADI4	
		CYBB	
		PNMT	
		GMPPB	
		NKX3-1	
		UBA7	
		UTS2	
		PIM3	
		SERINC3	
		STMN3	
		EXOC3	
		SNAPC4	
		OLIG3	
		AAMP	
		ZBTB46	
		AMIGO3	
		PRXL2B	
		LOC100996583	
		GLP2R	
		MME	

		RNASEH2C	
		CCL17	
		GPSM3	
		PBX2	
		CYP4F3	
		GPR12	
		LBR	
		PHB	
		IER3	
		CREM	
		SDC1	
		REG1A	
		PRPF8	
		COG6	
		HNMT	
		MIR210	
		ADCY10	
		NGFR	
		CLIC1	
		IL15RA	
		LINC00243	
		HMGB2	
		WASHC4	
		STING1	
		ASGR1	
		IKBKB	
		CLDN3	
		RNASE2	
		PERP	
		GSN	
		LPL	
		NAGLU	
		IFNAR1	
		TUBB6	
		CD244	
		PTPRS	
		SULT1A3	
		S1PR1	
		SLC22A23	
		SFTPD	
		MAGI1	
		MIR499A	
		DDR1	
		CACNA2D1	
		VARS2	
		COL13A1	
		GCKR	
		GTF2H4	

		ACTR3B	
		CPXM2	
		PRRC2A	
		KPNA7	
		CAVIN1	
		CALHM6	
		MIR26B	
		HCG9	
		HCG26	
		MIR588	
		CCR2	
		THADA	
		IL1RAP	
		RARRES2	
		BDKRB2	
		ABL1	
		TERF2	
		CCL22	
		OSCAR	
		PAK1	
		CD1A	
		TEP1	
		CLU	
		FPR1	
		SULT1A2	
		NR5A2	
		ALDH1A1	
		CXCL11	
		ACHE	
		IKBKG	
		ADORA2A	
		S100A1	
		MFGE8	
		CBL	
		TMPO	
		GSDMA	
		HPS3	
		MTM1	
		FTCD	
		ASPH	
		PPP2R2C	
		PPM1K	
		MSLN	
		MUC5B	
		ANKH	
		ASGR2	
		GOLM1	
		SIGLEC8	

		PPFIBP2	
		HOGA1	
		AGBL2	
		ZNF354A	
		TCFL5	
		PAM16	
		LIN54	
		ISG20L2	
		AGBL3	
		CDH26	
		MUC15	
		USP50	
		MUCL1	
		C6orf62	
		MUC21	
		KAAG1	
		FRG2C	
		MIR4741	
		MT-TF	
		FCRL3	
		KIR2DL3	
		PRDX4	
		ZMIZ1	
		RMI2	
		TRB	
		FKBP5	
		PSD	
		TACR2	
		DAB2	
		TPM1	
		LBP	
		CISD1	
		PTGDR2	
		SMARCB1	
		SLC3A2	
		IRF4	
		RHOH	
		ACP1	
		ABCB8	
		FCGRT	
		CCL19	
		POU5F1	
		PSMA6	
		RUNX1	
		PPP1R14A	
		MRC1	
		ITGB7	
		SIGIRR	

		TNFRSF8	
		LALBA	
		GART	
		CD34	
		SAA4	
		DRD2	
		IFNAR2	
		FGF23	
		HLA-T	
		LST1	
		EPN3	
		AQP4	
		AQP7	
		MAGI2	
		ADRA2A	
		TRAF1	
		FLNA	
		CYP27B1	
		RAVER2	
		CAVIN3	
		PLIN2	
		HLA-DQA2	
		PRKCB	
		CNTF	
		HPSE	
		G6PC	
		ETS2	
		IL24	
		SYP	
		TRG	
		ANGPT2	
		CAMK4	
		RBP4	
		VIL1	
		FYN	
		USF1	
		MAML2	
		LIME1	
		ARHGAP45	
		RBP3	
		HLA-DMA	
		TRPV4	
		LTB	
		ALDOB	
		SGF29	
		CA2	
		DEFA6	
		FLG	

		RNF128	
		SLC6A14	
		KHDRBS3	
		APOA4	
		NBN	
		TMPRSS6	
		ADCYAP1	
		TNFSF14	
		CCR7	
		ANXA7	
		PMEL	
		CYP2J2	
		IL17RD	
		MCM2	
		BLVRB	
		ELF3	
		LTBR	
		STX2	
		CASP14	
		TFAP2A	
		ACADS	
		PARD3	
		CXCL3	
		PTGDS	
		IGES	
		SMARCA4	
		RNF5	
		IFITM3	
		HLA-DMB	
		HLA-DOB	
		DEFA1	
		KLKB1	
		CCN1	
		HLA-DOA	
		BCL3	
		POSTN	
		PF4	
		TBXAS1	
		PPBP	
		SOD3	
		BCL2L12	
		LYRM4	
		ADCY7	
		MTMR3	
		HORMAD2	
		STC1	
		TICAM1	
		MECP2	

		FEN1	
		ADAR	
		MIR124-3	
		CXCR5	
		UBC	
		EIF2S1	
		PSMB10	
		IL36A	
		RARG	
		PITX1	
		IL1F10	
		GABPA	
		MAFK	
		IL36B	
		RPL35P3	
		ITGA5	
		CXCL13	
		CEBPB	
		C1GALT1C1	
		ADAM10	
		FFAR2	
		SYVN1	
		AGFG1	
		BLOC1S2	
		DPEP1	
		NTSR1	
		HTRA1	
		CYSLTR1	
		RPS2P34	
		LOC442427	
		HSP90B1	
		MGAM	
		MAP3K8	
		SLC16A1	
		EED	
		PHOX2B	
		MAPKAPK2	
		PPP5C	
		TRIB1	
		TSPAN33	
		CIDEB	
		CD5	
		CCRL2	
		HCRTR1	
		CCR4	
		GABBR1	
		C2	
		ATRIP	

		TNC	
		SHC1	
		KHDRBS1	
		HSPB1	
		CA1	
		GZMM	
		FOXE1	
		LEPQTL1	
		IFNB1	
		MAD2L1	
		NPHP1	
		HSF2	
		TNFRSF10D	
		PRKN	
		TNFRSF10C	
		NACA	
		CSNK1A1L	
		FKBP15	
		FAM215A	
		BORCS8-MEF2B	
		OCTN3	
		FOSL2	
		TNFSF18	
		TAGAP	
		RIPK3	
		TRIM21	
		CLSTN2	
		DDIT4	
		CAV2	
		CAV3	
		LRG1	
		DEFB104A	
		FLVCR1	
		CD160	
		NCF2	
		CPOX	
		LAG3	
		ALDH2	
		ACAT2	
		LGALS2	
		TXNIP	
		MIR206	
		KCNQ1	
		CBS	
		APAF1	
		ATF2	
		TRAP1	
		NTAN1	

		KIR2DS2	
		KIR2DL2	
		CD48	
		GIMAP5	
		CD1D	
		SLPI	
		ANKRD55	
		INTS11	
		AHSG	
		DSG1	
		TNFAIP6	
		SCNN1B	
		RRM2B	
		SDHA	
		HSD17B10	
		HMGA1	
		SCNN1G	
		TFPI	
		SERPINF1	
		PTPRO	
		CYC1	
		KCNE3	
		SLC18A3	
		CITED2	
		LMAN1	
		GTF2E2	
		RHOB	
		AQP9	
		PDCD1LG2	
		IFITM1	
		MXI1	
		EYA4	
		TUSC3	
		UQCRQ	
		TTBK2	
		NMB	
		GPR55	
		GAS7	
		SERPINA12	
		NEK6	
		MGAT5	
		ACSM3	
		SMOX	
		MARVELD2	
		CCL16	
		APBA1	
		CADM2	
		NUCKS1	

		RC3H1	
		BAHD1	
		ANP32E	
		TAC4	
		MT-ND4	
		S100Z	
		IGHG3	
		EXOC3-AS1	
		PRAC2	
		MIR4728	
		LOC110594336	
		KIR3DL2	
		PDGFA	
		KSR1	
		MPG	
		GALC	
		CTSW	
		CRTC1	
		IL22RA2	
		NR4A1	
		AZU1	
		SGK1	
		VNN1	
		CXCL6	
		ITGB3	
		CTSL	
		PTGIR	
		GPX4	
		ADCY3	
		CARD11	
		ATF4	
		RIT1	
		HDAC7	
		RPS6KA4	
		LNPEP	
		TNNI2	
		REV3L	
		RGS14	
		PMPCA	
		ITIH4	
		CD226	
		FADS2	
		PNKD	
		ITPKA	
		FIBP	
		CALM3	
		NDUFAF1	
		MANBA	

		BRD7	
		RASSF5	
		SKAP2	
		CTDSP1	
		CEP250	
		LPXN	
		DUSP16	
		CD6	
		ACSL6	
		PDLIM4	
		SLC7A10	
		PLCL1	
		CRTC3	
		MSTO1	
		SEMA6D	
		GPR18	
		CHP1	
		ZNF365	
		NDFIP1	
		ATXN2L	
		SLC39A11	
		CPEB4	
		SFMBT1	
		DOK3	
		FCRLA	
		TMBIM1	
		ZNF300	
		SNX20	
		SNX32	
		ARHGAP30	
		TSPAN14	
		PHTF1	
		TMEM50B	
		RFTN2	
		NUSAP1	
		TTYH3	
		CCDC85B	
		YDJC	
		JRKL	
		PUSL1	
		CCDC116	
		ZNF831	
		NXPE4	
		C10orf55	
		AHSA2P	
		TRAF3IP2-AS1	
		IRF1-AS1	
		FLJ31356	

		IRF3	
		LAMP2	
		STX8	
		NFKB2	
		EPHB4	
		SUFU	
		BANF1	
		CAPN10	
		SLC35D1	
		ARFRP1	
		ZNF341	
		WDR6	
		MIR611	
		TEC	
		KRT17	
		ANG	
		BLZF1	
		FOXP1	
		DNAL4	
		OSM	
		DDX58	
		ADAM15	
		RING1	
		CYBC1	
		SUOX	
		FLNC	
		AIF1	
		ZNRD1	
		MX1	
		VIPR1	
		MIR130A	
		MIR429	
		CACNA1E	
		CD83	
		ARFGAP1	
		C4BPB	
		SLC23A3	
		RO60	
		PER3	
		BDH2	
		LRRC3C	
		GPR183	
		MEP1A	
		FOXO1	
		CASP4	
		SYK	
		PEX5	
		NFIL3	

		GBGT1	
		NEU1	
		CHRNA5	
		CUX1	
		KCNN1	
		DDX39B	
		ATP6V1G2	
		DEFA3	
		SLC9A1	
		ST14	
		SPI1	
		PRSS8	
		IL9R	
		ATP6V0A1	
		SCD	
		HADH	
		TERF2IP	
		ATG7	
		ACAP1	
		OVGP1	
		CHD4	
		CD38	
		VCL	
		CORO1A	
		TRAF3	
		IL17C	
		OSMR	
		BDKRB1	
		GP2	
		ARRB2	
		FLI1	
		CD2	
		PTPN6	
		TNFRSF17	
		PLEK	
		SSRP1	
		FGL2	
		SUN2	
		AKR1A1	
		ETV5	
		CD207	
		KEAP1	
		RXRB	
		CD81	
		SH2B3	
		GRB7	
		ABCD4	
		DNMT3L	

		CTDP1	
		SLC39A7	
		HSD17B8	
		MYT1L	
		CDK12	
		FNBP1	
		IL22RA1	
		NCKIPSD	
		NOP2	
		OR2H2	
		SLC48A1	
		ENGASE	
		MPIG6B	
		C3orf62	
		INS-IGF2	
		CCDC26	
		PHETA1	
		SNHG7	
		MSTO2P	
		FAS-AS1	
		TNK2	
		MLNR	
		IRF7	
		GAS6	
		PFKM	
		SLC23A1	
		SLC2A14	
		IL32	
		ARRB1	
		CAMK2G	
		MACF1	
		GIP	
		HLA-DRB4	
		CYP2A6	
		MCCD1	
		PROC	
		KCNMA1	
		GHSR	
		SLC39A8	
		APOBEC3G	
		SLC39A12	
		DCLRE1B	
		SELPLG	
		HLA-DQB2	
		PRRT1	
		SNHG32	
		AOC3	
		MDC1	

		IGHM	
		SLC19A1	
		CD300LF	
		MYDGF	
		C19orf33	
		RGN	
		SESN2	
		TIAL1	
		CSNK2B	
		EHMT2	
		BRD2	
		LSM2	
		B3GALT4	
		ATF6B	
		GPANK1	
		HLA-DRB6	
		CYP21A1P	
		HLA-DQB1-AS1	
		ALOX15B	
		IGSF3	
		CARD16	
		TRD	
		MIR19B1	
		MAP3K11	
		TRIM31	
		MLXIPL	
		TST	
		C1GALT1	
		DUSP1	
		CGN	
		HCG27	
		ENSG00000271581	
		APOA1	
		OTC	
		UCP2	
		IL17D	
		SECTM1	
		MMP19	
		DSC2	
		CMA1	
		H2AC21	
		CD84	
		GDNF	
		SKI	
		SLC22A2	
		SLC26A6	
		CXCR6	
		NFATC2	

		MARCKS	
		RXRA	
		APLN	
		AKR1B1	
		CYTH1	
		FBXW11	
		RUNX2	
		EDN2	
		TNPO1	
		MFSD2A	
		RBPJ	
		CUEDC2	
		INSL5	
		PRKAA1	
		DAG1	
		P4HA2	
		CNTNAP2	
		SYNGR1	
		CCL8	
		TENM3	
		CLEC16A	
		CTIF	
		PROX2	
		KRT38	
		AIM2	
		SMPD1	
		FAAH	
		CTSE	
		EIF3C	
		NUPR1	
		FAM92B	
		PFKFB3	
		SLC9A8	
		ASCL2	
		ZFP91	
		CIRBP	
		SPHK1	
		DBH	
		DDAH2	
		SYNGAP1	
		KIFC1	
		PPT2	
		WDR46	
		VPS52	
		FKBPL	
		NELFE	
		EGFL8	
		ZBTB12	

		SAPCD1	
		HCG23	
		HCG25	
		NONHSAG045982.2	
		HSALNG0049430	
		PTPN1	
		PIP5K1A	
		TNXB	
		CRB1	
		RAB5B	
		DHX16	
		CDSN	
		ATP6V1F	
		NSMCE2	
		NCR3	
		ABCF1	
		ZFP57	
		PPP1R10	
		ZNF687	
		GNL1	
		LRRC2	
		TCF19	
		NRM	
		ATAT1	
		PRR3	
		PSORS1C2	
		LY6G5B	
		C6orf47	
		HLA-H	
		SPATA48	
		HCG22	
		HLA-J	
		HCG18	
		HCG4B	
		TPI1P2	
		ZNRD1ASP	
		HLA-L	
		HLA-K	
		LINC01273	
		MICD	
		TRIM31-AS1	
		LINC01271	
		FLNC-AS1	
		HLA-W	
		RPL23AP1	
		LOC285626	
		STK19B	
		ENSG00000250264	

		MICE	
		HLA-U	
		RPL3P2	
		LINC02009	
		WASHC5-AS1	
		TSBP1-AS1	
		PAIP1P1	
		ENSG00000249738	
		ENSG00000230533	
		RN7SL636P	
		ENSG00000235620	
		ENSG00000271553	
		ENSG00000269667	
		ENSG00000270120	
		ENSG00000251136	
		lnc-IKZF1-6	
		ENSG00000272540	
		lnc-IRF5-3	
		ENSG00000242162	
		lnc-NBN-5	
		ENSG00000285040	
		LOC645266	
		RF00017-6602	
		RF00005-109	
		ENSG00000237669	
		ENSG00000227766	
		ENSG00000230521	
		NONHSAG019426.2	
		ENSG00000224163	
		piR-56133-186	
		LOC105372657	
		SELENBP1	
		MAZ	
		CHMP5	
		GC	
		AR	
		HMGCR	
		CNR2	
		HHIP	
		HRH1	
		BIN1	
		CCDC88B	
		NR3C2	
		SIK2	
		LILRB4	
		CCL13	
		MIR133A1	
		PAH	

		MRTFA	
		DSC1	
		IL34	
		ETV6	
		CXCL16	
		IKZF4	
		MIR346	
		PDZK1	
		MIR449A	
		SPINK1	
		PLAA	
		MAPK10	
		FAP	
		SLC11A2	
		DSC3	
		LY96	
		MIR590	
		RAC2	
		BMP2	
		KLF1	
		CST3	
		CDB2	
		PA2G4	
		PEBP1	
		PRKG1	
		ACAT1	
		CD247	
		PIK3CG	
		ABCC6	
		B3GAT1	
		WARS1	
		BCL2L15	
		NPY	
		UBE2N	
		NELL1	
		TIMP4	
		CFL1	
		CUL2	
		USP20	
		UBAP2L	
		TMEM258	
		EGR2	
		BRMS1	
		UBASH3A	
		TRIM39-RPP21	
		ADM	
		USP7	
		IRAK3	

		ENPP2	
		NUP107	
		LCT	
		ITGA1	
		ARHGEF6	
		REEP6	
		CAMK2A	
		RAD50	
		TUBB	
		THRA	
		FES	
		PRKAB1	
		PTGIS	
		PRKAR2A	
		SCNN1A	
		GLS	
		GALT	
		F12	
		CACNA1S	
		PDXK	
		PRKD2	
		PPP1CB	
		CIT	
		GRK6	
		HINT1	
		NEK9	
		CTSF	
		ATXN2	
		MAN1B1	
		AMT	
		ERN1	
		SLC25A20	
		IMPDH2	
		POFUT1	
		POLR1D	
		RAPGEF3	
		IL11RA	
		VAMP1	
		EFNB2	
		PLXNB1	
		SLC34A1	
		TMED10	
		SEC24C	
		CHRNB2	
		ANXA11	
		TNNT3	
		TUFM	
		UBE2G2	

		LAMB2	
		EFEMP2	
		PNPLA8	
		POLR2E	
		PPIF	
		PPOX	
		SLC26A4	
		PTPRK	
		PPP1R1B	
		STK36	
		HHEX	
		HDAC11	
		HOXA10	
		SENP1	
		HIBCH	
		ABI1	
		ADAMTS4	
		BATF	
		AUH	
		FCER1G	
		LTBP2	
		LZTR1	
		ARPC1B	
		NPEPPS	
		EMP2	
		NRCAM	
		RAB1B	
		TCAP	
		TCP1	
		PRPF6	
		SCNN1D	
		MUS81	
		HOXA11	
		NEUROD2	
		RPL3	
		CCNT1	
		AFF4	
		ASH1L	
		LHX3	
		UQCRC1	
		TRPM2	
		TOM1	
		HLA-F	
		DSE	
		THBS3	
		PPIL2	
		JDP2	
		KLC2	

		CLK2	
		GNPDA1	
		HIBADH	
		AGPAT1	
		LTBP3	
		BRAP	
		FLVCR2	
		ANAPC2	
		TOP3B	
		KIF3A	
		VAMP3	
		UBE2J2	
		PHB2	
		FLAD1	
		RBMX	
		CST6	
		NDUFAF3	
		RBM4	
		POLA2	
		SETD1A	
		PPA2	
		ITIH3	
		PASK	
		DBN1	
		HIPK1	
		NCAPD2	
		SART1	
		DPM3	
		EMG1	
		OLIG2	
		GDI2	
		CELSR3	
		ATP6V1G3	
		B3GNT2	
		B4GALT5	
		LPAR5	
		FDX1	
		CAMTA1	
		APOM	
		MED1	
		TNS1	
		LIMD1	
		TTLL5	
		FKRP	
		NRBP1	
		RALGAPA1	
		KDELR2	
		IFT172	

		PPP2R3C	
		SLC6A7	
		TAS1R3	
		SLC2A13	
		SLC16A10	
		STARD3	
		SON	
		GRHL3	
		RIN1	
		HIF3A	
		NDST2	
		NAB1	
		COG5	
		CPSF3	
		GLYAT	
		RPL37	
		RPL24	
		OLIG1	
		DAP3	
		CD248	
		ACTR1A	
		BAZ1A	
		B3GALT6	
		CARD6	
		AAGAB	
		APOBEC3A	
		ASAP2	
		ASIC2	
		ACYP1	
		ATP8B2	
		FAU	
		THEM4	
		THEMIS	
		UQCR10	
		USP19	
		USP21	
		WTAP	
		STK19	
		RCE1	
		PKIG	
		SNX27	
		ITGB1BP1	
		RAPGEF6	
		SH3PXD2B	
		PAPOLG	
		PYGO2	
		RBM17	
		KLHDC8B	

		RFT1	
		DAZAP1	
		RLN2	
		DAGLB	
		DRAP1	
		HBP1	
		HCN3	
		MPPE1	
		MRPL11	
		DIS3L	
		SF3B2	
		GPSM1	
		CH25H	
		GPN1	
		RPL26L1	
		RSPH4A	
		CDK11A	
		CDK11B	
		BBS1	
		AURKAIP1	
		LRCH4	
		MAN2A2	
		ARIH2	
		APOL6	
		TOR1B	
		TIPIN	
		YY1AP1	
		USP37	
		UCKL1	
		USP36	
		ZBTB40	
		UBE2Q1	
		TRIM8	
		PGAP3	
		PGS1	
		SLC25A28	
		NRBF2	
		SEC31B	
		PMF1	
		SLC12A9	
		REXO2	
		NDOR1	
		DIDO1	
		SDF4	
		SENP7	
		DNAJC27	
		MED24	
		CBLL1	

		ADO	
		ATP6V0E1	
		LYRM7	
		ERRFI1	
		ADGRL2	
		TRAPPC10	
		WIPF2	
		ING4	
		STAC2	
		DUSP8	
		RBM7	
		PRELID1	
		SPSB2	
		SDF2L1	
		SNAPC5	
		PLB1	
		PPP1R11	
		RAB24	
		P4HTM	
		QSOX2	
		INO80	
		ST7	
		FNIP1	
		NFKBIZ	
		RNPEPL1	
		COMMD7	
		MRPL20	
		DNAH17	
		RTF1	
		GPR25	
		CEP76	
		CEP192	
		DUSP12	
		RNF123	
		MYOZ1	
		DPH5	
		RSPH3	
		BCAP29	
		BAG6	
		LPCAT3	
		FBXL20	
		BNIP1	
		LY6G6F	
		FNDC3A	
		ASB8	
		ASCC2	
		ATAD3B	
		TNRC6C	

		TRIM26	
		UBLCP1	
		YTHDF1	
		UBAC1	
		TSPAN32	
		SLC9A4	
		ZNF384	
		TAPBPL	
		RBM4B	
		PNRC2	
		PLEKHG6	
		TATDN1	
		TBC1D8	
		TBKBP1	
		SHISA5	
		PWP2	
		QRICH1	
		TMEM199	
		LSM14A	
		LY6G6C	
		EAPP	
		NAALADL1	
		SGIP1	
		SBK1	
		SERBP1	
		CEP89	
		CENPO	
		MXRA8	
		NKD1	
		NT5DC2	
		OIP5	
		CCNL2	
		FAM177A1	
		FCAMR	
		FITM2	
		CASC3	
		ACAP3	
		ASB6	
		ANKZF1	
		BOD1	
		TRIM10	
		VARS1	
		ZC3H12C	
		ZNF142	
		UBTD2	
		YIF1A	
		VPS51	
		RNF145	

		NPAS4	
		ENDOU	
		THAP7	
		PRM2	
		IFNLR1	
		IFFO1	
		SNN	
		TCTEX1D1	
		KANSL2	
		RAVER1	
		TCHHL1	
		QPCTL	
		PSMG2	
		GRAMD1B	
		CMC1	
		CYTL1	
		DALRD3	
		NGRN	
		DYDC1	
		DYDC2	
		MIEN1	
		MIER1	
		GON4L	
		DONSON	
		DNAJC28	
		MXD3	
		DUPD1	
		MYRF	
		OAZ3	
		OLFML2B	
		GID8	
		GAL3ST2	
		CDC42SE2	
		MFSD9	
		FUT11	
		FYB1	
		ABHD16A	
		FAM53B	
		EVX1	
		EXD1	
		AMZ1	
		ANKRD30A	
		TRIM15	
		ZNF366	
		WDR43	
		ZC3H4	
		ZWILCH	
		PTRHD1	

		SPATA2	
		PPP1R18	
		PAXBP1	
		SKOR1	
		TMCO4	
		TMEM106C	
		C9orf78	
		NICN1	
		EIF1AD	
		NACC2	
		MUSTN1	
		ELOVL3	
		RPAP3	
		RPP25L	
		FRMD8	
		ACRBP	
		FBXO24	
		C11orf68	
		C6orf15	
		FAM118A	
		KRTCAP2	
		UQCC1	
		ZFPL1	
		YLPM1	
		LIX1	
		ZNF507	
		SUPT7L	
		IQCH	
		TCTA	
		PCMTD2	
		C5orf24	
		LY6G6D	
		CNEP1R1	
		RIC8B	
		EHBP1L1	
		DXO	
		MIER2	
		GPATCH1	
		GPATCH2L	
		GMEB2	
		NUDT13	
		RSBN1	
		OR10AD1	
		CCDC51	
		FCF1	
		LURAP1L	
		BPIFA2	
		BPIFB3	

		BPIFB4	
		CARS1	
		KRT222	
		TNP2	
		TRIM4	
		TRIM40	
		TTPAL	
		LCA5L	
		ZNF512	
		CREBRF	
		EMSY	
		GRID2IP	
		CPTP	
		HEATR3	
		DCST2	
		MTERF4	
		OSGIN2	
		CFAP70	
		CFAP298	
		CDADC1	
		CCDC36	
		C1orf189	
		FAXDC2	
		BOLA2	
		ZMAT5	
		VWA7	
		WDR78	
		WHRN	
		PFN3	
		SLC45A1	
		SYS1	
		LINGO4	
		PLEKHN1	
		RASL11A	
		TMEM89	
		CNOT11	
		SEPTIN1	
		SEPTIN8	
		DUSP28	
		SAP25	
		MFSD4B	
		CCDC71	
		CCDC82	
		BPIFA3	
		ZNF774	
		NSG2	
		PCP4L1	
		CNOT9	

		DNLZ	
		CATIP	
		AQP12A	
		AQP12B	
		TSGA10IP	
		PRM3	
		DEXI	
		C17orf67	
		TMA7	
		ZNRD2	
		TMEM116	
		PRXL2A	
		LY6G5C	
		EIPR1	
		OR5B12	
		OR5B21	
		CFAP126	
		CCDC184	
		ABRAXAS2	
		AGAP5	
		FAM205A	
		C11orf21	
		LYRM9	
		ANKRD65	
		KRTAP5-5	
		ZUP1	
		PCNX3	
		LY6G6E	
		MUCL3	
		RUSC1-AS1	
		MFSD13A	
		BICRA	
		TRAPPC3L	
		KRTAP5-6	
		LCE3A	
		LCE3B	
		KHDC4	
		INKA1	
		EEF1AKMT2	
		OR5B2	
		C9orf163	
		IGF2-AS	
		INAFM1	
		STIMATE	
		ARIH2OS	
		TTLL8	
		TMEM250	
		C1QTNF12	

		C20orf203	
		TNXA	
		LINC02694	
		PI4KAP2	
		TSBP1	
		DELEC1	
		GABARAPL3	
		MEIKIN	
		C3orf84	
		CALHM4	
		MIR219A1	
		SCARNA12	
		ATP6V1G2-DDX39B	
		STIMATE-MUSTN1	
		HCG4	
		GVQW3	
		MSH5-SAPCD1	
		HOXA10-AS	
		SCARNA10	
		ZFP91-CNTF	
		LINC01620	
		TEX41	
		PPT2-EGFL8	
		JAZF1-AS1	
		JMJD1C-AS1	
		MIR3909	
		SCARNA11	
		MIR1301	
		MIR1260B	
		GBAP1	
		ACTA2-AS1	
		FLG-AS1	
		IL21-AS1	
		SNORD16	
		CD27-AS1	
		ADAM1A	
		FAM99B	
		THAP7-AS1	
		SLC26A4-AS1	
		SUGT1P3	
		MIR647	
		HCG17	
		SATB1-AS1	
		FAM205BP	
		C1RL-AS1	
		C20orf181	
		LINC00484	
		LINC00993	

		LINC01185	
		ZSWIM8-AS1	
		IFITM4P	
		SNORD124	
		RBFADN	
		IGLV5-52	
		MMP24OS	
		NALT1	
		CRTC3-AS1	
		MIR4686	
		MIR4425	
		HORMAD2-AS1	
		DKFZP434A062	
		MIR1208	
		CATIP-AS1	
		LURAP1L-AS1	
		FLJ31104	
		ARHGEF38-IT1	
		ASH1L-AS1	
		TNRC6C-AS1	
		WAKMAR2	
		ZBTB46-AS1	
		LINC00824	
		LINC01430	
		LINC01220	
		LINC01250	
		ENSG00000244255	
		PDCL3P4	
		OVOL1-AS1	
		TET2-AS1	
		MIR623	
		HCG21	
		MIR4673	
		LOC101928093	
		CDC37P1	
		ETS1-AS1	
		FAM238C	
		TNFRSF14-AS1	
		LINC00548	
		LINC00581	
		SMG1P5	
		LINC00892	
		LINC02800	
		LINC02098	
		MIR6090	
		NRAD1	
		LOC101927533	
		BSN-DT	

		MIR6513	
		MIR3936HG	
		CCR5AS	
		UBE2Q1-AS1	
		LINC02202	
		SNORA58B	
		IRS3P	
		SLC12A9-AS1	
		IL6R-AS1	
		ENSG00000249624	
		MIR6771	
		MIR4456	
		DDR1-DT	
		LOC101929574	
		MHENCR	
		RPL7P32	
		LOC101928120	
		CCND3P1	
		ETF1P1	
		ANKRD33B-AS1	
		LINC01845	
		LINC01989	
		LINC02555	
		LINC02570	
		ENSG00000263020	
		LINC01147	
		RANP1	
		ZBTB11-AS1	
		LINC02421	
		ENSG00000254295	
		ENSG00000236039	
		ENSG00000238160	
		ENSG00000238290	
		ENSG00000245156	
		IL1R1-AS1	
		SUMO2P1	
		ENSG00000253736	
		EIF2S2P3	
		ENSG00000229694	
		MICB-DT	
		RNA5SP192	
		RPS19P3	
		RNY4P10	
		RPL23AP12	
		RNU7-57P	
		RPS5P3	
		ENSG00000229990	
		LOC101928372	

		ABHD17AP4	
		ENSG00000258539	
		LINC02708	
		ENSG00000260302	
		ZNF90P1	
		LINC00604	
		ENSG00000253508	
		LINC02213	
		RNU6-344P	
		ENSG00000227938	
		SPTLC1P1	
		ENSG00000248753	
		ENSG00000241764	
		ENSG00000238280	
		ENSG00000234630	
		ENSG00000247121	
		ENSG00000248373	
		SNRPGP7	
		ENSG00000243696	
		ENSG00000253445	
		ENSG00000253111	
		ENSG00000234261	
		ENSG00000234290	
		ENSG00000234117	
		ENSG00000234132	
		ENSG00000254855	
		ENSG00000255038	
		ENSG00000254461	
		ENSG00000255135	
		ENSG00000255320	
		ENSG00000254810	
		HMGB3P4	
		HNRNPCP4	
		RNU1-134P	
		EBAG9P1	
		RN7SL391P	
		ENSG00000231128	
		ENSG00000231355	
		ENSG00000230534	
		ENSG00000230684	
		SERBP1P3	
		GLULP4	
		RNA5SP184	
		DPPA5P4	
		ENSG00000226681	
		NPM1P17	
		ENSG00000226812	
		ENSG00000225421	

		ENSG00000212228	
		RNU6-43P	
		RNU6-474P	
		RNU6-543P	
		RNU6-638P	
		ENSG00000199550	
		RNU6-70P	
		ENSG00000224228	
		ENSG00000224478	
		ENSG00000224934	
		ENSG00000225172	
		RPL21P33	
		RNU6-850P	
		RNU6-919P	
		ENSG00000204758	
		RPSAP64	
		ENSG00000228778	
		ENSG00000228863	
		ENSG00000228412	
		ENSG00000229299	
		CCT5P2	
		CCR12P	
		CBX3P9	
		ENSG00000284779	
		ENSG00000285551	
		BTF3P2	
		LOC112267968	
		ENSG00000255966	
		ENSG00000256967	
		KRT18P39	
		ENSG00000259005	
		ENSG00000258407	
		ENSG00000258559	
		ENSG00000258646	
		ENSG00000258740	
		ENSG00000258820	
		ENSG00000261338	
		LINC02354	
		LINC02723	
		ENSG00000260257	
		ENSG00000256433	
		TRG-TCC2-6	
		ENSG00000259202	
		TRL-CAG1-6	
		ENSG00000236471	
		ENSG00000235888	
		ENSG00000261573	
		ENSG00000270640	

		ENSG00000271855	
		ENSG00000271781	
		ENSG00000272305	
		ENSG00000262151	
		ENSG00000263080	
		ENSG00000263033	
		ENSG00000263766	
		ENSG00000261367	
		ENSG00000273154	
		ENSG00000266202	
		ENSG00000267199	
		ENSG00000267480	
		ENSG00000268746	
		ENSG00000268810	
		ENSG00000269514	
		ENSG00000269621	
		ENSG00000269919	
		ENSG00000270124	
		ENSG00000270210	
		CICP4	
		RPS21P8	
		RPS23P10	
		RNU6-320P	
		RNU6-351P	
		PEBP1P3	
		ENSG00000248734	
		ENSG00000238326	
		LOC105378083	
		ENSG00000249743	
		RN7SKP226	
		ENSG00000232124	
		RNU6-299P	
		RPLP0P7	
		ENSG00000184441	
		ENSG00000199332	
		ENSG00000199473	
		RPL5P26	
		ENSG00000219159	
		RNU6-925P	
		ENSG00000202533	
		ENSG00000205537	
		ENSG00000283782	
		ENSG00000284633	
		FGFR1OP2P1	
		ENSG00000283321	
		AIMP1P2	
		LINC01958	
		ENSG00000260651	

		ENSG00000260773	
		ENSG00000260577	
		ENSG00000260233	
		ENSG00000259314	
		ENSG00000235434	
		ENSG00000234929	
		ENSG00000272109	
		ENSG00000261644	
		ENSG00000263893	
		ENSG00000263756	
		ENSG00000267303	
		ENSG00000269570	
		ENSG00000270000	
		ENSG00000270212	
		LINC02863	
		lnc-ITGB1BP1-3	
		LOC101927745	
		ENSG00000251049	
		ENSG00000253955	
		ENSG00000237773	
		POLR2LP1	
		hsa-miR-5096-095	
		LOC105369302	
		ENSG00000247853	
		SNRPGP8	
		ENSG00000250993	
		ENSG00000251459	
		KCCAT333	
		HMGN2P18	
		lnc-GCA-5	
		RNU6-144P	
		EIF1P7	
		lnc-TMED10-5	
		lnc-TMEM50B-3	
		RN7SKP113	
		RN7SL51P	
		RN7SKP211	
		ENSG00000231557	
		ENSG00000230074	
		ENSG00000230537	
		RNU6-222P	
		NIPA2P5	
		LOC100129776	
		lnc-IL10-5	
		ENSG00000225931	
		ENSG00000226032	
		ENSG00000197254	
		ENSG00000197536	

		RNU6-704P	
		RPL35P9	
		ENSG00000219410	
		lnc-HOXA13-1	
		ENSG00000222701	
		ENSG00000200677	
		RNU7-15P	
		ENSG00000228037	
		ENSG00000228430	
		lnc-ARHGAP20-52	
		ENSG00000286503	
		lnc-ASIC2-2	
		lnc-PRDM1-1	
		ENSG00000281883	
		ENSG00000284829	
		ENSG00000285082	
		ENSG00000285413	
		ENSG00000285446	
		LOC390314	
		BTF3L4P3	
		ENSG00000287771	
		lnc-SCNN1A-1	
		lnc-SDF4-1	
		ENSG00000258860	
		LINC02300	
		YBX1P5	
		ENSG00000261025	
		KRT18P56	
		LOC107984360	
		PIGCP2	
		ENSG00000237371	
		ENSG00000237422	
		ENSG00000236318	
		lnc-MAP3K7-3	
		ENSG00000234789	
		ENSG00000272072	
		ENSG00000272459	
		ENSG00000262020	
		ENSG00000272791	
		ENSG00000273055	
		ENSG00000273176	
		ENSG00000266527	
		LINC02635	
		ENSG00000272477	
		lnc-TM9SF2-4	
		lnc-TM9SF2-7	
		piR-50893	
		lnc-USP36-4	

		lnc-ZCCHC24-7	
		lnc-IRF1-7	
		HSALNG0086605	
		lnc-LIPG-7	
		lnc-MRPL23-2	
		ENSG00000242044-001	
		lnc-MIEN1-1	
		lnc-MMEL1-1	
		JA662168	
		lnc-NDFIP1-1	
		piR-55650-032	
		lnc-NEK7-4	
		piR-37824	
		ENSG00000234062	
		lnc-NFKBIZ-2	
		lnc-NFKBIZ-3	
		ENSG00000253683	
		ENSG00000249650	
		lnc-MTPAP-7	
		piR-33458	
		lnc-CLEC16A-4	
		piR-42694-019	
		lnc-TH-1	
		HNRNPA1P41	
		lnc-TSPAN32-4	
		piR-34911-011	
		piR-43104-029	
		lnc-TRRAP-5	
		lnc-ZC3H12C-5	
		HSALNG0021688	
		lnc-DUSP1-7	
		lnc-TNFRSF14-3	
		lnc-TNFRSF1A-1	
		lnc-TNFSF18-3	
		piR-50444-308	
		lnc-TMED10-2	
		lnc-TMED10-4	
		lnc-TMEM268-4	
		lnc-TMEM17-10	
		piR-51327	
		lnc-FAM109A-1	
		HSALNG0001696	
		piR-51137-090	
		piR-43105-342	
		lnc-FAP-3	
		lnc-USP25-6	
		lnc-DDR1-4	
		CHCHD2P3	

		piR-50437-058	
		lnc-VEGFA-1	
		lnc-FCGR3A-2	
		lnc-FCGR3A-4	
		piR-52079-016	
		piR-52079-043	
		lnc-WASHC5-9	
		lnc-FLI1-5	
		HSALNG0017394	
		lnc-FNBP1-2	
		lnc-FOSL2-2	
		lnc-EMC8-1	
		lnc-UBAC2-4	
		piR-59241	
		ENSG00000206734	
		LOC100507103	
		lnc-IL15RA-4	
		LOC100533842	
		piR-44878-042	
		ENSG00000213386	
		NONHSAG001750.2	
		NONHSAG003874.2	
		NONHSAG041785.2	
		piR-53431-298	
		piR-45035-151	
		ENSG00000224431	
		ENSG00000224988	
		lnc-HLA-A-2	
		lnc-HOXA11-1	
		lnc-HOXA11-3	
		lnc-HOXA13-3	
		NONHSAG007397.2	
		HSALNG0046732	
		lnc-GRB7-1	
		lnc-GSDMC-13	
		lnc-IL6ST-2	
		NXPE2P1	
		RF00483	
		lnc-CTNND2-10	
		lnc-CREB5-4	
		lnc-CNTF-2	
		lnc-ATP6V1G3-6	
		lnc-REL-2	
		RF00017-5119	
		ENSG00000286116	
		lnc-ATG5-7	
		RF00017-5399	
		RF00017-5414	

		AC003959	
		lnc-ANKMY1-3	
		ENSG00000283648	
		lnc-PSORS1C2-1	
		lnc-PTGIR-1	
		lnc-PTGIR-2	
		RF00017-4587	
		lnc-APOBR-1	
		RF00017-4625	
		RF00017-4964	
		ENSG00000285837	
		lnc-PWP2-2	
		lnc-ARFRP1-1	
		lnc-CALM3-3	
		ENSG00000287850	
		lnc-SEH1L-5	
		AY077737	
		lnc-CCT4-2	
		lnc-CEP76-3	
		lnc-SLC34A1-3	
		lnc-SNX13-5	
		RF00017-5695	
		lnc-RNF39-8	
		piR-48877	
		RF00017-6553	
		lnc-SBNO2-2	
		lnc-RTN4IP1-6	
		lnc-RTTN-6	
		lnc-C1QTNF1-9	
		ENSG00000286974	
		RF00017-3549	
		lnc-AHR-4	
		ENSG00000283265	
		ENSG00000283286	
		ENSG00000283360	
		ENSG00000283573	
		piR-48950-118	
		RF00017-6464	
		piR-57133-098	
		ENSG00000287597	
		RF00017-6488	
		lnc-SCGB2B2-133	
		lnc-SCGB2B2-4	
		lnc-SCNN1A-2	
		RF00017-717	
		lnc-C9orf78-2	
		piR-31937-163	
		ENSG00000283579	

		RF00017-5418	
		piR-48759-287	
		RF00017-3286	
		RF00017-349	
		AB372574	
		lnc-OLIG3-1	
		lnc-NXPE3-2	
		RF00017-1621	
		RF00017-1272	
		L13712-019	
		ENSG00000275693	
		ENSG00000276609	
		ENSG00000278708	
		RF00017-2113	
		ENSG00000260249	
		RF00017-2930	
		RF00017-2425	
		RF00017-2721	
		ENSG00000236710	
		ENSG00000271267	
		ENSG00000271992	
		lnc-PLEKHG6-4	
		ENSG00000272644	
		ENSG00000273466	
		ENSG00000274038	
		ENSG00000274737	
		ENSG00000268069	
		piR-58538-001	
		piR-58538-016	
		lnc-TRIB1-1	
		piR-43107-300	
		HSALNG0007877	
		lnc-WNT4-6	
		piR-52079-091	
		lnc-IQCH-5	
		lnc-ZNF365-3	
		HSALNG0088853	
		ENSG00000227836	
		lnc-ITGB1BP1-2	
		HSALNG0088095	
		HSALNG0130695	
		piR-30396	
		piR-61945-308	
		lnc-LYRM9-3	
		ENSG00000237553	
		piR-46391-002	
		hsa-miR-5095-436	
		ENSG00000242299	

		ENSG00000240023	
		ENSG00000238138	
		piR-55186-001	
		LOC105373724	
		ENSG00000244061	
		piR-38319	
		lnc-MMEL1-2	
		piR-38259	
		piR-55281-150	
		piR-46501-010	
		piR-45932-058	
		piR-46002-152	
		lnc-NBN-9	
		piR-47234	
		RF00001-253	
		ENSG00000254926	
		ENSG00000254755	
		RF00001-209	
		HG983680	
		lnc-TRAPPC3L-2	
		piR-52079-106	
		lnc-ZMAT5-2	
		piR-52294-068	
		piR-43408-221	
		lnc-DYRK2-15	
		piR-43099-059	
		RF00994-809	
		RF00994-753	
		lnc-TMEM258-1	
		lnc-DSE-1	
		lnc-DNLZ-1	
		piR-51267-027	
		piR-51449	
		HQ292134	
		piR-51137-089	
		lnc-TTC33-6	
		lnc-USP20-5	
		LOC102723798	
		lnc-LAMB4-3	
		piR-33804-078	
		lnc-CYTL1-3	
		piR-50437-360	
		piR-58297-114	
		piR-50346	
		lnc-WASF3-4	
		HSALNG0015265	
		piR-43325-002	
		piR-59907-003	

		HSALNG0017398	
		piR-61240-151	
		piR-44610-008	
		piR-44610-011	
		NONHSAG017238.2	
		lnc-IKZF1-5	
		lnc-IL12B-2	
		lnc-IL19-2	
		lnc-HDAC11-4	
		ENSG00000213440	
		NONHSAG026080.2	
		piR-43939-002	
		lnc-GOT1-1	
		NONHSAG043568.2	
		HSALNG0073924	
		NONHSAG045774.2	
		lnc-ICAM3-1	
		piR-61101-576	
		LOC100289118	
		lnc-HLA-DQA1-9	
		lnc-HLA-DRB1-7	
		NONHSAG031883.2-001	
		NONHSAG031883.2-002	
		LOC100419513	
		lnc-HNF4A-1	
		ENSG00000220412	
		piR-45012-401	
		NONHSAG008489.2	
		HSALNG0043662	
		piR-36455	
		piR-52916-031	
		HSALNG0050236	
		ENSG00000227758	
		piR-37170-040	
		piR-61514-156	
		lnc-CTIF-9	
		RF00066-120	
		piR-50308-096	
		lnc-ARHGAP20-11	
		ENSG00000285616	
		lnc-R3HDML-1	
		piR-56480-015	
		ENSG00000286368	
		lnc-ATP6V1G3-5	
		piR-32461-022	
		RF00017-5114	
		ENSG00000286186	
		RF00017-3998	

		lnc-PRKCD-2	
		lnc-AMZ1-1	
		piR-39701-054	
		lnc-PSMA6-6	
		RF00017-4375	
		piR-56341-125	
		piR-48325-110	
		piR-48325-111	
		lnc-PTCD2-5	
		piR-56341-188	
		lnc-PTGER4-8	
		RF00017-458	
		RF00017-4588	
		RF00017-4629	
		RF00017-4970	
		piR-32285-085	
		RF00017-4711	
		ENSG00000285552	
		ENSG00000285560	
		lnc-CCDC8-6	
		lnc-SFMBT1-5	
		lnc-CAB39L-9	
		lnc-CDH3-5	
		piR-49732-033	
		lnc-SLC37A1-2	
		piR-41306-110	
		lnc-CEP76-2	
		piR-33432-055	
		RF00026-1103	
		ENSG00000286629	
		RF00017-5677	
		piR-48759-204	
		piR-56902	
		piR-48852	
		lnc-BOLL-6	
		ENSG00000288064	
		piR-57133-396	
		ENSG00000287967	
		lnc-ADCY7-3	
		RF00017-3409	
		lnc-RIPK2-3	
		RF00017-356	
		RF00017-3596	
		ENSG00000280010	
		RF00017-3761	
		RF00017-3762	
		piR-48259	
		ENSG00000283504	

		lnc-ANKRD33B-5	
		lnc-ANKRD33B-6	
		lnc-RPL21-6	
		piR-31937-161	
		RF00017-3132	
		5EW4_A-043	
		RF00017-3201	
		piR-48820-008	
		RF00017-7988	
		piR-41306-095	
		lnc-STMN3-5	
		piR-55655-375	
		piR-47211-583	
		piR-39098-183	
		piR-55948-040	
		piR-39099-151	
		piR-55783	
		RF00017-1397	
		piR-48007	
		piR-55829-003	
		piR-31292-113	
		RF00017-092	
		RF00017-299	
		lnc-PDGFB-3	
		ENSG00000235679	
		LOC105371080	
		ZYXP1	
		ENSG00000262488	
		ENSG00000272779	
		ENSG00000272980	
		ENSG00000273112	
		piR-31937-039	
		ENSG00000275103	
		ENSG00000270204	
		HSALNG0133311	
		piR-58538-006	
		piR-58538-007	
		piR-58538-008	
		piR-58538-009	
		piR-58538-010	
		piR-58538-011	
		piR-58538-012	
		piR-58538-013	
		piR-58538-014	
		piR-58538-015	
		piR-60146-087	
		LOC101927897	
		piR-38580-144	

		LOC105377139	
		LOC105372877	
		LOC105373831	
		LOC105369818	
		ENSG00000253654	
		ENSG00000250839	
		LOC646347	
		piR-43105-054	
		HSALNG0032571	
		RF00994-819	
		HSALNG0001583	
		LOC102724748	
		ENSG00000230785	
		LOC100509370	
		ENSG00000213080	
		piR-36393-409	
		HSALNG0041356	
		lnc-ICAM3-2	
		LOC100420500	
		ENSG00000229172	
		piR-56451-093	
		piR-48749-023	
		piR-56759-041	
		piR-40110-155	
		ENSG00000285810	
		piR-33212	
		piR-40398	
		ENSG00000279625	
		ENSG00000286565	
		piR-39767-002	
		piR-55655-210	
		RF00017-239	
		RF00017-2046	
		ENSG00000270335	
		ENSG00000253843	
		ENSG00000244245	
		LOC105375444	
		lnc-TNFSF15-5	
		ENSG00000233020	
		ENSG00000224104	
		piR-32810-138	
		ENSG00000287290	
		ENSG00000286119	
		ENSG00000276949	
		ENSG00000277191	
		GKN1	
		VASP	
		SOCS2	

		TFPI2	
		FMR1	
		CLCN5	
		VCAN	
		INPP5D	
		TAB1	
		FADS1	
		PNPLA3	
		HIC1	
		IPMK	
		MGAT3	
		PRICKLE2	
		ASAH1	
		CALB2	
		SATB1	
		EPAS1	
		ALDH9A1	
		GALK1	
		HMGA2	
		TNFSF13	
		REG1B	
		SOAT1	
		ALPK1	
		ABCB7	
		CD99	
		SELENOS	
		ZNF281	
		HCK	
		CACNA1C	
		PGM1	
		RPS6KA2	
		VRK1	
		EIF2AK2	
		POU2F1	
		CSF2RA	
		ATP2B2	
		IRS2	
		SPRY4	
		CTSZ	
		SPHK2	
		PLTP	
		RASGRF1	
		CLN3	
		RNASET2	
		LAMA5	
		NLRP2	
		IL31RA	
		SPRED1	

		PADI2	
		CDC37	
		SULF2	
		ST8SIA4	
		CREB5	
		UBQLN4	
		NOX3	
		SPRED2	
		RAB13	
		FLRT1	
		EPS8L2	
		POP4	
		RBFOX1	
		DBP	
		CEBPG	
		ZPBP	
		SYT4	
		STXBP4	
		MMP28	
		RSPO3	
		TMTC2	
		MPPED2	
		CSMD2	
		TM9SF4	
		TUBD1	
		PF4V1	
		SLC10A4	
		SNX7	
		RABEP2	
		GALNTL6	
		IZUMO1	
		SLAIN2	
		OR2AT4	
		KIAA1841	
		TRPT1	
		PHACTR2	
		ZNF532	
		PRRC1	
		MORC4	
		BABAM2	
		LRRC61	
		TM6SF2	
		C1orf53	
		RIMBP3	
		C2orf74	
		BORCS5	
		PPAN-P2RY11	
		ZNF300P1	

		MIR6727	
		OR2AT1P	
		OR7E116P	
		RPL21P108	
		RPL18P7	
		RPL35AP7	
		RNU7-67P	
		LAMP3	
		NTRK3	
		BMPR2	
		CCNE1	
		ACO1	
		LTC4S	
		TRIM5	
		SLCO4A1	
		PDLIM1	
		TRAIP	
		UBASH3B	
		ABCF2	
		NCR1	
		IL31	
		C13orf42	
		TRIM33	
		IQGAP1	
		SSTR2	
		AMPD2	
		AMPD3	
		TAOK1	
		CASP6	
		CDH11	
		GRN	
		SLC7A5	
		USP8	
		SPINT2	
		RNF31	
		SHARPIN	
		IL17RE	
		CD101	
		MYLK	
		PRLR	
		PGK1	
		CPT1A	
		NR1H3	
		CES1	
		AKR1C2	
		ADAM9	
		ARHGDIA	
		GRM8	

		MTNR1B	
		CBR1	
		FPR2	
		ANGPTL4	
		ACADVL	
		ITGB6	
		MGLL	
		TLE1	
		CYP26B1	
		HES1	
		ANXA4	
		PON2	
		PANX1	
		MTNR1A	
		GGH	
		GALNT2	
		AKR1C1	
		TPP1	
		JUNB	
		GSTA1	
		HNF4G	
		ADAM19	
		SULT1B1	
		HSPB2	
		GPC5	
		TAGLN2	
		GSTK1	
		GSTA2	
		CLDN11	
		NCAPD3	
		ALDOC	
		FLOT2	
		FN3K	
		USP25	
		PPP2R5E	
		PPP3R2	
		PSG1	
		HNRNPH3	
		RNF20	
		FIS1	
		CALCOCO2	
		USO1	
		NOXO1	
		IBSP	
		PANX2	
		LSAMP	
		CLEC4A	
		EXOSC1	

		PIH1D1	
		PTER	
		CLEC2D	
		CNN3	
		BEST2	
		MAGI3	
		DNAH12	
		DNPH1	
		ZNF649	
		ACTL8	
		BEST4	
		ZG16	
		PROSER1	
		C10orf67	
		H2BC3	
		MIR595	
		MIR1246	
		LOC400867	
		NM	
		IL6STP1	
		OMP	
		ABCC4	
		MIR425	
		MYH9	
		ACTB	
		TTF2	
		STK38	
		CHKB	
		SREBF2	
		TRAF4	
		CD47	
		NAA15	
		PIK3CD	
		LIMK2	
		KMT2A	
		ACTR2	
		CHD3	
		CD177	
		YBX2	
		TARS1	
		GRAP2	
		P2RY6	
		PPM1D	
		TSPO	
		TRAF5	
		NDRG1	
		PROK2	
		LPO	

		AQP3	
		MAP3K1	
		PON3	
		KLRB1	
		ICA1	
		OLFM4	
		OTUD1	
		PNOC	
		KAT2B	
		LTB4R	
		MIP	
		AQP10	
		ARNT	
		NTF4	
		DVL2	
		CCL28	
		ITGAV	
		TPM3	
		MPST	
		FUT8	
		MNAT1	
		CCDC22	
		HDAC4	
		GFRA1	
		GUCY2D	
		MDM4	
		MIR22HG	
		PI4KB	
		EGLN3	
		EIF2B4	
		CLSPN	
		ATF1	
		MCM3	
		FSTL1	
		FUT7	
		UBD	
		DYRK1A	
		AXL	
		PLD1	
		RYR2	
		WEE1	
		P2RY2	
		GRM3	
		CYP11B1	
		CHRNA7	
		TNFRSF11A	
		ITGB5	
		SLC29A2	

		DLG1	
		RPSA	
		B4GALT1	
		CNTN1	
		PTGFR	
		KL	
		MAP3K14	
		CTRC	
		CHD2	
		DOCK2	
		AKR1B10	
		CACNA1I	
		FABP1	
		VAV2	
		VPS35	
		UBE2D3	
		INHBA	
		SLCO2B1	
		ENPEP	
		KCNN3	
		CYFIP2	
		COPS5	
		VAPA	
		UBB	
		SLC8A3	
		IGF2BP3	
		ITGA11	
		SLC28A2	
		SEPHS1	
		DPP6	
		ROBO2	
		CDH13	
		CACNB1	
		FABP5	
		TIRAP	
		TSFM	
		SLIT1	
		NRG3	
		SORBS1	
		CNN1	
		CPD	
		CADPS	
		SMYD2	
		NSMAF	
		PTPRR	
		CAPZB	
		HNRNPM	
		ELMO1	

		SBF1	
		GPM6A	
		CD200R1	
		MEGF10	
		ABCB9	
		LRRC4C	
		CAPZA1	
		ARID5B	
		CALU	
		TNFRSF19	
		VPS26A	
		SMARCD3	
		PDSS2	
		PIK3AP1	
		PARVB	
		HCST	
		GPR158	
		DNER	
		CDK18	
		ALDH1L2	
		LRP1B	
		ARHGAP24	
		EPB41L2	
		AUTS2	
		TIGAR	
		SNRPC	
		PLCH1	
		SOX13	
		TAF3	
		STAB2	
		ELL2	
		NCR2	
		CRISP3	
		COL19A1	
		MRPL28	
		MTSS1	
		MEF2B	
		ABI3	
		BRWD1	
		LRRTM4	
		FMNL2	
		LY75	
		USH2A	
		SMYD1	
		KCNIP4	
		POU6F2	
		RALGAPA2	
		CCDC40	

		ANO2	
		CDH22	
		HUNK	
		JAKMIP1	
		SYTL1	
		TMEM132D	
		CSMD1	
		MPRIP	
		HOXD3	
		SCUBE1	
		CCNG2	
		MDGA2	
		TRIM6	
		GDE1	
		RAB3C	
		TBC1D14	
		SYT9	
		MON2	
		CPNE8	
		DERL3	
		DNAH10	
		RTCB	
		SGCZ	
		DNASE2B	
		EYS	
		FCGBP	
		AVIL	
		UBE2U	
		VPS13C	
		SLC38A10	
		RBM26	
		LINGO2	
		CNTNAP5	
		PLD5	
		CEP131	
		SAMD14	
		CCDC93	
		FRY	
		ADAMDEC1	
		ARMC3	
		UMODL1	
		JPH4	
		SHISA6	
		ZNF804B	
		EFCAB6	
		SNTG1	
		TMEM182	
		KMT5B	

		CEP170B	
		CEACAM4	
		BEND7	
		ZNF385D	
		RELL1	
		TMEM255B	
		EMC9	
		TTLL2	
		TOPAZ1	
		TENT2	
		KIAA1257	
		WASHC2C	
		TRIM6-TRIM34	
		REC114	
		NPSR1-AS1	
		LINC00469	
		CLRN1-AS1	
		MIR19B2	
		SNORD48	
		ZNF815P	
		PRICKLE2-AS3	
		LINC00331	
		LACTB2-AS1	
		ANKRD34C-AS1	
		LINC01342	
		LOC101929710	
		FILNC1	
		LINC02367	
		IATPR	
		LINC00836	
		ISCA2P1	
		LOC102724404	
		LOC100506258	
		LRRTM4-AS1	
		PPIAP33	
		IDDM15	
		LOC101927484	

Table S3: GGQLD-compound-target-UC Network.

Drug	Mol ID	Molecule name	Target
huanglian	MOL000098	quercetin	ABCG2
gancao	MOL000098	quercetin	ABCG2
huangqin	MOL000449	Stigmasterol	ADRA2A
huangqin	MOL002714	baicalein	AHR
huanglian	MOL000098	quercetin	AHR
gancao	MOL000422	kaempferol	AHR
gancao	MOL000098	quercetin	AHR
huanglian	MOL000098	quercetin	ALOX5
gancao	MOL000422	kaempferol	ALOX5
gancao	MOL000098	quercetin	ALOX5
gegen	MOL000358	beta-sitosterol	BAX
huangqin	MOL001689	acacetin	BAX
huangqin	MOL000173	wogonin	BAX
huangqin	MOL002714	baicalein	BAX
huangqin	MOL000358	beta-sitosterol	BAX
huanglian	MOL000098	quercetin	BAX
gancao	MOL000422	kaempferol	BAX
gancao	MOL000098	quercetin	BAX
gegen	MOL000358	beta-sitosterol	BCL2
huangqin	MOL001689	acacetin	BCL2
huangqin	MOL000173	wogonin	BCL2
huangqin	MOL002714	baicalein	BCL2
huangqin	MOL002928	oroxylin a	BCL2
huangqin	MOL000358	beta-sitosterol	BCL2
huanglian	MOL000098	quercetin	BCL2
gancao	MOL000422	kaempferol	BCL2
gancao	MOL004328	naringenin	BCL2
gancao	MOL000497	licochalcone a	BCL2
gancao	MOL000098	quercetin	BCL2
huangqin	MOL012245	5,7,4'-trihydroxy-6-methoxyflavanone	CA2
huangqin	MOL012245	5,7,4'-trihydroxy-6-methoxyflavanone	CA2
huangqin	MOL012266	rivularin	CA2
gancao	MOL004815	(E)-1-(2,4-dihydroxyphenyl)-3-(2,2-dimethylchromen-6-yl)prop-2-en-1-one	CA2
gancao	MOL004835	Glypallichalcone	CA2
gancao	MOL004841	Licochalcone B	CA2
gancao	MOL000497	licochalcone a	CA2
gegen	MOL000358	beta-sitosterol	CASP3
huangqin	MOL001689	acacetin	CASP3
huangqin	MOL000173	wogonin	CASP3
huangqin	MOL002714	baicalein	CASP3
huangqin	MOL002928	oroxylin a	CASP3
huangqin	MOL000358	beta-sitosterol	CASP3
huanglian	MOL000098	quercetin	CASP3

gancao	MOL000422	kaempferol	CASP3
gancao	MOL004328	naringenin	CASP3
gancao	MOL000098	quercetin	CASP3
gancao	MOL004328	naringenin	CAT
huangqin	MOL000173	wogonin	CCL2
huanglian	MOL000098	quercetin	CCL2
gancao	MOL000098	quercetin	CCL2
huangqin	MOL000173	wogonin	CCND1
huanglian	MOL000098	quercetin	CCND1
gancao	MOL000497	licochalcone a	CCND1
gancao	MOL000098	quercetin	CCND1
huanglian	MOL000098	quercetin	CD40LG
gancao	MOL000098	quercetin	CD40LG
huangqin	MOL001689	acacetin	CDKN1A
huangqin	MOL000173	wogonin	CDKN1A
huanglian	MOL000098	quercetin	CDKN1A
gancao	MOL000098	quercetin	CDKN1A
gegen	MOL000392	formononetin	CHEK1
gegen	MOL002959	3'-Methoxydaidzein	CHEK1
huangqin	MOL001689	acacetin	CHEK1
huangqin	MOL000173	wogonin	CHEK1
huangqin	MOL002917	5,2',6'-Trihydroxy-7,8-dimethoxyflavone	CHEK1
huangqin	MOL002932	Panicolin	CHEK1
huangqin	MOL002933	5,7,4'-Trihydroxy-8-methoxyflavone	CHEK1
huangqin	MOL002934	NEOBAICALEIN	CHEK1
huangqin	MOL000525	Norwogonin	CHEK1
huangqin	MOL008206	Moslosooflavone	CHEK1
huanglian	MOL002668	Worenine	CHEK1
gancao	MOL002311	Glycyrol	CHEK1
gancao	MOL000239	Jaranol	CHEK1
gancao	MOL000354	isorhamnetin	CHEK1
gancao	MOL003656	Lupiwighteone	CHEK1
gancao	MOL003896	7-Methoxy-2-methyl isoflavone	CHEK1
gancao	MOL000392	formononetin	CHEK1
gancao	MOL000417	Calycosin	CHEK1
gancao	MOL004811	Glyasperin C	CHEK1
gancao	MOL004814	Isotrifoliol	CHEK1
gancao	MOL004815	(E)-1-(2,4-dihydroxyphenyl)-3-(2,2-dimethylchromen-6-yl)prop-2-en-1-one	CHEK1
gancao	MOL004820	kanzonols W	CHEK1
gancao	MOL004824	(2S)-6-(2,4-dihydroxyphenyl)-2-(2-hydroxypropan-2-yl)-4-methoxy-2,3-dihydrofuro[3,2-g]chromen-7-one	CHEK1
gancao	MOL004827	Semilicoisoflavone B	CHEK1
gancao	MOL004828	Glepidotin A	CHEK1

gancao	MOL004833	Phaseolinisoflavan	CHEK1
gancao	MOL004835	Glypallichalcone	CHEK1
gancao	MOL004841	Licochalcone B	CHEK1
gancao	MOL004849	3-(2,4-dihydroxyphenyl)-8-(1,1-dimethylprop-2-enyl)-7-hydroxy-5-methoxy-coumarin	CHEK1
gancao	MOL004855	Licoricone	CHEK1
gancao	MOL004856	Gancaonin A	CHEK1
gancao	MOL004857	Gancaonin B	CHEK1
gancao	MOL004863	3-(3,4-dihydroxyphenyl)-5,7-dihydroxy-8-(3-methylbut-2-enyl)chromone	CHEK1
gancao	MOL004864	5,7-dihydroxy-3-(4-methoxyphenyl)-8-(3-methylbut-2-enyl)chromone	CHEK1
gancao	MOL004866	2-(3,4-dihydroxyphenyl)-5,7-dihydroxy-6-(3-methylbut-2-enyl)chromone	CHEK1
gancao	MOL004879	Glycyrin	CHEK1
gancao	MOL004883	Licoisoflavone	CHEK1
gancao	MOL004884	Licoisoflavone B	CHEK1
gancao	MOL004907	Glyzaglabrin	CHEK1
gancao	MOL004908	Glabridin	CHEK1
gancao	MOL004912	Glabrone	CHEK1
gancao	MOL004913	1,3-dihydroxy-9-methoxy-6-benzofurano[3,2-c]chromenone	CHEK1
gancao	MOL004914	1,3-dihydroxy-8,9-dimethoxy-6-benzofurano[3,2-c]chromenone	CHEK1
gancao	MOL004915	Eurycarpin A	CHEK1
gancao	MOL004957	HMO	CHEK1
gancao	MOL004966	3'-Hydroxy-4'-O-Methylglabridin	CHEK1
gancao	MOL000497	licochalcone a	CHEK1
gancao	MOL004974	3'-Methoxyglabridin	CHEK1
gancao	MOL004978	2-[(3R)-8,8-dimethyl-3,4-dihydro-2H-pyrano[6,5-f]chromen-3-yl]-5-methoxyphenol	CHEK1
gancao	MOL004990	7,2',4'-trihydroxy − 5-methoxy-3 − arylcoumarin	CHEK1
gancao	MOL004991	7-Acetoxy-2-methylisoflavone	CHEK1
gancao	MOL000500	Vestitol	CHEK1
gancao	MOL005000	Gancaonin G	CHEK1
gancao	MOL005012	Licoagroisoflavone	CHEK1
gancao	MOL005016	Odoratin	CHEK1
gancao	MOL005017	Phaseol	CHEK1
gancao	MOL005020	dehydroglyasperins C	CHEK1
huanglian	MOL000098	quercetin	CHUK
gancao	MOL000098	quercetin	CHUK
huanglian	MOL000098	quercetin	CLDN4
gancao	MOL000098	quercetin	CLDN4
huanglian	MOL000098	quercetin	CRP
gancao	MOL000098	quercetin	CRP

huanglian	MOL000098	quercetin	CXCL10
gancao	MOL000098	quercetin	CXCL10
huangqin	MOL000173	wogonin	CXCL8
huanglian	MOL000098	quercetin	CXCL8
gancao	MOL000098	quercetin	CXCL8
huanglian	MOL000098	quercetin	CYP1A1
gancao	MOL000422	kaempferol	CYP1A1
gancao	MOL000098	quercetin	CYP1A1
huanglian	MOL000098	quercetin	CYP1B1
gancao	MOL000422	kaempferol	CYP1B1
gancao	MOL000098	quercetin	CYP1B1
huangqin	MOL002928	oroxylin a	CYP2C9
huanglian	MOL000098	quercetin	CYP3A4
gancao	MOL000422	kaempferol	CYP3A4
gancao	MOL000098	quercetin	CYP3A4
huanglian	MOL000098	quercetin	DUOX2
gancao	MOL000098	quercetin	DUOX2
huanglian	MOL000098	quercetin	EGF
gancao	MOL000098	quercetin	EGF
huanglian	MOL000098	quercetin	EGFR
gancao	MOL000098	quercetin	EGFR
gegen	MOL000392	formononetin	ESR1
gegen	MOL002959	3'-Methoxydaidzein	ESR1
huangqin	MOL000173	wogonin	ESR1
huangqin	MOL000228	(2R)-7-hydroxy-5-methoxy-2-phenylchroman-4-one	ESR1
huangqin	MOL002933	5,7,4'-Trihydroxy-8-methoxyflavone	ESR1
huangqin	MOL002934	NEOBAICALEIN	ESR1
huangqin	MOL000073	ent-Epicatechin	ESR1
huangqin	MOL001458	coptisine	ESR1
huangqin	MOL002897	epiberberine	ESR1
huanglian	MOL001454	berberine	ESR1
huanglian	MOL002894	berberrubine	ESR1
huanglian	MOL001458	coptisine	ESR1
huanglian	MOL002897	epiberberine	ESR1
huanglian	MOL000785	palmatine	ESR1
huanglian	MOL002668	Worenine	ESR1
gancao	MOL001792	DFV	ESR1
gancao	MOL002311	Glycyrol	ESR1
gancao	MOL002565	Medicarpin	ESR1
gancao	MOL000354	isorhamnetin	ESR1
gancao	MOL003656	Lupiwighteone	ESR1
gancao	MOL003896	7-Methoxy-2-methyl isoflavone	ESR1
gancao	MOL000392	formononetin	ESR1
gancao	MOL000417	Calycosin	ESR1
gancao	MOL004328	naringenin	ESR1

gancao	MOL004805	(2S)-2-[4-hydroxy-3-(3-methylbut-2-enyl)phenyl]-8,8-dimethyl-2,3-dihydropyrano[2,3-f]chromen-4-one	ESR1
gancao	MOL004806	euchrenone	ESR1
gancao	MOL004808	glyasperin B	ESR1
gancao	MOL004810	glyasperin F	ESR1
gancao	MOL004811	Glyasperin C	ESR1
gancao	MOL004814	Isotrifoliol	ESR1
gancao	MOL004815	(E)-1-(2,4-dihydroxyphenyl)-3-(2,2-dimethylchromen-6-yl)prop-2-en-1-one	ESR1
gancao	MOL004820	kanzonols W	ESR1
gancao	MOL004824	(2S)-6-(2,4-dihydroxyphenyl)-2-(2-hydroxypropan-2-yl)-4-methoxy-2,3-dihydrofuro[3,2-g]chromen-7-one	ESR1
gancao	MOL004827	Semilicoisoflavone B	ESR1
gancao	MOL004828	Glepidotin A	ESR1
gancao	MOL004829	Glepidotin B	ESR1
gancao	MOL004833	Phaseolinisoflavan	ESR1
gancao	MOL004835	Glypallichalcone	ESR1
gancao	MOL004838	8-(6-hydroxy-2-benzofuranyl)-2,2-dimethyl-5-chromenol	ESR1
gancao	MOL004841	Licochalcone B	ESR1
gancao	MOL004848	licochalcone G	ESR1
gancao	MOL004849	3-(2,4-dihydroxyphenyl)-8-(1,1-dimethylprop-2-enyl)-7-hydroxy-5-methoxy-coumarin	ESR1
gancao	MOL004855	Licoricone	ESR1
gancao	MOL004856	Gancaonin A	ESR1
gancao	MOL004857	Gancaonin B	ESR1
gancao	MOL004863	3-(3,4-dihydroxyphenyl)-5,7-dihydroxy-8-(3-methylbut-2-enyl)chromone	ESR1
gancao	MOL004864	5,7-dihydroxy-3-(4-methoxyphenyl)-8-(3-methylbut-2-enyl)chromone	ESR1
gancao	MOL004879	Glycyrin	ESR1
gancao	MOL004882	Licocoumarone	ESR1
gancao	MOL004883	Licoisoflavone	ESR1
gancao	MOL004884	Licoisoflavone B	ESR1
gancao	MOL004885	licoisoflavanone	ESR1
gancao	MOL004891	shinpterocarpin	ESR1
gancao	MOL004898	(E)-3-[3,4-dihydroxy-5-(3-methylbut-2-enyl)phenyl]-1-(2,4-dihydroxyphenyl)prop-2-en-1-one	ESR1
gancao	MOL004904	licopyranocoumarin	ESR1
gancao	MOL004907	Glyzaglabrin	ESR1
gancao	MOL004908	Glabridin	ESR1
gancao	MOL004910	Glabranin	ESR1
gancao	MOL004911	Glabrene	ESR1

gancao	MOL004912	Glabrone	ESR1
gancao	MOL004913	1,3-dihydroxy-9-methoxy-6-benzofurano[3,2-c]chromenone	ESR1
gancao	MOL004914	1,3-dihydroxy-8,9-dimethoxy-6-benzofurano[3,2-c]chromenone	ESR1
gancao	MOL004915	Eurycarpin A	ESR1
gancao	MOL004935	Sigmoidin-B	ESR1
gancao	MOL004941	(2R)-7-hydroxy-2-(4-hydroxyphenyl)chroman-4-one	ESR1
gancao	MOL004945	(2S)-7-hydroxy-2-(4-hydroxyphenyl)-8-(3-methylbut-2-enyl)chroman-4-one	ESR1
gancao	MOL004948	Isoglycyrol	ESR1
gancao	MOL004949	Isolicoflavonol	ESR1
gancao	MOL004957	HMO	ESR1
gancao	MOL004959	1-Methoxyphaseollidin	ESR1
gancao	MOL004961	Quercetin der.	ESR1
gancao	MOL004966	3'-Hydroxy-4'-O-Methylglabridin	ESR1
gancao	MOL000497	licochalcone a	ESR1
gancao	MOL004974	3'-Methoxyglabridin	ESR1
gancao	MOL004978	2-[(3R)-8,8-dimethyl-3,4-dihydro-2H-pyrano[6,5-f]chromen-3-yl]-5-methoxyphenol	ESR1
gancao	MOL004980	Inflacoumarin A	ESR1
gancao	MOL004988	Kanzonol F	ESR1
gancao	MOL004989	6-prenylated eriodictyol	ESR1
gancao	MOL004990	7,2',4'-trihydroxy − 5-methoxy-3 − arylcoumarin	ESR1
gancao	MOL004991	7-Acetoxy-2-methylisoflavone	ESR1
gancao	MOL004993	8-prenylated eriodictyol	ESR1
gancao	MOL000500	Vestitol	ESR1
gancao	MOL005000	Gancaonin G	ESR1
gancao	MOL005001	Gancaonin H	ESR1
gancao	MOL005003	Licoagrocarpin	ESR1
gancao	MOL005007	Glyasperins M	ESR1
gancao	MOL005008	Glycyrrhiza flavonol A	ESR1
gancao	MOL005012	Licoagroisoflavone	ESR1
gancao	MOL005016	Odoratin	ESR1
gancao	MOL005017	Phaseol	ESR1
gancao	MOL005018	Xambioona	ESR1
gancao	MOL005020	dehydroglyasperins C	ESR1
huangqin	MOL001689	acacetin	FASN
gancao	MOL004328	naringenin	FASN
huangqin	MOL002714	baicalein	FOS
huanglian	MOL000098	quercetin	FOS
gancao	MOL000098	quercetin	FOS
huanglian	MOL000098	quercetin	GSTM1
gancao	MOL000422	kaempferol	GSTM1
gancao	MOL000098	quercetin	GSTM1

huanglian	MOL000098	quercetin	GSTP1
gancao	MOL000422	kaempferol	GSTP1
gancao	MOL004328	naringenin	GSTP1
gancao	MOL000098	quercetin	GSTP1
huanglian	MOL000098	quercetin	HMOX1
gancao	MOL000422	kaempferol	HMOX1
gancao	MOL000098	quercetin	HMOX1
huanglian	MOL002903	(R)-Canadine	HTR3A
gancao	MOL001484	Inermine	HTR3A
gancao	MOL004891	shinpterocarpin	HTR3A
huanglian	MOL000098	quercetin	ICAM1
gancao	MOL000422	kaempferol	ICAM1
gancao	MOL000098	quercetin	ICAM1
huanglian	MOL000098	quercetin	IFNG
gancao	MOL000098	quercetin	IFNG
huangqin	MOL002714	baicalein	IGF2
huanglian	MOL000098	quercetin	IGF2
gancao	MOL000098	quercetin	IGF2
gancao	MOL000422	kaempferol	IKBKB
huanglian	MOL000098	quercetin	IL10
gancao	MOL000098	quercetin	IL10
huanglian	MOL000098	quercetin	IL1A
gancao	MOL000098	quercetin	IL1A
huanglian	MOL000098	quercetin	IL1B
gancao	MOL000098	quercetin	IL1B
huanglian	MOL000098	quercetin	IL2
gancao	MOL000098	quercetin	IL2
gegen	MOL000392	formononetin	IL4
gancao	MOL000392	formononetin	IL4
huangqin	MOL000173	wogonin	IL6
huangqin	MOL002928	oroxylin a	IL6
huanglian	MOL000098	quercetin	IL6
gancao	MOL000098	quercetin	IL6
huanglian	MOL000098	quercetin	IRF1
gancao	MOL000098	quercetin	IRF1
huangqin	MOL000173	wogonin	KDR
huangqin	MOL002927	Skullcapflavone II	KDR
huangqin	MOL000552	5,2'-Dihydroxy-6,7,8-trimethoxyflavone	KDR
huangqin	MOL012266	rivularin	KDR
gancao	MOL002311	Glycyrol	KDR
gancao	MOL004808	glyasperin B	KDR
gancao	MOL004824	(2S)-6-(2,4-dihydroxyphenyl)-2-(2-hydroxypropan-2-yl)-4-methoxy-2,3-dihydrofuro[3,2-g]chromen-7-one	KDR
gancao	MOL004828	Glepidotin A	KDR

gancao	MOL004848	licochalcone G	KDR
gancao	MOL004849	3-(2,4-dihydroxyphenyl)-8-(1,1-dimethylprop-2-enyl)-7-hydroxy-5-methoxy-coumarin	KDR
gancao	MOL004855	Licoricone	KDR
gancao	MOL004857	Gancaonin B	KDR
gancao	MOL004879	Glycyrin	KDR
gancao	MOL004883	Licoisoflavone	KDR
gancao	MOL004903	liquiritin	KDR
gancao	MOL004904	licopyranocoumarin	KDR
gancao	MOL004935	Sigmoidin-B	KDR
gancao	MOL004959	1-Methoxyphaseollidin	KDR
gancao	MOL004966	3'-Hydroxy-4'-O-Methylglabridin	KDR
gancao	MOL005001	Gancaonin H	KDR
gancao	MOL005007	Glyasperins M	KDR
gancao	MOL005017	Phaseol	KDR
huanglian	MOL000098	quercetin	MAPK1
gancao	MOL004328	naringenin	MAPK1
gancao	MOL000497	licochalcone a	MAPK1
gancao	MOL000098	quercetin	MAPK1
gegen	MOL000392	formononetin	MAPK14
gegen	MOL002959	3'-Methoxydaidzein	MAPK14
huangqin	MOL000173	wogonin	MAPK14
huangqin	MOL002933	5,7,4'-Trihydroxy-8-methoxyflavone	MAPK14
huangqin	MOL008206	Moslosooflavone	MAPK14
gancao	MOL002311	Glycyrol	MAPK14
gancao	MOL000354	isorhamnetin	MAPK14
gancao	MOL003656	Lupiwighteone	MAPK14
gancao	MOL003896	7-Methoxy-2-methyl isoflavone	MAPK14
gancao	MOL000392	formononetin	MAPK14
gancao	MOL000417	Calycosin	MAPK14
gancao	MOL004805	(2S)-2-[4-hydroxy-3-(3-methylbut-2-enyl)phenyl]-8,8-dimethyl-2,3-dihydropyrano[2,3-f]chromen-4-one	MAPK14
gancao	MOL004810	glyasperin F	MAPK14
gancao	MOL004811	Glyasperin C	MAPK14
gancao	MOL004814	Isotrifoliol	MAPK14
gancao	MOL004815	(E)-1-(2,4-dihydroxyphenyl)-3-(2,2-dimethylchromen-6-yl)prop-2-en-1-one	MAPK14
gancao	MOL004820	kanzonols W	MAPK14
gancao	MOL004824	(2S)-6-(2,4-dihydroxyphenyl)-2-(2-hydroxypropan-2-yl)-4-methoxy-2,3-dihydrofuro[3,2-g]chromen-7-one	MAPK14
gancao	MOL004828	Glepidotin A	MAPK14
gancao	MOL004833	Phaseolinisoflavan	MAPK14
gancao	MOL004835	Glypallichalcone	MAPK14

gancao	MOL004841	Licochalcone B	MAPK14
gancao	MOL004848	licochalcone G	MAPK14
gancao	MOL004849	3-(2,4-dihydroxyphenyl)-8-(1,1-dimethylprop-2-enyl)-7-hydroxy-5-methoxy-coumarin	MAPK14
gancao	MOL004863	3-(3,4-dihydroxyphenyl)-5,7-dihydroxy-8-(3-methylbut-2-enyl)chromone	MAPK14
gancao	MOL004864	5,7-dihydroxy-3-(4-methoxyphenyl)-8-(3-methylbut-2-enyl)chromone	MAPK14
gancao	MOL004883	Licoisoflavone	MAPK14
gancao	MOL004891	shinpterocarpin	MAPK14
gancao	MOL004898	(E)-3-[3,4-dihydroxy-5-(3-methylbut-2-enyl)phenyl]-1-(2,4-dihydroxyphenyl)prop-2-en-1-one	MAPK14
gancao	MOL004907	Glyzaglabrin	MAPK14
gancao	MOL004908	Glabridin	MAPK14
gancao	MOL004911	Glabrene	MAPK14
gancao	MOL004912	Glabrone	MAPK14
gancao	MOL004913	1,3-dihydroxy-9-methoxy-6-benzofurano[3,2-c]chromenone	MAPK14
gancao	MOL004914	1,3-dihydroxy-8,9-dimethoxy-6-benzofurano[3,2-c]chromenone	MAPK14
gancao	MOL004915	Eurycarpin A	MAPK14
gancao	MOL004957	HMO	MAPK14
gancao	MOL004959	1-Methoxyphaseollidin	MAPK14
gancao	MOL004961	Quercetin der.	MAPK14
gancao	MOL004966	3'-Hydroxy-4'-O-Methylglabridin	MAPK14
gancao	MOL000497	licochalcone a	MAPK14
gancao	MOL004974	3'-Methoxyglabridin	MAPK14
gancao	MOL004978	2-[(3R)-8,8-dimethyl-3,4-dihydro-2H-pyrano[6,5-f]chromen-3-yl]-5-methoxyphenol	MAPK14
gancao	MOL004990	7,2',4'-trihydroxy − 5-methoxy-3 − arylcoumarin	MAPK14
gancao	MOL004991	7-Acetoxy-2-methylisoflavone	MAPK14
gancao	MOL000500	Vestitol	MAPK14
gancao	MOL005000	Gancaonin G	MAPK14
gancao	MOL005003	Licoagrocarpin	MAPK14
gancao	MOL005012	Licoagroisoflavone	MAPK14
gancao	MOL005016	Odoratin	MAPK14
gancao	MOL005017	Phaseol	MAPK14
gancao	MOL005020	dehydroglyasperins C	MAPK14
huangqin	MOL000173	wogonin	MMP1
huanglian	MOL000098	quercetin	MMP1
gancao	MOL000422	kaempferol	MMP1
gancao	MOL000098	quercetin	MMP1
huanglian	MOL000098	quercetin	MMP2
gancao	MOL000098	quercetin	MMP2
huanglian	MOL000098	quercetin	MMP3

gancao	MOL000098	quercetin	MMP3
huangqin	MOL002714	baicalein	MMP9
huanglian	MOL000098	quercetin	MMP9
gancao	MOL000098	quercetin	MMP9
huangqin	MOL002714	baicalein	MPO
huanglian	MOL000098	quercetin	MPO
gancao	MOL000098	quercetin	MPO
huangqin	MOL002714	baicalein	NFATC1
huanglian	MOL000098	quercetin	NFE2L2
gancao	MOL000098	quercetin	NFE2L2
huanglian	MOL000098	quercetin	NFKBIA
gancao	MOL000098	quercetin	NFKBIA
gegen	MOL000392	formononetin	NOS2
gegen	MOL002959	3'-Methoxydaidzein	NOS2
huangqin	MOL001689	acacetin	NOS2
huangqin	MOL000173	wogonin	NOS2
huangqin	MOL002909	5,7,2,5-tetrahydroxy-8,6-dimethoxyflavone	NOS2
huangqin	MOL002915	Salvigenin	NOS2
huangqin	MOL002917	5,2',6'-Trihydroxy-7,8-dimethoxyflavone	NOS2
huangqin	MOL002927	Skullcapflavone II	NOS2
huangqin	MOL002928	oroxylin a	NOS2
huangqin	MOL002932	Panicolin	NOS2
huangqin	MOL002933	5,7,4'-Trihydroxy-8-methoxyflavone	NOS2
huangqin	MOL002934	NEOBAICALEIN	NOS2
huangqin	MOL000525	Norwogonin	NOS2
huangqin	MOL000552	5,2'-Dihydroxy-6,7,8-trimethoxyflavone	NOS2
huangqin	MOL001458	coptisine	NOS2
huangqin	MOL002897	epiberberine	NOS2
huangqin	MOL008206	Moslosooflavone	NOS2
huangqin	MOL012266	rivularin	NOS2
huanglian	MOL001454	berberine	NOS2
huanglian	MOL002894	berberrubine	NOS2
huanglian	MOL002904	Berlambine	NOS2
huanglian	MOL001458	coptisine	NOS2
huanglian	MOL002897	epiberberine	NOS2
huanglian	MOL000785	palmatine	NOS2
huanglian	MOL002668	Worenine	NOS2
gancao	MOL002311	Glycyrol	NOS2
gancao	MOL000239	Jaranol	NOS2
gancao	MOL002565	Medicarpin	NOS2
gancao	MOL000354	isorhamnetin	NOS2
gancao	MOL003656	Lupiwighteone	NOS2
gancao	MOL003896	7-Methoxy-2-methyl isoflavone	NOS2
gancao	MOL000392	formononetin	NOS2
gancao	MOL000417	Calycosin	NOS2

gancao	MOL000422	kaempferol	NOS2
gancao	MOL004805	(2S)-2-[4-hydroxy-3-(3-methylbut-2-enyl)phenyl]-8,8-dimethyl-2,3-dihydropyrano[2,3-f]chromen-4-one	NOS2
gancao	MOL004806	euchrenone	NOS2
gancao	MOL004808	glyasperin B	NOS2
gancao	MOL004810	glyasperin F	NOS2
gancao	MOL004811	Glyasperin C	NOS2
gancao	MOL004814	Isotrifoliol	NOS2
gancao	MOL004815	(E)-1-(2,4-dihydroxyphenyl)-3-(2,2-dimethylchromen-6-yl)prop-2-en-1-one	NOS2
gancao	MOL004820	kanzonols W	NOS2
gancao	MOL004824	(2S)-6-(2,4-dihydroxyphenyl)-2-(2-hydroxypropan-2-yl)-4-methoxy-2,3-dihydrofuro[3,2-g]chromen-7-one	NOS2
gancao	MOL004827	Semilicoisoflavone B	NOS2
gancao	MOL004828	Glepidotin A	NOS2
gancao	MOL004833	Phaseolinisoflavan	NOS2
gancao	MOL004835	Glypallichalcone	NOS2
gancao	MOL004838	8-(6-hydroxy-2-benzofuranyl)-2,2-dimethyl-5-chromenol	NOS2
gancao	MOL004841	Licochalcone B	NOS2
gancao	MOL004848	licochalcone G	NOS2
gancao	MOL004849	3-(2,4-dihydroxyphenyl)-8-(1,1-dimethylprop-2-enyl)-7-hydroxy-5-methoxy-coumarin	NOS2
gancao	MOL004855	Licoricone	NOS2
gancao	MOL004856	Gancaonin A	NOS2
gancao	MOL004857	Gancaonin B	NOS2
gancao	MOL004863	3-(3,4-dihydroxyphenyl)-5,7-dihydroxy-8-(3-methylbut-2-enyl)chromone	NOS2
gancao	MOL004864	5,7-dihydroxy-3-(4-methoxyphenyl)-8-(3-methylbut-2-enyl)chromone	NOS2
gancao	MOL004879	Glycyrin	NOS2
gancao	MOL004883	Licoisoflavone	NOS2
gancao	MOL004884	Licoisoflavone B	NOS2
gancao	MOL004885	licoisoflavanone	NOS2
gancao	MOL004891	shinpterocarpin	NOS2
gancao	MOL004904	licopyranocoumarin	NOS2
gancao	MOL004907	Glyzaglabrin	NOS2
gancao	MOL004908	Glabridin	NOS2
gancao	MOL004910	Glabranin	NOS2
gancao	MOL004911	Glabrene	NOS2
gancao	MOL004912	Glabrone	NOS2
gancao	MOL004915	Eurycarpin A	NOS2

gancao	MOL004945	(2S)-7-hydroxy-2-(4-hydroxyphenyl)-8-(3-methylbut-2-enyl)chroman-4-one	NOS2
gancao	MOL004948	Isoglycyrol	NOS2
gancao	MOL004949	Isolicoflavonol	NOS2
gancao	MOL004957	HMO	NOS2
gancao	MOL004959	1-Methoxyphaseollidin	NOS2
gancao	MOL004961	Quercetin der.	NOS2
gancao	MOL004966	3'-Hydroxy-4'-O-Methylglabridin	NOS2
gancao	MOL000497	licochalcone a	NOS2
gancao	MOL004974	3'-Methoxyglabridin	NOS2
gancao	MOL004978	2-[(3R)-8,8-dimethyl-3,4-dihydro-2H-pyrano[6,5-f]chromen-3-yl]-5-methoxyphenol	NOS2
gancao	MOL004989	6-prenylated eriodictyol	NOS2
gancao	MOL004990	7,2',4'-trihydroxy − 5-methoxy-3 − arylcoumarin	NOS2
gancao	MOL004991	7-Acetoxy-2-methylisoflavone	NOS2
gancao	MOL000500	Vestitol	NOS2
gancao	MOL005000	Gancaonin G	NOS2
gancao	MOL005003	Licoagrocarpin	NOS2
gancao	MOL005007	Glyasperins M	NOS2
gancao	MOL005008	Glycyrrhiza flavonol A	NOS2
gancao	MOL005012	Licoagroisoflavone	NOS2
gancao	MOL005016	Odoratin	NOS2
gancao	MOL005018	Xambioona	NOS2
gancao	MOL005020	dehydroglyasperins C	NOS2
gegen	MOL000392	formononetin	NOS3
huangqin	MOL002915	Salvigenin	NOS3
huangqin	MOL002927	Skullcapflavone II	NOS3
huangqin	MOL000552	5,2'-Dihydroxy-6,7,8-trimethoxyflavone	NOS3
huangqin	MOL001458	coptisine	NOS3
huangqin	MOL002897	epiberberine	NOS3
huangqin	MOL012266	rivularin	NOS3
huanglian	MOL001454	berberine	NOS3
huanglian	MOL002894	berberrubine	NOS3
huanglian	MOL002904	Berlambine	NOS3
huanglian	MOL001458	coptisine	NOS3
huanglian	MOL002897	epiberberine	NOS3
huanglian	MOL000785	palmatine	NOS3
huanglian	MOL000098	quercetin	NOS3
huanglian	MOL000098	quercetin	NOS3
gancao	MOL000354	isorhamnetin	NOS3
gancao	MOL003896	7-Methoxy-2-methyl isoflavone	NOS3
gancao	MOL000392	formononetin	NOS3
gancao	MOL000422	kaempferol	NOS3
gancao	MOL004828	Glepidotin A	NOS3
gancao	MOL004829	Glepidotin B	NOS3

gancao	MOL004910	Glabranin	NOS3
gancao	MOL004959	1-Methoxyphaseollidin	NOS3
gancao	MOL004978	2-[(3R)-8,8-dimethyl-3,4-dihydro-2H-pyrano[6,5-f]chromen-3-yl]-5-methoxyphenol	NOS3
gancao	MOL004991	7-Acetoxy-2-methylisoflavone	NOS3
gancao	MOL005000	Gancaonin G	NOS3
gancao	MOL005003	Licoagrocarpin	NOS3
gancao	MOL000098	quercetin	NOS3
gancao	MOL000098	quercetin	NOS3
huanglian	MOL000098	quercetin	NPEPPS
gancao	MOL000098	quercetin	NPEPPS
huanglian	MOL000098	quercetin	NQO1
gancao	MOL000098	quercetin	NQO1
huanglian	MOL000098	quercetin	NR1I2
gancao	MOL000422	kaempferol	NR1I2
gancao	MOL000098	quercetin	NR1I2
huanglian	MOL000098	quercetin	ODC1
gancao	MOL000098	quercetin	ODC1
huangqin	MOL000449	Stigmasterol	PLAU
huanglian	MOL000098	quercetin	PLAU
gancao	MOL000098	quercetin	PLAU
gancao	MOL004328	naringenin	PLB1
gegen	MOL000358	beta-sitosterol	PON1
huangqin	MOL000358	beta-sitosterol	PON1
huanglian	MOL000098	quercetin	PON1
gancao	MOL000098	quercetin	PON1
huanglian	MOL000098	quercetin	PPARA
gancao	MOL004328	naringenin	PPARA
gancao	MOL000098	quercetin	PPARA
gegen	MOL000392	formononetin	PPARG
gegen	MOL000392	formononetin	PPARG
gegen	MOL002959	3'-Methoxydaidzein	PPARG
huangqin	MOL000173	wogonin	PPARG
huangqin	MOL002933	5,7,4'-Trihydroxy-8-methoxyflavone	PPARG
huangqin	MOL002934	NEOBAICALEIN	PPARG
huangqin	MOL000525	Norwogonin	PPARG
huangqin	MOL008206	Moslosooflavone	PPARG
huanglian	MOL000098	quercetin	PPARG
huanglian	MOL000098	quercetin	PPARG
gancao	MOL002311	Glycyrol	PPARG
gancao	MOL000354	isorhamnetin	PPARG
gancao	MOL003656	Lupiwighteone	PPARG
gancao	MOL003896	7-Methoxy-2-methyl isoflavone	PPARG
gancao	MOL000392	formononetin	PPARG
gancao	MOL000392	formononetin	PPARG

gancao	MOL000417	Calycosin	PPARG
gancao	MOL000422	kaempferol	PPARG
gancao	MOL000422	kaempferol	PPARG
gancao	MOL004328	naringenin	PPARG
gancao	MOL004805	(2S)-2-[4-hydroxy-3-(3-methylbut-2-enyl)phenyl]-8,8-dimethyl-2,3-dihydropyrano[2,3-f]chromen-4-one	PPARG
gancao	MOL004808	glyasperin B	PPARG
gancao	MOL004810	glyasperin F	PPARG
gancao	MOL004811	Glyasperin C	PPARG
gancao	MOL004815	(E)-1-(2,4-dihydroxyphenyl)-3-(2,2-dimethylchromen-6-yl)prop-2-en-1-one	PPARG
gancao	MOL004820	kanzonols W	PPARG
gancao	MOL004824	(2S)-6-(2,4-dihydroxyphenyl)-2-(2-hydroxypropan-2-yl)-4-methoxy-2,3-dihydrofuro[3,2-g]chromen-7-one	PPARG
gancao	MOL004827	Semilicoisoflavone B	PPARG
gancao	MOL004828	Glepidotin A	PPARG
gancao	MOL004833	Phaseolinisoflavan	PPARG
gancao	MOL004835	Glypallichalcone	PPARG
gancao	MOL004841	Licochalcone B	PPARG
gancao	MOL004848	licochalcone G	PPARG
gancao	MOL004849	3-(2,4-dihydroxyphenyl)-8-(1,1-dimethylprop-2-enyl)-7-hydroxy-5-methoxy-coumarin	PPARG
gancao	MOL004855	Licoricone	PPARG
gancao	MOL004856	Gancaonin A	PPARG
gancao	MOL004857	Gancaonin B	PPARG
gancao	MOL004863	3-(3,4-dihydroxyphenyl)-5,7-dihydroxy-8-(3-methylbut-2-enyl)chromone	PPARG
gancao	MOL004864	5,7-dihydroxy-3-(4-methoxyphenyl)-8-(3-methylbut-2-enyl)chromone	PPARG
gancao	MOL004866	2-(3,4-dihydroxyphenyl)-5,7-dihydroxy-6-(3-methylbut-2-enyl)chromone	PPARG
gancao	MOL004879	Glycyrin	PPARG
gancao	MOL004883	Licoisoflavone	PPARG
gancao	MOL004884	Licoisoflavone B	PPARG
gancao	MOL004885	licoisoflavanone	PPARG
gancao	MOL004891	shinpterocarpin	PPARG
gancao	MOL004898	(E)-3-[3,4-dihydroxy-5-(3-methylbut-2-enyl)phenyl]-1-(2,4-dihydroxyphenyl)prop-2-en-1-one	PPARG
gancao	MOL004904	licopyranocoumarin	PPARG
gancao	MOL004907	Glyzaglabrin	PPARG
gancao	MOL004908	Glabridin	PPARG
gancao	MOL004911	Glabrene	PPARG
gancao	MOL004912	Glabrone	PPARG

gancao	MOL004913	1,3-dihydroxy-9-methoxy-6-benzofurano[3,2-c]chromenone	PPARG
gancao	MOL004914	1,3-dihydroxy-8,9-dimethoxy-6-benzofurano[3,2-c]chromenone	PPARG
gancao	MOL004915	Eurycarpin A	PPARG
gancao	MOL004949	Isolicoflavonol	PPARG
gancao	MOL004957	HMO	PPARG
gancao	MOL004959	1-Methoxyphaseollidin	PPARG
gancao	MOL004961	Quercetin der.	PPARG
gancao	MOL004966	3'-Hydroxy-4'-O-Methylglabridin	PPARG
gancao	MOL000497	licochalcone a	PPARG
gancao	MOL004974	3'-Methoxyglabridin	PPARG
gancao	MOL004978	2-[(3R)-8,8-dimethyl-3,4-dihydro-2H-pyrano[6,5-f]chromen-3-yl]-5-methoxyphenol	PPARG
gancao	MOL004980	Inflacoumarin A	PPARG
gancao	MOL004990	7,2',4'-trihydroxy − 5-methoxy-3 − arylcoumarin	PPARG
gancao	MOL004991	7-Acetoxy-2-methylisoflavone	PPARG
gancao	MOL000500	Vestitol	PPARG
gancao	MOL005000	Gancaonin G	PPARG
gancao	MOL005003	Licoagrocarpin	PPARG
gancao	MOL005007	Glyasperins M	PPARG
gancao	MOL005012	Licoagroisoflavone	PPARG
gancao	MOL005016	Odoratin	PPARG
gancao	MOL005017	Phaseol	PPARG
gancao	MOL005020	dehydroglyasperins C	PPARG
gancao	MOL000098	quercetin	PPARG
gancao	MOL000098	quercetin	PPARG
huanglian	MOL000098	quercetin	PRKCB
gancao	MOL000098	quercetin	PRKCB
gegen	MOL000392	formononetin	PTGS1
gegen	MOL000358	beta-sitosterol	PTGS1
gegen	MOL002959	3'-Methoxydaidzein	PTGS1
huangqin	MOL001689	acacetin	PTGS1
huangqin	MOL000173	wogonin	PTGS1
huangqin	MOL000228	(2R)-7-hydroxy-5-methoxy-2-phenylchroman-4-one	PTGS1
huangqin	MOL002714	baicalein	PTGS1
huangqin	MOL002910	Carthamidin	PTGS1
huangqin	MOL002913	Dihydrobaicalin_qt	PTGS1
huangqin	MOL002914	Eriodyctiol (flavanone)	PTGS1
huangqin	MOL002915	Salvigenin	PTGS1
huangqin	MOL002917	5,2',6'-Trihydroxy-7,8-dimethoxyflavone	PTGS1
huangqin	MOL002925	5,7,2',6'-Tetrahydroxyflavone	PTGS1
huangqin	MOL002927	Skullcapflavone II	PTGS1
huangqin	MOL002928	oroxylin a	PTGS1
huangqin	MOL002932	Panicolin	PTGS1

huangqin	MOL002933	5,7,4'-Trihydroxy-8-methoxyflavone	PTGS1
huangqin	MOL002937	DIHYDROOROXYLIN	PTGS1
huangqin	MOL000358	beta-sitosterol	PTGS1
huangqin	MOL000525	Norwogonin	PTGS1
huangqin	MOL000552	5,2'-Dihydroxy-6,7,8-trimethoxyflavone	PTGS1
huangqin	MOL000073	ent-Epicatechin	PTGS1
huangqin	MOL000449	Stigmasterol	PTGS1
huangqin	MOL001458	coptisine	PTGS1
huangqin	MOL008206	Moslosooflavone	PTGS1
huangqin	MOL012245	5,7,4'-trihydroxy-6-methoxyflavanone	PTGS1
huangqin	MOL012245	5,7,4'-trihydroxy-6-methoxyflavanone	PTGS1
huangqin	MOL012266	rivularin	PTGS1
huanglian	MOL002903	(R)-Canadine	PTGS1
huanglian	MOL001454	berberine	PTGS1
huanglian	MOL002894	berberrubine	PTGS1
huanglian	MOL002904	Berlambine	PTGS1
huanglian	MOL001458	coptisine	PTGS1
huanglian	MOL000785	palmatine	PTGS1
huanglian	MOL000098	quercetin	PTGS1
huanglian	MOL002668	Worenine	PTGS1
gancao	MOL001484	Inermine	PTGS1
gancao	MOL001792	DFV	PTGS1
gancao	MOL000239	Jaranol	PTGS1
gancao	MOL002565	Medicarpin	PTGS1
gancao	MOL000354	isorhamnetin	PTGS1
gancao	MOL003896	7-Methoxy-2-methyl isoflavone	PTGS1
gancao	MOL000392	formononetin	PTGS1
gancao	MOL000417	Calycosin	PTGS1
gancao	MOL000422	kaempferol	PTGS1
gancao	MOL004328	naringenin	PTGS1
gancao	MOL004810	glyasperin F	PTGS1
gancao	MOL004815	(E)-1-(2,4-dihydroxyphenyl)-3-(2,2-dimethylchromen-6-yl)prop-2-en-1-one	PTGS1
gancao	MOL004820	kanzonols W	PTGS1
gancao	MOL004828	Glepidotin A	PTGS1
gancao	MOL004829	Glepidotin B	PTGS1
gancao	MOL004835	Glypallichalcone	PTGS1
gancao	MOL004841	Licochalcone B	PTGS1
gancao	MOL004885	licoisoflavanone	PTGS1
gancao	MOL004891	shinpterocarpin	PTGS1
gancao	MOL004907	Glyzaglabrin	PTGS1
gancao	MOL004910	Glabranin	PTGS1
gancao	MOL004911	Glabrene	PTGS1
gancao	MOL004912	Glabrone	PTGS1
gancao	MOL004941	(2R)-7-hydroxy-2-(4-hydroxyphenyl)chroman-4-one	PTGS1

gancao	MOL004945	(2S)-7-hydroxy-2-(4-hydroxyphenyl)-8-(3-methylbut-2-enyl)chroman-4-one	PTGS1
gancao	MOL004957	HMO	PTGS1
gancao	MOL004959	1-Methoxyphaseollidin	PTGS1
gancao	MOL004961	Quercetin der.	PTGS1
gancao	MOL004966	3'-Hydroxy-4'-O-Methylglabridin	PTGS1
gancao	MOL000497	licochalcone a	PTGS1
gancao	MOL004974	3'-Methoxyglabridin	PTGS1
gancao	MOL004978	2-[(3R)-8,8-dimethyl-3,4-dihydro-2H-pyrano[6,5-f]chromen-3-yl]-5-methoxyphenol	PTGS1
gancao	MOL004980	Inflacoumarin A	PTGS1
gancao	MOL004990	7,2',4'-trihydroxy − 5-methoxy-3 − arylcoumarin	PTGS1
gancao	MOL004991	7-Acetoxy-2-methylisoflavone	PTGS1
gancao	MOL000500	Vestitol	PTGS1
gancao	MOL005003	Licoagrocarpin	PTGS1
gancao	MOL005007	Glyasperins M	PTGS1
gancao	MOL005016	Odoratin	PTGS1
gancao	MOL000098	quercetin	PTGS1
gegen	MOL000392	formononetin	PTGS2
gegen	MOL000358	beta-sitosterol	PTGS2
gegen	MOL002959	3'-Methoxydaidzein	PTGS2
huangqin	MOL001689	acacetin	PTGS2
huangqin	MOL000173	wogonin	PTGS2
huangqin	MOL000228	(2R)-7-hydroxy-5-methoxy-2-phenylchroman-4-one	PTGS2
huangqin	MOL002714	baicalein	PTGS2
huangqin	MOL002909	5,7,2,5-tetrahydroxy-8,6-dimethoxyflavone	PTGS2
huangqin	MOL002910	Carthamidin	PTGS2
huangqin	MOL002913	Dihydrobaicalin_qt	PTGS2
huangqin	MOL002914	Eriodyctiol (flavanone)	PTGS2
huangqin	MOL002915	Salvigenin	PTGS2
huangqin	MOL002917	5,2',6'-Trihydroxy-7,8-dimethoxyflavone	PTGS2
huangqin	MOL002925	5,7,2',6'-Tetrahydroxyflavone	PTGS2
huangqin	MOL002927	Skullcapflavone II	PTGS2
huangqin	MOL002928	oroxylin a	PTGS2
huangqin	MOL002932	Panicolin	PTGS2
huangqin	MOL002933	5,7,4'-Trihydroxy-8-methoxyflavone	PTGS2
huangqin	MOL002934	NEOBAICALEIN	PTGS2
huangqin	MOL002937	DIHYDROOROXYLIN	PTGS2
huangqin	MOL000358	beta-sitosterol	PTGS2
huangqin	MOL000525	Norwogonin	PTGS2
huangqin	MOL000552	5,2'-Dihydroxy-6,7,8-trimethoxyflavone	PTGS2
huangqin	MOL000073	ent-Epicatechin	PTGS2
huangqin	MOL000449	Stigmasterol	PTGS2
huangqin	MOL001458	coptisine	PTGS2
huangqin	MOL002897	epiberberine	PTGS2

huangqin	MOL008206	Moslosooflavone	PTGS2
huangqin	MOL012245	5,7,4'-trihydroxy-6-methoxyflavanone	PTGS2
huangqin	MOL012245	5,7,4'-trihydroxy-6-methoxyflavanone	PTGS2
huangqin	MOL012266	rivularin	PTGS2
huanglian	MOL002903	(R)-Canadine	PTGS2
huanglian	MOL001454	berberine	PTGS2
huanglian	MOL002894	berberrubine	PTGS2
huanglian	MOL002904	Berlambine	PTGS2
huanglian	MOL001458	coptisine	PTGS2
huanglian	MOL002897	epiberberine	PTGS2
huanglian	MOL000785	palmatine	PTGS2
huanglian	MOL000098	quercetin	PTGS2
huanglian	MOL002668	Worenine	PTGS2
gancao	MOL001484	Inermine	PTGS2
gancao	MOL001792	DFV	PTGS2
gancao	MOL002311	Glycyrol	PTGS2
gancao	MOL000239	Jaranol	PTGS2
gancao	MOL002565	Medicarpin	PTGS2
gancao	MOL000354	isorhamnetin	PTGS2
gancao	MOL003656	Lupiwighteone	PTGS2
gancao	MOL003896	7-Methoxy-2-methyl isoflavone	PTGS2
gancao	MOL000392	formononetin	PTGS2
gancao	MOL000417	Calycosin	PTGS2
gancao	MOL000422	kaempferol	PTGS2
gancao	MOL004328	naringenin	PTGS2
gancao	MOL004805	(2S)-2-[4-hydroxy-3-(3-methylbut-2-enyl)phenyl]-8,8-dimethyl-2,3-dihydropyrano[2,3-f]chromen-4-one	PTGS2
gancao	MOL004806	euchrenone	PTGS2
gancao	MOL004808	glyasperin B	PTGS2
gancao	MOL004810	glyasperin F	PTGS2
gancao	MOL004811	Glyasperin C	PTGS2
gancao	MOL004814	Isotrifoliol	PTGS2
gancao	MOL004815	(E)-1-(2,4-dihydroxyphenyl)-3-(2,2-dimethylchromen-6-yl)prop-2-en-1-one	PTGS2
gancao	MOL004820	kanzonols W	PTGS2
gancao	MOL004824	(2S)-6-(2,4-dihydroxyphenyl)-2-(2-hydroxypropan-2-yl)-4-methoxy-2,3-dihydrofuro[3,2-g]chromen-7-one	PTGS2
gancao	MOL004827	Semilicoisoflavone B	PTGS2
gancao	MOL004828	Glepidotin A	PTGS2
gancao	MOL004829	Glepidotin B	PTGS2
gancao	MOL004833	Phaseolinisoflavan	PTGS2
gancao	MOL004835	Glypallichalcone	PTGS2

gancao	MOL004838	8-(6-hydroxy-2-benzofuranyl)-2,2-dimethyl-5-chromenol	PTGS2
gancao	MOL004841	Licochalcone B	PTGS2
gancao	MOL004848	licochalcone G	PTGS2
gancao	MOL004849	3-(2,4-dihydroxyphenyl)-8-(1,1-dimethylprop-2-enyl)-7-hydroxy-5-methoxy-coumarin	PTGS2
gancao	MOL004855	Licoricone	PTGS2
gancao	MOL004856	Gancaonin A	PTGS2
gancao	MOL004857	Gancaonin B	PTGS2
gancao	MOL004863	3-(3,4-dihydroxyphenyl)-5,7-dihydroxy-8-(3-methylbut-2-enyl)chromone	PTGS2
gancao	MOL004864	5,7-dihydroxy-3-(4-methoxyphenyl)-8-(3-methylbut-2-enyl)chromone	PTGS2
gancao	MOL004866	2-(3,4-dihydroxyphenyl)-5,7-dihydroxy-6-(3-methylbut-2-enyl)chromone	PTGS2
gancao	MOL004879	Glycyrin	PTGS2
gancao	MOL004883	Licoisoflavone	PTGS2
gancao	MOL004884	Licoisoflavone B	PTGS2
gancao	MOL004885	licoisoflavanone	PTGS2
gancao	MOL004891	shinpterocarpin	PTGS2
gancao	MOL004898	(E)-3-[3,4-dihydroxy-5-(3-methylbut-2-enyl)phenyl]-1-(2,4-dihydroxyphenyl)prop-2-en-1-one	PTGS2
gancao	MOL004903	liquiritin	PTGS2
gancao	MOL004904	licopyranocoumarin	PTGS2
gancao	MOL004907	Glyzaglabrin	PTGS2
gancao	MOL004908	Glabridin	PTGS2
gancao	MOL004910	Glabranin	PTGS2
gancao	MOL004911	Glabrene	PTGS2
gancao	MOL004912	Glabrone	PTGS2
gancao	MOL004915	Eurycarpin A	PTGS2
gancao	MOL004924	(-)-Medicocarpin	PTGS2
gancao	MOL004935	Sigmoidin-B	PTGS2
gancao	MOL004941	(2R)-7-hydroxy-2-(4-hydroxyphenyl)chroman-4-one	PTGS2
gancao	MOL004945	(2S)-7-hydroxy-2-(4-hydroxyphenyl)-8-(3-methylbut-2-enyl)chroman-4-one	PTGS2
gancao	MOL004948	Isoglycyrol	PTGS2
gancao	MOL004949	Isolicoflavonol	PTGS2
gancao	MOL004957	HMO	PTGS2
gancao	MOL004959	1-Methoxyphaseollidin	PTGS2
gancao	MOL004961	Quercetin der.	PTGS2
gancao	MOL004966	3'-Hydroxy-4'-O-Methylglabridin	PTGS2
gancao	MOL000497	licochalcone a	PTGS2
gancao	MOL004974	3'-Methoxyglabridin	PTGS2
gancao	MOL004978	2-[(3R)-8,8-dimethyl-3,4-dihydro-2H-pyrano[6,5-f]chromen-3-yl]-5-methoxyphenol	PTGS2

gancao	MOL004980	Inflacoumarin A	PTGS2
gancao	MOL004988	Kanzonol F	PTGS2
gancao	MOL004989	6-prenylated eriodictyol	PTGS2
gancao	MOL004990	7,2',4'-trihydroxy − 5-methoxy-3 − arylcoumarin	PTGS2
gancao	MOL004991	7-Acetoxy-2-methylisoflavone	PTGS2
gancao	MOL004993	8-prenylated eriodictyol	PTGS2
gancao	MOL000500	Vestitol	PTGS2
gancao	MOL005000	Gancaonin G	PTGS2
gancao	MOL005001	Gancaonin H	PTGS2
gancao	MOL005003	Licoagrocarpin	PTGS2
gancao	MOL005007	Glyasperins M	PTGS2
gancao	MOL005008	Glycyrrhiza flavonol A	PTGS2
gancao	MOL005012	Licoagroisoflavone	PTGS2
gancao	MOL005016	Odoratin	PTGS2
gancao	MOL005017	Phaseol	PTGS2
gancao	MOL005018	Xambioona	PTGS2
gancao	MOL005020	dehydroglyasperins C	PTGS2
gancao	MOL000098	quercetin	PTGS2
huanglian	MOL000098	quercetin	RASSF1
gancao	MOL000098	quercetin	RASSF1
huangqin	MOL001689	acacetin	RELA
huangqin	MOL000173	wogonin	RELA
huangqin	MOL002714	baicalein	RELA
huanglian	MOL000098	quercetin	RELA
gancao	MOL000354	isorhamnetin	RELA
gancao	MOL000422	kaempferol	RELA
gancao	MOL004328	naringenin	RELA
gancao	MOL000497	licochalcone a	RELA
gancao	MOL000098	quercetin	RELA
huanglian	MOL000098	quercetin	SERPINE1
gancao	MOL000098	quercetin	SERPINE1
gegen	MOL000392	formononetin	SLC6A4
gegen	MOL000358	beta-sitosterol	SLC6A4
huangqin	MOL000228	(2R)-7-hydroxy-5-methoxy-2-phenylchroman-4-one	SLC6A4
huangqin	MOL000358	beta-sitosterol	SLC6A4
huanglian	MOL002903	(R)-Canadine	SLC6A4
gancao	MOL001792	DFV	SLC6A4
gancao	MOL002565	Medicarpin	SLC6A4
gancao	MOL003896	7-Methoxy-2-methyl isoflavone	SLC6A4
gancao	MOL000392	formononetin	SLC6A4
gancao	MOL004835	Glypallichalcone	SLC6A4
gancao	MOL004941	(2R)-7-hydroxy-2-(4-hydroxyphenyl)chroman-4-one	SLC6A4
gancao	MOL004957	HMO	SLC6A4
gancao	MOL000500	Vestitol	SLC6A4
gancao	MOL000422	kaempferol	SLPI

huanglian	MOL000098	quercetin	SPP1
gancao	MOL000098	quercetin	SPP1
huanglian	MOL000098	quercetin	STAT1
gancao	MOL000422	kaempferol	STAT1
gancao	MOL000098	quercetin	STAT1
gancao	MOL000497	licochalcone a	STAT3
gegen	MOL000358	beta-sitosterol	TGFB1
huangqin	MOL000358	beta-sitosterol	TGFB1
huanglian	MOL000098	quercetin	TGFB1
gancao	MOL000098	quercetin	TGFB1
huangqin	MOL000173	wogonin	TNF
huanglian	MOL000098	quercetin	TNF
gancao	MOL000422	kaempferol	TNF
gancao	MOL000098	quercetin	TNF
huangqin	MOL001689	acacetin	TP53
huangqin	MOL000173	wogonin	TP53
huangqin	MOL002714	baicalein	TP53
huanglian	MOL000098	quercetin	TP53
gancao	MOL000098	quercetin	TP53
huanglian	MOL000098	quercetin	VCAM1
gancao	MOL000422	kaempferol	VCAM1
gancao	MOL000098	quercetin	VCAM1
huangqin	MOL002714	baicalein	VEGFA
huanglian	MOL000098	quercetin	VEGFA
gancao	MOL000098	quercetin	VEGFA
huanglian	MOL000098	quercetin	XDH
gancao	MOL000354	isorhamnetin	XDH
gancao	MOL000422	kaempferol	XDH
gancao	MOL000098	quercetin	XDH

Table S4: GO and KEGG Pathway Enrichment Analysis of the C-DT Network.

ID	Description	GeneRatio	BgRatio	pvalue	p.adjust	qvalue	geneID	Count
GO:0005126	cytokine receptor binding	17/83	286/17697	1.70E-14	7.78E-12	3.51E-12	CD40LG/IFNG/CASP3/CCL2/CXCL10/CXCL8/IL10/IL1A/IL1B/IL2/IL4/IL6/STAT1/STAT3/TGFB1/TNF/VEGFA	17
GO:0020037	heme binding	12/83	135/17697	1.44E-12	2.20E-10	9.93E-11	CYP1A1/CYP1B1/CYP2C9/CYP3A4/MPO/NOS2/PTGS1/PTGS2/CAT/DUOX2/HMOX1/NOS3	12
GO:0048018	receptor ligand activity	17/83	482/17697	7.34E-11	6.71E-09	3.03E-09	CD40LG/IFNG/CCL2/CXCL10/CXCL8/EGF/IGF2/IL10/IL1A/IL1B/IL2/IL4/IL6/SPP1/TGFB1/TNF/VEGFA	17
GO:0001085	RNA polymerase II transcription factor binding	9/83	155/17697	4.54E-08	3.07E-06	1.39E-06	PPARG/AHR/ESR1/FOS/NFATC1/NFE2L2/PPARA/STAT3/TP53	9
GO:0001221	transcription cofactor binding	6/83	43/17697	4.70E-08	3.07E-06	1.39E-06	AHR/ESR1/NFATC1/NFE2L2/PPARA/RELA	6
GO:0016705	oxidoreductase activity, acting on paired donors, with incorporation or reduction of molecular oxygen	9/83	159/17697	5.66E-08	3.21E-06	1.45E-06	CYP1A1/CYP1B1/CYP2C9/CYP3A4/NOS2/PTGS1/PTGS2/HMOX1/NOS3	9

GO:0004879	nuclear receptor activity	6/83	47/17697	8.16E-08	3.39E-06	1.53E-06	NR1I2/PPARG/AHR/ESR1/PPARA/STAT3	6
GO:0098531	transcription factor activity, direct ligand regulated sequence-specific DNA binding	6/83	47/17697	8.16E-08	3.39E-06	1.53E-06	NR1I2/PPARG/AHR/ESR1/PPARA/STAT3	6
GO:0004601	peroxidase activity	6/83	52/17697	1.52E-07	5.41E-06	2.44E-06	MPO/PTGS1/PTGS2/CAT/DUOX2/GSTP1	6
GO:0016209	antioxidant activity	7/83	86/17697	1.54E-07	5.41E-06	2.44E-06	MPO/PTGS1/PTGS2/CAT/DUOX2/GSTP1/NQO1	7
GO:0019902	phosphatase binding	9/83	185/17697	2.07E-07	6.53E-06	2.95E-06	PPARG/BCL2/EGFR/MAPK1/MAPK14/PPARA/STAT1/STAT3/TP53	9
GO:0019207	kinase regulator activity	9/83	207/17697	5.36E-07	1.44E-05	6.50E-06	CASP3/CCND1/CDKN1A/CXCL10/EGF/GSTP1/IGF2/IL2/TGFB1	9
GO:0005178	integrin binding	7/83	132/17697	2.84E-06	6.82E-05	3.08E-05	EGFR/ICAM1/IGF2/IL1B/KDR/SPP1/VCAM1	7
GO:0101020	estrogen 16-alpha-hydroxylase activity	3/83	8/17697	5.48E-06	0.000119196	5.38E-05	CYP1A1/CYP1B1/CYP3A4	3
GO:0001228	DNA-binding transcription activator activity, RNA polymerase II-specific	11/83	439/17697	6.25E-06	0.000129927	5.87E-05	NR1I2/ESR1/FOS/IRF1/NFATC1/NFE2L2/PPARA/RELA/STAT1/STAT3/TP53	11

GO:0016653	oxidoreductase activity, acting on NAD(P)H, heme protein as acceptor	3/83	13/17697	2.75E-05	0.00041896	0.000189143	NOS2/NOS3/NQO1	3
GO:0016725	oxidoreductase activity, acting on CH or CH2 groups	3/83	13/17697	2.75E-05	0.00041896	0.000189143	CYP2C9/XDH/CYP3A4	3
GO:0008384	IkappaB kinase activity	2/83	3/17697	6.50E-05	0.000802834	0.000362445	CHUK/IKBKB	2
GO:0070576	vitamin D 24-hydroxylase activity	2/83	3/17697	6.50E-05	0.000802834	0.000362445	CYP1A1/CYP3A4	2
GO:0005506	iron ion binding	6/83	152/17697	7.96E-05	0.000957618	0.000432323	ALOX5/CYP1A1/CYP1B1/CYP2C9/XDH/CYP3A4	6
GO:0050661	NADP binding	4/83	52/17697	0.000102596	0.001202217	0.000542749	NOS2/CAT/FASN/NOS3	4
GO:0034617	tetrahydrobiopterin binding	2/83	4/17697	0.000129603	0.001316188	0.000594202	NOS2/NOS3	2
GO:0101021	estrogen 2-hydroxylase activity	2/83	4/17697	0.000129603	0.001316188	0.000594202	CYP1A1/CYP3A4	2
GO:0003707	steroid hormone receptor activity	4/83	56/17697	0.000137223	0.001363285	0.000615464	NR1I2/PPARG/ESR1/PPARA	4
GO:0048273	mitogen-activated protein kinase p38 binding	2/83	5/17697	0.000215346	0.002008429	0.000906719	MAPK14/NFATC1	2
GO:0051434	BH3 domain binding	2/83	5/17697	0.000215346	0.002008429	0.000906719	BAX/BCL2	2
GO:0030374	nuclear receptor transcription coactivator activity	4/83	67/17697	0.000275378	0.002516953	0.001136296	NR1I2/PPARG/PPARA/PRKCB	4
GO:0004064	arylesterase activity	2/83	6/17697	0.000322034	0.002830183	0.001277706	CA2/PON1	2

GO:0035326	enhancer binding	5/83	133/17697	0.000403716	0.003318997	0.001498384	AHR/NFATC1/NFE2L2/RELA/TP53	5
GO:0034618	arginine binding	2/83	7/17697	0.000449474	0.00342349	0.001545558	NOS2/NOS3	2
GO:0050839	cell adhesion molecule binding	9/83	499/17697	0.00054147	0.00399936	0.001805538	EGFR/FASN/ICAM1/IGF2/IL1B/KDR/SPP1/STAT1/VCAM1	9
GO:0030235	nitric-oxide synthase regulator activity	2/83	8/17697	0.000597472	0.004194185	0.001893494	EGFR/ESR1	2
GO:0097371	MDM2/MDM4 family protein binding	2/83	8/17697	0.000597472	0.004194185	0.001893494	PPARA/TP53	2
GO:0019825	oxygen binding	3/83	36/17697	0.000635203	0.00433265	0.001956005	CYP1A1/CYP1B1/CYP3A4	3
GO:0051879	Hsp90 protein binding	3/83	41/17697	0.000932488	0.005664335	0.002557203	CYP1A1/AHR/KDR	3
GO:0004252	serine-type endopeptidase activity	5/83	160/17697	0.000933847	0.005664335	0.002557203	MMP1/MMP2/MMP3/MMP9/PLAU	5
GO:0043176	amine binding	2/83	10/17697	0.000954385	0.005664335	0.002557203	HTR3A/SLC6A4	2
GO:0051378	serotonin binding	2/83	10/17697	0.000954385	0.005664335	0.002557203	HTR3A/SLC6A4	2
GO:0043295	glutathione binding	2/83	11/17697	0.00116292	0.006561167	0.002962084	GSTM1/GSTP1	2
GO:0004861	cyclin-dependent protein serine/threonine kinase inhibitor activity	2/83	12/17697	0.001391259	0.007429076	0.003353907	CASP3/CDKN1A	2
GO:1900750	oligopeptide binding	2/83	12/17697	0.001391259	0.007429076	0.003353907	GSTM1/GSTP1	2
GO:0017171	serine hydrolase activity	5/83	186/17697	0.001819695	0.008941942	0.004036901	MMP1/MMP2/MMP3/MMP9/PLAU	5
GO:0042165	neurotransmitter binding	3/83	55/17697	0.002189204	0.010421521	0.004704867	CRP/HTR3A/SLC6A4	3

GO:0097110	scaffold protein binding	3/83	59/17697	0.0026765	0.011875343	0.005361205	CHUK/IKBKB/NOS3	3
GO:0004051	arachidonate 5-lipoxygenase activity	1/83	1/17697	0.00469006	0.016733806	0.007554592	ALOX5	1
GO:0004313	[acyl-carrier-protein] S-acetyltransferase activity	1/83	1/17697	0.00469006	0.016733806	0.007554592	FASN	1
GO:0004316	3-oxoacyl-[acyl-carrier-protein] reductase (NADPH) activity	1/83	1/17697	0.00469006	0.016733806	0.007554592	FASN	1
GO:0008659	(3R)-hydroxymyristoyl-[acyl-carrier-protein] dehydratase activity	1/83	1/17697	0.00469006	0.016733806	0.007554592	FASN	1
GO:0019171	3-hydroxyacyl-[acyl-carrier-protein] dehydratase activity	1/83	1/17697	0.00469006	0.016733806	0.007554592	FASN	1
GO:0019912	cyclin-dependent protein kinase activating kinase activity	1/83	1/17697	0.00469006	0.016733806	0.007554592	CDKN1A	1
GO:0031177	phosphopantetheine binding	1/83	1/17697	0.00469006	0.016733806	0.007554592	FASN	1
GO:0035730	S-nitrosoglutathione binding	1/83	1/17697	0.00469006	0.016733806	0.007554592	GSTP1	1
GO:0035731	dinitrosyl-iron complex binding	1/83	1/17697	0.00469006	0.016733806	0.007554592	GSTP1	1

GO:0047117	enoyl-[acyl-carrier-protein] reductase (NADPH, A-specific) activity	1/83	1/17697	0.00469006	0.016733806	0.007554592	FASN	1
GO:0047451	3-hydroxyoctanoyl-[acyl-carrier-protein] dehydratase activity	1/83	1/17697	0.00469006	0.016733806	0.007554592	FASN	1
GO:0102131	3-oxo-glutaryl-[acp] methyl ester reductase activity	1/83	1/17697	0.00469006	0.016733806	0.007554592	FASN	1
GO:0102132	3-oxo-pimeloyl-[acp] methyl ester reductase activity	1/83	1/17697	0.00469006	0.016733806	0.007554592	FASN	1
GO:0070405	ammonium ion binding	3/83	75/17697	0.005268759	0.018521714	0.008361755	CRP/HTR3A/SLC6A4	3
GO:0004364	glutathione transferase activity	2/83	27/17697	0.007069306	0.023754947	0.010724334	GSTM1/GSTP1	2
GO:0072341	modified amino acid binding	3/83	89/17697	0.008455528	0.026077947	0.011773068	FASN/GSTM1/GSTP1	3
GO:0004063	aryldialkylphosphatase activity	1/83	2/17697	0.009358388	0.026077947	0.011773068	PON1	1
GO:0004314	[acyl-carrier-protein] S-malonyltransferase activity	1/83	2/17697	0.009358388	0.026077947	0.011773068	FASN	1
GO:0004315	3-oxoacyl-[acyl-carrier-protein] synthase activity	1/83	2/17697	0.009358388	0.026077947	0.011773068	FASN	1
GO:0004320	oleoyl-[acyl-carrier-protein] hydrolase activity	1/83	2/17697	0.009358388	0.026077947	0.011773068	FASN	1

GO:0004586	ornithine decarboxylase activity	1/83	2/17697	0.009358388	0.026077947	0.011773068	ODC1	1
GO:0005006	epidermal growth factor-activated receptor activity	1/83	2/17697	0.009358388	0.026077947	0.011773068	EGFR	1
GO:0016005	phospholipase A2 activator activity	1/83	2/17697	0.009358388	0.026077947	0.011773068	CASP3	1
GO:0016295	myristoyl-[acyl-carrier-protein] hydrolase activity	1/83	2/17697	0.009358388	0.026077947	0.011773068	FASN	1
GO:0016296	palmitoyl-[acyl-carrier-protein] hydrolase activity	1/83	2/17697	0.009358388	0.026077947	0.011773068	FASN	1
GO:0016297	acyl-[acyl-carrier-protein] hydrolase activity	1/83	2/17697	0.009358388	0.026077947	0.011773068	FASN	1
GO:0016419	S-malonyltransferase activity	1/83	2/17697	0.009358388	0.026077947	0.011773068	FASN	1
GO:0016420	malonyltransferase activity	1/83	2/17697	0.009358388	0.026077947	0.011773068	FASN	1
GO:0070026	nitric oxide binding	1/83	2/17697	0.009358388	0.026077947	0.011773068	GSTP1	1
GO:0016829	lyase activity	4/83	187/17697	0.011604819	0.031567872	0.014251533	CYP1A1/CA2/FASN/ODC1	4
GO:0050662	coenzyme binding	5/83	291/17697	0.011893684	0.03216221	0.014519851	XDH/NOS2/CAT/FASN/NOS3	5
GO:0042056	chemoattractant activity	2/83	38/17697	0.013695415	0.034410336	0.015534783	CXCL10/VEGFA	2
GO:0004938	alpha2-adrenergic receptor activity	1/83	3/17697	0.014005082	0.034410336	0.015534783	ADRA2A	1

GO:0005335	serotonin:sodium symporter activity	1/83	3/17697	0.014005082	0.034410336	0.015534783	SLC6A4	1
GO:0030343	vitamin D3 25-hydroxylase activity	1/83	3/17697	0.014005082	0.034410336	0.015534783	CYP3A4	1
GO:0043183	vascular endothelial growth factor receptor 1 binding	1/83	3/17697	0.014005082	0.034410336	0.015534783	VEGFA	1
GO:0070643	vitamin D 25-hydroxylase activity	1/83	3/17697	0.014005082	0.034410336	0.015534783	CYP3A4	1
GO:0102007	acyl-L-homoserine-lactone lactonohydrolase activity	1/83	3/17697	0.014005082	0.034410336	0.015534783	PON1	1
GO:0047485	protein N-terminus binding	3/83	109/17697	0.014622491	0.035735178	0.016132892	RELA/TGFB1/TP53	3
GO:0043177	organic acid binding	4/83	205/17697	0.015782628	0.037961374	0.017137923	NOS2/PPARG/NOS3/PPARA	4
GO:0004784	superoxide dismutase activity	1/83	4/17697	0.018630242	0.041735395	0.018841731	NQO1	1
GO:0008559	xenobiotic transmembrane transporting ATPase activity	1/83	4/17697	0.018630242	0.041735395	0.018841731	ABCG2	1
GO:0016721	oxidoreductase activity, acting on superoxide radicals as acceptor	1/83	4/17697	0.018630242	0.041735395	0.018841731	NQO1	1
GO:0032795	heterotrimeric G-protein binding	1/83	4/17697	0.018630242	0.041735395	0.018841731	ADRA2A	1
GO:0033265	choline binding	1/83	4/17697	0.018630242	0.041735395	0.018841731	CRP	1

GO:0035033	histone deacetylase regulator activity	1/83	4/17697	0.018630242	0.041735395	0.018841731	TP53	1
GO:0048408	epidermal growth factor binding	1/83	4/17697	0.018630242	0.041735395	0.018841731	EGFR	1
GO:0097200	cysteine-type endopeptidase activity involved in execution phase of apoptosis	1/83	4/17697	0.018630242	0.041735395	0.018841731	CASP3	1
GO:0042805	actinin binding	2/83	46/17697	0.019682793	0.043878227	0.019809127	PPARG/RELA	2
GO:1990841	promoter-specific chromatin binding	2/83	48/17697	0.02132268	0.047303227	0.021355367	STAT1/TP53	2
GO:0004046	aminoacylase activity	1/83	5/17697	0.023233966	0.048930519	0.022090019	CAT	1
GO:0022850	serotonin-gated cation-selective channel activity	1/83	5/17697	0.023233966	0.048930519	0.022090019	HTR3A	1
GO:0051380	norepinephrine binding	1/83	5/17697	0.023233966	0.048930519	0.022090019	ADRA2A	1

ID	Description	GeneRatio	BgRatio	pvalue	p.adjust	qvalue	geneID	Count
GO:0072593	reactive oxygen species metabolic process	26/83	284/18670	1.91E-27	7.79E-24	2.73E-24	CYP1A1/CYP1B1/XDH/IFNG/MPO/NOS2/PTGS2/BCL2/CAT/CDKN1A/CRP/DUOX2/EGFR/GSTP1/ICAM1/IL10/IL1B/MAPK14/MMP3/NFE2L2/NOS3/NQO1/STAT3/TGFB1/TNF/TP53	26
GO:0032496	response to lipopolysaccharide	25/83	330/18670	2.64E-24	5.37E-21	1.88E-21	CHUK/CYP1A1/MPO/NOS2/PTGS2/CASP3/CCL2/CXCL10/CXCL8/FOS/GSTP1/ICAM1/IL10/IL1B/IL6/MAPK1/MAPK14/NFKBIA/NOS3/RELA/SERPINE1/SLPI/TGFB1/TNF/VCAM1	25
GO:0000302	response to reactive oxygen species	22/83	232/18670	1.54E-23	1.57E-20	5.48E-21	CHUK/CYP1B1/MPO/BCL2/CASP3/CAT/EGFR/FOS/GSTP1/HMOX1/IL10/IL6/MAPK1/MMP2/MMP3/MMP9/NFE2L2/NOS3/NQO1/RELA/STAT1/TNF	22
GO:0006979	response to oxidative stress	26/83	451/18670	3.02E-22	2.46E-19	8.62E-20	CHUK/CYP1B1/MPO/PTGS1/PTGS2/BCL2/CASP3/CAT/DUOX2/EGFR/FOS/GSTP1/HMOX1/IL10/IL6/MAPK1/MMP2/MMP3/MMP9/NFE2L2/NOS3/NQO1/RELA/STAT1/TNF/TP53	26

GO:0031667	response to nutrient levels	26/83	499/18670	3.90E-21	2.65E-18	9.26E-19	CYP1A1/MPO/PPARG/PTGS2/BCL2/CAT/CCND1/CDKN1A/CXCL10/EGFR/GSTP1/HMOX1/ICAM1/IL1B/MAPK1/NFE2L2/NQO1/PON1/PPARA/RELA/SLC6A4/SPP1/STAT1/TGFB1/TP53/VCAM1	26
GO:2000377	regulation of reactive oxygen species metabolic process	19/83	195/18670	1.34E-20	7.79E-18	2.72E-18	CYP1B1/XDH/IFNG/PTGS2/BCL2/CDKN1A/CRP/EGFR/GSTP1/ICAM1/IL10/IL1B/MAPK14/MMP3/NFE2L2/STAT3/TGFB1/TNF/TP53	19
GO:0046677	response to antibiotic	22/83	327/18670	2.94E-20	1.50E-17	5.24E-18	CHUK/CYP1A1/CYP1B1/AHR/BCL2/CASP3/CAT/CCND1/GSTP1/HMOX1/HTR3A/ICAM1/IL10/IL2/IL6/NFE2L2/NQO1/RELA/STAT1/STAT3/TP53/VCAM1	22
GO:0034599	cellular response to oxidative stress	21/83	302/18670	1.21E-19	5.46E-17	1.91E-17	CHUK/CYP1B1/MPO/BCL2/CAT/EGFR/FOS/GSTP1/HMOX1/IL10/IL6/MAPK1/MMP2/MMP3/MMP9/NFE2L2/NOS3/NQO1/RELA/TNF/TP53	21
GO:0007568	aging	20/83	321/18670	8.67E-18	3.53E-15	1.24E-15	CYP1A1/MPO/PTGS2/BCL2/CAT/CDKN1A/CHEK1/FOS/ICAM1/IL10/MAPK1/MAPK14/NFE2L2/NQO1/RELA/SERPINE1/STAT3/TGFB1/TP53/VCAM1	20

GO:0051090	regulation of DNA-binding transcription factor activity	22/83	432/18670	1.15E-17	4.26E-15	1.49E-15	CHUK/CYP1B1/IKBKB/CD40LG/PPARG/CAT/ESR1/FOS/HMOX1/ICAM1/IL10/IL1B/IL6/MAPK1/MAPK14/NFKBIA/PRKCB/RELA/STAT3/TGFB1/TNF/VEGFA	22
GO:0071216	cellular response to biotic stimulus	18/83	236/18670	1.28E-17	4.33E-15	1.51E-15	NOS2/CCL2/CXCL10/CXCL8/GSTP1/ICAM1/IL10/IL1B/IL6/MAPK1/MAPK14/NFKBIA/NOS3/RELA/SERPINE1/TGFB1/TNF/TP53	18
GO:0048545	response to steroid hormone	21/83	385/18670	1.77E-17	5.15E-15	1.80E-15	NR1I2/PPARG/PTGS2/BCL2/CA2/CASP3/CCND1/CDKN1A/CLDN4/EGFR/ESR1/FOS/GSTP1/ICAM1/IL10/IL6/PPARA/RELA/SPP1/TGFB1/TNF	21
GO:0097237	cellular response to toxic substance	18/83	247/18670	2.88E-17	6.89E-15	2.41E-15	CYP1B1/ABCG2/MPO/PTGS1/PTGS2/CAT/DUOX2/GSTM1/GSTP1/HMOX1/IL10/IL6/KDR/NFE2L2/NOS3/NQO1/RELA/TNF	18
GO:0070482	response to oxygen levels	20/83	394/18670	4.61E-16	8.94E-14	3.13E-14	CYP1A1/NOS2/PPARG/PTGS2/BCL2/CASP3/CAT/CDKN1A/HMOX1/ICAM1/MMP2/NFE2L2/NPEPPS/PLAU/PPARA/SLC6A4/TGFB1/TP53/VCAM1/VEGFA	20

GO:0001819	positive regulation of cytokine production	21/83	464/18670	7.59E-16	1.41E-13	4.92E-14	CHUK/CYP1B1/CD40LG/IFNG/PTGS2/ADRA2A/HMOX1/IL10/IL1A/IL1B/IL2/IL4/IL6/IRF1/MAPK14/RELA/SERPINE1/STAT1/STAT3/TGFB1/TNF	21
GO:1901342	regulation of vasculature development	20/83	422/18670	1.72E-15	3.04E-13	1.06E-13	CYP1B1/XDH/PPARG/PTGS2/CXCL10/CXCL8/HMOX1/IL10/IL1A/IL1B/IL6/KDR/NFATC1/NFE2L2/NOS3/PRKCB/SERPINE1/STAT1/STAT3/VEGFA	20
GO:0035690	cellular response to drug	19/83	369/18670	2.13E-15	3.62E-13	1.27E-13	CYP1B1/NOS2/PTGS2/AHR/EGFR/HMOX1/ICAM1/IL10/IL1B/IL6/KDR/MAPK1/MMP3/NFE2L2/NQO1/RELA/TGFB1/TNF/TP53	19
GO:0033002	muscle cell proliferation	16/83	239/18670	7.59E-15	1.24E-12	4.33E-13	IFNG/PPARG/PTGS2/CDKN1A/EGFR/GSTP1/HMOX1/IL10/IL6/MAPK1/MAPK14/MMP2/MMP9/STAT1/STAT3/TNF	16
GO:0022407	regulation of cell-cell adhesion	19/83	403/18670	1.06E-14	1.66E-12	5.80E-13	CD40LG/IFNG/CASP3/CCL2/ICAM1/IGF2/IL10/IL1B/IL2/IL4/IL6/IRF1/MAPK14/PPARA/RELA/TGFB1/TNF/VCAM1/VEGFA	19
GO:0070661	leukocyte proliferation	17/83	298/18670	1.42E-14	2.14E-12	7.49E-13	CD40LG/AHR/BAX/BCL2/CASP3/CDKN1A/GSTP1/IGF2/IL10/IL1B/IL2/IL4/IL6/IRF1/TGFB1/TP53/VCAM1	17

GO:0010038	response to metal ion	18/83	364/18670	2.57E-14	3.38E-12	1.18E-12	CHUK/CYP1A1/PTGS2/BCL2/CA2/CASP3/CAT/CCND1/EGFR/FOS/HMOX1/ICAM1/IL1A/MAPK1/MMP9/NFE2L2/NQO1/VCAM1	18
GO:0034612	response to tumor necrosis factor	17/83	312/18670	3.02E-14	3.72E-12	1.30E-12	CHUK/IKBKB/CD40LG/PTGS2/CASP3/CCL2/CXCL8/GSTP1/ICAM1/MAPK1/MAPK14/NFE2L2/NFKBIA/RELA/STAT1/TNF/VCAM1	17
GO:0070663	regulation of leukocyte proliferation	15/83	222/18670	4.97E-14	5.79E-12	2.03E-12	CD40LG/AHR/BCL2/CASP3/CDKN1A/GSTP1/IGF2/IL10/IL1B/IL2/IL4/IL6/IRF1/TGFB1/VCAM1	15
GO:0097191	extrinsic apoptotic signaling pathway	15/83	224/18670	5.68E-14	6.30E-12	2.20E-12	IFNG/BAX/BCL2/CASP3/GSTP1/HMOX1/ICAM1/IL1A/IL1B/IL2/NOS3/RELA/SERPINE1/TGFB1/TNF	15
GO:0046651	lymphocyte proliferation	16/83	272/18670	5.72E-14	6.30E-12	2.20E-12	CD40LG/AHR/BAX/BCL2/CASP3/CDKN1A/IGF2/IL10/IL1B/IL2/IL4/IL6/IRF1/TGFB1/TP53/VCAM1	16
GO:1990748	cellular detoxification	12/83	112/18670	8.06E-14	8.01E-12	2.80E-12	ABCG2/MPO/PTGS1/PTGS2/CAT/DUOX2/GSTM1/GSTP1/NFE2L2/NOS3/NQO1/TNF	12
GO:0007159	leukocyte cell-cell adhesion	17/83	337/18670	1.06E-13	1.03E-11	3.60E-12	CD40LG/IFNG/CASP3/CCL2/ICAM1/IGF2/IL10/IL1B/IL2/IL4/IL6/IRF1/PPARA/RELA/TGFB1/TNF/VCAM1	17

GO:0071496	cellular response to external stimulus	17/83	339/18670	1.17E-13	1.11E-11	3.88E-12	PPARG/PTGS2/BCL2/CDKN1A/CHEK1/EGFR/FOS/GSTP1/HMOX1/ICAM1/IL1B/IRF1/MAPK1/NFE2L2/TGFB1/TP53/VCAM1	17
GO:0009410	response to xenobiotic stimulus	16/83	292/18670	1.72E-13	1.59E-11	5.56E-12	CYP1A1/CYP1B1/CYP2C9/CYP3A4/NR1I2/PPARG/PTGS1/AHR/EGFR/GSTM1/GSTP1/HTR3A/ICAM1/NQO1/RELA/TGFB1	16
GO:0050727	regulation of inflammatory response	19/83	485/18670	2.93E-13	2.60E-11	9.10E-12	NOS2/PPARG/PTGS2/EGFR/ESR1/GSTP1/IL10/IL1B/IL2/IL4/IL6/MAPK14/MMP3/MMP9/NFKBIA/PPARA/RELA/SERPINE1/TNF	19
GO:0050670	regulation of lymphocyte proliferation	14/83	208/18670	3.93E-13	3.27E-11	1.14E-11	CD40LG/AHR/BCL2/CASP3/CDKN1A/IGF2/IL10/IL1B/IL2/IL4/IL6/IRF1/TGFB1/VCAM1	14
GO:0009612	response to mechanical stimulus	14/83	210/18670	4.49E-13	3.58E-11	1.25E-11	MPO/PPARG/PTGS2/CHEK1/CXCL10/EGFR/FOS/IL1B/IRF1/MAPK14/NFKBIA/RELA/STAT1/TGFB1	14
GO:0048660	regulation of smooth muscle cell proliferation	13/83	169/18670	5.25E-13	4.11E-11	1.44E-11	IFNG/PPARG/PTGS2/CDKN1A/EGFR/GSTP1/HMOX1/IL10/IL6/MMP2/MMP9/STAT1/TNF	13
GO:0071466	cellular response to xenobiotic stimulus	13/83	180/18670	1.18E-12	8.59E-11	3.01E-11	CYP1A1/CYP1B1/CYP2C9/CYP3A4/NR1I2/PTGS1/AHR/EGFR/GSTM1/GSTP1/ICAM1/NQO1/TGFB1	13

GO:0042136	neurotransmitter biosynthetic process	11/83	106/18670	1.33E-12	9.54E-11	3.34E-11	CYP1B1/IFNG/NOS2/PTGS2/ICAM1/IL10/IL1B/NOS3/NQO1/SLC6A4/TNF	11
GO:0006809	nitric oxide biosynthetic process	10/83	77/18670	1.49E-12	1.04E-10	3.65E-11	CYP1B1/IFNG/NOS2/PTGS2/ICAM1/IL10/IL1B/NOS3/NQO1/TNF	10
GO:0097305	response to alcohol	14/83	233/18670	1.86E-12	1.20E-10	4.20E-11	PPARG/AHR/CAT/CCND1/CDKN1A/FOS/GSTP1/HTR3A/ICAM1/IL2/NQO1/STAT3/TGFB1/VCAM1	14
GO:0001101	response to acid chemical	16/83	343/18670	2.02E-12	1.28E-10	4.49E-11	CHUK/PPARG/PTGS2/CASP3/CAT/EGFR/GSTP1/ICAM1/KDR/MMP2/NQO1/PON1/RELA/SLC6A4/TNF/VEGFA	16
GO:1904019	epithelial cell apoptotic process	11/83	111/18670	2.23E-12	1.40E-10	4.90E-11	CD40LG/CCL2/HMOX1/ICAM1/IL10/IL4/IL6/KDR/NFE2L2/SERPINE1/TNF	11
GO:0071356	cellular response to tumor necrosis factor	15/83	291/18670	2.55E-12	1.58E-10	5.51E-11	CHUK/IKBKB/CD40LG/CCL2/CXCL8/GSTP1/ICAM1/MAPK1/MAPK14/NFE2L2/NFKBIA/RELA/STAT1/TNF/VCAM1	15
GO:0050731	positive regulation of peptidyl-tyrosine phosphorylation	13/83	192/18670	2.70E-12	1.64E-10	5.74E-11	IFNG/ADRA2A/EGF/ICAM1/IGF2/IL2/IL4/IL6/STAT3/TGFB1/TNF/TP53/VEGFA	13
GO:0042035	regulation of cytokine biosynthetic process	11/83	114/18670	3.00E-12	1.77E-10	6.20E-11	IFNG/HMOX1/IL10/IL1A/IL1B/IL4/IL6/IRF1/RELA/STAT3/TNF	11

GO:0062013	positive regulation of small molecule metabolic process	12/83	153/18670	3.48E-12	2.03E-10	7.09E-11	IFNG/NOS2/PPARG/PTGS2/EGF/IGF2/IL1B/IL4/NOS3/PPARA/STAT3/TNF	12
GO:2001057	reactive nitrogen species metabolic process	10/83	85/18670	4.12E-12	2.36E-10	8.27E-11	CYP1B1/IFNG/NOS2/PTGS2/ICAM1/IL10/IL1B/NOS3/NQO1/TNF	10
GO:1904035	regulation of epithelial cell apoptotic process	10/83	88/18670	5.88E-12	3.32E-10	1.16E-10	CD40LG/CCL2/HMOX1/ICAM1/IL4/IL6/KDR/NFE2L2/SERPINE1/TNF	10
GO:0048732	gland development	17/83	434/18670	6.18E-12	3.45E-10	1.21E-10	CYP1A1/XDH/BAX/BCL2/CCND1/EGF/EGFR/ESR1/FASN/HMOX1/IGF2/IL10/MAPK1/RELA/TGFB1/TNF/VEGFA	17
GO:0043434	response to peptide hormone	17/83	436/18670	6.65E-12	3.56E-10	1.25E-10	CHUK/PPARG/PTGS2/CA2/CAT/GSTP1/ICAM1/IGF2/IL10/IL1B/NFE2L2/PPARA/PRKCB/RELA/STAT1/STAT3/TGFB1	17
GO:0042089	cytokine biosynthetic process	11/83	123/18670	6.97E-12	3.69E-10	1.29E-10	IFNG/HMOX1/IL10/IL1A/IL1B/IL4/IL6/IRF1/RELA/STAT3/TNF	11
GO:0009314	response to radiation	17/83	448/18670	1.02E-11	5.27E-10	1.84E-10	PTGS2/BAX/BCL2/CASP3/CAT/CCND1/CDKN1A/CHEK1/CXCL10/EGFR/FOS/ICAM1/MAPK14/RELA/TGFB1/TP53/VCAM1	17
GO:0009615	response to virus	15/83	326/18670	1.29E-11	6.50E-10	2.28E-10	CHUK/CYP1A1/IKBKB/IFNG/BCL2/CXCL10/DUOX2/IL1B/IL6/IRF1/MAPK14/ODC1/RELA/STAT1/TNF	15

GO:0062012	regulation of small molecule metabolic process	17/83	459/18670	1.50E-11	7.31E-10	2.56E-10	IFNG/NOS2/PPARG/PTGS2/EGF/FASN/IGF2/IL1B/IL4/NOS3/NQO1/ODC1/PPARA/STAT3/TGFB1/TNF/TP53	17
GO:0044706	multi-multicellular organism process	13/83	222/18670	1.70E-11	8.13E-10	2.84E-10	CYP1A1/PTGS2/BCL2/CLDN4/ESR1/FOS/IL1B/MAPK1/MMP2/MMP9/SLC6A4/SPP1/TGFB1	13
GO:0061614	pri-miRNA transcription by RNA polymerase II	8/83	47/18670	2.95E-11	1.37E-09	4.78E-10	PPARG/FOS/IL10/PPARA/RELA/STAT3/TGFB1/TP53	8
GO:0030099	myeloid cell differentiation	16/83	416/18670	3.66E-11	1.64E-09	5.74E-10	IFNG/PPARG/CA2/CASP3/FASN/FOS/IL4/MAPK14/MMP9/NFKBIA/PRKCB/STAT1/STAT3/TGFB1/TNF/VEGFA	16
GO:0035094	response to nicotine	8/83	49/18670	4.20E-11	1.86E-09	6.51E-10	BCL2/CASP3/HMOX1/MAPK1/PPARA/RELA/TNF/VCAM1	8
GO:0150076	neuroinflammatory response	9/83	75/18670	4.30E-11	1.88E-09	6.59E-10	IFNG/PTGS2/EGFR/IL1B/IL4/IL6/MMP3/MMP9/TNF	9
GO:0018105	peptidyl-serine phosphorylation	14/83	299/18670	5.29E-11	2.25E-09	7.86E-10	CHUK/IKBKB/IFNG/PTGS2/BAX/BCL2/EGFR/IL6/MAPK1/MAPK14/PRKCB/TGFB1/TNF/VEGFA	14
GO:0050673	epithelial cell proliferation	16/83	434/18670	6.87E-11	2.74E-09	9.59E-10	XDH/PPARG/BAX/CCL2/CCND1/EGFR/ESR1/HMOX1/IL10/KDR/MAPK1/STAT1/STAT3/TGFB1/TNF/VEGFA	16

GO:0007259	JAK-STAT cascade	11/83	156/18670	9.35E-11	3.59E-09	1.26E-09	CYP1B1/IFNG/CCL2/EGF/IL10/IL2/IL4/IL6/STAT1/STAT3/TNF	11
GO:1904951	positive regulation of establishment of protein localization	16/83	456/18670	1.42E-10	5.37E-09	1.88E-09	CHUK/IFNG/PTGS2/BCL2/EGFR/IL10/IL1A/IL1B/IL2/IL6/MAPK1/MAPK14/NPEPPS/TGFB1/TNF/TP53	16
GO:0032103	positive regulation of response to external stimulus	14/83	323/18670	1.47E-10	5.49E-09	1.92E-09	PTGS2/CXCL10/CXCL8/EGFR/IL1B/IL2/IL6/KDR/MAPK14/NFKBIA/SERPINE1/TGFB1/TNF/VEGFA	14
GO:0010888	negative regulation of lipid storage	6/83	19/18670	1.66E-10	6.16E-09	2.16E-09	PPARG/CRP/IL6/NFKBIA/PPARA/TNF	6
GO:0008202	steroid metabolic process	14/83	331/18670	2.02E-10	7.17E-09	2.51E-09	CYP1A1/CYP1B1/CYP2C9/CYP3A4/IFNG/NR1I2/CAT/ESR1/FASN/IL1B/IL4/PON1/SPP1/TNF	14
GO:0048771	tissue remodeling	11/83	179/18670	4.10E-10	1.40E-08	4.91E-09	BAX/CA2/EGFR/IL1A/IL2/IL6/MMP2/NOS3/SPP1/TGFB1/TP53	11
GO:0150077	regulation of neuroinflammatory response	7/83	41/18670	5.28E-10	1.76E-08	6.16E-09	PTGS2/IL1B/IL4/IL6/MMP3/MMP9/TNF	7
GO:0002700	regulation of production of molecular mediator of immune response	10/83	139/18670	5.78E-10	1.91E-08	6.70E-09	CD40LG/HMOX1/IL10/IL1B/IL2/IL4/IL6/MAPK14/TGFB1/TNF	10

GO:0007565	female pregnancy	11/83	192/18670	8.65E-10	2.82E-08	9.86E-09	PTGS2/BCL2/CLDN4/ESR1/FOS/IL1B/MAPK1/MMP2/MMP9/SPP1/TGFB1	11
GO:0048872	homeostasis of number of cells	12/83	246/18670	8.87E-10	2.87E-08	1.00E-08	BAX/BCL2/CASP3/HMOX1/IL2/IL6/MAPK14/NOS3/STAT1/STAT3/TGFB1/VEGFA	12
GO:0009895	negative regulation of catabolic process	13/83	308/18670	9.79E-10	3.12E-08	1.09E-08	NOS2/ADRA2A/BCL2/EGFR/HMOX1/IL10/IL1B/MAPK14/PPARA/RELA/STAT3/TNF/TP53	13
GO:0002685	regulation of leukocyte migration	11/83	196/18670	1.08E-09	3.40E-08	1.19E-08	CCL2/CXCL10/CXCL8/HMOX1/ICAM1/IL6/MAPK14/SERPINE1/TGFB1/TNF/VEGFA	11
GO:0006631	fatty acid metabolic process	14/83	383/18670	1.35E-09	4.11E-08	1.44E-08	ALOX5/CYP1A1/CYP1B1/CYP2C9/CYP3A4/PPARG/PTGS1/PTGS2/FASN/GSTP1/IL1B/MAPK14/PON1/PPARA	14
GO:0006953	acute-phase response	7/83	47/18670	1.44E-09	4.33E-08	1.52E-08	PTGS2/CRP/IL1A/IL1B/IL6/STAT3/TNF	7
GO:1901653	cellular response to peptide	14/83	385/18670	1.45E-09	4.33E-08	1.52E-08	PPARG/CA2/GSTP1/ICAM1/IGF2/IL1B/NFE2L2/PRKCB/RELA/STAT1/STAT3/TGFB1/TP53/VCAM1	14
GO:0045787	positive regulation of cell cycle	14/83	389/18670	1.65E-09	4.88E-08	1.71E-08	CYP1A1/BAX/CCND1/CDKN1A/CHEK1/EGF/EGFR/IGF2/IL10/IL1A/IL1B/SLC6A4/TGFB1/TP53	14
GO:0030856	regulation of epithelial cell differentiation	10/83	156/18670	1.79E-09	5.21E-08	1.82E-08	IKBKB/XDH/IFNG/CCND1/IL1B/MMP9/SERPINE1/STAT1/TNF/VEGFA	10

GO:1905952	regulation of lipid localization	10/83	157/18670	1.90E-09	5.50E-08	1.93E-08	PPARG/CRP/EGF/IL1B/IL6/NFKBIA/PON1/PPARA/SPP1/TNF	10
GO:0050708	regulation of protein secretion	15/83	472/18670	2.22E-09	6.38E-08	2.23E-08	CHUK/CD40LG/IFNG/NOS2/ADRA2A/CRP/EGFR/IL10/IL1A/IL1B/IL2/IL6/MAPK14/TGFB1/TNF	15
GO:0031668	cellular response to extracellular stimulus	12/83	268/18670	2.35E-09	6.68E-08	2.34E-08	PPARG/PTGS2/BCL2/CDKN1A/FOS/GSTP1/HMOX1/ICAM1/MAPK1/NFE2L2/TP53/VCAM1	12
GO:0071241	cellular response to inorganic substance	11/83	217/18670	3.15E-09	8.78E-08	3.07E-08	CHUK/CYP1A1/PTGS2/EGFR/FOS/HMOX1/MAPK1/MMP3/MMP9/NFE2L2/NQO1	11
GO:0048871	multicellular organismal homeostasis	15/83	485/18670	3.21E-09	8.90E-08	3.12E-08	PTGS2/BAX/BCL2/CA2/CLDN4/EGFR/IL1A/IL1B/IL4/IL6/NOS3/SPP1/STAT3/TNF/VEGFA	15
GO:0048144	fibroblast proliferation	8/83	84/18670	3.58E-09	9.73E-08	3.40E-08	PPARG/BAX/CDKN1A/EGFR/ESR1/GSTP1/TGFB1/TP53	8
GO:0043062	extracellular structure organization	14/83	422/18670	4.69E-09	1.21E-07	4.23E-08	CYP1B1/MPO/ICAM1/IL6/KDR/MMP1/MMP2/MMP3/MMP9/SERPINE1/SPP1/TGFB1/TNF/VCAM1	14
GO:0050900	leukocyte migration	15/83	499/18670	4.72E-09	1.21E-07	4.23E-08	CCL2/CXCL10/CXCL8/HMOX1/ICAM1/IL10/IL1B/IL6/MAPK14/MMP1/SERPINE1/TGFB1/TNF/VCAM1/VEGFA	15

GO:0002791	regulation of peptide secretion	15/83	500/18670	4.85E-09	1.23E-07	4.32E-08	CHUK/CD40LG/IFNG/NOS2/ADRA2A/CRP/EGFR/IL10/IL1A/IL1B/IL2/IL6/MAPK14/TGFB1/TNF	15
GO:0003018	vascular process in circulatory system	10/83	173/18670	4.88E-09	1.23E-07	4.32E-08	PTGS2/ADRA2A/CRP/EGFR/HMOX1/ICAM1/NOS3/SLC6A4/TGFB1/VEGFA	10
GO:0034103	regulation of tissue remodeling	8/83	88/18670	5.21E-09	1.30E-07	4.55E-08	BAX/CA2/EGFR/IL2/IL6/SPP1/TGFB1/TP53	8
GO:0042445	hormone metabolic process	11/83	232/18670	6.33E-09	1.52E-07	5.31E-08	CYP1A1/CYP1B1/CYP2C9/CYP3A4/IFNG/DUOX2/ESR1/IL1B/PLB1/SPP1/TNF	11
GO:0048511	rhythmic process	12/83	295/18670	6.90E-09	1.60E-07	5.59E-08	NOS2/PPARG/AHR/CASP3/CLDN4/EGFR/ESR1/NOS3/PPARA/SERPINE1/SLC6A4/TP53	12
GO:0003158	endothelium development	9/83	132/18670	7.08E-09	1.63E-07	5.70E-08	IKBKB/XDH/CXCL10/FASN/ICAM1/IL1B/KDR/TNF/VEGFA	9
GO:0033619	membrane protein proteolysis	7/83	59/18670	7.51E-09	1.71E-07	5.97E-08	IFNG/ADRA2A/IL10/IL1B/RELA/TGFB1/TNF	7
GO:1905953	negative regulation of lipid localization	7/83	61/18670	9.53E-09	2.10E-07	7.34E-08	PPARG/CRP/EGF/IL6/NFKBIA/PPARA/TNF	7
GO:0051098	regulation of binding	13/83	373/18670	9.82E-09	2.13E-07	7.45E-08	IFNG/PPARG/BAX/BCL2/EGF/HMOX1/IL10/MMP9/NFKBIA/PON1/PPARA/SLPI/TGFB1	13

GO:0046824	positive regulation of nucleocytoplasmic transport	7/83	62/18670	1.07E-08	2.31E-07	8.07E-08	IFNG/PTGS2/IL1B/MAPK1/MAPK14/TGFB1/TP53	7
GO:0010573	vascular endothelial growth factor production	6/83	36/18670	1.12E-08	2.35E-07	8.22E-08	CYP1B1/PTGS2/IL1A/IL1B/IL6/TGFB1	6
GO:0010742	macrophage derived foam cell differentiation	6/83	36/18670	1.12E-08	2.35E-07	8.22E-08	PPARG/CRP/NFKBIA/PPARA/STAT1/TGFB1	6
GO:0090077	foam cell differentiation	6/83	36/18670	1.12E-08	2.35E-07	8.22E-08	PPARG/CRP/NFKBIA/PPARA/STAT1/TGFB1	6
GO:0006606	protein import into nucleus	9/83	143/18670	1.43E-08	2.85E-07	9.96E-08	IFNG/PTGS2/CDKN1A/MAPK1/MAPK14/NFKBIA/STAT3/TGFB1/TP53	9
GO:0002367	cytokine production involved in immune response	8/83	102/18670	1.69E-08	3.31E-07	1.16E-07	HMOX1/IL10/IL1B/IL4/IL6/MAPK14/TGFB1/TNF	8
GO:1903829	positive regulation of cellular protein localization	12/83	324/18670	1.96E-08	3.76E-07	1.32E-07	IFNG/PTGS2/BCL2/EGF/EGFR/IL1B/MAPK1/MAPK14/NPEPPS/TGFB1/TNF/TP53	12
GO:0051767	nitric-oxide synthase biosynthetic process	5/83	20/18670	2.26E-08	4.17E-07	1.46E-07	IFNG/CCL2/GSTP1/KDR/STAT1	5

GO:0051769	regulation of nitric-oxide synthase biosynthetic process	5/83	20/18670	2.26E-08	4.17E-07	1.46E-07	IFNG/CCL2/GSTP1/KDR/STAT1	5
GO:0061028	establishment of endothelial barrier	6/83	41/18670	2.55E-08	4.68E-07	1.64E-07	IKBKB/FASN/ICAM1/IL1B/TNF/VEGFA	6
GO:0045807	positive regulation of endocytosis	9/83	153/18670	2.58E-08	4.71E-07	1.65E-07	IFNG/PPARG/CCL2/EGF/IL1B/IL4/SERPINE1/TNF/VEGFA	9
GO:0050920	regulation of chemotaxis	10/83	217/18670	4.25E-08	7.27E-07	2.54E-07	CCL2/CXCL10/CXCL8/GSTP1/IL6/KDR/MAPK14/SERPINE1/TGFB1/VEGFA	10
GO:0051170	import into nucleus	9/83	163/18670	4.47E-08	7.61E-07	2.66E-07	IFNG/PTGS2/CDKN1A/MAPK1/MAPK14/NFKBIA/STAT3/TGFB1/TP53	9
GO:0051043	regulation of membrane protein ectodomain proteolysis	5/83	23/18670	4.86E-08	8.21E-07	2.87E-07	IFNG/ADRA2A/IL10/IL1B/TNF	5
GO:0001505	regulation of neurotransmitter levels	12/83	354/18670	5.19E-08	8.64E-07	3.02E-07	CYP1B1/IFNG/NOS2/PTGS2/ICAM1/IL10/IL1B/NOS3/NQO1/PRKCB/SLC6A4/TNF	12
GO:0051403	stress-activated MAPK cascade	11/83	286/18670	5.46E-08	9.01E-07	3.15E-07	CHUK/IKBKB/XDH/CD40LG/EGFR/GSTP1/IL1B/MAPK1/MAPK14/TNF/VEGFA	11
GO:1901568	fatty acid derivative metabolic process	9/83	167/18670	5.51E-08	9.05E-07	3.17E-07	ALOX5/CYP1A1/CYP1B1/CYP2C9/PTGS1/PTGS2/FASN/IL1B/PON1	9

GO:0043536	positive regulation of blood vessel endothelial cell migration	7/83	79/18670	5.94E-08	9.68E-07	3.39E-07	PTGS2/HMOX1/KDR/NFE2L2/NOS3/TGFB1/VEGFA	7
GO:0031331	positive regulation of cellular catabolic process	12/83	361/18670	6.42E-08	1.03E-06	3.62E-07	IFNG/ADRA2A/BAX/EGF/HMOX1/IL1B/IL4/IL6/KDR/NFE2L2/PPARA/TNF	12
GO:0032102	negative regulation of response to external stimulus	12/83	365/18670	7.24E-08	1.15E-06	4.02E-07	PPARG/CCL2/CDKN1A/GSTP1/IL10/IL2/IL4/NOS3/PLAU/PPARA/SERPINE1/SPP1	12
GO:0019430	removal of superoxide radicals	5/83	25/18670	7.61E-08	1.20E-06	4.19E-07	MPO/NFE2L2/NOS3/NQO1/TNF	5
GO:0060557	positive regulation of vitamin D biosynthetic process	3/83	3/18670	8.47E-08	1.31E-06	4.57E-07	IFNG/IL1B/TNF	3
GO:0060559	positive regulation of calcidiol 1-monooxygenase activity	3/83	3/18670	8.47E-08	1.31E-06	4.57E-07	IFNG/IL1B/TNF	3
GO:0002718	regulation of cytokine production involved in immune response	7/83	84/18670	9.12E-08	1.40E-06	4.90E-07	HMOX1/IL10/IL1B/IL6/MAPK14/TGFB1/TNF	7
GO:0032800	receptor biosynthetic process	5/83	26/18670	9.40E-08	1.44E-06	5.03E-07	IFNG/PPARG/IL10/PPARA/TNF	5

GO:1903034	regulation of response to wounding	9/83	179/18670	1.00E-07	1.52E-06	5.32E-07	ADRA2A/CDKN1A/DUOX2/IL10/NFE2L2/NOS3/PLAU/SERPINE1/SPP1	9
GO:0071346	cellular response to interferon-gamma	9/83	180/18670	1.05E-07	1.59E-06	5.56E-07	IFNG/NOS2/PPARG/CCL2/ICAM1/IRF1/STAT1/TP53/VCAM1	9
GO:0009266	response to temperature stimulus	10/83	243/18670	1.23E-07	1.82E-06	6.38E-07	PPARG/PTGS2/CDKN1A/CXCL10/FOS/HMOX1/IL1A/MAPK1/NFKBIA/NOS3	10
GO:0046683	response to organophosphorus	8/83	134/18670	1.43E-07	2.10E-06	7.34E-07	PTGS2/AHR/DUOX2/FOS/IL1B/RELA/SLC6A4/STAT1	8
GO:0010623	programmed cell death involved in cell development	4/83	12/18670	1.75E-07	2.48E-06	8.68E-07	BAX/BCL2/IL1A/IL1B	4
GO:0051924	regulation of calcium ion transport	10/83	254/18670	1.85E-07	2.60E-06	9.10E-07	PTGS2/ADRA2A/BAX/BCL2/CCL2/CXCL10/EGF/ICAM1/NOS3/TGFB1	10
GO:0035296	regulation of tube diameter	8/83	143/18670	2.36E-07	3.20E-06	1.12E-06	PTGS2/ADRA2A/CRP/EGFR/HMOX1/ICAM1/NOS3/SLC6A4	8
GO:0050880	regulation of blood vessel size	8/83	143/18670	2.36E-07	3.20E-06	1.12E-06	PTGS2/ADRA2A/CRP/EGFR/HMOX1/ICAM1/NOS3/SLC6A4	8
GO:0097746	regulation of blood vessel diameter	8/83	143/18670	2.36E-07	3.20E-06	1.12E-06	PTGS2/ADRA2A/CRP/EGFR/HMOX1/ICAM1/NOS3/SLC6A4	8
GO:0071214	cellular response to abiotic stimulus	11/83	331/18670	2.39E-07	3.21E-06	1.12E-06	PTGS2/BAX/CASP3/CDKN1A/CHEK1/EGFR/IL1B/IRF1/MAPK14/TGFB1/TP53	11

GO:0104004	cellular response to environmental stimulus	11/83	331/18670	2.39E-07	3.21E-06	1.12E-06	PTGS2/BAX/CASP3/CDKN1A/CHEK1/EGFR/IL1B/IRF1/MAPK14/TGFB1/TP53	11
GO:0045930	negative regulation of mitotic cell cycle	11/83	338/18670	2.94E-07	3.76E-06	1.32E-06	BAX/BCL2/CCL2/CCND1/CDKN1A/CHEK1/EGFR/IL10/TGFB1/TNF/TP53	11
GO:0031334	positive regulation of protein complex assembly	10/83	268/18670	3.04E-07	3.87E-06	1.36E-06	IFNG/BAX/ESR1/ICAM1/MMP1/MMP3/TGFB1/TNF/TP53/VEGFA	10
GO:0046136	positive regulation of vitamin metabolic process	3/83	4/18670	3.38E-07	4.23E-06	1.48E-06	IFNG/IL1B/TNF	3
GO:0009896	positive regulation of catabolic process	12/83	423/18670	3.55E-07	4.42E-06	1.55E-06	IFNG/ADRA2A/BAX/EGF/HMOX1/IL1B/IL4/IL6/KDR/NFE2L2/PPARA/TNF	12
GO:0002440	production of molecular mediator of immune response	10/83	286/18670	5.52E-07	6.46E-06	2.26E-06	CD40LG/HMOX1/IL10/IL1B/IL2/IL4/IL6/MAPK14/TGFB1/TNF	10
GO:0002819	regulation of adaptive immune response	8/83	160/18670	5.58E-07	6.52E-06	2.28E-06	IL10/IL1B/IL2/IL4/IL6/IRF1/TGFB1/TNF	8
GO:0035924	cellular response to vascular endothelial growth factor stimulus	6/83	68/18670	5.64E-07	6.55E-06	2.29E-06	XDH/KDR/MAPK14/RELA/VCAM1/VEGFA	6

GO:0002460	adaptive immune response based on somatic recombination of immune receptors built from immunoglobulin superfamily domains	11/83	361/18670	5.64E-07	6.55E-06	2.29E-06	CD40LG/CRP/ICAM1/IL10/IL1B/IL2/IL4/IL6/STAT3/TGFB1/TNF	11
GO:0034405	response to fluid shear stress	5/83	37/18670	5.99E-07	6.87E-06	2.41E-06	PTGS2/CA2/NFE2L2/NOS3/TGFB1	5
GO:0060965	negative regulation of gene silencing by miRNA	4/83	16/18670	6.35E-07	7.24E-06	2.53E-06	PPARG/ESR1/TGFB1/TP53	4
GO:0032963	collagen metabolic process	7/83	115/18670	7.90E-07	8.76E-06	3.07E-06	PPARG/IL6/MMP1/MMP2/MMP3/MMP9/TGFB1	7
GO:0014805	smooth muscle adaptation	3/83	5/18670	8.42E-07	9.19E-06	3.22E-06	HMOX1/IL1B/NOS3	3
GO:1900371	regulation of purine nucleotide biosynthetic process	7/83	117/18670	8.87E-07	9.61E-06	3.36E-06	IFNG/NOS2/IL4/NOS3/PPARA/STAT3/TP53	7
GO:0014015	positive regulation of gliogenesis	6/83	74/18670	9.35E-07	1.01E-05	3.53E-06	PPARG/IL1B/IL6/RELA/TGFB1/TNF	6
GO:0042176	regulation of protein catabolic process	11/83	381/18670	9.59E-07	1.03E-05	3.60E-06	IFNG/NOS2/ADRA2A/EGF/EGFR/IL10/IL1B/NFE2L2/ODC1/RELA/TNF	11

GO:0071453	cellular response to oxygen levels	9/83	234/18670	9.61E-07	1.03E-05	3.60E-06	PPARG/PTGS2/BCL2/HMOX1/ICAM1/NFE2L2/NPEPPS/TP53/VEGFA	9
GO:0050663	cytokine secretion	9/83	240/18670	1.19E-06	1.23E-05	4.31E-06	CHUK/IFNG/NOS2/CRP/IL10/IL1A/IL1B/MAPK14/TNF	9
GO:0006775	fat-soluble vitamin metabolic process	5/83	43/18670	1.30E-06	1.33E-05	4.66E-06	CYP1A1/CYP3A4/IFNG/IL1B/TNF	5
GO:0060149	negative regulation of posttranscriptional gene silencing	4/83	19/18670	1.34E-06	1.36E-05	4.74E-06	PPARG/ESR1/TGFB1/TP53	4
GO:0060967	negative regulation of gene silencing by RNA	4/83	19/18670	1.34E-06	1.36E-05	4.74E-06	PPARG/ESR1/TGFB1/TP53	4
GO:0040013	negative regulation of locomotion	11/83	396/18670	1.40E-06	1.41E-05	4.92E-06	CYP1B1/PPARG/BCL2/CCL2/GSTP1/HMOX1/IL4/NFE2L2/SERPINE1/STAT3/TGFB1	11
GO:0010876	lipid localization	11/83	400/18670	1.54E-06	1.52E-05	5.31E-06	NOS2/PPARG/CRP/EGF/IL1B/IL6/NFKBIA/PON1/PPARA/SPP1/TNF	11
GO:0032368	regulation of lipid transport	7/83	127/18670	1.54E-06	1.52E-05	5.31E-06	PPARG/EGF/IL1B/NFKBIA/PON1/PPARA/SPP1	7
GO:2000045	regulation of G1/S transition of mitotic cell cycle	8/83	184/18670	1.60E-06	1.58E-05	5.51E-06	CYP1A1/BAX/BCL2/CCL2/CCND1/CDKN1A/EGFR/TP53	8
GO:0006970	response to osmotic stress	6/83	83/18670	1.85E-06	1.76E-05	6.18E-06	PTGS2/BAX/CASP3/EGFR/TNF/TP53	6

GO:0097756	negative regulation of blood vessel diameter	6/83	84/18670	1.98E-06	1.88E-05	6.58E-06	PTGS2/ADRA2A/CRP/EGFR/ICAM1/SLC6A4	6
GO:0002204	somatic recombination of immunoglobulin genes involved in immune response	5/83	47/18670	2.04E-06	1.91E-05	6.70E-06	CD40LG/IL10/IL2/IL4/TGFB1	5
GO:0002208	somatic diversification of immunoglobulins involved in immune response	5/83	47/18670	2.04E-06	1.91E-05	6.70E-06	CD40LG/IL10/IL2/IL4/TGFB1	5
GO:0045190	isotype switching	5/83	47/18670	2.04E-06	1.91E-05	6.70E-06	CD40LG/IL10/IL2/IL4/TGFB1	5
GO:0060759	regulation of response to cytokine stimulus	8/83	190/18670	2.04E-06	1.91E-05	6.70E-06	CHUK/IKBKB/IFNG/PPARG/GSTP1/IL6/STAT1/TNF	8
GO:0007596	blood coagulation	10/83	336/18670	2.37E-06	2.18E-05	7.64E-06	CD40LG/ADRA2A/IL6/IRF1/MAPK1/NFE2L2/NOS3/PLAU/PRKCB/SERPINE1	10
GO:0051781	positive regulation of cell division	6/83	87/18670	2.43E-06	2.23E-05	7.82E-06	CAT/IGF2/IL1A/IL1B/TGFB1/VEGFA	6
GO:0034504	protein localization to nucleus	9/83	262/18670	2.45E-06	2.24E-05	7.83E-06	IFNG/PTGS2/CDKN1A/MAPK1/MAPK14/NFKBIA/STAT3/TGFB1/TP53	9
GO:0007599	hemostasis	10/83	341/18670	2.70E-06	2.43E-05	8.52E-06	CD40LG/ADRA2A/IL6/IRF1/MAPK1/NFE2L2/NOS3/PLAU/PRKCB/SERPINE1	10

GO:0031622	positive regulation of fever generation	3/83	7/18670	2.93E-06	2.60E-05	9.09E-06	PTGS2/IL1B/TNF	3
GO:0002889	regulation of immunoglobulin mediated immune response	5/83	51/18670	3.08E-06	2.69E-05	9.43E-06	IL10/IL2/IL4/TGFB1/TNF	5
GO:0007566	embryo implantation	5/83	51/18670	3.08E-06	2.69E-05	9.43E-06	PTGS2/IL1B/MMP2/MMP9/SPP1	5
GO:0048638	regulation of developmental growth	10/83	347/18670	3.16E-06	2.75E-05	9.63E-06	BCL2/CDKN1A/IGF2/MAPK1/MAPK14/PPARA/SLC6A4/SPP1/STAT3/VEGFA	10
GO:0034764	positive regulation of transmembrane transport	8/83	204/18670	3.46E-06	2.98E-05	1.04E-05	IFNG/BAX/CA2/CCL2/CXCL10/HTR3A/MAPK14/NFE2L2	8
GO:0002449	lymphocyte mediated immunity	10/83	352/18670	3.58E-06	3.07E-05	1.07E-05	CD40LG/CRP/ICAM1/IL10/IL1B/IL2/IL4/IL6/TGFB1/TNF	10
GO:1901655	cellular response to ketone	6/83	93/18670	3.60E-06	3.07E-05	1.07E-05	PPARG/AHR/EGFR/ICAM1/SPP1/TGFB1	6
GO:0045840	positive regulation of mitotic nuclear division	5/83	53/18670	3.73E-06	3.16E-05	1.11E-05	EGF/IGF2/IL1A/IL1B/TGFB1	5
GO:0006959	humoral immune response	10/83	356/18670	3.96E-06	3.31E-05	1.16E-05	IFNG/BCL2/CCL2/CRP/CXCL10/CXCL8/IL1B/IL6/SLPI/TNF	10
GO:0002246	wound healing involved in inflammatory response	3/83	8/18670	4.67E-06	3.77E-05	1.32E-05	HMOX1/IL1A/TGFB1	3

GO:0016999	antibiotic metabolic process	7/83	151/18670	4.89E-06	3.93E-05	1.37E-05	CYP1A1/MPO/CAT/DUOX2/EGFR/MMP3/STAT3	7
GO:0052547	regulation of peptidase activity	11/83	452/18670	4.99E-06	3.98E-05	1.39E-05	XDH/PPARG/PTGS2/BAX/MAPK14/MMP9/SERPINE1/SLPI/STAT3/TNF/VEGFA	11
GO:0061900	glial cell activation	5/83	58/18670	5.85E-06	4.53E-05	1.59E-05	IFNG/EGFR/IL1B/IL6/TNF	5
GO:0035902	response to immobilization stress	4/83	27/18670	5.89E-06	4.56E-05	1.59E-05	CYP1A1/PPARG/FOS/TGFB1	4
GO:0042326	negative regulation of phosphorylation	11/83	468/18670	6.94E-06	5.24E-05	1.83E-05	XDH/IFNG/BAX/CASP3/CDKN1A/GSTP1/IL1B/IL2/PPARA/STAT3/TGFB1	11
GO:0032768	regulation of monooxygenase activity	5/83	64/18670	9.54E-06	6.93E-05	2.42E-05	IFNG/EGFR/IL1B/NOS3/TNF	5
GO:0051607	defense response to virus	8/83	238/18670	1.07E-05	7.67E-05	2.69E-05	IFNG/BCL2/CXCL10/IL1B/IL6/IRF1/RELA/STAT1	8
GO:0008210	estrogen metabolic process	4/83	32/18670	1.19E-05	8.40E-05	2.94E-05	CYP1A1/CYP1B1/CYP2C9/CYP3A4	4
GO:0010822	positive regulation of mitochondrion organization	6/83	117/18670	1.36E-05	9.44E-05	3.30E-05	BAX/BCL2/KDR/MMP9/NPEPPS/TP53	6
GO:0046886	positive regulation of hormone biosynthetic process	3/83	11/18670	1.36E-05	9.44E-05	3.30E-05	IFNG/IL1B/TNF	3
GO:0051188	cofactor biosynthetic process	9/83	326/18670	1.43E-05	9.88E-05	3.46E-05	CYP1A1/IFNG/PTGS2/DUOX2/FASN/NFE2L2/PPARA/STAT3/TP53	9

GO:0045926	negative regulation of growth	8/83	249/18670	1.49E-05	0.0001023	3.58E-05	PPARG/BCL2/CDKN1A/PPARA/SLC6A4/SPP1/TGFB1/TP53	8
GO:0071383	cellular response to steroid hormone stimulus	8/83	250/18670	1.53E-05	0.0001046	3.66E-05	NR1I2/PPARG/EGFR/ESR1/GSTP1/ICAM1/PPARA/TGFB1	8
GO:0001558	regulation of cell growth	10/83	416/18670	1.54E-05	0.0001052	3.68E-05	PPARG/BCL2/CDKN1A/EGFR/IL2/PPARA/SPP1/TGFB1/TP53/VEGFA	10
GO:0061138	morphogenesis of a branching epithelium	7/83	182/18670	1.66E-05	0.0001118	3.91E-05	BCL2/EGF/ESR1/IL10/TGFB1/TNF/VEGFA	7
GO:1905477	positive regulation of protein localization to membrane	6/83	122/18670	1.72E-05	0.0001152	4.03E-05	IFNG/BCL2/EGFR/TGFB1/TNF/TP53	6
GO:0002538	arachidonic acid metabolite production involved in inflammatory response	2/83	2/18670	1.95E-05	0.0001277	4.47E-05	ALOX5/SERPINE1	2
GO:0002540	leukotriene production involved in inflammatory response	2/83	2/18670	1.95E-05	0.0001277	4.47E-05	ALOX5/SERPINE1	2

GO:0072363	regulation of glycolytic process by positive regulation of transcription from RNA polymerase II promoter	2/83	2/18670	1.95E-05	0.0001277	4.47E-05	PPARA/TP53	2
GO:0050804	modulation of chemical synaptic transmission	10/83	436/18670	2.31E-05	0.0001473	5.16E-05	PTGS2/CA2/CCL2/EGFR/IL1B/MAPK1/PRKCB/SLC6A4/STAT3/TNF	10
GO:0099177	regulation of trans-synaptic signaling	10/83	437/18670	2.36E-05	0.0001488	5.21E-05	PTGS2/CA2/CCL2/EGFR/IL1B/MAPK1/PRKCB/SLC6A4/STAT3/TNF	10
GO:0010827	regulation of glucose transmembrane transport	5/83	78/18670	2.52E-05	0.0001574	5.51E-05	IL1B/MAPK14/NFE2L2/PRKCB/TNF	5
GO:0007548	sex differentiation	8/83	270/18670	2.66E-05	0.0001657	5.80E-05	BAX/BCL2/CASP3/CCND1/ESR1/ICAM1/NOS3/VEGFA	8
GO:0030213	hyaluronan biosynthetic process	3/83	14/18670	2.98E-05	0.0001823	6.38E-05	EGF/IL1B/TGFB1	3
GO:0042572	retinol metabolic process	4/83	41/18670	3.24E-05	0.0001975	6.91E-05	CYP1A1/CYP1B1/CYP3A4/PLB1	4
GO:0000082	G1/S transition of mitotic cell cycle	8/83	279/18670	3.37E-05	0.0002043	7.15E-05	CYP1A1/BAX/BCL2/CCL2/CCND1/CDKN1A/EGFR/TP53	8
GO:0006109	regulation of carbohydrate metabolic process	7/83	206/18670	3.67E-05	0.0002205	7.72E-05	IFNG/EGF/IGF2/PPARA/STAT3/TGFB1/TP53	7

GO:0032409	regulation of transporter activity	8/83	283/18670	3.72E-05	0.0002211	7.74E-05	IFNG/PPARG/ADRA2A/BCL2/CCL2/HTR3A/MMP9/PON1	8
GO:0071229	cellular response to acid chemical	7/83	209/18670	4.03E-05	0.0002373	8.30E-05	PPARG/EGFR/KDR/MMP2/SLC6A4/TNF/VEGFA	7
GO:0031056	regulation of histone modification	6/83	143/18670	4.23E-05	0.0002473	8.65E-05	CHEK1/IGF2/IL1B/TGFB1/TP53/VEGFA	6
GO:1902004	positive regulation of amyloid-beta formation	3/83	16/18670	4.55E-05	0.0002629	9.20E-05	IFNG/CASP3/TNF	3
GO:0000077	DNA damage checkpoint	6/83	145/18670	4.57E-05	0.0002635	9.22E-05	BAX/CCND1/CDKN1A/CHEK1/MAPK14/TP53	6
GO:0000075	cell cycle checkpoint	7/83	216/18670	4.96E-05	0.0002847	9.96E-05	BAX/CCND1/CDKN1A/CHEK1/MAPK14/TGFB1/TP53	7
GO:0050777	negative regulation of immune response	6/83	150/18670	5.52E-05	0.0003096	0.00011	PPARG/HMOX1/IL10/IL2/TGFB1/TNF	6
GO:0016049	cell growth	10/83	484/18670	5.59E-05	0.0003128	0.00011	PPARG/BCL2/CDKN1A/EGFR/IL2/PPARA/SPP1/TGFB1/TP53/VEGFA	10
GO:0033590	response to cobalamin	2/83	3/18670	5.84E-05	0.0003215	0.00011	EGFR/RELA	2
GO:0035633	maintenance of permeability of blood-brain barrier	2/83	3/18670	5.84E-05	0.0003215	0.00011	PTGS2/IL6	2

GO:0098679	regulation of carbohydrate catabolic process by regulation of transcription from RNA polymerase II promoter	2/83	3/18670	5.84E-05	0.0003215	0.00011	PPARA/TP53	2
GO:2000097	regulation of smooth muscle cell-matrix adhesion	2/83	3/18670	5.84E-05	0.0003215	0.00011	PLAU/SERPINE1	2
GO:0034250	positive regulation of cellular amide metabolic process	6/83	153/18670	6.16E-05	0.0003355	0.00012	IFNG/CASP3/IL6/MAPK1/NFE2L2/TNF	6
GO:0150078	positive regulation of neuroinflammatory response	3/83	18/18670	6.59E-05	0.0003554	0.00012	IL1B/IL6/TNF	3
GO:1900221	regulation of amyloid-beta clearance	3/83	18/18670	6.59E-05	0.0003554	0.00012	IFNG/IL4/TNF	3
GO:0051205	protein insertion into membrane	4/83	49/18670	6.61E-05	0.0003555	0.00012	BAX/BCL2/EGFR/TP53	4
GO:0042119	neutrophil activation	10/83	498/18670	7.09E-05	0.0003782	0.00013	ALOX5/MPO/CAT/CXCL8/GSTP1/MAPK1/MAPK14/MMP9/PLAU/SLPI	10
GO:0002446	neutrophil mediated immunity	10/83	499/18670	7.20E-05	0.0003826	0.00013	ALOX5/MPO/CAT/GSTP1/IL6/MAPK1/MAPK14/MMP9/PLAU/SLPI	10

GO:0046879	hormone secretion	8/83	312/18670	7.40E-05	0.0003922	0.00014	IFNG/NOS2/ADRA2A/EGFR/IL1B/IL6/SPP1/TNF	8
GO:0043900	regulation of multi-organism process	9/83	405/18670	7.75E-05	0.0004072	0.00014	IFNG/NOS2/BCL2/CXCL8/IL1B/PLB1/SLPI/STAT1/TNF	9
GO:1902993	positive regulation of amyloid precursor protein catabolic process	3/83	20/18670	9.14E-05	0.0004691	0.00016	IFNG/CASP3/TNF	3
GO:0009914	hormone transport	8/83	322/18670	9.21E-05	0.0004713	0.00016	IFNG/NOS2/ADRA2A/EGFR/IL1B/IL6/SPP1/TNF	8
GO:0036499	PERK-mediated unfolded protein response	3/83	21/18670	0.00011	0.0005317	0.00019	CCL2/CXCL8/NFE2L2	3
GO:0071498	cellular response to fluid shear stress	3/83	21/18670	0.00011	0.0005317	0.00019	PTGS2/CA2/NFE2L2	3
GO:0038095	Fc-epsilon receptor signaling pathway	6/83	169/18670	0.00011	0.0005317	0.00019	CHUK/IKBKB/FOS/MAPK1/NFATC1/RELA	6
GO:0042742	defense response to bacterium	8/83	330/18670	0.00011	0.0005428	0.00019	MPO/NOS2/CRP/IL10/IL6/SERPINE1/SLPI/TNF	8
GO:0006808	regulation of nitrogen utilization	2/83	4/18670	0.00012	0.0005723	0.0002	BAX/BCL2	2
GO:1903347	negative regulation of bicellular tight junction assembly	2/83	4/18670	0.00012	0.0005723	0.0002	IKBKB/TNF	2

GO:1904659	glucose transmembrane transport	5/83	108/18670	0.00012	0.0005861	0.00021	IL1B/MAPK14/NFE2L2/PRKCB/TNF	5
GO:0010869	regulation of receptor biosynthetic process	3/83	22/18670	0.00012	0.0005987	0.00021	IFNG/PPARG/PPARA	3
GO:1905330	regulation of morphogenesis of an epithelium	6/83	180/18670	0.00015	0.0007184	0.00025	CXCL10/ESR1/STAT1/TGFB1/TNF/VEGFA	6
GO:0045088	regulation of innate immune response	9/83	452/18670	0.00018	0.0008149	0.00029	CHUK/IKBKB/IFNG/PPARG/ESR1/IRF1/NFKBIA/RELA/STAT1	9
GO:0009404	toxin metabolic process	3/83	25/18670	0.00018	0.0008263	0.00029	CYP1A1/CYP1B1/NFE2L2	3
GO:1905475	regulation of protein localization to membrane	6/83	187/18670	0.00019	0.0008417	0.00029	IFNG/BCL2/EGFR/TGFB1/TNF/TP53	6
GO:0019740	nitrogen utilization	2/83	5/18670	0.00019	0.0008589	0.0003	BAX/BCL2	2
GO:0022614	membrane to membrane docking	2/83	5/18670	0.00019	0.0008589	0.0003	ICAM1/VCAM1	2
GO:0032847	regulation of cellular pH reduction	2/83	5/18670	0.00019	0.0008589	0.0003	BCL2/CA2	2
GO:0033591	response to L-ascorbic acid	2/83	5/18670	0.00019	0.0008589	0.0003	CAT/GSTP1	2

GO:0035509	negative regulation of myosin-light-chain-phosphatase activity	2/83	5/18670	0.00019	0.0008589	0.0003	IKBKB/TNF	2
GO:0061302	smooth muscle cell-matrix adhesion	2/83	5/18670	0.00019	0.0008589	0.0003	PLAU/SERPINE1	2
GO:0045927	positive regulation of growth	7/83	270/18670	0.0002	0.0008818	0.00031	BCL2/EGFR/IGF2/IL2/MAPK1/MAPK14/VEGFA	7
GO:0051187	cofactor catabolic process	4/83	65/18670	0.0002	0.0008836	0.00031	MPO/CAT/DUOX2/HMOX1	4
GO:0019079	viral genome replication	5/83	122/18670	0.00021	0.0009244	0.00032	BCL2/CCL2/CXCL8/SLPI/TNF	5
GO:0150063	visual system development	8/83	366/18670	0.00022	0.0009622	0.00034	CYP1A1/CYP1B1/BAX/BCL2/EGFR/STAT3/TGFB1/VEGFA	8
GO:0051346	negative regulation of hydrolase activity	9/83	466/18670	0.00022	0.0009668	0.00034	IKBKB/PTGS2/MMP9/NOS3/SERPINE1/SLPI/TNF/TP53/VEGFA	9
GO:0019748	secondary metabolic process	4/83	67/18670	0.00022	0.0009746	0.00034	CYP1A1/CYP1B1/BCL2/NFE2L2	4
GO:0006909	phagocytosis	8/83	369/18670	0.00023	0.0010103	0.00035	IFNG/PPARG/CCL2/CRP/IL1B/MAPK1/TGFB1/TNF	8
GO:0033627	cell adhesion mediated by integrin	4/83	68/18670	0.00024	0.0010233	0.00036	CYP1B1/ICAM1/PLAU/SERPINE1	4
GO:0050918	positive chemotaxis	4/83	68/18670	0.00024	0.0010233	0.00036	CXCL10/CXCL8/KDR/VEGFA	4
GO:0042698	ovulation cycle	4/83	69/18670	0.00025	0.0010735	0.00038	CASP3/EGFR/ESR1/NOS3	4

GO:1902932	positive regulation of alcohol biosynthetic process	3/83	28/18670	0.00026	0.0010766	0.00038	IFNG/IL1B/TNF	3
GO:0002576	platelet degranulation	5/83	128/18670	0.00027	0.0011121	0.00039	EGF/IGF2/SERPINE1/TGFB1/VEGFA	5
GO:0030278	regulation of ossification	6/83	203/18670	0.00029	0.00119	0.00042	BCL2/IL6/MAPK1/MAPK14/TGFB1/TNF	6
GO:0035265	organ growth	6/83	204/18670	0.0003	0.0012176	0.00043	BCL2/ESR1/MAPK1/MAPK14/PPARA/SLC6A4	6
GO:0043312	neutrophil degranulation	9/83	485/18670	0.0003	0.0012262	0.00043	ALOX5/MPO/CAT/GSTP1/MAPK1/MAPK14/MMP9/PLAU/SLPI	9
GO:0002283	neutrophil activation involved in immune response	9/83	488/18670	0.00031	0.0012739	0.00045	ALOX5/MPO/CAT/GSTP1/MAPK1/MAPK14/MMP9/PLAU/SLPI	9
GO:0050890	cognition	7/83	296/18670	0.00035	0.0013886	0.00049	PTGS2/CASP3/EGFR/FOS/MAPK1/SLC6A4/TNF	7
GO:0006914	autophagy	9/83	496/18670	0.00035	0.0014038	0.00049	IFNG/BCL2/CASP3/HMOX1/IL10/IL4/KDR/STAT3/TP53	9
GO:0061919	process utilizing autophagic mechanism	9/83	496/18670	0.00035	0.0014038	0.00049	IFNG/BCL2/CASP3/HMOX1/IL10/IL4/KDR/STAT3/TP53	9
GO:0045454	cell redox homeostasis	4/83	76/18670	0.00037	0.0014471	0.00051	MPO/NOS2/NFE2L2/NOS3	4
GO:0046390	ribose phosphate biosynthetic process	7/83	300/18670	0.00038	0.0014919	0.00052	IFNG/FASN/IL4/PPARA/STAT3/TGFB1/TP53	7

GO:0042744	hydrogen peroxide catabolic process	3/83	32/18670	0.00038	0.0015027	0.00053	MPO/CAT/DUOX2	3
GO:0046685	response to arsenic-containing substance	3/83	32/18670	0.00038	0.0015027	0.00053	CYP1A1/CDKN1A/HMOX1	3
GO:0034620	cellular response to unfolded protein	5/83	140/18670	0.0004	0.0015604	0.00055	BAX/CCL2/CCND1/CXCL8/NFE2L2	5
GO:0038089	positive regulation of cell migration by vascular endothelial growth factor signaling pathway	2/83	7/18670	0.0004	0.0015604	0.00055	KDR/VEGFA	2
GO:0001570	vasculogenesis	4/83	79/18670	0.00042	0.0016194	0.00057	XDH/KDR/TGFB1/VEGFA	4
GO:0030193	regulation of blood coagulation	4/83	79/18670	0.00042	0.0016194	0.00057	NFE2L2/NOS3/PLAU/SERPINE1	4
GO:1900046	regulation of hemostasis	4/83	80/18670	0.00044	0.0016957	0.00059	NFE2L2/NOS3/PLAU/SERPINE1	4
GO:0034205	amyloid-beta formation	3/83	34/18670	0.00046	0.0017422	0.00061	IFNG/CASP3/TNF	3
GO:1905954	positive regulation of lipid localization	4/83	82/18670	0.00049	0.0018403	0.00064	IL1B/NFKBIA/PON1/SPP1	4
GO:0110111	negative regulation of animal organ morphogenesis	3/83	35/18670	0.0005	0.0018673	0.00065	BCL2/STAT1/TNF	3

GO:0061351	neural precursor cell proliferation	5/83	147/18670	0.0005	0.0018673	0.00065	EGF/SLC6A4/TGFB1/TP53/VEGFA	5
GO:0050818	regulation of coagulation	4/83	84/18670	0.00053	0.0019611	0.00069	NFE2L2/NOS3/PLAU/SERPINE1	4
GO:1900125	regulation of hyaluronan biosynthetic process	2/83	8/18670	0.00054	0.0019611	0.00069	EGF/TGFB1	2
GO:0001942	hair follicle development	4/83	86/18670	0.00058	0.0021032	0.00074	BCL2/EGFR/RELA/TNF	4
GO:0070542	response to fatty acid	4/83	86/18670	0.00058	0.0021032	0.00074	PPARG/PTGS2/CAT/PON1	4
GO:0090559	regulation of membrane permeability	4/83	86/18670	0.00058	0.0021032	0.00074	BAX/BCL2/STAT3/TP53	4
GO:0030212	hyaluronan metabolic process	3/83	37/18670	0.00059	0.0021088	0.00074	EGF/IL1B/TGFB1	3
GO:1905314	semi-lunar valve development	3/83	37/18670	0.00059	0.0021088	0.00074	NFATC1/NOS3/TGFB1	3
GO:1901861	regulation of muscle tissue development	5/83	155/18670	0.00064	0.0022449	0.00079	BCL2/MAPK1/MAPK14/PPARA/TGFB1	5
GO:1900407	regulation of cellular response to oxidative stress	4/83	88/18670	0.00064	0.0022449	0.00079	IL10/MMP3/NFE2L2/TNF	4
GO:1901863	positive regulation of muscle tissue development	4/83	88/18670	0.00064	0.0022449	0.00079	BCL2/MAPK1/MAPK14/TGFB1	4

GO:0031348	negative regulation of defense response	6/83	239/18670	0.00069	0.0023871	0.00084	PPARG/GSTP1/IL10/IL2/IL4/PPARA	6
GO:0048087	positive regulation of developmental pigmentation	2/83	9/18670	0.00069	0.0023871	0.00084	BAX/BCL2	2
GO:0098926	postsynaptic signal transduction	2/83	9/18670	0.00069	0.0023871	0.00084	RELA/STAT3	2
GO:0046434	organophosphate catabolic process	6/83	248/18670	0.00083	0.0028167	0.00099	XDH/IFNG/PON1/PPARA/STAT3/TP53	6
GO:0045069	regulation of viral genome replication	4/83	95/18670	0.00085	0.0028524	0.001	BCL2/CXCL8/SLPI/TNF	4
GO:1901031	regulation of response to reactive oxygen species	3/83	42/18670	0.00086	0.0028524	0.001	IL10/NFE2L2/TNF	3
GO:0021936	regulation of cerebellar granule cell precursor proliferation	2/83	10/18670	0.00086	0.0028524	0.001	EGF/SLC6A4	2
GO:0097327	response to antineoplastic agent	4/83	96/18670	0.00088	0.0029278	0.00102	EGFR/HMOX1/ICAM1/TGFB1	4
GO:0031341	regulation of cell killing	4/83	98/18670	0.00096	0.0031168	0.00109	IFNG/NOS2/ICAM1/IL4	4
GO:0007611	learning or memory	6/83	256/18670	0.00098	0.0031948	0.00112	PTGS2/CASP3/EGFR/FOS/MAPK1/SLC6A4	6

GO:0016241	regulation of macroautophagy	5/83	171/18670	0.00099	0.0032194	0.00113	CASP3/HMOX1/IL4/KDR/TP53	5
GO:0010522	regulation of calcium ion transport into cytosol	4/83	102/18670	0.00111	0.0035218	0.00123	BAX/BCL2/CXCL10/TGFB1	4
GO:0006986	response to unfolded protein	5/83	176/18670	0.00113	0.0035589	0.00125	BAX/CCL2/CCND1/CXCL8/NFE2L2	5
GO:0140014	mitotic nuclear division	6/83	264/18670	0.00115	0.0036124	0.00126	CHEK1/EGF/IGF2/IL1A/IL1B/TGFB1	6
GO:1905269	positive regulation of chromatin organization	4/83	103/18670	0.00115	0.0036124	0.00126	IL1B/TGFB1/TP53/VEGFA	4
GO:0045861	negative regulation of proteolysis	7/83	363/18670	0.00116	0.0036421	0.00127	PTGS2/IL10/MMP9/SERPINE1/SLPI/TP53/VEGFA	7
GO:0019233	sensory perception of pain	4/83	104/18670	0.00119	0.0037121	0.0013	PTGS2/CCL2/IL10/MAPK1	4
GO:0098732	macromolecule deacylation	4/83	104/18670	0.00119	0.0037121	0.0013	IFNG/TGFB1/TP53/VEGFA	4
GO:0070849	response to epidermal growth factor	3/83	47/18670	0.00119	0.0037121	0.0013	EGFR/GSTP1/MAPK1	3
GO:0006527	arginine catabolic process	2/83	12/18670	0.00125	0.0038603	0.00135	NOS2/NOS3	2
GO:0002269	leukocyte activation involved in inflammatory response	3/83	48/18670	0.00127	0.0038846	0.00136	IFNG/IL6/TNF	3

GO:0007595	lactation	3/83	48/18670	0.00127	0.0038846	0.00136	XDH/CCND1/VEGFA	3
GO:0101023	vascular endothelial cell proliferation	3/83	48/18670	0.00127	0.0038846	0.00136	PPARG/CCL2/STAT3	3
GO:1905562	regulation of vascular endothelial cell proliferation	3/83	48/18670	0.00127	0.0038846	0.00136	PPARG/CCL2/STAT3	3
GO:1901617	organic hydroxy compound biosynthetic process	6/83	271/18670	0.00131	0.0039947	0.0014	CYP3A4/IFNG/FASN/IL1B/TNF/TP53	6
GO:0062014	negative regulation of small molecule metabolic process	4/83	107/18670	0.00132	0.0040266	0.00141	PPARA/STAT3/TGFB1/TP53	4
GO:0001701	in utero embryonic development	7/83	373/18670	0.00136	0.0041064	0.00144	EGFR/IGF2/IL10/MAPK1/NOS3/TP53/VEGFA	7
GO:0010718	positive regulation of epithelial to mesenchymal transition	3/83	50/18670	0.00143	0.0042965	0.0015	IL1B/IL6/TGFB1	3
GO:0014854	response to inactivity	2/83	14/18670	0.00172	0.0050473	0.00177	CAT/IL10	2
GO:0051712	positive regulation of killing of cells of other organism	2/83	14/18670	0.00172	0.0050473	0.00177	IFNG/NOS2	2

GO:1905050	positive regulation of metallopeptidase activity	2/83	14/18670	0.00172	0.0050473	0.00177	MAPK14/STAT3		2
GO:0014706	striated muscle tissue development	7/83	390/18670	0.00175	0.0051272	0.00179	BCL2/FOS/MAPK1/MAPK14/PPARA/TGFB1/VEGFA		7
GO:0072524	pyridine-containing compound metabolic process	5/83	195/18670	0.00177	0.0051978	0.00182	IFNG/PTGS2/PPARA/STAT3/TP53		5
GO:0090287	regulation of cellular response to growth factor stimulus	6/83	292/18670	0.00192	0.0055608	0.00195	XDH/IL1B/PRKCB/TGFB1/TP53/VEGFA		6
GO:0097755	positive regulation of blood vessel diameter	3/83	59/18670	0.0023	0.0064885	0.00227	EGFR/HMOX1/NOS3		3
GO:0006006	glucose metabolic process	5/83	209/18670	0.0024	0.0067436	0.00236	IGF2/MAPK14/PPARA/TNF/TP53		5
GO:0007405	neuroblast proliferation	3/83	61/18670	0.00253	0.007022	0.00246	TGFB1/TP53/VEGFA		3
GO:0033599	regulation of mammary gland epithelial cell proliferation	2/83	17/18670	0.00254	0.007022	0.00246	BAX/CCND1		2
GO:0044872	lipoprotein localization	2/83	17/18670	0.00254	0.007022	0.00246	PPARG/PRKCB		2

GO:1905331	negative regulation of morphogenesis of an epithelium	2/83	17/18670	0.00254	0.007022	0.00246	STAT1/TNF	2
GO:0007204	positive regulation of cytosolic calcium ion concentration	6/83	319/18670	0.00298	0.0080526	0.00282	BAX/BCL2/CXCL10/ESR1/IL2/TGFB1	6
GO:0007006	mitochondrial membrane organization	4/83	134/18670	0.00301	0.0081322	0.00285	BAX/BCL2/STAT3/TP53	4
GO:0060359	response to ammonium ion	4/83	135/18670	0.00309	0.0083249	0.00291	CASP3/HTR3A/MAPK1/RELA	4
GO:1905207	regulation of cardiocyte differentiation	3/83	66/18670	0.00317	0.0084739	0.00297	EGFR/PPARA/TGFB1	3
GO:1905710	positive regulation of membrane permeability	3/83	66/18670	0.00317	0.0084739	0.00297	BAX/BCL2/TP53	3
GO:0035728	response to hepatocyte growth factor	2/83	19/18670	0.00318	0.0084739	0.00297	IL10/RELA	2
GO:0009308	amine metabolic process	4/83	138/18670	0.00334	0.0088905	0.00311	CYP1A1/NQO1/ODC1/VCAM1	4
GO:0097709	connective tissue replacement	2/83	20/18670	0.00352	0.0092971	0.00325	IL1A/TGFB1	2
GO:0044282	small molecule catabolic process	7/83	445/18670	0.00365	0.0096051	0.00336	CYP1A1/NOS2/NOS3/PON1/PPARA/TGFB1/TP53	7
GO:0048285	organelle fission	7/83	449/18670	0.00383	0.0100348	0.00351	CHEK1/EGF/IGF2/IL1A/IL1B/KDR/TGFB1	7

GO:0051900	regulation of mitochondrial depolarization	2/83	21/18670	0.00388	0.0100892	0.00353	BCL2/KDR	2
GO:0051881	regulation of mitochondrial membrane potential	3/83	72/18670	0.00405	0.0104783	0.00367	BAX/BCL2/KDR	3
GO:0044321	response to leptin	2/83	22/18670	0.00426	0.0108652	0.0038	CCND1/STAT3	2
GO:1901685	glutathione derivative metabolic process	2/83	22/18670	0.00426	0.0108652	0.0038	GSTM1/GSTP1	2
GO:1901687	glutathione derivative biosynthetic process	2/83	22/18670	0.00426	0.0108652	0.0038	GSTM1/GSTP1	2
GO:0009692	ethylene metabolic process	1/83	1/18670	0.00445	0.0109276	0.00382	CYP1A1	1
GO:0010477	response to sulfur dioxide	1/83	1/18670	0.00445	0.0109276	0.00382	ICAM1	1
GO:0019341	dibenzo-p-dioxin catabolic process	1/83	1/18670	0.00445	0.0109276	0.00382	CYP1A1	1
GO:0035732	nitric oxide storage	1/83	1/18670	0.00445	0.0109276	0.00382	GSTP1	1
GO:0048673	collateral sprouting of intact axon in response to injury	1/83	1/18670	0.00445	0.0109276	0.00382	SPP1	1

GO:0048683	regulation of collateral sprouting of intact axon in response to injury	1/83	1/18670	0.00445	0.0109276	0.00382	SPP1	1
GO:0048685	negative regulation of collateral sprouting of intact axon in response to injury	1/83	1/18670	0.00445	0.0109276	0.00382	SPP1	1
GO:0060551	regulation of fructose 1,6-bisphosphate metabolic process	1/83	1/18670	0.00445	0.0109276	0.00382	IFNG	1
GO:0060552	positive regulation of fructose 1,6-bisphosphate metabolic process	1/83	1/18670	0.00445	0.0109276	0.00382	IFNG	1
GO:1903788	positive regulation of glutathione biosynthetic process	1/83	1/18670	0.00445	0.0109276	0.00382	NFE2L2	1
GO:1905313	transforming growth factor beta receptor signaling pathway involved in heart development	1/83	1/18670	0.00445	0.0109276	0.00382	TGFB1	1

GO:1905603	regulation of maintenance of permeability of blood-brain barrier	1/83	1/18670	0.00445	0.0109276	0.00382	IL6	1
GO:1990268	response to gold nanoparticle	1/83	1/18670	0.00445	0.0109276	0.00382	MPO	1
GO:0008360	regulation of cell shape	4/83	150/18670	0.0045	0.0110486	0.00387	CCL2/ICAM1/KDR/VEGFA	4
GO:2001039	negative regulation of cellular response to drug	2/83	23/18670	0.00465	0.0113264	0.00396	IL10/NFE2L2	2
GO:0048678	response to axon injury	3/83	77/18670	0.00489	0.0118799	0.00416	BAX/BCL2/SPP1	3
GO:0055074	calcium ion homeostasis	7/83	471/18670	0.00497	0.01203	0.00421	BAX/BCL2/CXCL10/ESR1/IL2/PRKCB/TGFB1	7
GO:0002092	positive regulation of receptor internalization	2/83	24/18670	0.00506	0.0121898	0.00427	EGF/VEGFA	2
GO:0051220	cytoplasmic sequestering of protein	2/83	24/18670	0.00506	0.0121898	0.00427	IL10/NFKBIA	2
GO:0016569	covalent chromatin modification	7/83	474/18670	0.00514	0.0123375	0.00432	CHEK1/IGF2/IL1B/PRKCB/TGFB1/TP53/VEGFA	7
GO:0031639	plasminogen activation	2/83	25/18670	0.00548	0.0130491	0.00457	PLAU/SERPINE1	2

GO:0051894	positive regulation of focal adhesion assembly	2/83	25/18670	0.00548	0.0130491	0.00457	KDR/VEGFA	2
GO:0110110	positive regulation of animal organ morphogenesis	3/83	81/18670	0.00563	0.0133628	0.00468	BAX/TGFB1/VEGFA	3
GO:0006839	mitochondrial transport	5/83	256/18670	0.00568	0.0134386	0.0047	BAX/BCL2/NPEPPS/STAT3/TP53	5
GO:1905048	regulation of metallopeptidase activity	2/83	26/18670	0.00592	0.0139089	0.00487	MAPK14/STAT3	2
GO:0035821	modification of morphology or physiology of other organism	4/83	164/18670	0.00616	0.014424	0.00505	IFNG/NOS2/SLPI/TGFB1	4
GO:1905208	negative regulation of cardiocyte differentiation	2/83	27/18670	0.00638	0.0148416	0.00519	EGFR/PPARA	2
GO:1905563	negative regulation of vascular endothelial cell proliferation	2/83	27/18670	0.00638	0.0148416	0.00519	PPARG/CCL2	2
GO:0001906	cell killing	4/83	168/18670	0.0067	0.015553	0.00544	IFNG/NOS2/ICAM1/IL4	4
GO:0098742	cell-cell adhesion via plasma-membrane adhesion molecules	5/83	273/18670	0.00741	0.0170137	0.00595	CLDN4/ICAM1/IL10/MAPK14/VCAM1	5

GO:0007589	body fluid secretion	3/83	93/18670	0.00825	0.0187358	0.00656	XDH/CCND1/VEGFA	3
GO:0003401	axis elongation	2/83	31/18670	0.00835	0.018866	0.0066	ESR1/TGFB1	2
GO:1903393	positive regulation of adherens junction organization	2/83	31/18670	0.00835	0.018866	0.0066	KDR/VEGFA	2
GO:0031647	regulation of protein stability	5/83	284/18670	0.0087	0.0190919	0.00668	BCL2/CASP3/CDKN1A/MAPK1/TP53	5
GO:0003169	coronary vein morphogenesis	1/83	2/18670	0.00887	0.0190919	0.00668	VEGFA	1
GO:0006788	heme oxidation	1/83	2/18670	0.00887	0.0190919	0.00668	HMOX1	1
GO:0014740	negative regulation of muscle hyperplasia	1/83	2/18670	0.00887	0.0190919	0.00668	NOS3	1
GO:0017143	insecticide metabolic process	1/83	2/18670	0.00887	0.0190919	0.00668	CYP1A1	1
GO:0043006	activation of phospholipase A2 activity by calcium-mediated signaling	1/83	2/18670	0.00887	0.0190919	0.00668	EGFR	1
GO:0046223	aflatoxin catabolic process	1/83	2/18670	0.00887	0.0190919	0.00668	NFE2L2	1
GO:0060057	apoptotic process involved in mammary gland involution	1/83	2/18670	0.00887	0.0190919	0.00668	BAX	1

GO:0060058	positive regulation of apoptotic process involved in mammary gland involution	1/83	2/18670	0.00887	0.0190919	0.00668	BAX	1
GO:0061847	response to cholecystokinin	1/83	2/18670	0.00887	0.0190919	0.00668	CHUK	1
GO:0140039	cell-cell adhesion in response to extracellular stimulus	1/83	2/18670	0.00887	0.0190919	0.00668	VCAM1	1
GO:1901377	organic heteropentacyclic compound catabolic process	1/83	2/18670	0.00887	0.0190919	0.00668	NFE2L2	1
GO:1902336	positive regulation of retinal ganglion cell axon guidance	1/83	2/18670	0.00887	0.0190919	0.00668	VEGFA	1
GO:1902724	positive regulation of skeletal muscle satellite cell proliferation	1/83	2/18670	0.00887	0.0190919	0.00668	STAT3	1
GO:1902728	positive regulation of growth factor dependent skeletal muscle satellite cell proliferation	1/83	2/18670	0.00887	0.0190919	0.00668	STAT3	1

GO:1904057	negative regulation of sensory perception of pain	1/83	2/18670	0.00887	0.0190919	0.00668	IL10	1
GO:1904613	cellular response to 2,3,7,8-tetrachlorodibenzodioxine	1/83	2/18670	0.00887	0.0190919	0.00668	AHR	1
GO:1905179	negative regulation of cardiac muscle tissue regeneration	1/83	2/18670	0.00887	0.0190919	0.00668	CDKN1A	1
GO:0006885	regulation of pH	3/83	98/18670	0.00952	0.0202485	0.00709	BCL2/CA2/MAPK1	3
GO:1905332	positive regulation of morphogenesis of an epithelium	2/83	35/18670	0.01056	0.0223062	0.00781	TGFB1/VEGFA	2
GO:0044272	sulfur compound biosynthetic process	4/83	192/18670	0.0106	0.0223637	0.00783	FASN/GSTM1/GSTP1/NFE2L2	4
GO:0050691	regulation of defense response to virus by host	2/83	37/18670	0.01176	0.0245453	0.00859	IL1B/STAT1	2
GO:0016052	carbohydrate catabolic process	4/83	199/18670	0.01196	0.0249585	0.00873	IFNG/PPARA/STAT3/TP53	4
GO:1905898	positive regulation of response to endoplasmic reticulum stress	2/83	38/18670	0.01238	0.02569	0.00899	BAX/NFE2L2	2

GO:0003340	negative regulation of mesenchymal to epithelial transition involved in metanephros morphogenesis	1/83	3/18670	0.01328	0.0265113	0.00928	STAT1	1
GO:0009822	alkaloid catabolic process	1/83	3/18670	0.01328	0.0265113	0.00928	CYP3A4	1
GO:0033387	putrescine biosynthetic process from ornithine	1/83	3/18670	0.01328	0.0265113	0.00928	ODC1	1
GO:0060523	prostate epithelial cord elongation	1/83	3/18670	0.01328	0.0265113	0.00928	ESR1	1
GO:0060948	cardiac vascular smooth muscle cell development	1/83	3/18670	0.01328	0.0265113	0.00928	VEGFA	1
GO:0061713	anterior neural tube closure	1/83	3/18670	0.01328	0.0265113	0.00928	CASP3	1
GO:0070458	cellular detoxification of nitrogen compound	1/83	3/18670	0.01328	0.0265113	0.00928	GSTM1	1
GO:0072135	kidney mesenchymal cell proliferation	1/83	3/18670	0.01328	0.0265113	0.00928	STAT1	1

GO:0072136	metanephric mesenchymal cell proliferation involved in metanephros development	1/83	3/18670	0.01328	0.0265113	0.00928	STAT1	1
GO:1900673	olefin metabolic process	1/83	3/18670	0.01328	0.0265113	0.00928	CYP1A1	1
GO:1901376	organic heteropentacyclic compound metabolic process	1/83	3/18670	0.01328	0.0265113	0.00928	NFE2L2	1
GO:1901666	positive regulation of NAD+ ADP-ribosyltransferase activity	1/83	3/18670	0.01328	0.0265113	0.00928	TGFB1	1
GO:1902263	apoptotic process involved in embryonic digit morphogenesis	1/83	3/18670	0.01328	0.0265113	0.00928	BAX	1
GO:1903660	negative regulation of complement-dependent cytotoxicity	1/83	3/18670	0.01328	0.0265113	0.00928	IL4	1
GO:0006898	receptor-mediated endocytosis	5/83	316/18670	0.01335	0.0266184	0.00931	CXCL8/EGF/IL4/SERPINE1/VEGFA	5
GO:0051701	interaction with host	4/83	209/18670	0.0141	0.027944	0.00978	CXCL8/EGFR/ICAM1/TGFB1	4

GO:0005976	polysaccharide metabolic process	3/83	114/18670	0.0143	0.028255	0.00989	EGF/IGF2/TGFB1	3
GO:0042752	regulation of circadian rhythm	3/83	114/18670	0.0143	0.028255	0.00989	PPARG/PPARA/TP53	3
GO:2001020	regulation of response to DNA damage stimulus	4/83	214/18670	0.01525	0.0299834	0.01049	BCL2/CHEK1/EGFR/TP53	4
GO:0030866	cortical actin cytoskeleton organization	2/83	43/18670	0.01567	0.0306846	0.01074	IKBKB/TNF	2
GO:0010463	mesenchymal cell proliferation	2/83	44/18670	0.01637	0.0319313	0.01117	STAT1/VEGFA	2
GO:1900274	regulation of phospholipase C activity	2/83	45/18670	0.01708	0.0332256	0.01163	EGFR/ESR1	2
GO:0019730	antimicrobial humoral response	3/83	122/18670	0.01712	0.0332894	0.01165	CXCL10/CXCL8/SLPI	3
GO:0002062	chondrocyte differentiation	3/83	123/18670	0.0175	0.0334352	0.0117	MAPK14/RELA/TGFB1	3
GO:0018916	nitrobenzene metabolic process	1/83	4/18670	0.01767	0.0334352	0.0117	GSTM1	1
GO:0060355	positive regulation of cell adhesion molecule production	1/83	4/18670	0.01767	0.0334352	0.0117	IL1B	1
GO:0060364	frontal suture morphogenesis	1/83	4/18670	0.01767	0.0334352	0.0117	TGFB1	1
GO:0060574	intestinal epithelial cell maturation	1/83	4/18670	0.01767	0.0334352	0.0117	CDKN1A	1

GO:0090170	regulation of Golgi inheritance	1/83	4/18670	0.01767	0.0334352	0.0117	MAPK1	1
GO:0099179	regulation of synaptic membrane adhesion	1/83	4/18670	0.01767	0.0334352	0.0117	MAPK14	1
GO:1902262	apoptotic process involved in blood vessel morphogenesis	1/83	4/18670	0.01767	0.0334352	0.0117	BAX	1
GO:0001754	eye photoreceptor cell differentiation	2/83	46/18670	0.01781	0.0335629	0.01174	STAT3/VEGFA	2
GO:0043330	response to exogenous dsRNA	2/83	46/18670	0.01781	0.0335629	0.01174	MAPK1/NFKBIA	2
GO:0097720	calcineurin-mediated signaling	2/83	46/18670	0.01781	0.0335629	0.01174	NFATC1/TNF	2
GO:0030865	cortical cytoskeleton organization	2/83	48/18670	0.0193	0.036153	0.01265	IKBKB/TNF	2
GO:0016311	dephosphorylation	6/83	478/18670	0.01966	0.036798	0.01288	IKBKB/IFNG/BCL2/PON1/TGFB1/TNF	6
GO:0051260	protein homooligomerization	5/83	351/18670	0.0201	0.0374692	0.01311	BAX/CAT/HMOX1/SLC6A4/TP53	5
GO:0002931	response to ischemia	2/83	50/18670	0.02084	0.0386827	0.01354	BCL2/TP53	2
GO:0030260	entry into host cell	3/83	134/18670	0.0219	0.0393095	0.01376	CXCL8/EGFR/ICAM1	3
GO:0044409	entry into host	3/83	134/18670	0.0219	0.0393095	0.01376	CXCL8/EGFR/ICAM1	3

GO:0051806	entry into cell of other organism involved in symbiotic interaction	3/83	134/18670	0.0219	0.0393095	0.01376	CXCL8/EGFR/ICAM1	3
GO:0051828	entry into other organism involved in symbiotic interaction	3/83	134/18670	0.0219	0.0393095	0.01376	CXCL8/EGFR/ICAM1	3
GO:0019049	evasion or tolerance of host defenses by virus	1/83	5/18670	0.02203	0.0393095	0.01376	TGFB1	1
GO:0019659	glucose catabolic process to lactate	1/83	5/18670	0.02203	0.0393095	0.01376	TP53	1
GO:0043504	mitochondrial DNA repair	1/83	5/18670	0.02203	0.0393095	0.01376	TP53	1
GO:0044413	avoidance of host defenses	1/83	5/18670	0.02203	0.0393095	0.01376	TGFB1	1
GO:0044415	evasion or tolerance of host defenses	1/83	5/18670	0.02203	0.0393095	0.01376	TGFB1	1
GO:0046813	receptor-mediated virion attachment to host cell	1/83	5/18670	0.02203	0.0393095	0.01376	ICAM1	1
GO:0051410	detoxification of nitrogen compound	1/83	5/18670	0.02203	0.0393095	0.01376	GSTM1	1

GO:0051832	avoidance of defenses of other organism involved in symbiotic interaction	1/83	5/18670	0.02203	0.0393095	0.01376	TGFB1	1
GO:0051834	evasion or tolerance of defenses of other organism involved in symbiotic interaction	1/83	5/18670	0.02203	0.0393095	0.01376	TGFB1	1
GO:0071672	negative regulation of smooth muscle cell chemotaxis	1/83	5/18670	0.02203	0.0393095	0.01376	GSTP1	1
GO:0071881	adenylate cyclase-inhibiting adrenergic receptor signaling pathway	1/83	5/18670	0.02203	0.0393095	0.01376	ADRA2A	1
GO:0072162	metanephric mesenchymal cell differentiation	1/83	5/18670	0.02203	0.0393095	0.01376	STAT1	1
GO:0072719	cellular response to cisplatin	1/83	5/18670	0.02203	0.0393095	0.01376	HMOX1	1
GO:0042593	glucose homeostasis	4/83	241/18670	0.02251	0.0400713	0.01402	PPARG/ADRA2A/ICAM1/STAT3	4
GO:1905517	macrophage migration	2/83	53/18670	0.02324	0.0411732	0.01441	CCL2/MAPK14	2

GO:0001837	epithelial to mesenchymal transition	3/83	140/18670	0.02453	0.0432889	0.01515	IL1B/IL6/TGFB1	3
GO:0120192	tight junction assembly	2/83	56/18670	0.02575	0.0451687	0.01581	IKBKB/TNF	2
GO:0140253	cell-cell fusion	2/83	56/18670	0.02575	0.0451687	0.01581	CXCL10/MAPK14	2
GO:0071322	cellular response to carbohydrate stimulus	3/83	144/18670	0.02638	0.0451687	0.01581	ADRA2A/ICAM1/PRKCB	3
GO:0003100	regulation of systemic arterial blood pressure by endothelin	1/83	6/18670	0.02638	0.0451687	0.01581	NOS3	1
GO:0045917	positive regulation of complement activation	1/83	6/18670	0.02638	0.0451687	0.01581	IL1B	1
GO:0060440	trachea formation	1/83	6/18670	0.02638	0.0451687	0.01581	MAPK1	1
GO:0061304	retinal blood vessel morphogenesis	1/83	6/18670	0.02638	0.0451687	0.01581	CYP1B1	1
GO:0071895	odontoblast differentiation	1/83	6/18670	0.02638	0.0451687	0.01581	SERPINE1	1
GO:0097475	motor neuron migration	1/83	6/18670	0.02638	0.0451687	0.01581	VEGFA	1
GO:0110112	regulation of lipid transporter activity	1/83	6/18670	0.02638	0.0451687	0.01581	PPARG	1
GO:1905178	regulation of cardiac muscle tissue regeneration	1/83	6/18670	0.02638	0.0451687	0.01581	CDKN1A	1

GO:2000259	positive regulation of protein activation cascade	1/83	6/18670	0.02638	0.0451687	0.01581	IL1B	1
GO:0090257	regulation of muscle system process	4/83	259/18670	0.02835	0.0482429	0.01688	PTGS2/ADRA2A/NOS3/PPARA	4
GO:0120193	tight junction organization	2/83	60/18670	0.02926	0.0496198	0.01736	IKBKB/TNF	2

ID	Description	GeneRatio	BgRatio	pvalue	p.adjust	qvalue	geneID	Count
GO:0031983	vesicle lumen	13/83	339/19717	1.64E-09	3.55E-07	2.55E-07	ALOX5/MPO/CAT/EGF/EGFR/GSTP1/IGF2/MAPK1/MAPK14/SERPINE1/SLPI/TGFB1/VEGFA	13
GO:0034774	secretory granule lumen	12/83	321/19717	9.73E-09	1.06E-06	7.58E-07	ALOX5/MPO/CAT/EGF/GSTP1/IGF2/MAPK1/MAPK14/SERPINE1/SLPI/TGFB1/VEGFA	12
GO:0045121	membrane raft	11/83	315/19717	8.44E-08	3.78E-06	2.71E-06	IKBKB/PTGS2/CASP3/EGFR/HMOX1/ICAM1/KDR/MAPK1/NOS3/SLC6A4/TNF	11
GO:0098857	membrane microdomain	11/83	316/19717	8.71E-08	3.78E-06	2.71E-06	IKBKB/PTGS2/CASP3/EGFR/HMOX1/ICAM1/KDR/MAPK1/NOS3/SLC6A4/TNF	11
GO:0044798	nuclear transcription factor complex	7/83	201/19717	2.22E-05	0.000494	0.000355	NR1I2/PPARG/FOS/NFATC1/PPARA/STAT3/TP53	7
GO:0005667	transcription factor complex	9/83	365/19717	2.28E-05	0.000494	0.000355	NR1I2/PPARG/AHR/FOS/NFATC1/PPARA/RELA/STAT3/TP53	9
GO:0005901	caveola	4/83	80/19717	0.00036	0.005611	0.004028	PTGS2/HMOX1/MAPK1/NOS3	4
GO:1902554	serine/threonine protein kinase complex	4/83	88/19717	0.00052	0.007527	0.005404	CHUK/IKBKB/CCND1/CDKN1A	4

GO:0008385	IkappaB kinase complex	2/83	9/19717	0.00062	0.008386	0.00602	CHUK/IKBKB	2
GO:0035631	CD40 receptor complex	2/83	11/19717	0.00094	0.011992	0.008609	CHUK/IKBKB	2
GO:0000790	nuclear chromatin	7/83	377/19717	0.00105	0.012598	0.009044	ESR1/IRF1/NFATC1/RELA/STAT1/STAT3/TP53	7
GO:0046930	pore complex	2/83	23/19717	0.00418	0.035134	0.025223	BAX/BCL2	2
GO:0034752	cytosolic aryl hydrocarbon receptor complex	1/83	1/19717	0.00421	0.035134	0.025223	AHR	1
GO:0097144	BAX complex	1/83	1/19717	0.00421	0.035134	0.025223	BAX	1
GO:0005902	microvillus	3/83	83/19717	0.00519	0.040202	0.028862	CA2/TGFB1/VCAM1	3
GO:0045177	apical part of cell	6/83	384/19717	0.00562	0.042072	0.030205	CA2/CLDN4/DUOX2/EGFR/PLB1/VCAM1	6
GO:0005925	focal adhesion	6/83	405/19717	0.00723	0.045579	0.032722	CAT/EGFR/ICAM1/MAPK1/PLAU/SLC6A4	6
GO:0062023	collagen-containing extracellular matrix	6/83	406/19717	0.00732	0.045579	0.032722	ICAM1/MMP2/MMP9/SERPINE1/SLPI/TGFB1	6
GO:0005924	cell-substrate adherens junction	6/83	408/19717	0.00749	0.045579	0.032722	CAT/EGFR/ICAM1/MAPK1/PLAU/SLC6A4	6
GO:0030055	cell-substrate junction	6/83	412/19717	0.00784	0.045579	0.032722	CAT/EGFR/ICAM1/MAPK1/PLAU/SLC6A4	6
GO:0070435	Shc-EGFR complex	1/83	2/19717	0.0084	0.045579	0.032722	EGFR	1

GO:0071065	alpha9-beta1 integrin-vascular cell adhesion molecule-1 complex	1/83	2/19717	0.0084	0.045579	0.032722	VCAM1	1
GO:0097057	TRAF2-GSTP1 complex	1/83	2/19717	0.0084	0.045579	0.032722	GSTP1	1
GO:0099154	serotonergic synapse	1/83	2/19717	0.0084	0.045579	0.032722	SLC6A4	1
GO:1904602	serotonin-activated cation-selective channel complex	1/83	2/19717	0.0084	0.045579	0.032722	HTR3A	1
GO:0009925	basal plasma membrane	2/83	34/19717	0.009	0.047636	0.034199	CLDN4/EGFR	2

ID	Description	GeneRatio	BgRatio	pvalue	p.adjust	qvalue	geneID	Count
hsa04933	AGE-RAGE signaling pathway in diabetic complications	24/78	100/8041	4.62E-28	1.06E-25	3.99E-26	581/596/836/6347/595/3576/3383/3552/3553/3569/5594/1432/4313/4772/4846/5579/5970/5054/6772/6774/7040/7124/7412/7422	24
hsa05418	Fluid shear stress and atherosclerosis	24/78	139/8041	2.38E-24	2.72E-22	1.03E-22	1147/3551/3458/596/6347/2353/2944/2950/3162/3383/3552/3553/3791/1432/4313/4318/4780/4846/1728/5970/7124/7157/7412/7422	24
hsa04657	IL-17 signaling pathway	20/78	94/8041	3.12E-22	2.38E-20	8.99E-21	1147/3551/3458/5743/836/6347/3627/3576/2353/3553/3565/3569/5594/1432/4312/4314/4318/4792/5970/7124	20
hsa04668	TNF signaling pathway	19/78	112/8041	3.83E-19	2.19E-17	8.26E-18	1147/3551/5743/836/6347/3627/2353/3383/3553/3569/3659/5594/1432/4314/4318/4792/5970/7124/7412	19
hsa05161	Hepatitis B	21/78	162/8041	1.35E-18	6.17E-17	2.32E-17	1147/3551/581/596/836/1026/3576/2353/3569/5594/1432/4318/4772/4792/5579/5970/6772/6774/7040/7124/7157	21
hsa05142	Chagas disease (American trypanosomiasis)	18/78	102/8041	1.75E-18	6.67E-17	2.51E-17	1147/3551/3458/4843/6347/3576/2353/3586/3553/3558/3569/5594/1432/4792/5970/5054/7040/7124	18
hsa05140	Leishmaniasis	16/78	77/8041	1.05E-17	3.42E-16	1.29E-16	3458/4843/5743/2353/3586/3552/3553/3565/5594/1432/4792/5579/5970/6772/7040/7124	16
hsa05163	Human cytomegalovirus infection	22/78	225/8041	8.65E-17	2.48E-15	9.33E-16	1147/3551/5743/581/836/6347/595/1026/3576/1956/3553/3569/5594/1432/4772/4792/5579/5970/6774/7124/7157/7422	22

hsa04659	Th17 cell differentiation	17/78	107/8041	1.12E-16	2.84E-15	1.07E-15	1147/3551/3458/196/2353/3553/3558/3565/3569/5594/1432/4772/4792/5970/6772/6774/7040	17
hsa05160	Hepatitis C	19/78	155/8041	2.16E-16	4.95E-15	1.87E-15	1147/3551/3458/581/836/595/1026/1364/3627/1950/1956/5594/4792/5465/5970/6772/6774/7124/7157	19
hsa05145	Toxoplasmosis	17/78	112/8041	2.50E-16	5.20E-15	1.96E-15	240/1147/3551/959/3458/4843/596/836/3586/5594/1432/4792/5970/6772/6774/7040/7124	17
hsa05167	Kaposi sarcoma-associated herpesvirus infection	20/78	189/8041	5.83E-16	1.11E-14	4.20E-15	1147/3551/5743/581/836/595/1026/3576/2353/3383/3569/5594/1432/4772/4792/5970/6772/6774/7157/7422	20
hsa05219	Bladder cancer	12/78	41/8041	1.82E-15	3.20E-14	1.21E-14	595/1026/3576/1950/1956/5594/4312/4313/4318/11186/7157/7422	12
hsa05212	Pancreatic cancer	14/78	76/8041	8.25E-15	1.35E-13	5.09E-14	1147/3551/581/595/1026/1950/1956/5594/5970/6772/6774/7040/7157/7422	14
hsa05215	Prostate cancer	15/78	97/8041	1.28E-14	1.96E-13	7.39E-14	1147/3551/596/595/1026/1950/1956/2950/5594/4314/4318/4792/5328/5970/7157	15
hsa05164	Influenza A	18/78	170/8041	2.04E-14	2.92E-13	1.10E-13	1147/3551/3458/581/836/6347/3627/3576/3383/3552/3553/3569/5594/4792/5579/5970/6772/7124	18
hsa05321	Inflammatory bowel disease (IBD)	13/78	65/8041	2.62E-14	3.52E-13	1.33E-13	3458/3586/3552/3553/3558/3565/3569/4772/5970/6772/6774/7040/7124	13
hsa04625	C-type lectin receptor signaling pathway	15/78	104/8041	3.76E-14	4.78E-13	1.80E-13	1147/3551/5743/3586/3553/3558/3569/3659/5594/1432/4772/4792/5970/6772/7124	15

hsa04066	HIF-1 signaling pathway	15/78	109/8041	7.70E-14	9.28E-13	3.50E-13	3458/4843/596/1026/1950/1956/3162/3569/5594/4846/5579/5970/5054/6774/7422	15
hsa05162	Measles	16/78	138/8041	1.64E-13	1.87E-12	7.06E-13	1147/3551/581/596/836/595/2353/3552/3553/3558/3569/4792/5970/6772/6774/7157	16
hsa05133	Pertussis	13/78	76/8041	2.24E-13	2.44E-12	9.20E-13	4843/836/3576/2353/3586/3552/3553/3569/3659/5594/1432/5970/7124	13
hsa05135	Yersinia infection	15/78	120/8041	3.31E-13	3.44E-12	1.30E-12	1147/3551/6347/3576/2353/3586/3553/3558/3569/5594/1432/4772/4792/5970/7124	15
hsa05205	Proteoglycans in cancer	18/78	205/8041	5.44E-13	5.41E-12	2.04E-12	836/595/1026/1956/2099/3481/3791/5594/1432/4313/4318/5328/5579/6774/7040/7124/7157/7422	18
hsa04064	NF-kappa B signaling pathway	14/78	104/8041	7.74E-13	6.81E-12	2.57E-12	1147/3551/959/5743/596/3576/3383/3553/4792/5328/5579/5970/7124/7412	14
hsa04620	Toll-like receptor signaling pathway	14/78	104/8041	7.74E-13	6.81E-12	2.57E-12	1147/3551/3627/3576/2353/3553/3569/5594/1432/4792/5970/6696/6772/7124	14
hsa04660	T cell receptor signaling pathway	14/78	104/8041	7.74E-13	6.81E-12	2.57E-12	1147/3551/959/3458/2353/3586/3558/3565/5594/1432/4772/4792/5970/7124	14
hsa04380	Osteoclast differentiation	15/78	128/8041	8.70E-13	7.38E-12	2.78E-12	1147/3551/3458/5468/2353/3552/3553/5594/1432/4772/4792/5970/6772/7040/7124	15
hsa05144	Malaria	11/78	50/8041	9.52E-13	7.79E-12	2.94E-12	959/3458/6347/3576/3383/3586/3553/3569/7040/7124/7412	11

hsa05235	PD-L1 expression and PD-1 checkpoint pathway in cancer	13/78	89/8041	1.87E-12	1.47E-11	5.56E-12	1147/3551/3458/1950/1956/2353/5594/1432/4772/4792/5970/6772/6774	13
hsa05323	Rheumatoid arthritis	13/78	93/8041	3.34E-12	2.55E-11	9.62E-12	3458/6347/3576/2353/3383/3552/3553/3569/4312/4314/7040/7124/7422	13
hsa05169	Epstein-Barr virus infection	17/78	201/8041	4.85E-12	3.58E-11	1.35E-11	1147/3551/581/596/836/595/1026/3627/3383/3569/1432/4792/5970/6772/6774/7124/7157	17
hsa05166	Human T-cell leukemia virus 1 infection	17/78	219/8041	1.94E-11	1.39E-10	5.22E-11	1147/3551/581/595/1026/1111/2353/3383/3558/3569/5594/4772/4792/5970/7040/7124/7157	17
hsa04010	MAPK signaling pathway	19/78	294/8041	2.67E-11	1.85E-10	6.99E-11	1147/3551/836/1950/1956/2353/3481/3552/3553/3791/5594/1432/4772/5579/5970/7040/7124/7157/7422	19
hsa04658	Th1 and Th2 cell differentiation	12/78	92/8041	5.65E-11	3.70E-10	1.39E-10	1147/3551/3458/2353/3558/3565/5594/1432/4772/4792/5970/6772	12
hsa05222	Small cell lung cancer	12/78	92/8041	5.65E-11	3.70E-10	1.39E-10	1147/3551/4843/5743/581/596/836/595/1026/4792/5970/7157	12
hsa01522	Endocrine resistance	12/78	98/8041	1.21E-10	7.69E-10	2.90E-10	581/596/595/1026/1956/2099/2353/5594/1432/4313/4318/7157	12
hsa05152	Tuberculosis	15/78	180/8041	1.26E-10	7.83E-10	2.95E-10	3458/4843/581/596/836/3586/3552/3553/3569/5594/1432/5970/6772/7040/7124	15
hsa04926	Relaxin signaling pathway	13/78	129/8041	2.31E-10	1.39E-09	5.24E-10	4843/1956/2353/5594/1432/4312/4313/4318/4792/4846/5970/7040/7422	13
hsa04068	FoxO signaling pathway	13/78	131/8041	2.80E-10	1.65E-09	6.20E-10	1147/3551/847/595/1026/1950/1956/3586/3569/5594/1432/6774/7040	13

hsa05210	Colorectal cancer	11/78	86/8041	4.73E-10	2.71E-09	1.02E-09	581/596/836/595/1026/1950/1956/2353/5594/7040/7157	11
hsa05225	Hepatocellular carcinoma	14/78	168/8041	5.63E-10	3.15E-09	1.19E-09	581/595/1026/1956/2944/2950/3162/3481/5594/4780/1728/5579/7040/7157	14
hsa05223	Non-small cell lung cancer	10/78	68/8041	7.54E-10	4.11E-09	1.55E-09	581/595/1026/1950/1956/5594/5579/11186/6774/7157	10
hsa05143	African trypanosomiasis	8/78	37/8041	1.67E-09	8.91E-09	3.36E-09	3458/3383/3586/3553/3569/5579/7124/7412	8
hsa05220	Chronic myeloid leukemia	10/78	76/8041	2.33E-09	1.21E-08	4.57E-09	1147/3551/581/595/1026/5594/4792/5970/7040/7157	10
hsa05146	Amoebiasis	11/78	102/8041	3.04E-09	1.55E-08	5.84E-09	3458/4843/836/3576/3586/3553/3569/5579/5970/7040/7124	11
hsa04218	Cellular senescence	13/78	160/8041	3.39E-09	1.67E-08	6.30E-09	595/1026/1111/3576/3552/3569/5594/1432/4772/5970/5054/7040/7157	13
hsa01521	EGFR tyrosine kinase inhibitor resistance	10/78	79/8041	3.43E-09	1.67E-08	6.30E-09	581/596/1950/1956/3569/3791/5594/5579/6774/7422	10
hsa05206	MicroRNAs in cancer	17/78	310/8041	4.41E-09	2.10E-08	7.92E-09	1545/3551/5743/596/836/595/1026/1956/3162/5594/4318/5328/5579/11186/6774/7157/7422	17
hsa04151	PI3K-Akt signaling pathway	18/78	354/8041	4.75E-09	2.22E-08	8.37E-09	1147/3551/596/595/1026/1950/1956/3481/3558/3565/3569/3791/5594/4846/5970/6696/7157/7422	18
hsa05130	Pathogenic Escherichia coli infection	14/78	202/8041	6.32E-09	2.90E-08	1.09E-08	1147/3551/581/836/1364/3576/2353/3553/3569/5594/1432/4792/5970/7124	14
hsa05165	Human papillomavirus infection	17/78	330/8041	1.13E-08	5.07E-08	1.91E-08	1147/3551/5743/581/836/595/1026/1950/1956/3659/5594/5970/6696/6772/7124/7157/7422	17

hsa05170	Human immunodeficiency virus 1 infection	14/78	212/8041	1.18E-08	5.19E-08	1.96E-08	1147/3551/581/596/836/1111/2353/5594/1432/4772/4792/5579/5970/7124	14
hsa05132	Salmonella infection	14/78	214/8041	1.33E-08	5.69E-08	2.14E-08	1147/3551/581/596/836/3576/2353/3553/3569/5594/1432/4792/5970/7124	14
hsa01523	Antifolate resistance	7/78	31/8041	1.34E-08	5.69E-08	2.14E-08	1147/3551/9429/3553/3569/5970/7124	7
hsa04621	NOD-like receptor signaling pathway	13/78	181/8041	1.52E-08	6.34E-08	2.39E-08	1147/3551/596/6347/3576/3553/3569/5594/1432/4792/5970/6772/7124	13
hsa04917	Prolactin signaling pathway	9/78	70/8041	1.92E-08	7.83E-08	2.95E-08	595/2099/2353/3659/5594/1432/5970/6772/6774	9
hsa04630	JAK-STAT signaling pathway	12/78	162/8041	4.09E-08	1.64E-07	6.20E-08	3458/596/595/1026/1950/1956/3586/3558/3565/3569/6772/6774	12
hsa04210	Apoptosis	11/78	136/8041	6.39E-08	2.52E-07	9.51E-08	1147/3551/581/596/836/2353/5594/4792/5970/7124/7157	11
hsa04932	Non-alcoholic fatty liver disease (NAFLD)	11/78	149/8041	1.64E-07	6.36E-07	2.40E-07	3551/581/836/3576/3552/3553/3569/5465/5970/7040/7124	11
hsa04622	RIG-I-like receptor signaling pathway	8/78	70/8041	3.16E-07	1.19E-06	4.48E-07	1147/3551/3627/3576/1432/4792/5970/7124	8
hsa05120	Epithelial cell signaling in Helicobacter pylori infection	8/78	70/8041	3.16E-07	1.19E-06	4.48E-07	1147/3551/836/3576/1956/1432/4792/5970	8
hsa04115	p53 signaling pathway	8/78	72/8041	3.95E-07	1.46E-06	5.50E-07	581/596/836/595/1026/1111/5054/7157	8
hsa05131	Shigellosis	13/78	239/8041	4.04E-07	1.47E-06	5.54E-07	1147/3551/581/596/3576/1956/3553/5594/1432/4792/5970/7124/7157	13

hsa01524	Platinum drug resistance	8/78	73/8041	4.40E-07	1.57E-06	5.94E-07	581/596/836/1026/2944/2950/5594/7157	8
hsa05214	Glioma	8/78	75/8041	5.44E-07	1.92E-06	7.22E-07	581/595/1026/1950/1956/5594/5579/7157	8
hsa04931	Insulin resistance	9/78	108/8041	8.57E-07	2.97E-06	1.12E-06	3551/3569/4792/4846/5465/5579/5970/6774/7124	9
hsa04662	B cell receptor signaling pathway	8/78	82/8041	1.09E-06	3.66E-06	1.38E-06	1147/3551/2353/5594/4772/4792/5579/5970	8
hsa05014	Amyotrophic lateral sclerosis (ALS)	7/78	57/8041	1.10E-06	3.66E-06	1.38E-06	581/596/836/847/1432/7124/7157	7
hsa05134	Legionellosis	7/78	57/8041	1.10E-06	3.66E-06	1.38E-06	836/3576/3553/3569/4792/5970/7124	7
hsa05213	Endometrial cancer	7/78	58/8041	1.24E-06	4.05E-06	1.53E-06	581/595/1026/1950/1956/5594/7157	7
hsa05216	Thyroid cancer	6/78	37/8041	1.25E-06	4.05E-06	1.53E-06	5468/581/595/1026/5594/7157	6
hsa04370	VEGF signaling pathway	7/78	59/8041	1.40E-06	4.45E-06	1.68E-06	5743/3791/5594/1432/4846/5579/7422	7
hsa04726	Serotonergic synapse	9/78	115/8041	1.46E-06	4.58E-06	1.72E-06	240/1559/5742/5743/836/3359/5594/5579/6532	9
hsa05330	Allograft rejection	6/78	38/8041	1.48E-06	4.58E-06	1.72E-06	959/3458/3586/3558/3565/7124	6
hsa05202	Transcriptional misregulation in cancer	11/78	186/8041	1.52E-06	4.65E-06	1.75E-06	4353/5468/581/1026/3576/3569/4314/4318/5328/5970/7157	11
hsa04062	Chemokine signaling pathway	11/78	189/8041	1.78E-06	5.37E-06	2.03E-06	1147/3551/6347/3627/3576/5594/4792/5579/5970/6772/6774	11
hsa04071	Sphingolipid signaling pathway	9/78	119/8041	1.95E-06	5.79E-06	2.18E-06	581/596/5594/1432/4846/5579/5970/7124/7157	9
hsa04623	Cytosolic DNA-sensing pathway	7/78	63/8041	2.20E-06	6.46E-06	2.44E-06	1147/3551/3627/3553/3569/4792/5970	7
hsa05332	Graft-versus-host disease	6/78	41/8041	2.35E-06	6.82E-06	2.57E-06	3458/3552/3553/3558/3569/7124	6

hsa05221	Acute myeloid leukemia	7/78	67/8041	3.35E-06	9.60E-06	3.62E-06	1147/3551/4353/595/5594/5970/6774	7
hsa04920	Adipocytokine signaling pathway	7/78	69/8041	4.10E-06	1.16E-05	4.37E-06	1147/3551/4792/5465/5970/6774/7124	7
hsa04060	Cytokine-cytokine receptor interaction	13/78	294/8041	4.17E-06	1.16E-05	4.38E-06	959/3458/6347/3627/3576/3586/3552/3553/3558/3565/3569/7040/7124	13
hsa05218	Melanoma	7/78	72/8041	5.46E-06	1.51E-05	5.68E-06	581/595/1026/1950/1956/5594/7157	7
hsa04672	Intestinal immune network for IgA production	6/78	49/8041	6.88E-06	1.88E-05	7.07E-06	959/3586/3558/3565/3569/7040	6
hsa05224	Breast cancer	9/78	147/8041	1.11E-05	3.00E-05	1.13E-05	581/595/1026/1950/1956/2099/2353/5594/7157	9
hsa05226	Gastric cancer	9/78	149/8041	1.24E-05	3.31E-05	1.25E-05	581/596/595/1026/1950/1956/5594/7040/7157	9
hsa04014	Ras signaling pathway	11/78	232/8041	1.29E-05	3.39E-05	1.28E-05	1147/3551/1950/1956/3481/3791/5594/5579/11186/5970/7422	11
hsa05204	Chemical carcinogenesis	7/78	83/8041	1.41E-05	3.68E-05	1.39E-05	1543/1545/1559/1576/5743/2944/2950	7
hsa04921	Oxytocin signaling pathway	9/78	154/8041	1.62E-05	4.17E-05	1.57E-05	5743/595/1026/1956/2353/5594/4772/4846/5579	9
hsa04722	Neurotrophin signaling pathway	8/78	119/8041	1.79E-05	4.57E-05	1.72E-05	3551/581/596/5594/1432/4792/5970/7157	8
hsa05020	Prion diseases	5/78	35/8041	1.95E-05	4.91E-05	1.85E-05	581/3552/3553/3569/5594	5
hsa05203	Viral carcinogenesis	10/78	204/8041	2.48E-05	6.17E-05	2.33E-05	581/836/595/1026/1111/5594/4792/5970/6774/7157	10

hsa04061	Viral protein interaction with cytokine and cytokine receptor	7/78	100/8041	4.78E-05	0.000118	4.44E-05	6347/3627/3576/3586/3558/3569/7124	7
hsa04915	Estrogen signaling pathway	8/78	138/8041	5.24E-05	0.000128	4.81E-05	596/1956/2099/2353/5594/4313/4318/4846	8
hsa04940	Type I diabetes mellitus	5/78	43/8041	5.44E-05	0.000131	4.95E-05	3458/3552/3553/3558/7124	5
hsa00980	Metabolism of xenobiotics by cytochrome P450	6/78	77/8041	9.41E-05	0.000224	8.46E-05	1543/1545/1559/1576/2944/2950	6
hsa04670	Leukocyte transendothelial migration	7/78	113/8041	0.0001043	0.000246	9.28E-05	1364/3383/1432/4313/4318/5579/7412	7
hsa04510	Focal adhesion	9/78	201/8041	0.0001307	0.000305	0.00012	596/595/1950/1956/3791/5594/5579/6696/7422	9
hsa04217	Necroptosis	8/78	159/8041	0.000142	0.000329	0.00012	3458/581/596/3552/3553/6772/6774/7124	8
hsa05310	Asthma	4/78	31/8041	0.0002113	0.000484	0.00018	959/3586/3565/7124	4
hsa05168	Herpes simplex virus 1 infection	14/78	491/8041	0.0002283	0.000518	0.0002	1147/3551/3458/581/596/836/6347/3553/3569/4792/5970/6772/7124/7157	14
hsa04650	Natural killer cell mediated cytotoxicity	7/78	131/8041	0.0002621	0.000589	0.00022	3458/836/3383/5594/4772/5579/7124	7
hsa00590	Arachidonic acid metabolism	5/78	63/8041	0.0003418	0.00076	0.00029	240/1559/5742/5743/151056	5
hsa04664	Fc epsilon RI signaling pathway	5/78	68/8041	0.0004882	0.001075	0.00041	240/3565/5594/1432/7124	5

hsa04928	Parathyroid hormone synthesis, secretion and action	6/78	106/8041	0.0005421	0.001182	0.00045	596/1026/1956/2353/5594/5579	6
hsa05010	Alzheimer disease	11/78	369/8041	0.0007991	0.001726	0.00065	1147/3551/4843/5743/836/3552/3553/3569/5594/5970/7124	11
hsa00983	Drug metabolism - other enzymes	5/78	79/8041	0.0009716	0.002079	0.00078	7498/1576/4353/2944/2950	5
hsa04935	Growth hormone synthesis, secretion and action	6/78	119/8041	0.0009984	0.002117	0.0008	2353/5594/1432/5579/6772/6774	6
hsa04919	Thyroid hormone signaling pathway	6/78	121/8041	0.001089	0.002288	0.00086	595/2099/5594/5579/6772/7157	6
hsa04012	ErbB signaling pathway	5/78	85/8041	0.0013516	0.002814	0.00106	1026/1950/1956/5594/5579	5
hsa04913	Ovarian steroidogenesis	4/78	51/8041	0.0014493	0.00299	0.00113	240/1543/1545/5743	4
hsa04211	Longevity regulating pathway	5/78	89/8041	0.0016597	0.003392	0.00128	5468/581/847/5970/7157	5
hsa05320	Autoimmune thyroid disease	4/78	53/8041	0.0016736	0.003392	0.00128	959/3586/3558/3565	4
hsa04912	GnRH signaling pathway	5/78	93/8041	0.0020165	0.004051	0.00153	1956/5594/1432/4313/5579	5
hsa04371	Apelin signaling pathway	6/78	137/8041	0.0020606	0.004103	0.00155	4843/595/5594/4846/5054/6696	6
hsa05231	Choline metabolism in cancer	5/78	98/8041	0.0025379	0.00501	0.00189	1950/1956/2353/5594/5579	5
hsa05416	Viral myocarditis	4/78	60/8041	0.002648	0.005148	0.00194	959/836/595/3383	4
hsa04640	Hematopoietic cell lineage	5/78	99/8041	0.0026529	0.005148	0.00194	3552/3553/3565/3569/7124	5

hsa00591	Linoleic acid metabolism	3/78	29/8041	0.002676	0.00515	0.00194	1559/1576/151056	3
hsa04215	Apoptosis - multiple species	3/78	32/8041	0.0035574	0.006789	0.00256	581/596/836	3
hsa04015	Rap1 signaling pathway	7/78	210/8041	0.0041226	0.007802	0.00294	1950/1956/3791/5594/1432/5579/7422	7
hsa05211	Renal cell carcinoma	4/78	69/8041	0.0043962	0.008252	0.00311	1026/5594/7040/7422	4
hsa00982	Drug metabolism - cytochrome P450	4/78	72/8041	0.0051186	0.00953	0.00359	1559/1576/2944/2950	4

Table S5: GO and KEGG enrichment of the 10 hub genes.

Category	Term	Count	%	PValue	Genes	List Total	Pop Hits	Pop Total	Fold Enrichment	Bonferroni
KEGG_PATHWAY	hsa05144:Malaria	7	70	7.87E-12	ICAM1, IL6, TNF, CCL2, CXCL8, IL1B, IL10	10	49	6879	98.27142857	5.82E-10
KEGG_PATHWAY	hsa05321:Inflammatory bowel disease (IBD)	7	70	4.20E-11	IL4, IL6, TNF, IL1B, IL10, STAT3, IL2	10	64	6879	75.2390625	3.11E-09
GOTERM_BP_DIRECT	GO:0006955~immune response	8	80	2.04E-10	IL4, IL6, TNF, CCL2, CXCL8, IL1B, IL10, IL2	10	421	16792	31.9087886	1.02E-07
KEGG_PATHWAY	hsa05142:Chagas disease (American trypanosomiasis)	7	70	8.37E-10	IL6, TNF, CCL2, CXCL8, IL1B, IL10, IL2	10	104	6879	46.30096154	6.19E-08
GOTERM_BP_DIRECT	GO:0071222~cellular response to lipopolysaccharide	6	60	1.56E-09	ICAM1, IL6, TNF, CCL2, CXCL8, IL10	10	113	16792	89.16106195	7.78E-07
KEGG_PATHWAY	hsa04060:Cytokine-cytokine receptor interaction	8	80	2.14E-09	IL4, IL6, TNF, CCL2, CXCL8, IL1B, IL10, IL2	10	243	6879	22.64691358	1.58E-07
GOTERM_CC_DIRECT	GO:0005615~extracellular space	9	90	7.35E-09	IL4, ICAM1, IL6, TNF, CCL2, CXCL8, IL1B, IL10, IL2	10	1347	18224	12.17639198	2.57E-07
GOTERM_MF_DIRECT	GO:0005125~cytokine activity	6	60	1.42E-08	IL4, IL6, TNF, IL1B, IL10, IL2	10	176	16881	57.54886364	6.10E-07

KEGG_PATHWAY	hsa05323:Rheumatoid arthritis	6	60	3.70E-08	ICAM1, IL6, TNF, CCL2, CXCL8, IL1B	10	88	6879	46.90227273	2.74E-06
KEGG_PATHWAY	hsa05143:African trypanosomiasis	5	50	5.44E-08	ICAM1, IL6, TNF, IL1B, IL10	10	33	6879	104.2272727	4.02E-06
GOTERM_BP_DIRECT	GO:0045471~response to ethanol	5	50	1.78E-07	IL4, ICAM1, CCL2, STAT3, IL2	10	105	16792	79.96190476	8.88E-05
GOTERM_BP_DIRECT	GO:0051091~positive regulation of sequence-specific DNA binding transcription factor activity	5	50	1.78E-07	IL4, IL6, TNF, IL1B, IL10	10	105	16792	79.96190476	8.88E-05
KEGG_PATHWAY	hsa04621:NOD-like receptor signaling pathway	5	50	4.82E-07	IL6, TNF, CCL2, CXCL8, IL1B	10	56	6879	61.41964286	3.56E-05
GOTERM_BP_DIRECT	GO:0006954~inflammatory response	6	60	6.67E-07	IL6, TNF, CCL2, CXCL8, IL1B, IL10	10	379	16792	26.58364116	3.34E-04
KEGG_PATHWAY	hsa05164:Influenza A	6	60	1.14E-06	ICAM1, IL6, TNF, CCL2, CXCL8, IL1B	10	174	6879	23.72068966	8.40E-05
GOTERM_CC_DIRECT	GO:0005576~extracellular region	8	80	1.27E-06	IL4, IL6, TNF, CCL2, CXCL8, IL1B, IL10, IL2	10	1610	18224	9.055403727	4.46E-05

GOTERM_BP_DIRECT	GO:0045429~positive regulation of nitric oxide biosynthetic process	4	40	1.30E-06	ICAM1, IL6, TNF, IL1B	10	43	16792	156.2046512	6.50E-04
KEGG_PATHWAY	hsa05133:Pertussis	5	50	1.58E-06	IL6, TNF, CXCL8, IL1B, IL10	10	75	6879	45.86	1.17E-04
GOTERM_BP_DIRECT	GO:0045944~positive regulation of transcription from RNA polymerase II promoter	7	70	2.82E-06	IL4, IL6, TNF, IL1B, IL10, STAT3, IL2	10	981	16792	11.98205912	0.001411278
GOTERM_BP_DIRECT	GO:0045893~positive regulation of transcription, DNA-templated	6	60	3.03E-06	IL4, IL6, TNF, IL1B, IL10, STAT3	10	515	16792	19.56349515	0.001512996
GOTERM_BP_DIRECT	GO:0071407~cellular response to organic cyclic compound	4	40	3.41E-06	TNF, CCL2, IL1B, STAT3	10	59	16792	113.8440678	0.001703257
GOTERM_BP_DIRECT	GO:0071347~cellular response to interleukin-1	4	40	5.97E-06	ICAM1, IL6, CCL2, CXCL8	10	71	16792	94.6028169	0.002983
KEGG_PATHWAY	hsa05146:Amoebiasis	5	50	6.33E-06	IL6, TNF, CXCL8, IL1B, IL10	10	106	6879	32.44811321	4.68E-04

KEGG_PATHWAY	hsa04668:TNF signaling pathway	5	50	6.57E-06	ICAM1, IL6, TNF, CCL2, IL1B	10	107	6879	32.14485981	4.86E-04
KEGG_PATHWAY	hsa05332:Graft-versus-host disease	4	40	8.29E-06	IL6, TNF, IL1B, IL2	10	33	6879	83.38181818	6.13E-04
GOTERM_BP_DIRECT	GO:0045080~positive regulation of chemokine biosynthetic process	3	30	1.15E-05	IL4, TNF, IL1B	10	10	16792	503.76	0.005716476
KEGG_PATHWAY	hsa05330:Allograft rejection	4	40	1.18E-05	IL4, TNF, IL10, IL2	10	37	6879	74.36756757	8.71E-04
GOTERM_BP_DIRECT	GO:0042493~response to drug	5	50	1.24E-05	IL4, ICAM1, IL6, IL10, STAT3	10	304	16792	27.61842105	0.006157518
GOTERM_BP_DIRECT	GO:0034116~positive regulation of heterotypic cell-cell adhesion	3	30	1.40E-05	TNF, IL1B, IL10	10	11	16792	457.9636364	0.006980435
KEGG_PATHWAY	hsa05162:Measles	5	50	1.56E-05	IL4, IL6, IL1B, STAT3, IL2	10	133	6879	25.86090226	0.001154686
KEGG_PATHWAY	hsa04630:Jak-STAT signaling pathway	5	50	2.20E-05	IL4, IL6, IL10, STAT3, IL2	10	145	6879	23.72068966	0.001625566
GOTERM_BP_DIRECT	GO:0071356~cellular response to tumor necrosis factor	4	40	2.23E-05	ICAM1, IL6, CCL2, CXCL8	10	110	16792	61.06181818	0.011101174

KEGG_PATHWAY	hsa04672:Intestinal immune network for IgA production	4	40	2.44E-05	IL4, IL6, IL10, IL2	10	47	6879	58.54468085	0.001804166
GOTERM_BP_DIRECT	GO:0008285~negative regulation of cell proliferation	5	50	3.49E-05	IL6, CXCL8, IL1B, IL10, STAT3	10	396	16792	21.2020202	0.01732211
KEGG_PATHWAY	hsa05134:Legionellosis	4	40	3.72E-05	IL6, TNF, CXCL8, IL1B	10	54	6879	50.95555556	0.002745883
GOTERM_BP_DIRECT	GO:0019221~cytokine-mediated signaling pathway	4	40	3.77E-05	IL6, CCL2, IL1B, STAT3	10	131	16792	51.27328244	0.018656028
GOTERM_BP_DIRECT	GO:0051092~positive regulation of NF-kappaB transcription factor activity	4	40	3.94E-05	ICAM1, IL6, TNF, IL1B	10	133	16792	50.50225564	0.019511452
GOTERM_BP_DIRECT	GO:0042832~defense response to protozoan	3	30	4.35E-05	IL4, IL6, IL10	10	19	16792	265.1368421	0.021496428
GOTERM_BP_DIRECT	GO:0043066~negative regulation of apoptotic process	5	50	6.02E-05	IL4, IL6, IL10, STAT3, IL2	10	455	16792	18.45274725	0.029634188
GOTERM_MF_DIRECT	GO:0008083~growth factor activity	4	40	6.99E-05	IL4, IL6, IL10, IL2	10	162	16881	41.68148148	0.002999149
GOTERM_BP_DIRECT	GO:0007568~aging	4	40	7.49E-05	IL6, CCL2, IL10, STAT3	10	165	16792	40.70787879	0.03677355

KEGG_PATHWAY	hsa05140:Leishmaniasis	4	40	8.47E-05	IL4, TNF, IL1B, IL10	10	71	6879	38.75492958	0.006246085
GOTERM_BP_DIRECT	GO:0070374~positive regulation of ERK1 and ERK2 cascade	4	40	8.92E-05	ICAM1, IL6, TNF, CCL2	10	175	16792	38.38171429	0.043645807
GOTERM_BP_DIRECT	GO:2000352~negative regulation of endothelial cell apoptotic process	3	30	9.58E-05	IL4, ICAM1, IL10	10	28	16792	179.9142857	0.046787861
GOTERM_BP_DIRECT	GO:0043200~response to amino acid	3	30	1.18E-04	ICAM1, IL6, CCL2	10	31	16792	162.5032258	0.057197065
GOTERM_CC_DIRECT	GO:0009897~external side of plasma membrane	4	40	1.26E-04	IL4, ICAM1, IL6, TNF	10	213	18224	34.22347418	0.004384981
GOTERM_BP_DIRECT	GO:0031663~lipopolysaccharide-mediated signaling pathway	3	30	1.26E-04	TNF, CCL2, IL1B	10	32	16792	157.425	0.060875581
GOTERM_BP_DIRECT	GO:0043491~protein kinase B signaling	3	30	1.34E-04	TNF, CCL2, IL1B	10	33	16792	152.6545455	0.064656158
GOTERM_BP_DIRECT	GO:0097192~extrinsic apoptotic signaling pathway in absence of ligand	3	30	1.42E-04	IL4, IL1B, IL2	10	34	16792	148.1647059	0.068537352

KEGG_PATHWAY	hsa04064:NF-kappa B signaling pathway	4	40	1.55E-04	ICAM1, TNF, CXCL8, IL1B	10	87	6879	31.62758621	0.011432621
KEGG_PATHWAY	hsa04640:Hematopoietic cell lineage	4	40	1.55E-04	IL4, IL6, TNF, IL1B	10	87	6879	31.62758621	0.011432621
KEGG_PATHWAY	hsa04660:T cell receptor signaling pathway	4	40	2.35E-04	IL4, TNF, IL10, IL2	10	100	6879	27.516	0.017242121
GOTERM_BP_DIRECT	GO:0032755~positive regulation of interleukin-6 production	3	30	2.50E-04	IL6, TNF, IL1B	10	45	16792	111.9466667	0.117429624
GOTERM_BP_DIRECT	GO:0032729~positive regulation of interferon-gamma production	3	30	2.61E-04	TNF, IL1B, IL2	10	46	16792	109.5130435	0.122395467
KEGG_PATHWAY	hsa04620:Toll-like receptor signaling pathway	4	40	2.79E-04	IL6, TNF, CXCL8, IL1B	10	106	6879	25.95849057	0.020456913
GOTERM_BP_DIRECT	GO:0000165~MAPK cascade	4	40	2.94E-04	TNF, CCL2, IL1B, IL2	10	262	16792	25.63664122	0.136826677
GOTERM_BP_DIRECT	GO:0010628~positive regulation of gene expression	4	40	2.94E-04	IL6, TNF, IL1B, STAT3	10	262	16792	25.63664122	0.136826677

GOTERM_BP_DIRECT	GO:0006959~humoral immune response	3	30	4.01E-04	IL6, TNF, CCL2	10	57	16792	88.37894737	0.181862768
GOTERM_BP_DIRECT	GO:0042102~positive regulation of T cell proliferation	3	30	4.45E-04	IL4, IL6, IL1B	10	60	16792	83.96	0.199427294
GOTERM_BP_DIRECT	GO:0051384~response to glucocorticoid	3	30	5.22E-04	IL6, TNF, IL10	10	65	16792	77.50153846	0.229742991
GOTERM_BP_DIRECT	GO:0030593~neutrophil chemotaxis	3	30	5.38E-04	CCL2, CXCL8, IL1B	10	66	16792	76.32727273	0.235945054
KEGG_PATHWAY	hsa05310:Asthma	3	30	6.49E-04	IL4, TNF, IL10	10	30	6879	68.79	0.046940911
KEGG_PATHWAY	hsa05161:Hepatitis B	4	40	7.02E-04	IL6, TNF, CXCL8, STAT3	10	145	6879	18.97655172	0.050643944
KEGG_PATHWAY	hsa04932:Non-alcoholic fatty liver disease (NAFLD)	4	40	7.90E-04	IL6, TNF, CXCL8, IL1B	10	151	6879	18.22251656	0.056833398
KEGG_PATHWAY	hsa05152:Tuberculosis	4	40	0.001254976	IL6, TNF, IL1B, IL10	10	177	6879	15.54576271	0.088739572
KEGG_PATHWAY	hsa04940:Type I diabetes mellitus	3	30	0.001275118	TNF, IL1B, IL2	10	42	6879	49.13571429	0.090098514
KEGG_PATHWAY	hsa05168:Herpes simplex infection	4	40	0.001382244	IL6, TNF, CCL2, IL1B	10	183	6879	15.03606557	0.09729263

GOTERM_BP_DIRECT	GO:0060559~positive regulation of calcidiol 1-monooxygenase activity	2	20	0.001607143	TNF, IL1B	10	3	16792	1119.466667	0.552562178
GOTERM_BP_DIRECT	GO:0032800~receptor biosynthetic process	2	20	0.001607143	TNF, IL10	10	3	16792	1119.466667	0.552562178
GOTERM_BP_DIRECT	GO:0002693~positive regulation of cellular extravasation	2	20	0.001607143	ICAM1, CCL2	10	3	16792	1119.466667	0.552562178
GOTERM_BP_DIRECT	GO:0006935~chemotaxis	3	30	0.001822944	IL4, CCL2, CXCL8	10	122	16792	41.29180328	0.598401954
KEGG_PATHWAY	hsa05320:Autoimmune thyroid disease	3	30	0.001950471	IL4, IL10, IL2	10	52	6879	39.68653846	0.134524166
GOTERM_BP_DIRECT	GO:0030730~sequestering of triglyceride	2	20	0.002142346	TNF, IL1B	10	4	16792	839.6	0.657787094
GOTERM_BP_DIRECT	GO:0071677~positive regulation of mononuclear cell migration	2	20	0.002142346	IL4, TNF	10	4	16792	839.6	0.657787094
GOTERM_BP_DIRECT	GO:0010574~regulation of vascular endothelial growth factor production	2	20	0.002142346	IL6, CCL2	10	4	16792	839.6	0.657787094

GOTERM_BP_DIRECT	GO:0045191~regulation of isotype switching	2	20	0.002142346	IL4, IL10	10	4	16792	839.6	0.657787094
GOTERM_BP_DIRECT	GO:0008360~regulation of cell shape	3	30	0.002391086	ICAM1, IL6, CCL2	10	140	16792	35.98285714	0.69789311
GOTERM_BP_DIRECT	GO:0031622~positive regulation of fever generation	2	20	0.002677295	TNF, IL1B	10	5	16792	671.68	0.738270221
GOTERM_BP_DIRECT	GO:0042092~type 2 immune response	2	20	0.002677295	IL4, IL10	10	5	16792	671.68	0.738270221
GOTERM_BP_DIRECT	GO:0002740~negative regulation of cytokine secretion involved in immune response	2	20	0.003211989	TNF, IL10	10	6	16792	559.7333333	0.799828172
GOTERM_BP_DIRECT	GO:0060664~epithelial cell proliferation involved in salivary gland morphogenesis	2	20	0.003211989	IL6, TNF	10	6	16792	559.7333333	0.799828172
KEGG_PATHWAY	hsa05166:HTLV-I infection	4	40	0.003541918	ICAM1, IL6, TNF, IL2	10	254	6879	10.83307087	0.230925348

GOTERM_BP_DIRECT	GO:1903140~regulation of establishment of endothelial barrier	2	20	0.003746428	TNF, IL1B	10	7	16792	479.7714286	0.84691035
GOTERM_BP_DIRECT	GO:0048304~positive regulation of isotype switching to IgG isotypes	2	20	0.004280612	IL4, IL2	10	8	16792	419.8	0.882920255
GOTERM_BP_DIRECT	GO:0010888~negative regulation of lipid storage	2	20	0.004280612	IL6, TNF	10	8	16792	419.8	0.882920255
GOTERM_BP_DIRECT	GO:0045348~positive regulation of MHC class II biosynthetic process	2	20	0.004280612	IL4, IL10	10	8	16792	419.8	0.882920255
GOTERM_BP_DIRECT	GO:0070102~interleukin-6-mediated signaling pathway	2	20	0.004814542	IL6, STAT3	10	9	16792	373.1555556	0.910461304
GOTERM_BP_DIRECT	GO:0002237~response to molecule of bacterial origin	2	20	0.004814542	CXCL8, IL10	10	9	16792	373.1555556	0.910461304
KEGG_PATHWAY	hsa05132:Salmonella infection	3	30	0.004901231	IL6, CXCL8, IL1B	10	83	6879	24.86385542	0.304818873

GOTERM_BP_DIRECT	GO:0051024~positive regulation of immunoglobulin secretion	2	20	0.005881637	IL6, IL2	10	11	16792	305.3090909	0.947634178
GOTERM_BP_DIRECT	GO:0036499~PERK-mediated unfolded protein response	2	20	0.006414804	CCL2, CXCL8	10	12	16792	279.8666667	0.959954281
GOTERM_BP_DIRECT	GO:0031669~cellular response to nutrient levels	2	20	0.006414804	ICAM1, IL6	10	12	16792	279.8666667	0.959954281
GOTERM_BP_DIRECT	GO:0045019~negative regulation of nitric oxide biosynthetic process	2	20	0.006947716	IL4, IL10	10	13	16792	258.3384615	0.969376324
GOTERM_BP_DIRECT	GO:0035729~cellular response to hepatocyte growth factor stimulus	2	20	0.007480374	IL6, IL10	10	14	16792	239.8857143	0.976581903
GOTERM_BP_DIRECT	GO:0007267~cell-cell signaling	3	30	0.007649246	IL1B, IL10, IL2	10	254	16792	19.83307087	0.978491898
GOTERM_BP_DIRECT	GO:0043122~regulation of I-kappaB kinase/NF-kappaB signaling	2	20	0.008012778	TNF, IL1B	10	15	16792	223.8933333	0.982092336

GOTERM_BP_DIRECT	GO:0051044~positive regulation of membrane protein ectodomain proteolysis	2	20	0.008012778	TNF, IL1B	10	15	16792	223.8933333	0.982092336
KEGG_PATHWAY	hsa04931:Insulin resistance	3	30	0.008181539	IL6, TNF, STAT3	10	108	6879	19.10833333	0.455520065
KEGG_PATHWAY	hsa05145:Toxoplasmosis	3	30	0.008477263	TNF, IL10, STAT3	10	110	6879	18.76090909	0.467403685
GOTERM_BP_DIRECT	GO:0050995~negative regulation of lipid catabolic process	2	20	0.008544928	TNF, IL1B	10	16	16792	209.9	0.986306347
GOTERM_BP_DIRECT	GO:0044130~negative regulation of growth of symbiont in host	2	20	0.008544928	TNF, IL10	10	16	16792	209.9	0.986306347
GOTERM_BP_DIRECT	GO:0048566~embryonic digestive tract development	2	20	0.008544928	TNF, CXCL8	10	16	16792	209.9	0.986306347
GOTERM_BP_DIRECT	GO:0042523~positive regulation of tyrosine phosphorylation of Stat5 protein	2	20	0.009076825	IL4, IL2	10	17	16792	197.5529412	0.989528889
GOTERM_BP_DIRECT	GO:0032722~positive regulation of chemokine production	2	20	0.009076825	IL6, TNF	10	17	16792	197.5529412	0.989528889

GOTERM_BP_DIRECT	GO:0042346~positive regulation of NF-kappaB import into nucleus	2	20	0.011201875	TNF, IL1B	10	21	16792	159.9238095	0.996420544
GOTERM_BP_DIRECT	GO:0046427~positive regulation of JAK-STAT cascade	2	20	0.011732505	IL6, IL10	10	22	16792	152.6545455	0.99726312
KEGG_PATHWAY	hsa05160:Hepatitis C	3	30	0.012219782	TNF, CXCL8, STAT3	10	133	6879	15.51654135	0.597409038
KEGG_PATHWAY	hsa04068:FoxO signaling pathway	3	30	0.012396488	IL6, IL10, STAT3	10	134	6879	15.40074627	0.602703889
GOTERM_BP_DIRECT	GO:0050715~positive regulation of cytokine secretion	2	20	0.013322874	TNF, IL10	10	25	16792	134.336	0.998776708
GOTERM_BP_DIRECT	GO:0032757~positive regulation of interleukin-8 production	2	20	0.013852491	TNF, IL1B	10	26	16792	129.1692308	0.999064722
GOTERM_BP_DIRECT	GO:0042104~positive regulation of activated T cell proliferation	2	20	0.014381856	IL4, IL2	10	27	16792	124.3851852	0.999284936
GOTERM_BP_DIRECT	GO:0032715~negative regulation of interleukin-6 production	2	20	0.014910968	TNF, IL10	10	28	16792	119.9428571	0.999453309

GOTERM_BP_DIRECT	GO:0071549~cellular response to dexamethasone stimulus	2	20	0.015439828	IL6, CCL2	10	29	16792	115.8068966	0.999582043
GOTERM_BP_DIRECT	GO:0050766~positive regulation of phagocytosis	2	20	0.015439828	TNF, IL1B	10	29	16792	115.8068966	0.999582043
GOTERM_BP_DIRECT	GO:0044344~cellular response to fibroblast growth factor stimulus	2	20	0.015968435	CCL2, CXCL8	10	30	16792	111.9466667	0.999680468
GOTERM_BP_DIRECT	GO:0071392~cellular response to estradiol stimulus	2	20	0.01649679	IL6, IL10	10	31	16792	108.3354839	0.999755718
GOTERM_BP_DIRECT	GO:0007259~JAK-STAT cascade	2	20	0.017024892	CCL2, STAT3	10	32	16792	104.95	0.99981325
GOTERM_BP_DIRECT	GO:0046677~response to antibiotic	2	20	0.017024892	IL6, CCL2	10	32	16792	104.95	0.99981325
GOTERM_BP_DIRECT	GO:2001240~negative regulation of extrinsic apoptotic signaling pathway in absence of ligand	2	20	0.019661627	TNF, IL1B	10	37	16792	90.76756757	0.999951247

GOTERM_BP_DIRECT	GO:0042517~positive regulation of tyrosine phosphorylation of Stat3 protein	2	20	0.020188218	IL6, STAT3	10	38	16792	88.37894737	0.999962733
GOTERM_BP_DIRECT	GO:0007165~signal transduction	4	40	0.020198097	CCL2, CXCL8, IL1B, STAT3	10	1161	16792	5.78535745	0.99996292
GOTERM_BP_DIRECT	GO:0006953~acute-phase response	2	20	0.020714559	IL6, STAT3	10	39	16792	86.11282051	0.999971513
GOTERM_BP_DIRECT	GO:0030890~positive regulation of B cell proliferation	2	20	0.020714559	IL4, IL2	10	39	16792	86.11282051	0.999971513
GOTERM_BP_DIRECT	GO:1901215~negative regulation of neuron death	2	20	0.021240648	IL6, STAT3	10	40	16792	83.96	0.999978225
GOTERM_BP_DIRECT	GO:0002548~monocyte chemotaxis	2	20	0.022292072	IL6, CCL2	10	42	16792	79.96190476	0.999987278
GOTERM_BP_DIRECT	GO:0045599~negative regulation of fat cell differentiation	2	20	0.022292072	IL6, TNF	10	42	16792	79.96190476	0.999987278
GOTERM_BP_DIRECT	GO:0030155~regulation of cell adhesion	2	20	0.022817408	ICAM1, CXCL8	10	43	16792	78.10232558	0.999990276
GOTERM_BP_DIRECT	GO:0014823~response to activity	2	20	0.022817408	CCL2, IL10	10	43	16792	78.10232558	0.999990276

KEGG_PATHWAY	hsa04062:Chemokine signaling pathway	3	30	0.023101733	CCL2, CXCL8, STAT3	10	186	6879	11.09516129	0.822641872
GOTERM_BP_DIRECT	GO:0007155~cell adhesion	3	30	0.023632805	ICAM1, CCL2, IL2	10	459	16792	10.9751634	0.999993594
GOTERM_BP_DIRECT	GO:0008284~positive regulation of cell proliferation	3	30	0.024312383	IL6, STAT3, IL2	10	466	16792	10.81030043	0.999995477
GOTERM_BP_DIRECT	GO:0009408~response to heat	2	20	0.025440324	IL6, CCL2	10	48	16792	69.96666667	0.999997464
GOTERM_MF_DIRECT	GO:0008009~chemokine activity	2	20	0.025828822	CCL2, CXCL8	10	49	16881	68.90204082	0.675423643
GOTERM_BP_DIRECT	GO:0014070~response to organic cyclic compound	2	20	0.025964156	IL4, ICAM1	10	49	16792	68.53877551	0.999998061
GOTERM_BP_DIRECT	GO:0048661~positive regulation of smooth muscle cell proliferation	2	20	0.031709807	IL6, TNF	10	60	16792	55.97333333	0.999999899
GOTERM_BP_DIRECT	GO:0046330~positive regulation of JNK cascade	2	20	0.034311493	TNF, IL1B	10	65	16792	51.66769231	0.999999974
GOTERM_BP_DIRECT	GO:0043547~positive regulation of GTPase activity	3	30	0.034778593	ICAM1, CCL2, IL2	10	565	16792	8.916106195	0.999999979
GOTERM_BP_DIRECT	GO:0030183~B cell differentiation	2	20	0.034831084	IL4, IL10	10	66	16792	50.88484848	0.99999998

GOTERM_BP_DIRECT	GO:0050796~regulation of insulin secretion	2	20	0.035350426	TNF, IL1B	10	67	16792	50.12537313	0.999999985
GOTERM_BP_DIRECT	GO:0032868~response to insulin	2	20	0.035350426	IL6, IL10	10	67	16792	50.12537313	0.999999985
GOTERM_BP_DIRECT	GO:0035690~cellular response to drug	2	20	0.036388366	CCL2, IL1B	10	69	16792	48.67246377	0.999999991
GOTERM_BP_DIRECT	GO:0033138~positive regulation of peptidyl-serine phosphorylation	2	20	0.036906963	IL6, TNF	10	70	16792	47.97714286	0.999999993
GOTERM_BP_DIRECT	GO:0070098~chemokine-mediated signaling pathway	2	20	0.037425313	CCL2, CXCL8	10	71	16792	47.30140845	0.999999995
GOTERM_BP_DIRECT	GO:0050729~positive regulation of inflammatory response	2	20	0.038461267	CCL2, IL2	10	73	16792	46.00547945	0.999999997
GOTERM_BP_DIRECT	GO:0050731~positive regulation of peptidyl-tyrosine phosphorylation	2	20	0.04311081	ICAM1, IL6	10	82	16792	40.95609756	1
KEGG_PATHWAY	hsa05020:Prion diseases	2	20	0.043638711	IL6, IL1B	10	34	6879	40.46470588	0.963184801

GOTERM_BP_DIRECT	GO:0051897~positive regulation of protein kinase B signaling	2	20	0.044141324	IL6, TNF	10	84	16792	39.98095238	1
GOTERM_BP_DIRECT	GO:0050830~defense response to Gram-positive bacterium	2	20	0.04465621	IL6, TNF	10	85	16792	39.51058824	1
GOTERM_BP_DIRECT	GO:0006928~movement of cell or subcellular component	2	20	0.045170851	CXCL8, STAT3	10	86	16792	39.05116279	1
GOTERM_BP_DIRECT	GO:0042593~glucose homeostasis	2	20	0.052860938	IL6, STAT3	10	101	16792	33.25148515	1
GOTERM_BP_DIRECT	GO:0000187~activation of MAPK activity	2	20	0.055921525	TNF, IL1B	10	107	16792	31.38691589	1
GOTERM_BP_DIRECT	GO:0045766~positive regulation of angiogenesis	2	20	0.059988631	CXCL8, IL1B	10	115	16792	29.20347826	1
GOTERM_BP_DIRECT	GO:0001934~positive regulation of protein phosphorylation	2	20	0.066060091	TNF, IL1B	10	127	16792	26.44409449	1
KEGG_PATHWAY	hsa05150:Staphylococcus aureus infection	2	20	0.068510167	ICAM1, IL10	10	54	6879	25.47777778	0.994761815
KEGG_PATHWAY	hsa04151:PI3K-Akt signaling pathway	3	30	0.071485709	IL4, IL6, IL2	10	345	6879	5.98173913	0.995866139

KEGG_PATHWAY	hsa04623:Cytosolic DNA-sensing pathway	2	20	0.080728926	IL6, IL1B	10	64	6879	21.496875	0.998028367
KEGG_PATHWAY	hsa04664:Fc epsilon RI signaling pathway	2	20	0.085576397	IL4, TNF	10	68	6879	20.23235294	0.998666756
KEGG_PATHWAY	hsa04622:RIG-I-like receptor signaling pathway	2	20	0.087991602	TNF, CXCL8	10	70	6879	19.65428571	0.998903739
KEGG_PATHWAY	hsa04920:Adipocytokine signaling pathway	2	20	0.087991602	TNF, STAT3	10	70	6879	19.65428571	0.998903739
KEGG_PATHWAY	hsa05200:Pathways in cancer	3	30	0.08979987	IL6, CXCL8, STAT3	10	393	6879	5.251145038	0.999053479
GOTERM_BP_DIRECT	GO:0050776~regulation of immune response	2	20	0.091476749	IL4, ICAM1	10	178	16792	18.86741573	1
GOTERM_CC_DIRECT	GO:0045121~membrane raft	2	20	0.097273751	ICAM1, TNF	10	206	18224	17.69320388	0.97217325
KEGG_PATHWAY	hsa05410:Hypertrophic cardiomyopathy (HCM)	2	20	0.097595816	IL6, TNF	10	78	6879	17.63846154	0.99949918

Benjamini	FDR
5.82E-10	8.24E-09
1.55E-09	4.39E-08
1.02E-07	2.94E-07
2.06E-08	8.76E-07
3.89E-07	2.24E-06
3.95E-08	2.24E-06
2.57E-07	6.51E-06
6.10E-07	1.32E-05

5.47E-07	3.87E-05
6.71E-07	5.69E-05
2.96E-05	2.56E-04
2.96E-05	2.56E-04
5.09E-06	5.04E-04
8.34E-05	9.61E-04
1.05E-05	0.001188
2.23E-05	0.001127651

1.30E-04	0.001871815
1.30E-05	0.001649666
2.35E-04	0.004067387
2.16E-04	0.004360758
2.13E-04	0.004909584
3.32E-04	0.008603759
4.68E-05	0.006620679

4.42E-05	0.006873743
5.11E-05	0.008673056
5.73E-04	0.016509801
6.70E-05	0.012318896
5.61E-04	0.017787405
5.84E-04	0.020172693
8.25E-05	0.016340182
1.08E-04	0.023008352
8.58E-04	0.032145925

1.13E-04	0.02553822
0.001247357	0.050313602
1.62E-04	0.03888411
0.001254695	0.054223729
0.001230762	0.056733958
0.001277468	0.062567049
0.001669835	0.08660158
0.001500701	0.064983448
0.001969991	0.107849138

3.48E-04	0.088583516
0.002228859	0.12844673
0.002279199	0.137912055
0.002673601	0.169487325
0.001463802	0.111155067
0.002727027	0.180726752
0.00278117	0.192322662
0.002835939	0.204274637

6.05E-04	0.162504255
6.05E-04	0.162504255
8.69E-04	0.24570017
0.004792967	0.35912421
0.004823854	0.37531528
9.84E-04	0.291919767
0.005241208	0.422878104
0.005241208	0.422878104

0.00689766	0.576437414
0.007386846	0.638563363
0.008385004	0.748972206
0.008374598	0.772079244
0.002182994	0.67772191
0.002257073	0.732396739
0.002435045	0.82419343
0.003710162	1.305769178
0.003624912	1.326599964
0.003783817	1.437323396

0.024075677	2.289595451
0.024075677	2.289595451
0.024075677	2.289595451
0.026475666	2.593291399
0.005146562	2.022753388
0.030173197	3.04117385
0.030173197	3.04117385
0.030173197	3.04117385

0.030173197	3.04117385
0.032702607	3.388640319
0.035579794	3.78701545
0.035579794	3.78701545
0.041447582	4.527163702
0.041447582	4.527163702
0.009013178	3.645680981

0.046981843	5.261661731
0.05221017	5.990552338
0.05221017	5.990552338
0.05221017	5.990552338
0.057157204	6.713878
0.057157204	6.713878
0.012046284	5.01258623

0.06781708	8.144002813
0.072099723	8.850885334
0.072099723	8.850885334
0.07616968	9.552369657
0.080042396	10.24849669
0.080075428	10.46815075
0.082025504	10.93930702

0.082025504	10.93930702
0.019419423	8.239278676
0.019494707	8.525239643
0.085513106	11.62484096
0.085513106	11.62484096
0.085513106	11.62484096
0.088846204	12.30513848
0.088846204	12.30513848

0.106537477	14.97475379
0.10926173	15.62945867
0.02719413	12.07522184
0.02678399	12.23971345
0.120995775	17.56370848
0.123306186	18.19862898
0.125525546	18.82869678
0.12765914	19.45394871

0.129711854	20.07442127
0.129711854	20.07442127
0.131688207	20.69015074
0.133592391	21.30117307
0.135428295	21.90752399
0.135428295	21.90752399
0.152512395	24.87043831

0.153944544	25.44949564
0.151729223	25.46031857
0.153062092	26.02412409
0.153062092	26.02412409
0.154418129	26.59435728
0.159213621	27.72177102
0.159213621	27.72177102
0.160426933	28.27901754
0.160426933	28.27901754

0.048215555	21.70100365
0.163460582	29.13603279
0.165545058	29.84300448
0.170336999	31.00194386
0.312764434	21.61371442
0.171309381	31.53409104
0.203020013	37.12559854
0.215303304	39.51503911
0.215297378	39.93498018
0.213011433	39.98200147

0.213321687	40.44538635
0.213321687	40.44538635
0.216403777	41.36153221
0.216661218	41.81434697
0.216912137	42.26369181
0.21981997	43.15207704
0.240749123	46.98487541
0.087637512	37.31315805

0.243213504	47.80114906
0.24312795	48.20459674
0.243044596	48.60495004
0.276221558	54.25452141
0.287165355	56.33774128
0.302093685	58.96961733
0.324820636	62.62507588
0.132326692	52.42300887
0.134489139	53.98989411

0.147613497	58.56422214
0.152533108	60.7949306
0.153157238	61.86530337
0.153157238	61.86530337
0.152766543	62.64931314
0.42020697	74.87987711
0.591571893	59.60454435
0.161991535	65.86521177
